# Supplementary figures and images for: Safety of antidepressants in a primary care cohort of adults with obesity and depression
Source: PLoS One. 2021 Jan 29;16(1):e0245722. doi: 10.1371/journal.pone.0245722 (PMC7846000; doi:10.1371/journal.pone.0245722)

**Figure S1: Proportion of people on higher dose (40mg+) citalopram by length of exposure**

**
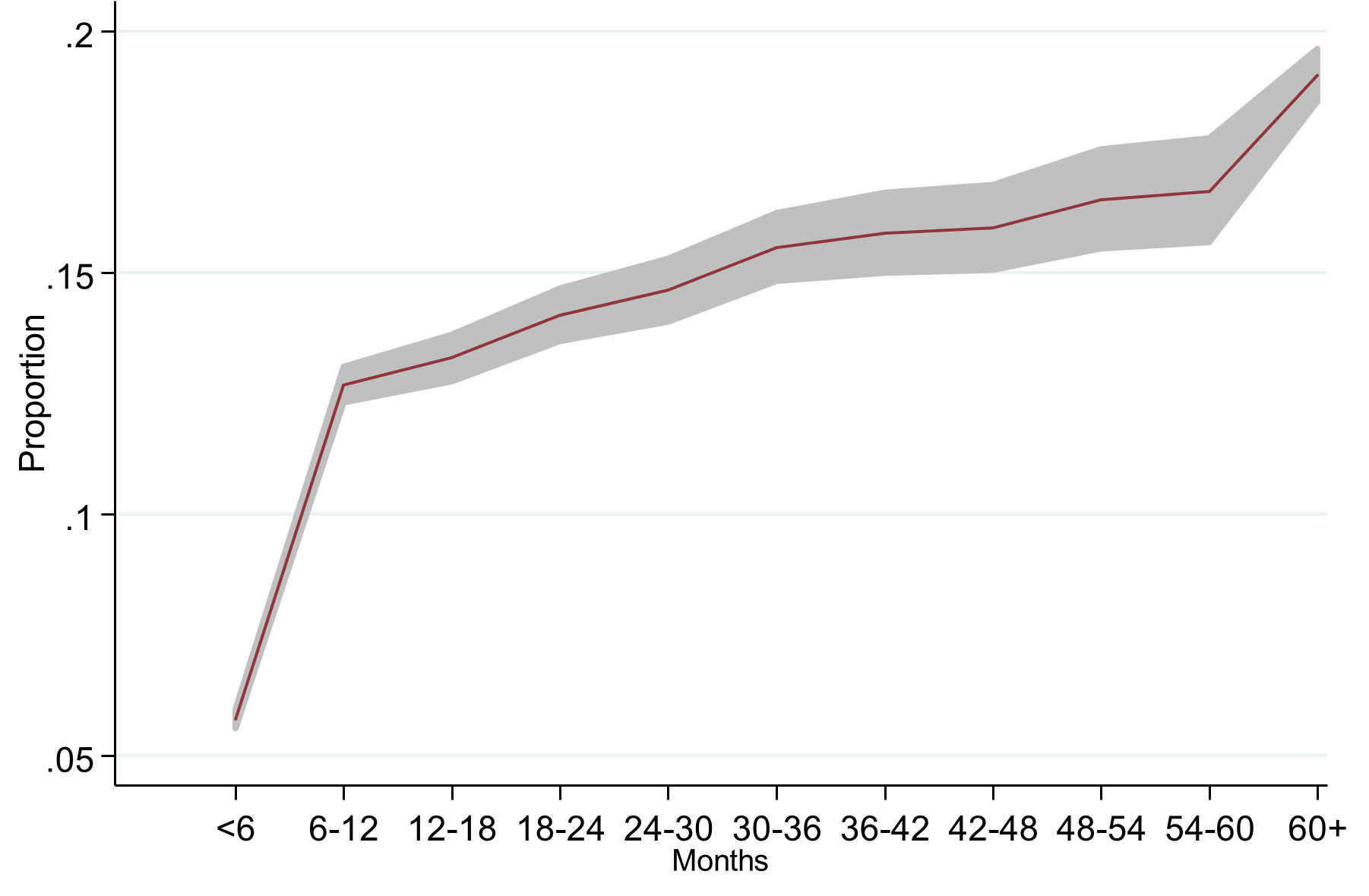
**

Supplement: S1 Fig — (DOCX) [file pone.0245722.s002.docx]

**Figure S2: Proportion of people on higher dose (100mg+) sertraline by length of exposure**

**
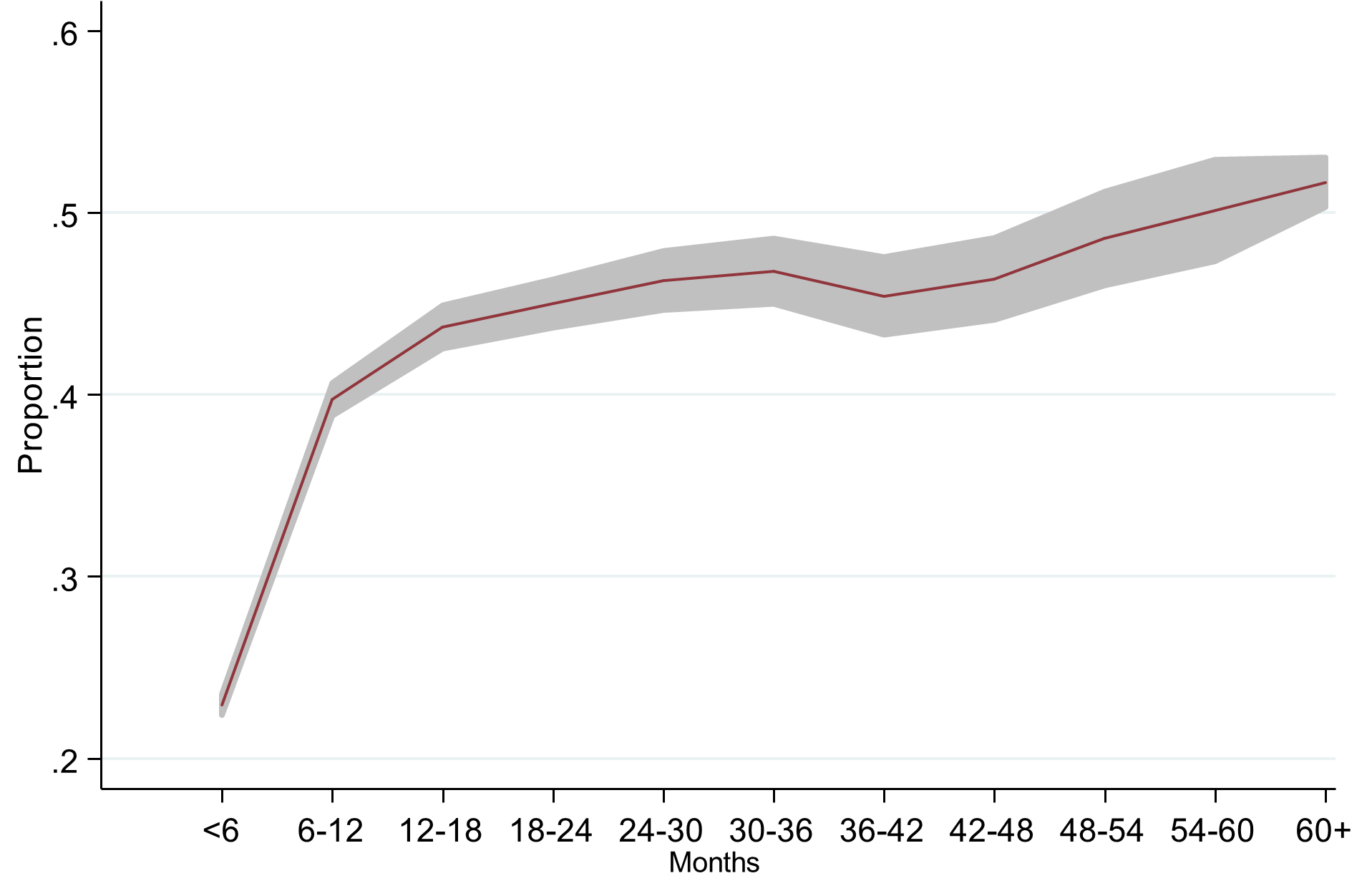
**

Supplement: S2 Fig — (DOCX) [file pone.0245722.s003.docx]
