# Supplementary material for: Safety of antidepressants in a primary care cohort of adults with obesity and depression
Source: PLoS One. 2021 Jan 29;16(1):e0245722. doi: 10.1371/journal.pone.0245722 (PMC7846000; doi:10.1371/journal.pone.0245722)
Supplement: S1 Table — (DOCX) [file pone.0245722.s004.docx]

**Table S1. RECORD* checklist (Reporting of studies conducted using observational routinely-collected data;** [**https://www.record-statement.org/checklist.php**](https://www.record-statement.org/checklist.php)**)**

|  | **Item No.** | **STROBE items** | **Location in manuscript where items are reported** | **RECORD items** | **Location in manuscript where items are reported** |
| --- | --- | --- | --- | --- | --- |
| **Title and Abstract** | | | | | |
|  | 1 | 1. Indicate the study’s design with a commonly used term in the title or the abstract 2. Provide in the abstract an informative and balanced summary of what was done and what was found | 🗹 Cohort listed in **Title** and **Abstract**. Source of data (CPRD) cited in **Abstract**.  🗹 See **Abstract** | RECORD 1.1: The type of data used should be specified in the title or abstract. When possible, the name of the databases used should be included.  RECORD 1.2: If applicable, the geographic region and timeframe within which the study took place should be reported in the title or abstract.  RECORD 1.3: If linkage between databases was conducted for the study, this should be clearly stated in the title or abstract. | 🗹 Source of data (CPRD) cited in **Abstract**  🗹 UK listed in **Abstract**  🗹 Linked hospital and mortality data mentioned in the **Abstract** |
| **Introduction** | | | | | |
| Background rationale | 2 | Explain the scientific background and rationale for the  investigation being reported | 🗹 See **Introduction** |  |  |
| Objectives | 3 | State specific objectives, including any prespecified hypotheses | 🗹 See end of I**ntroduction** for objectives (safety of antidepressants – also stated in **Title**) |  |  |
| **Methods** | | | | | |
| Study Design | 4 | Present key elements of study design early in the paper | 🗹 Sub-headings **Data sources** and **Study design** |  |  |
| Setting | 5 | Describe the setting, locations, and relevant dates, including  periods of recruitment, exposure, follow-up, and data collection | 🗹 Settting and locations (**Data sources**)  🗹 Observation period [recruitment & follow-up] (**Study design**)  🗹 Exposures (**Exposure measures**) |  |  |
| Participants | 6 | 1. *Cohort study* - Give the eligibility criteria, and the sources and methods of selection of participants. Describe methods of follow-up   *Case-control study* - Give the eligibility criteria, and the sources and methods of case ascertainment and control selection. Give the rationale for the choice of cases and controls  *Cross-sectional study* - Give the eligibility criteria, and the sources and methods of selection of participants   1. *Cohort study* For matched studies, give matching criteria and number of exposed and unexposed   *Case-control study* - For matched studies, give matching criteria and the number of controls per case | 🗹 Eligibility criteria (**Study design**)  🗹 Sources (**Data sources**)  🗹 Method of selection (**Data sources** and **Study design**)  🗹 Follow-up (**Statistical analyses**)  N/A | RECORD 6.1: The methods of study population selection (such as codes or algorithms used to identify subjects) should be listed in detail. If this is not possible, an explanation should be provided.  RECORD 6.2: Any validation studies of the codes or algorithms used to select the population should be referenced. If validation was conducted for this study and not published elsewhere, detailed methods and results should be provided.  RECORD 6.3: If the study involved linkage of databases, consider use of a flow diagram or other graphical display to demonstrate the data linkage process, including the number of individuals with linked data at each stage. | 🗹 All codes listed in supplementary material  🗹 Codes were identified from the literature and free-text searching of Read codes but validated by the principal author.  🗷 Information on non-linked data not available because of restrictions with extraction process for the CPRD data |
| Variables | 7 | Clearly define all outcomes, exposures, predictors, potential confounders, and effect modifiers. Give diagnostic criteria, if applicable. | 🗹 Outcomes, exposures and covariates listed under their respective sub-headings and relevant codes provided | RECORD 7.1: A complete list of codes and algorithms used to classify exposures, outcomes, confounders, and effect modifiers should be provided. If these cannot be reported, an explanation should be provided. | 🗹 See supplementary material |
| Data sources/ measurement | 8 | For each variable of interest, give sources of data and details of methods of assessment (measurement).  Describe comparability of assessment methods if there is more than one group | 🗹 See **Exposures**, **Covariate**s and **Outcomes** (and codes provided) |  |  |
| Bias | 9 | Describe any efforts to address potential sources of bias | 🗹 Control for immortal time bias in analysis (**Statistical analyses**) |  |  |
| Study size | 10 | Explain how the study size was arrived at | 🗷 All subjects on the CPRD meeting eligibility criteria were selected. Feasibility counts for sample size included in the cited ISAC protocol number. |  |  |
| Quantitative variables | 11 | Explain how quantitative variables were handled in the analyses. If applicable, describe  which groupings were chosen, and why | 🗹 Groupings for calendar year included (quartiles); BMI categories listed and referenced. |  |  |
| Statistical methods | 12 | 1. Describe all statistical methods, including those used to control for confounding 2. Describe any methods used to examine subgroups and interactions 3. Explain how missing data were addressed 4. *Cohort study* - If applicable, explain how loss to follow-up was addressed   *Case-control study* - If applicable, explain how matching of cases and controls was addressed  *Cross-sectional study* - If applicable, describe analytical methods taking account of sampling strategy   1. Describe any sensitivity analyses | 🗹 See **Statistical analyses**  🗹 Interaction analysis described in the **Sensitivity analyses** results section  🗹 Missing data for covariates treated as a separate category ‘not known’ – specified in sub-heading **Covariates** |  |  |
| Data access and cleaning methods |  | - |  | RECORD 12.1: Authors should describe the extent to which the investigators had access to the database population used to create the study population.  RECORD 12.2: Authors should provide information on the data cleaning methods used in the study. | 🗹 This is an established research database and the authors were not part of this component.  🗹 Some of the data cleaning process (although not all) is described in the supplementary material **Table S2**. |
| Linkage |  | - |  | RECORD 12.3: State whether the study included person-level, institutional-level, or other data linkage across two or more databases. The methods of linkage and methods of linkage quality evaluation should be provided. | 🗹 Person-level linkage was used for this analysis as specified in the methods (**Data sources**) |
| **Results** | | | | | |
| Participants | 13 | 1. Report the numbers of individuals at each stage of the study (*e.g.*, numbers potentially eligible, examined for eligibility, confirmed eligible, included in the study, completing follow-up, and analysed) 2. Give reasons for non- participation at each stage. 3. Consider use of a flow diagram | 🗹 Number of eligible patients included in the results.  The extraction from the CPRD was based on pre-agreed criteria (e.g. linkage available and up-to-standard practices) so information on excluded participants was not available.  🗹 Where available, number of excluded participants is shown in the methods (see **Study Design**).  🗷 Information on non-linked data not available because of restrictions with extraction process for the CPRD data | RECORD 13.1: Describe in detail the selection of the persons included in the study (*i.e.,* study population selection) including filtering based on data quality, data availability and linkage. The selection of included persons can be described in the text and/or by means of the study flow diagram. |  |
| Descriptive data | 14 | 1. Give characteristics of study participants (*e.g.*, demographic, clinical, social) and information on exposures and potential confounders 2. Indicate the number of participants with missing data for each variable of interest 3. *Cohort study* - summarise follow-up time (*e.g.*, average and total amount) | 🗹 Baseline characteristics (**Table 1**)  🗹 Baseline characteristics (**Table 1**)  🗹 Baseline characteristics (**Table 1** – median and range of follow-up given) |  |  |
| Outcome data | 15 | *Cohort study* - Report numbers of outcome events or summary measures over time  *Case-control study* - Report numbers in each exposure category, or summary measures of exposure  *Cross-sectional study* - Report numbers of outcome events or summary measures | 🗹 See heading **Safety of antidepressants** for individual outcomes |  |  |
| Main results | 16 | 1. Give unadjusted estimates and, if applicable, confounder- adjusted estimates and their precision (e.g., 95% confidence interval). Make clear which confounders were adjusted for and why they were included 2. Report category boundaries when continuous variables were categorized 3. If relevant, consider translating estimates of relative risk into absolute risk for a meaningful time period | 🗹 See narrative (under sub-heading **Safety of antidepressants**), **Figure 1** and **Figure 2**.  All potential confounders included and justified in the methods section (**Statistical analyses**)  🗹 see **Table 1**  🗷 Not done |  |  |
| Other analyses | 17 | Report other analyses done— e.g., analyses of subgroups and interactions, and sensitivity analyses | 🗹 see sub-heading **Effect of Time-varying exposures on outcomes** |  |  |
| **Discussion** | | | | | |
| Key results | 18 | Summarise key results with  reference to study objectives | 🗹 See sub-heading **Safety of antidepressants** |  |  |
| Limitations | 19 | Discuss limitations of the study, taking into account sources of potential bias or imprecision.  Discuss both direction and magnitude of any potential bias | 🗹 See limitations paragraph (starting at 5^th^ paragraph) in the **Discussion** | RECORD 19.1: Discuss the implications of using data that were not created or collected to answer the specific research question(s). Include discussion of misclassification bias, unmeasured confounding, missing data, and changing eligibility over time, as they pertain to the study being  reported. | 🗹 Residual and unmeasured confounding (e.g. sleep apnoea, diet, exercise, personality, mental ill-health, severity of depression), misclassification bias (adherence to medication), confounding by indication, selection bias (by BMI measurement) all discussed |
| Interpretation | 20 | Give a cautious overall interpretation of results considering objectives, limitations, multiplicity of analyses, results from similar studies, and other relevant evidence | 🗹 See **Discussion**  and **Conclusions** |  |  |
| Generalisability | 21 | Discuss the generalisability (external validity) of the study results | 🗹 Cautious interpretation comparing the general UK population provided |  |  |
| **Other Information** | | | | | |
| Funding | 22 | Give the source of funding and the role of the funders for the present study and, if applicable, for the original study on which the present article is based | 🗹 See **Acknowledgements** |  |  |
| Accessibility of protocol, raw data, and programming  code |  | .. |  | RECORD 22.1: Authors should provide information on how to access any supplemental information such as the study protocol, raw data, or  programming code. | 🗹 ISAC study protocol listed and available to view on-line. Raw data not available because the authors are not the data controllers. |

*Reference: Benchimol EI, Smeeth L, Guttmann A, Harron K, Moher D, Petersen I, Sørensen HT, von Elm E, Langan SM, the RECORD Working Committee. The REporting of studies Conducted using Observational Routinely-collected health Data (RECORD) Statement. *PLoS Medicine* 2015; 2(10): e1001885. https://doi.org/10.1371/journal.pmed.1001885

*Checklist is protected under Creative Commons Attribution ([CC BY](http://creativecommons.org/licenses/by/4.0/)) licence.
