# Supplementary material for: Safety of antidepressants in a primary care cohort of adults with obesity and depression
Source: PLoS One. 2021 Jan 29;16(1):e0245722. doi: 10.1371/journal.pone.0245722 (PMC7846000; doi:10.1371/journal.pone.0245722)
Supplement: S2 Table — (DOCX) [file pone.0245722.s005.docx]

**Table S2: Read codes for overweight/obesity***

| **OVERWEIGHT**  BMI measurement 25–29 kg/m^2^  Or | | |
| --- | --- | --- |
| **Medical Code** | **Read Code** | **Read Code Description** |
| 103499 | 22AA.00 | Overweight |
| **OBESE**  BMI measurement 30–39 kg/m^2^  Or | | |
| **Medical Code** | **Read Code** | **Read Code Description** |
| 70898 | C38z.00 | Obesity and other hyperalimentation NOS |
| 21744 | 9OK..11 | Obesity clinic administration |
| 40153 | 66CZ.00 | Obesity monitoring NOS |
| 55585 | 9OK6.00 | Obesity monitoring 3rd letter |
| 52036 | 9OK3.00 | Obesity monitoring default |
| 38799 | C380000 | Obesity due to excess calories |
| 110415 | 66CX.00 | Obesity multidisciplinary case review |
| 70950 | 9OK7.00 | Obesity monitoring verbal inv. |
| 49409 | 9OK4.00 | Obesity monitoring 1st letter |
| 66406 | C38..00 | Obesity and other hyperalimentation |
| 11461 | 66C..00 | Obesity monitoring |
| 108610 | 22KD.00 | Obese class II (body mass index 35.0 - 39.9) |
| 103574 | C38y011 | Obesity hypoventilation syndrome |
| 55586 | 9OK5.00 | Obesity monitoring 2nd letter |
| 52735 | 9OKZ.00 | Obesity monitoring admin.NOS |
| 47439 | 9OKA.00 | Obesity monitoring check done |
| 430 | C380.00 | Obesity |
| 32843 | 9OK..00 | Obesity monitoring admin. |
| 67517 | 9OK8.00 | Obesity monitor phone invite |
| 108694 | 22KC.00 | Obese class I (body mass index 30.0 - 34.9) |
| **SEVERELY OBESE**  BMI measurement 40+ kg/m^2^ *  Or | | |
| **Medical Code** | **Read Code** | **Read Code Description** |
| 108478 | 22KE.00 | Obese class III (BMI equal to or greater than 40.0) |

* As part of the data cleaning procedure, we searched for possible errors in coding (BMI>50 kg/m^2^), which involved searching through all previous and subsequent BMI measurements. Where there was an obvious error (BMI change >20 kg/m^2^), we took the closest alternative measurement (n=191). We excluded patients with a BMI >60 kg/m^2^ if there was no other indication in their record that this was correct or if their previous/subsequent BMI measurement indicated that they were of normal weight/underweight (n=184 in total)
