# Supplementary material for: Safety of antidepressants in a primary care cohort of adults with obesity and depression
Source: PLoS One. 2021 Jan 29;16(1):e0245722. doi: 10.1371/journal.pone.0245722 (PMC7846000; doi:10.1371/journal.pone.0245722)
Supplement: S3 Table — (DOCX) [file pone.0245722.s006.docx]

**Table S3. Read codes for depression**

| **Read Code** | **Read Code Description** |
| --- | --- |
| E2B..00 | Depressive disorder NEC |
| Eu32z11 | [X]Depression NOS |
| E112.14 | Endogenous depression |
| E200300 | Anxiety with depression |
| E135.00 | Agitated depression |
| E204.00 | Neurotic depression reactive type |
| E290.00 | Brief depressive reaction |
| 2257 | O/E - depressed |
| 1B17.00 | Depressed |
| E11..12 | Depressive psychoses |
| E204.11 | Postnatal depression |
| 1465 | H/O: depression |
| 62T1.00 | Puerperal depression |
| Eu32z00 | [X]Depressive episode, unspecified |
| E2B0.00 | Postviral depression |
| Eu32z12 | [X]Depressive disorder NOS |
| Eu33.00 | [X]Recurrent depressive disorder |
| E2B1.00 | Chronic depression |
| Eu32.00 | [X]Depressive episode |
| Eu53012 | [X]Postpartum depression NOS |
| E112.11 | Agitated depression |
| Eu32z14 | [X] Reactive depression NOS |
| E113700 | Recurrent depression |
| E112.12 | Endogenous depression first episode |
| Eu32y00 | [X]Other depressive episodes |
| E113.11 | Endogenous depression - recurrent |
| E112.13 | Endogenous depression first episode |
| E112z00 | Single major depressive episode NOS |
| Eu32.13 | [X]Single episode of reactive depression |
| Eu34113 | [X]Neurotic depression |
| Eu41211 | [X]Mild anxiety depression |
| E130.00 | Reactive depressive psychosis |
| Eu34111 | [X]Depressive neurosis |
| Eu33.11 | [X]Recurrent episodes of depressive reaction |
| Eu33.13 | [X]Recurrent episodes of reactive depression |
| Eu32.11 | [X]Single episode of depressive reaction |
| E11z200 | Masked depression |
| Eu32100 | [X]Moderate depressive episode |
| Eu32200 | [X]Severe depressive episode without psychotic symptoms |
| 1B1U.00 | Symptoms of depression |
| 1BT..00 | Depressed mood |
| 1B1U.11 | Depressive symptoms |
| E112.00 | Single major depressive episode |
| Eu32400 | [X]Mild depression |
| Eu32y11 | [X]Atypical depression |
| Eu33212 | [X]Major depression, recurrent without psychotic symptoms |
| Eu33211 | [X]Endogenous depression without psychotic symptoms |
| Eu32000 | [X]Mild depressive episode |
| Eu41200 | [X]Mixed anxiety and depressive disorder |
| Eu32300 | [X]Severe depressive episode with psychotic symptoms |
| 9H91.00 | Depression medication review |
| 9H90.00 | Depression annual review |
| Eu53011 | [X]Postnatal depression NOS |
| E113200 | Recurrent major depressive episodes, moderate |
| E113.00 | Recurrent major depressive episode |
| E112200 | Single major depressive episode, moderate |
| E112300 | Single major depressive episode, severe, without psychosis |
| Eu34114 | [X]Persistant anxiety depression |
| E112100 | Single major depressive episode, mild |
| Eu33315 | [X]Recurrent severe episodes of psychotic depression |
| E130.11 | Psychotic reactive depression |
| Eu32.12 | [X]Single episode of psychogenic depression |
| 6G00.00 | Postnatal depression counselling |
| Eu3y111 | [X]Recurrent brief depressive episodes |
| Eu33.12 | [X]Recurrent episodes of psychogenic depression |
| Eu33400 | [X]Recurrent depressive disorder, currently in remission |
| Eu32212 | [X]Single episode major depression w'out psychotic symptoms |
| Eu33311 | [X]Endogenous depression with psychotic symptoms |
| Eu32313 | [X]Single episode of psychotic depression |
| Eu32311 | [X]Single episode of major depression and psychotic symptoms |
| E113400 | Recurrent major depressive episodes, severe, with psychosis |
| E113z00 | Recurrent major depressive episode NOS |
| E113300 | Recurrent major depressive episodes, severe, no psychosis |
| E11y200 | Atypical depressive disorder |
| Eu32z13 | [X]Prolonged single episode of reactive depression |
| Eu33312 | [X]Manic-depress psychosis,depressed type+psychotic symptoms |
| Eu33.14 | [X]Seasonal depressive disorder |
| Eu32314 | [X]Single episode of reactive depressive psychosis |
| E113100 | Recurrent major depressive episodes, mild |
| Eu33213 | [X]Manic-depress psychosis,depressd,no psychotic symptoms |
| Eu33100 | [X]Recurrent depressive disorder, current episode moderate |
| Eu33000 | [X]Recurrent depressive disorder, current episode mild |
| 9H92.00 | Depression interim review |
| 8CAa.00 | Patient given advice about management of depression |
| 9k4..00 | Depression - enhanced services administration |
| Eu33314 | [X]Recurr severe episodes/psychogenic depressive psychosis |
| E112400 | Single major depressive episode, severe, with psychosis |
| 8HHq.00 | Referral for guided self-help for depression |
| Eu92000 | [X]Depressive conduct disorder |
| Eu33313 | [X]Recurr severe episodes/major depression+psychotic symptom |
| Eu33200 | [X]Recurr depress disorder cur epi severe without psyc sympt |
| E112000 | Single major depressive episode, unspecified |
| E113000 | Recurrent major depressive episodes, unspecified |
| E290z00 | Brief depressive reaction NOS |
| Eu33z11 | [X]Monopolar depression NOS |
| Eu33316 | [X]Recurrent severe episodes/reactive depressive psychosis |
| Eu32211 | [X]Single episode agitated depressn w'out psychotic symptoms |
| 9HA0.00 | On depression register |
| E112500 | Single major depressive episode, partial or unspec remission |
| Eu33z00 | [X]Recurrent depressive disorder, unspecified |
| 8BK0.00 | Depression management programme |
| Eu33300 | [X]Recurrent depress disorder cur epi severe with psyc symp |
| Eu33y00 | [X]Other recurrent depressive disorders |
| 9Ov..00 | Depression monitoring administration |
| Eu32312 | [X]Single episode of psychogenic depressive psychosis |
| E113600 | Recurrent major depressive episodes, in full remission |
| E113500 | Recurrent major depressive episodes,partial/unspec remission |
| Eu32y12 | [X]Single episode of masked depression NOS |
| E112600 | Single major depressive episode, in full remission |
| Eu32213 | [X]Single episode vital depression w'out psychotic symptoms |
| 9k40.00 | Depression - enhanced service completed |
| 9Ov0.00 | Depression monitoring first letter |
| 9Ov1.00 | Depression monitoring second letter |
| Eu33214 | [X]Vital depression, recurrent without psychotic symptoms |
| 9Ov3.00 | Depression monitoring verbal invite |
| ZRLfI00 | Health of the Nation Outcome Scale item 7 - depressed mood |
| 9kQ..00 | On full dose long term treatment depression - enh serv admin |
| Eu32600 | [X]Major depression, moderately severe |
| Eu32500 | [X]Major depression, mild |
| Eu32700 | [X]Major depression, severe without psychotic symptoms |
| Eu32800 | [X]Major depression, severe with psychotic symptoms |
| 8ID..00 | Postnatal depression not discussed |
| Eu32B00 | [X]Antenatal depression |
