# Supplementary material for: Safety of antidepressants in a primary care cohort of adults with obesity and depression
Source: PLoS One. 2021 Jan 29;16(1):e0245722. doi: 10.1371/journal.pone.0245722 (PMC7846000; doi:10.1371/journal.pone.0245722)
Supplement: S4 Table — (DOCX) [file pone.0245722.s007.docx]

**Table S4. Product codes for Antipsychotic and Antimania medications**

| **Antipsychotic Medication** | | |
| --- | --- | --- |
| **Product Code** | **Product Name** | **Drug substance name** |
| 52076 | Amisulpride 12.5mg/5ml oral solution | Amisulpride |
| 4992 | Solian 200 tablets (Sanofi) | Amisulpride |
| 63598 | Amisulpride 400mg tablets (Zentiva) | Amisulpride |
| 16768 | Solian 50 tablets (Sanofi) | Amisulpride |
| 34927 | Amisulpride 200mg tablets (Zentiva) | Amisulpride |
| 55625 | Amisulpride 50mg/5ml oral suspension | Amisulpride |
| 51558 | Amisulpride 12.5mg/5ml oral suspension | Amisulpride |
| 6482 | Amisulpride 100mg/ml oral solution sugar free | Amisulpride |
| 5071 | Amisulpride 200mg tablets | Amisulpride |
| 31576 | Solian 100mg/ml oral solution (Sanofi) | Amisulpride |
| 11938 | Amisulpride 25mg/5ml oral suspension | Amisulpride |
| 41714 | Amisulpride 50mg tablets (Zentiva) | Amisulpride |
| 62202 | Amisulpride 50mg tablets (A A H Pharmaceuticals Ltd) | Amisulpride |
| 41702 | Amisulpride 100mg tablets (Zentiva) | Amisulpride |
| 6524 | Amisulpride 100mg tablets | Amisulpride |
| 26544 | Solian 100 tablets (Sanofi) | Amisulpride |
| 5927 | Amisulpride 400mg tablets | Amisulpride |
| 6109 | Solian 400 tablets (Sanofi) | Amisulpride |
| 46969 | Amisulpride 200mg tablets (A A H Pharmaceuticals Ltd) | Amisulpride |
| 46889 | Amisulpride 25mg/5ml oral solution | Amisulpride |
| 4876 | Amisulpride 50mg tablets | Amisulpride |
| 1453 | Triptafen m 2mg+10mg Tablet (Goldshield Pharmaceuticals Ltd) | Amitriptyline hydrochloride/Perphenazine |
| 6894 | Perphenazine 2mg with Amitriptyline 25mg tablet | Amitriptyline Hydrochloride/Perphenazine |
| 16323 | Perphenazine 2mg with Amitriptyline 10mg tablet | Amitriptyline Hydrochloride/Perphenazine |
| 65773 | Aripiprazole 30mg tablets (Zentiva) | Aripiprazole |
| 71594 | Aripiprazole 7.5mg/ml oral solution | Aripiprazole |
| 32076 | Aripiprazole 10mg orodispersible tablets sugar free | Aripiprazole |
| 57114 | Abilify 5mg tablets (Mawdsley-Brooks & Company Ltd) | Aripiprazole |
| 31098 | Aripiprazole 15mg orodispersible tablets sugar free | Aripiprazole |
| 46705 | Abilify 9.75mg/1.3ml solution for injection vials (Otsuka Pharmaceuticals (U.K.) Ltd) | Aripiprazole |
| 16575 | Aripiprazole 1mg/ml oral solution | Aripiprazole |
| 66479 | Aripiprazole 400mg powder and solvent for suspension for injection pre-filled syringes | Aripiprazole |
| 68152 | Abilify Maintena 400mg powder and solvent for prolonged-release suspension for injection pre-filled syringes (Otsuka Pharmaceuticals (U.K.) Ltd) | Aripiprazole |
| 14858 | Abilify 15mg tablets (Otsuka Pharmaceuticals (U.K.) Ltd) | Aripiprazole |
| 29879 | Abilify 30mg tablets (Otsuka Pharmaceuticals (U.K.) Ltd) | Aripiprazole |
| 6573 | Aripiprazole 15mg tablets | Aripiprazole |
| 38080 | Abilify 1mg/ml oral solution (Otsuka Pharmaceuticals (U.K.) Ltd) | Aripiprazole |
| 6561 | Aripiprazole 10mg tablets | Aripiprazole |
| 38375 | Aripiprazole 9.75mg/1.3ml solution for injection vials | Aripiprazole |
| 14344 | Aripiprazole 5mg tablets | Aripiprazole |
| 37606 | Abilify 10mg orodispersible tablets (Otsuka Pharmaceuticals (U.K.) Ltd) | Aripiprazole |
| 49699 | Abilify 5mg tablets (Sigma Pharmaceuticals Plc) | Aripiprazole |
| 16561 | Aripiprazole 30mg tablets | Aripiprazole |
| 61650 | Aripiprazole 400mg powder and solvent for suspension for injection vials | Aripiprazole |
| 71481 | Aripiprazole 10mg tablets (Alliance Healthcare (Distribution) Ltd) | Aripiprazole |
| 38010 | Abilify 15mg orodispersible tablets (Otsuka Pharmaceuticals (U.K.) Ltd) | Aripiprazole |
| 18132 | Abilify 5mg tablets (Otsuka Pharmaceuticals (U.K.) Ltd) | Aripiprazole |
| 65671 | Aripiprazole 5mg tablets (Zentiva) | Aripiprazole |
| 24358 | Abilify 10mg tablets (Otsuka Pharmaceuticals (U.K.) Ltd) | Aripiprazole |
| 61075 | Abilify Maintena 400mg powder and solvent for prolonged-release suspension for injection vials (Otsuka Pharmaceuticals (U.K.) Ltd) | Aripiprazole |
| 47365 | Anquil 250microgram tablets (Kyowa Kirin Ltd) | Benperidol |
| 21744 | Anquil 250microgram Tablet (Concord Pharmaceuticals Ltd) | Benperidol |
| 2540 | Benperidol 250microgram tablets | Benperidol |
| 31796 | Benquil 250microgram tablets (Concord Pharmaceuticals Ltd) | Benperidol |
| 15418 | Largactil forte 100mg/5ml Oral suspension (Hawgreen Ltd) | Chlorpromazine embonate |
| 67820 | Chlorpromazine 100mg/5ml oral suspension | Chlorpromazine hydrochloride |
| 34668 | Chlorpromazine 25mg tablets (Teva UK Ltd) | Chlorpromazine hydrochloride |
| 19002 | Largactil 100mg Suppository (Rhone-Poulenc Rorer Ltd) | Chlorpromazine Hydrochloride |
| 34630 | Chlorpromazine 50mg tablets (Thornton & Ross Ltd) | Chlorpromazine hydrochloride |
| 17227 | Chloractil 25mg Tablet (DDSA Pharmaceuticals Ltd) | Chlorpromazine hydrochloride |
| 37705 | Chlorpromazine 100mg/5ml suspension | Chlorpromazine Hydrochloride |
| 46960 | Chlorpromazine 100mg tablets (IVAX Pharmaceuticals UK Ltd) | Chlorpromazine hydrochloride |
| 28862 | Chloractil 100mg Tablet (DDSA Pharmaceuticals Ltd) | Chlorpromazine hydrochloride |
| 31171 | Chlorpromazine 50mg tablets (A A H Pharmaceuticals Ltd) | Chlorpromazine hydrochloride |
| 67747 | Chlorpromazine 25mg tablets (Almus Pharmaceuticals Ltd) | Chlorpromazine hydrochloride |
| 60908 | Chlorpromazine 10mg capsules | Chlorpromazine hydrochloride |
| 65246 | Chlorpromazine 10mg/5ml oral suspension | Chlorpromazine hydrochloride |
| 2814 | Largactil 25mg Tablet (Hawgreen Ltd) | Chlorpromazine hydrochloride |
| 4434 | Chlorpromazine 50mg/5ml oral solution | Chlorpromazine hydrochloride |
| 9190 | Chlorpromazine 25mg/5ml oral solution sugar free | Chlorpromazine hydrochloride |
| 44186 | Chlorpromazine 25mg/5ml oral solution (A A H Pharmaceuticals Ltd) | Chlorpromazine hydrochloride |
| 588 | Chlorpromazine 25mg tablets | Chlorpromazine hydrochloride |
| 67247 | Largactil 50mg tablets (Waymade Healthcare Plc) | Chlorpromazine hydrochloride |
| 58702 | Largactil 100mg tablets (Sanofi) | Chlorpromazine hydrochloride |
| 8311 | Chlorpromazine 25mg/ml injection | Chlorpromazine Hydrochloride |
| 10434 | Largactil 25mg/5ml Oral solution (Hawgreen Ltd) | Chlorpromazine hydrochloride |
| 31175 | Chlorpromazine 25mg tablets (A A H Pharmaceuticals Ltd) | Chlorpromazine hydrochloride |
| 34736 | Chlorpromazine 100mg tablets (Teva UK Ltd) | Chlorpromazine hydrochloride |
| 55011 | Largactil 25mg tablets (Sanofi) | Chlorpromazine hydrochloride |
| 58703 | Largactil 50mg tablets (Sanofi) | Chlorpromazine hydrochloride |
| 7514 | Largactil 50mg/2ml solution for injection ampoules (Sanofi) | Chlorpromazine hydrochloride |
| 2154 | Chlorpromazine 100mg tablets | Chlorpromazine hydrochloride |
| 45281 | Chlorpromazine 100mg/5ml oral solution (Rosemont Pharmaceuticals Ltd) | Chlorpromazine hydrochloride |
| 7493 | Largactil 100mg Tablet (Hawgreen Ltd) | Chlorpromazine hydrochloride |
| 31172 | Chlorpromazine 50mg tablets (Teva UK Ltd) | Chlorpromazine hydrochloride |
| 56862 | Chlorpromazine 25mg/5ml syrup (Rosemont Pharmaceuticals Ltd) | Chlorpromazine hydrochloride |
| 2474 | Chlorpromazine 10mg tablets | Chlorpromazine hydrochloride |
| 3348 | Chlorpromazine 50mg tablets | Chlorpromazine hydrochloride |
| 57550 | Largactil 25mg/5ml syrup (Sanofi) | Chlorpromazine hydrochloride |
| 3952 | Chlorpromazine 25mg/5ml oral solution | Chlorpromazine hydrochloride |
| 8519 | Chlorpromazine 100mg/5ml oral solution | Chlorpromazine hydrochloride |
| 8771 | Largactil 10mg Tablet (Hawgreen Ltd) | Chlorpromazine hydrochloride |
| 22606 | Chlorpromazine 25mg/1ml solution for injection ampoules | Chlorpromazine hydrochloride |
| 35929 | Chlorpromazine 50mg/2ml solution for injection ampoules | Chlorpromazine hydrochloride |
| 37871 | Chlorpromazine 25mg/5ml Oral solution (Rosemont Pharmaceuticals Ltd) | Chlorpromazine hydrochloride |
| 34693 | Chlorpromazine 25mg tablets (Thornton & Ross Ltd) | Chlorpromazine hydrochloride |
| 8506 | Chlorpromazine 100mg suppository | Chlorpromazine Hydrochloride |
| 25653 | Chloractil 50mg Tablet (DDSA Pharmaceuticals Ltd) | Chlorpromazine hydrochloride |
| 58492 | Chlorpromazine 100mg tablets (Waymade Healthcare Plc) | Chlorpromazine hydrochloride |
| 3772 | Largactil 50mg Tablet (Hawgreen Ltd) | Chlorpromazine hydrochloride |
| 61153 | Chlorpromazine 25mg tablets (Phoenix Healthcare Distribution Ltd) | Chlorpromazine hydrochloride |
| 41645 | Chlorpromazine 25mg/ml Injection (Antigen Pharmaceuticals) | Chlorpromazine Hydrochloride |
| 31184 | Chlorpromazine 25mg tablets (IVAX Pharmaceuticals UK Ltd) | Chlorpromazine hydrochloride |
| 30111 | Chlorprothixene 50mg tablets | Chlorprothixene |
| 28147 | Taractan 15mg Tablet (Roche Products Ltd) | Chlorprothixene |
| 21199 | Denzapine 100mg tablets (Britannia Pharmaceuticals Ltd) | Clozapine |
| 40586 | Clozapine 50mg tablets | Clozapine |
| 14112 | Clozaril 100mg tablets (Mylan) | Clozapine |
| 41428 | Denzapine 200mg tablets (Britannia Pharmaceuticals Ltd) | Clozapine |
| 42242 | Clozapine 50mg/ml oral suspension sugar free | Clozapine |
| 47233 | Zaponex 25mg tablets (Leyden Delta B.V.) | Clozapine |
| 40587 | Clozapine 200mg tablets | Clozapine |
| 45444 | Denzapine 50mg/ml oral suspension (Britannia Pharmaceuticals Ltd) | Clozapine |
| 8046 | Clozapine 25mg tablets | Clozapine |
| 41070 | Denzapine 50mg tablets (Britannia Pharmaceuticals Ltd) | Clozapine |
| 30487 | Denzapine 25mg tablets (Britannia Pharmaceuticals Ltd) | Clozapine |
| 8047 | Clozapine 100mg tablets | Clozapine |
| 47302 | Zaponex 100mg tablets (Leyden Delta B.V.) | Clozapine |
| 17958 | Clozaril 25mg tablets (Mylan) | Clozapine |
| 15128 | Droperidol 1mg/ml liquid | Droperidol |
| 15171 | Droleptan 10mg Tablet (Janssen-Cilag Ltd) | Droperidol |
| 22609 | Droperidol 5mg/ml injection | Droperidol |
| 13369 | Droleptan 1mg/ml Oral solution (Janssen-Cilag Ltd) | Droperidol |
| 3773 | Droperidol 10mg tablets | Droperidol |
| 21125 | Droleptan 5mg/ml Injection (Janssen-Cilag Ltd) | Droperidol |
| 42229 | Droperidol oral liquid | Droperidol |
| 53634 | Droperidol capsules | Droperidol |
| 14889 | Depixol Conc 100mg/1ml solution for injection ampoules (Lundbeck Ltd) | Flupentixol decanoate |
| 18175 | Flupentixol 100mg/1ml solution for injection ampoules | Flupentixol decanoate |
| 1733 | Flupentixol decanoate 20mg/ml Injection | Flupentixol Decanoate |
| 2276 | Flupentixol 40mg/2ml solution for injection ampoules | Flupentixol decanoate |
| 19283 | Depixol 20mg/1ml solution for injection ampoules (Lundbeck Ltd) | Flupentixol decanoate |
| 57170 | Psytixol 100mg/1ml solution for injection ampoules (Mylan) | Flupentixol decanoate |
| 2136 | Depixol 20mg/ml Injection (Lundbeck Ltd) | Flupentixol Decanoate |
| 18155 | Flupentixol 50mg/0.5ml solution for injection ampoules | Flupentixol decanoate |
| 8712 | Flupentixol decanoate 100mg/ml Injection | Flupentixol Decanoate |
| 60782 | Psytixol 20mg/1ml solution for injection ampoules (Mylan) | Flupentixol decanoate |
| 14966 | Flupentixol 20mg/1ml solution for injection ampoules | Flupentixol decanoate |
| 18197 | Depixol Conc 50mg/0.5ml solution for injection ampoules (Lundbeck Ltd) | Flupentixol decanoate |
| 59816 | Psytixol 50mg/0.5ml solution for injection ampoules (Mylan) | Flupentixol decanoate |
| 2156 | Depixol 40mg/2ml solution for injection ampoules (Lundbeck Ltd) | Flupentixol decanoate |
| 14839 | Flupentixol 200mg/1ml solution for injection ampoules | Flupentixol decanoate |
| 14130 | Depixol Low Volume 200mg/1ml solution for injection ampoules (Lundbeck Ltd) | Flupentixol decanoate |
| 57762 | Psytixol 40mg/2ml solution for injection ampoules (Mylan) | Flupentixol decanoate |
| 2155 | Depixol -conc 100mg/ml Injection (Lundbeck Ltd) | Flupentixol Decanoate |
| 55620 | Flupentixol Liquid | Flupentixol Dihydrochloride |
| 5707 | Flupentixol 3mg tablets | Flupentixol dihydrochloride |
| 5712 | Depixol 3mg tablets (Lundbeck Ltd) | Flupentixol dihydrochloride |
| 35391 | Fluphenazine decanoate 50mg/0.5ml solution for injection ampoules | Fluphenazine decanoate |
| 12128 | Modecate concentrate 100mg/ml Injection (Sanofi-Synthelabo Ltd) | Fluphenazine Decanoate |
| 3926 | Modecate 25mg/ml Injection (Sanofi-Synthelabo Ltd) | Fluphenazine Decanoate |
| 66652 | Decazate 25mg/ml Injection (Berk Pharmaceuticals Ltd) | Fluphenazine Decanoate |
| 35723 | Fluphenazine decanoate 50mg/2ml solution for injection ampoules | Fluphenazine decanoate |
| 35455 | Modecate Concentrate 100mg/1ml solution for injection ampoules (Sanofi) | Fluphenazine decanoate |
| 35065 | Fluphenazine decanoate 25mg/1ml solution for injection ampoules | Fluphenazine decanoate |
| 10514 | Fluphenazine decanoate 100mg/ml Injection | Fluphenazine Decanoate |
| 33780 | Modecate 25mg/1ml solution for injection ampoules (Sanofi) | Fluphenazine decanoate |
| 41970 | Fluphenazine decanoate 25mg/1ml solution for injection ampoules (Hospira UK Ltd) | Fluphenazine decanoate |
| 41971 | Fluphenazine decanoate 25mg/ml Injection (Antigen Pharmaceuticals) | Fluphenazine decanoate |
| 35122 | Modecate 12.5mg/0.5ml solution for injection ampoules (Sanofi) | Fluphenazine decanoate |
| 9022 | Fluphenazine decanoate 25mg/ml Injection | Fluphenazine Decanoate |
| 35176 | Fluphenazine decanoate 100mg/1ml solution for injection ampoules | Fluphenazine decanoate |
| 35487 | Modecate Concentrate 50mg/0.5ml solution for injection ampoules (Sanofi) | Fluphenazine decanoate |
| 35530 | Fluphenazine decanoate 12.5mg/0.5ml solution for injection ampoules | Fluphenazine decanoate |
| 35445 | Modecate 50mg/2ml solution for injection ampoules (Sanofi) | Fluphenazine decanoate |
| 25835 | Moditen enanthate 25mg/ml Injection (Sanofi-Synthelabo Ltd) | Fluphenazine Enantate |
| 17190 | Fluphenazine enanthate 25mg/ml Injection | Fluphenazine Enantate |
| 5212 | Fluphenazine 1mg tablets | Fluphenazine hydrochloride |
| 5597 | Moditen 1mg tablets (Sanofi) | Fluphenazine hydrochloride |
| 10827 | Fluspirilene 2mg/ml Injection | Fluspirilene |
| 8044 | Redeptin 2mg/ml Injection (Janssen-Cilag Ltd) | Fluspirilene |
| 9975 | Haloperidol 1mg/ml sugar free Oral solution | Haloperidol |
| 28679 | Dozic 2mg/ml Oral solution (Rosemont Pharmaceuticals Ltd) | Haloperidol |
| 24494 | Haldol 10mg/ml Liquid (Janssen-Cilag Ltd) | Haloperidol |
| 60942 | Haloperidol 5mg/5ml oral solution | Haloperidol |
| 47149 | Haloperidol 1mg/5ml oral solution | Haloperidol |
| 53649 | Haloperidol 2mg/5ml oral suspension | Haloperidol |
| 47013 | Haloperidol 1mg/5ml oral suspension | Haloperidol |
| 38262 | Haloperidol 5mg/1ml solution for injection ampoules | Haloperidol |
| 34339 | Haloperidol 1.5mg tablets (A A H Pharmaceuticals Ltd) | Haloperidol |
| 45810 | Haloperidol 10mg/5ml oral solution sugar free | Haloperidol |
| 43020 | Haloperidol Oral solution | Haloperidol |
| 2621 | Haloperidol 5mg tablets | Haloperidol |
| 13338 | Serenace 5mg tablets (Teva UK Ltd) | Haloperidol |
| 70584 | Haloperidol 500micrograms/5ml oral solution | Haloperidol |
| 5192 | Haloperidol 1mg/5ml sugar free Oral solution | Haloperidol |
| 32051 | Haloperidol 5mg Tablet (Generics (UK) Ltd) | Haloperidol |
| 13105 | Haloperidol 2mg/ml Oral solution | Haloperidol |
| 55848 | Haloperidol 5mg/1ml solution for injection ampoules (AMCo) | Haloperidol |
| 52050 | Haloperidol 1.5mg/5ml oral suspension | Haloperidol |
| 42000 | Haloperidol 2mg/ml Liquid (Rosemont Pharmaceuticals Ltd) | Haloperidol |
| 12387 | Haloperidol 20mg tablets | Haloperidol |
| 43431 | Haloperidol 500microgram tablets (A A H Pharmaceuticals Ltd) | Haloperidol |
| 12921 | Haldol 2mg/ml oral solution (Janssen-Cilag Ltd) | Haloperidol |
| 43520 | Haloperidol 1.5mg tablets (Teva UK Ltd) | Haloperidol |
| 34272 | Haloperidol 5mg/ml Injection (Antigen Pharmaceuticals) | Haloperidol |
| 47808 | Haloperidol 10mg/5ml oral solution sugar free (A A H Pharmaceuticals Ltd) | Haloperidol |
| 7436 | Serenace 5mg/1ml solution for injection ampoules (IVAX Pharmaceuticals UK Ltd) | Haloperidol |
| 4234 | Haloperidol 5mg/ml Injection | Haloperidol |
| 329 | Haloperidol 1.5mg tablets | Haloperidol |
| 49207 | Haloperidol 2mg/5ml oral solution | Haloperidol |
| 41546 | Haloperidol 1mg/ml Liquid (Hillcross Pharmaceuticals Ltd) | Haloperidol |
| 42807 | Haloperidol 500microgram Tablet (Lagap) | Haloperidol |
| 34039 | Haloperidol 1mg/ml Liquid (Rosemont Pharmaceuticals Ltd) | Haloperidol |
| 34903 | Haloperidol 5mg tablets (IVAX Pharmaceuticals UK Ltd) | Haloperidol |
| 42895 | Haloperidol 5mg tablets (Teva UK Ltd) | Haloperidol |
| 2419 | Haloperidol 500microgram capsules | Haloperidol |
| 71415 | Haloperidol 5mg/5ml oral suspension | Haloperidol |
| 6523 | Haldol 5mg/ml Injection (Janssen-Cilag Ltd) | Haloperidol |
| 8153 | Serenace 2mg/ml liquid (Teva UK Ltd) | Haloperidol |
| 5545 | Serenace 500microgram capsules (Teva UK Ltd) | Haloperidol |
| 38540 | Haldol 5mg/1ml solution for injection ampoules (Janssen-Cilag Ltd) | Haloperidol |
| 8979 | Serenace 1.5mg tablets (Teva UK Ltd) | Haloperidol |
| 3671 | Haloperidol 500microgram tablets | Haloperidol |
| 475 | Haloperidol 10mg tablets | Haloperidol |
| 3233 | Haloperidol 2mg/ml sugar free Liquid | Haloperidol |
| 67364 | Haloperidol 5mg tablets (Crescent Pharma Ltd) | Haloperidol |
| 55871 | Haloperidol 2mg/ml Liquid (Hillcross Pharmaceuticals Ltd) | Haloperidol |
| 70112 | Haloperidol 200micrograms/ml oral solution sugar free | Haloperidol |
| 11213 | Haloperidol 2mg/5ml sugar free Oral solution | Haloperidol |
| 2620 | Haloperidol 1mg/ml Oral solution | Haloperidol |
| 13483 | Serenace 20mg tablets (Teva UK Ltd) | Haloperidol |
| 6134 | Dozic 5mg/5ml oral solution (Rosemont Pharmaceuticals Ltd) | Haloperidol |
| 17379 | Haloperidol 1.5mg/5ml sugar free Oral solution | Haloperidol |
| 22660 | Haldol 5mg tablets (Janssen-Cilag Ltd) | Haloperidol |
| 23678 | Haldol 10mg tablets (Janssen-Cilag Ltd) | Haloperidol |
| 32838 | Haloperidol 1.5mg tablets (IVAX Pharmaceuticals UK Ltd) | Haloperidol |
| 13484 | Serenace 10mg tablets (Teva UK Ltd) | Haloperidol |
| 45880 | Haloperidol 5mg/5ml oral solution sugar free | Haloperidol |
| 69365 | Haloperidol 1mg/ml sugar free Oral solution (Pinewood Healthcare) | Haloperidol |
| 36771 | Haloperidol 250micrograms/5ml oral suspension | Haloperidol |
| 10435 | Haloperidol 10mg/ml Oral solution | Haloperidol |
| 2094 | Haldol decanoate 50mg/1ml solution for injection ampoules (Janssen-Cilag Ltd) | Haloperidol decanoate |
| 15814 | Haloperidol decanoate 100mg/1ml solution for injection ampoules | Haloperidol decanoate |
| 12386 | Haldol decanoate 100mg/1ml solution for injection ampoules (Janssen-Cilag Ltd) | Haloperidol decanoate |
| 10565 | Haloperidol decanoate 50mg/1ml solution for injection ampoules | Haloperidol decanoate |
| 8445 | Stelabid Tablet (GlaxoSmithKline Consumer Healthcare) | Isopropamide Iodide/Trifluoperazine Hydrochloride |
| 61083 | Levomepromazine 25mg/1ml solution for injection ampoules (A A H Pharmaceuticals Ltd) | Levomepromazine hydrochloride |
| 4442 | Nozinan 25mg/1ml solution for injection ampoules (Sanofi) | Levomepromazine hydrochloride |
| 6064 | Levomepromazine 25mg/1ml solution for injection ampoules | Levomepromazine hydrochloride |
| 52846 | Nozinan 25mg/1ml solution for injection ampoules (Lexon (UK) Ltd) | Levomepromazine hydrochloride |
| 59938 | Levomepromazine 25mg/1ml solution for injection ampoules (Wockhardt UK Ltd) | Levomepromazine hydrochloride |
| 60719 | Levomepromazine 5mg/5ml oral solution | Levomepromazine maleate |
| 71201 | Levomepromazine 62.5mg/5ml oral suspension | Levomepromazine maleate |
| 28231 | Levinan 6mg Tablet (Link Pharmaceuticals Ltd) | Levomepromazine maleate |
| 70515 | Levomepromazine 6mg/5ml oral solution | Levomepromazine maleate |
| 60250 | Levomepromazine 3mg/5ml oral solution | Levomepromazine maleate |
| 72003 | Levomepromazine 5mg/5ml oral suspension | Levomepromazine maleate |
| 4232 | Nozinan 25mg tablets (Sanofi) | Levomepromazine maleate |
| 49606 | Levinan 6mg tablets (Kyowa Kirin Ltd) | Levomepromazine maleate |
| 5014 | Levomepromazine 25mg tablets | Levomepromazine maleate |
| 7390 | Levomepromazine 6mg tablets | Levomepromazine maleate |
| 53951 | Levomepromazine 6.25mg/5ml oral solution | Levomepromazine maleate |
| 64673 | Levomepromazine 10mg/5ml oral suspension | Levomepromazine maleate |
| 65843 | Levomepromazine 6mg/5ml oral suspension | Levomepromazine maleate |
| 65865 | Levomepromazine 6.25mg/5ml oral suspension | Levomepromazine maleate |
| 21339 | Veractil 25mg Tablet (Rhone-Poulenc Rorer Ltd) | Levomepromazine maleate |
| 40782 | Levomepromazine 6mg Tablet | Levomepromazine Maleate |
| 72037 | Levomepromazine 100mg tablets | Levomepromazine maleate |
| 68660 | Loxapine 9.1mg/dose inhalation powder | Loxapine |
| 64210 | Latuda 74mg tablets (Sunovion Pharmaceuticals Europe Ltd) | Lurasidone hydrochloride |
| 67717 | Latuda 18.5mg tablets (Sunovion Pharmaceuticals Europe Ltd) | Lurasidone hydrochloride |
| 62387 | Lurasidone 37mg tablets | Lurasidone hydrochloride |
| 62517 | Lurasidone 74mg tablets | Lurasidone hydrochloride |
| 62463 | Latuda 37mg tablets (Sunovion Pharmaceuticals Europe Ltd) | Lurasidone hydrochloride |
| 62924 | Lurasidone 18.5mg tablets | Lurasidone hydrochloride |
| 56072 | Olanzapine 20mg orodispersible tablets | Olanzapine |
| 5653 | Olanzapine 7.5mg tablets | Olanzapine |
| 16407 | Zyprexa 15mg Velotabs (Eli Lilly and Company Ltd) | Olanzapine |
| 36163 | Zyprexa 20mg tablets (Eli Lilly and Company Ltd) | Olanzapine |
| 33883 | Zyprexa 20mg Velotabs (Eli Lilly and Company Ltd) | Olanzapine |
| 18453 | Zyprexa 2.5mg tablets (Eli Lilly and Company Ltd) | Olanzapine |
| 13820 | Zyprexa 10mg tablets (Eli Lilly and Company Ltd) | Olanzapine |
| 47098 | Olanzapine 5mg orodispersible tablets sugar free | Olanzapine |
| 63833 | Olanzapine 2.5mg tablets (Zentiva) | Olanzapine |
| 70219 | Olanzapine 5mg orodispersible tablets (Teva UK Ltd) | Olanzapine |
| 56143 | Olanzapine 15mg orodispersible tablets | Olanzapine |
| 65707 | Olanzapine 20mg orodispersible tablets sugar free (Actavis UK Ltd) | Olanzapine |
| 71367 | Olanzapine 5mg orodispersible tablets sugar free (Actavis UK Ltd) | Olanzapine |
| 14717 | Zyprexa 5mg Velotabs (Eli Lilly and Company Ltd) | Olanzapine |
| 29540 | Olanzapine 20mg tablets | Olanzapine |
| 55622 | Olanzapine 10mg orodispersible tablets | Olanzapine |
| 18024 | Zyprexa 5mg tablets (Eli Lilly and Company Ltd) | Olanzapine |
| 57270 | Olanzapine 2.5mg/5ml oral suspension | Olanzapine |
| 69674 | Olanzapine 2.5mg/5ml oral solution | Olanzapine |
| 6838 | Olanzapine 15mg Orodispersible tablet | Olanzapine |
| 6023 | Olanzapine 10mg Orodispersible tablet | Olanzapine |
| 6412 | Olanzapine 5mg Orodispersible tablet | Olanzapine |
| 23431 | Olanzapine 10mg powder for solution for injection vials | Olanzapine |
| 47103 | Olanzapine 15mg orodispersible tablets sugar free | Olanzapine |
| 53848 | Zalasta 5mg orodispersible tablets (Consilient Health Ltd) | Olanzapine |
| 63615 | Olanzapine 2.5mg tablets (Dr Reddy's Laboratories (UK) Ltd) | Olanzapine |
| 61103 | Olanzapine 15mg oral lyophilisates sugar free | Olanzapine |
| 53556 | Olanzapine 10mg oral lyophilisates sugar free | Olanzapine |
| 13888 | Zyprexa 10mg Velotabs (Eli Lilly and Company Ltd) | Olanzapine |
| 1249 | Olanzapine 10mg tablets | Olanzapine |
| 56265 | Olanzapine 20mg oral lyophilisates sugar free | Olanzapine |
| 68346 | Zalasta 20mg tablets (Consilient Health Ltd) | Olanzapine |
| 70640 | Olanzapine 12.5mg/5ml oral suspension | Olanzapine |
| 47093 | Olanzapine 20mg orodispersible tablets sugar free | Olanzapine |
| 71478 | Olanzapine 2.5mg tablets (Alliance Healthcare (Distribution) Ltd) | Olanzapine |
| 45953 | Zyprexa 10mg powder for solution for injection vials (Eli Lilly and Company Ltd) | Olanzapine |
| 69511 | Olanzapine 10mg tablets (Teva UK Ltd) | Olanzapine |
| 57616 | Olanzapine 20mg tablets (Teva UK Ltd) | Olanzapine |
| 59143 | Olanzapine 2.5mg tablets (Teva UK Ltd) | Olanzapine |
| 70367 | Olanzapine 10mg tablets (Alliance Healthcare (Distribution) Ltd) | Olanzapine |
| 58854 | Olanzapine 10mg tablets (Actavis UK Ltd) | Olanzapine |
| 55667 | Olanzapine 15mg tablets (Actavis UK Ltd) | Olanzapine |
| 70415 | Olanzapine 15mg tablets (Teva UK Ltd) | Olanzapine |
| 57160 | Olanzapine 5mg oral lyophilisates sugar free | Olanzapine |
| 6850 | Olanzapine 15mg tablets | Olanzapine |
| 65944 | Olanzapine 2.5mg tablets (Mylan) | Olanzapine |
| 70542 | Olanzapine 5mg orodispersible tablets sugar free (Alliance Healthcare (Distribution) Ltd) | Olanzapine |
| 58147 | Olanzapine 10mg tablets (Zentiva) | Olanzapine |
| 68347 | Zalasta 5mg tablets (Consilient Health Ltd) | Olanzapine |
| 2656 | Olanzapine 2.5mg tablets | Olanzapine |
| 52001 | Olanzapine 2.5mg tablets (Aspire Pharma Ltd) | Olanzapine |
| 16103 | Olanzapine 20mg Orodispersible tablet | Olanzapine |
| 50214 | Olanzapine 5mg orodispersible tablets | Olanzapine |
| 47063 | Olanzapine 10mg orodispersible tablets sugar free | Olanzapine |
| 21964 | Zyprexa 7.5mg tablets (Eli Lilly and Company Ltd) | Olanzapine |
| 63155 | Olanzapine 7.5mg tablets (Actavis UK Ltd) | Olanzapine |
| 19976 | Zyprexa 15mg tablets (Eli Lilly and Company Ltd) | Olanzapine |
| 3281 | Olanzapine 5mg tablets | Olanzapine |
| 71670 | Olanzapine 5mg tablets (Alliance Healthcare (Distribution) Ltd) | Olanzapine |
| 69121 | Olanzapine embonate 405mg powder and solvent for suspension for injection vials | Olanzapine embonate monohydrate |
| 46422 | Olanzapine embonate 300mg powder and solvent for suspension for injection vials | Olanzapine embonate monohydrate |
| 55268 | Zypadhera 300mg powder and solvent for suspension for injection vials (Eli Lilly and Company Ltd) | Olanzapine embonate monohydrate |
| 43914 | Olanzapine embonate 210mg powder and solvent for suspension for injection vials | Olanzapine embonate monohydrate |
| 71734 | Zypadhera 405mg powder and solvent for suspension for injection vials (Eli Lilly and Company Ltd) | Olanzapine embonate monohydrate |
| 8921 | Integrin 10mg Capsule (Sanofi-Synthelabo Ltd) | Oxypertine |
| 27211 | Integrin 40mg Tablet (Sanofi-Synthelabo Ltd) | Oxypertine |
| 37501 | Paliperidone 9mg modified-release tablets | Paliperidone |
| 37717 | Paliperidone 3mg modified-release tablets | Paliperidone |
| 36954 | Invega 6mg modified-release tablets (Janssen-Cilag Ltd) | Paliperidone |
| 36116 | Paliperidone 6mg modified-release tablets | Paliperidone |
| 46436 | Xeplion 75mg/0.75ml suspension for injection pre-filled syringes (Janssen-Cilag Ltd) | Paliperidone palmitate |
| 46447 | Paliperidone 100mg/1ml suspension for injection pre-filled syringes | Paliperidone palmitate |
| 70867 | Paliperidone 525mg/2.625ml prolonged-release suspension for injection pre-filled syringes | Paliperidone palmitate |
| 46351 | Paliperidone 150mg/1.5ml suspension for injection pre-filled syringes | Paliperidone palmitate |
| 46435 | Xeplion 150mg/1.5ml suspension for injection pre-filled syringes (Janssen-Cilag Ltd) | Paliperidone palmitate |
| 46224 | Paliperidone 50mg/0.5ml suspension for injection pre-filled syringes | Paliperidone palmitate |
| 46556 | Paliperidone 75mg/0.75ml suspension for injection pre-filled syringes | Paliperidone palmitate |
| 70433 | Trevicta 350mg/1.75ml prolonged-release suspension for injection pre-filled syringes (Janssen-Cilag Ltd) | Paliperidone palmitate |
| 47162 | Xeplion 50mg/0.5ml suspension for injection pre-filled syringes (Janssen-Cilag Ltd) | Paliperidone palmitate |
| 46434 | Xeplion 100mg/1ml suspension for injection pre-filled syringes (Janssen-Cilag Ltd) | Paliperidone palmitate |
| 70122 | Trevicta 525mg/2.625ml prolonged-release suspension for injection pre-filled syringes (Janssen-Cilag Ltd) | Paliperidone palmitate |
| 68297 | Trevicta 263mg/1.315ml prolonged-release suspension for injection pre-filled syringes (Janssen-Cilag Ltd) | Paliperidone palmitate |
| 21064 | Neulactil 25mg Tablet (JHC Healthcare Ltd) | Pericyazine |
| 7834 | Pericyazine 2.5mg tablets | Pericyazine |
| 8032 | Pericyazine 10mg tablets | Pericyazine |
| 8031 | Neulactil 10mg Tablet (JHC Healthcare Ltd) | Pericyazine |
| 39830 | Neulactil 2.5mg tablets (Sanofi) | Pericyazine |
| 40881 | Neulactil 10mg tablets (Sanofi) | Pericyazine |
| 15472 | Pericyazine 25mg tablet | Pericyazine |
| 12195 | Pericyazine 10mg/5ml oral solution | Pericyazine |
| 7833 | Neulactil 2.5mg Tablet (JHC Healthcare Ltd) | Pericyazine |
| 13902 | Neulactil Forte syrup (Sanofi) | Pericyazine |
| 67777 | Pericyazine 2.5mg tablets (Zentiva) | Pericyazine |
| 25909 | Perphenazine 4mg/5ml Oral solution sugar free | Perphenazine |
| 228 | Fentazin 5mg/ml Injection (Goldshield Pharmaceuticals Ltd) | Perphenazine |
| 840 | Fentazin 2mg tablets (AMCo) | Perphenazine |
| 609 | Perphenazine 2mg tablets | Perphenazine |
| 14987 | Perphenazine 2mg/5ml oral solution sugar free | Perphenazine |
| 2157 | Perphenazine 4mg tablets | Perphenazine |
| 7919 | Fentazin 4mg tablets (AMCo) | Perphenazine |
| 17087 | Perphenazine 5mg/ml injection | Perphenazine |
| 15047 | Orap 4mg tablets (Eumedica Pharmaceuticals) | Pimozide |
| 5821 | Pimozide 4mg tablets | Pimozide |
| 8637 | Pimozide 10mg tablet | Pimozide |
| 27148 | Orap 10mg Tablet (Janssen-Cilag Ltd) | Pimozide |
| 64567 | Pimozide 1mg tablets | Pimozide |
| 35488 | Piportil Depot 100mg/2ml solution for injection ampoules (Sanofi) | Pipotiazine palmitate |
| 35235 | Piportil Depot 50mg/1ml solution for injection ampoules (Sanofi) | Pipotiazine palmitate |
| 36394 | Pipotiazine 100mg/2ml solution for injection ampoules | Pipotiazine palmitate |
| 10944 | Pipotiazine palmitate 50mg/ml depot injection | Pipotiazine Palmitate |
| 12340 | Piportil 50mg/ml Depot injection (JHC Healthcare Ltd) | Pipotiazine Palmitate |
| 35684 | Pipotiazine 50mg/1ml solution for injection ampoules | Pipotiazine palmitate |
| 54429 | Stemetil 5mg tablets (Waymade Healthcare Plc) | Prochlorperazine maleate |
| 1990 | Prochlorperazine 25mg suppositories | Prochlorperazine maleate |
| 54458 | Prochlorperazine 5mg Tablet (Teva UK Ltd) | Prochlorperazine maleate |
| 39887 | Stemetil 5mg tablets (Sanofi) | Prochlorperazine maleate |
| 1434 | Prochlorperazine 5mg suppositories | Prochlorperazine maleate |
| 51551 | Stemetil 5mg tablets (Mawdsley-Brooks & Company Ltd) | Prochlorperazine maleate |
| 32772 | Prochlorperazine 5mg tablets (Mylan) | Prochlorperazine maleate |
| 512 | Stemetil 5mg Tablet (Castlemead Healthcare Ltd) | Prochlorperazine maleate |
| 500 | Buccastem 3mg Tablet (Reckitt Benckiser Healthcare (UK) Ltd) | Prochlorperazine maleate |
| 13607 | Proziere 5mg tablets (Ashbourne Pharmaceuticals Ltd) | Prochlorperazine maleate |
| 51579 | Stemetil 5mg tablets (Sigma Pharmaceuticals Plc) | Prochlorperazine maleate |
| 32551 | Prochlorperazine 5mg tablets (IVAX Pharmaceuticals UK Ltd) | Prochlorperazine maleate |
| 227 | Stemetil 5mg suppositories (Sanofi) | Prochlorperazine maleate |
| 55038 | Prochlorperazine 5mg tablets (Sigma Pharmaceuticals Plc) | Prochlorperazine maleate |
| 17849 | Vertigon spansule 15 15mg Spansule (GlaxoSmithKline Consumer Healthcare) | Prochlorperazine Maleate |
| 1234 | Stemetil 25mg suppositories (Sanofi) | Prochlorperazine maleate |
| 15438 | Prochlorperazine maleate 15mg modified release capsul | Prochlorperazine Maleate |
| 62115 | Prochlorperazine 5mg tablets (Genesis Pharmaceuticals Ltd) | Prochlorperazine maleate |
| 34344 | Prochlorperazine 5mg tablets (A A H Pharmaceuticals Ltd) | Prochlorperazine maleate |
| 61592 | Prochlorperazine 5mg tablets (Almus Pharmaceuticals Ltd) | Prochlorperazine maleate |
| 32876 | Prochlorperazine 5mg tablets (Teva UK Ltd) | Prochlorperazine maleate |
| 50462 | Stemetil 5mg tablets (DE Pharmaceuticals) | Prochlorperazine maleate |
| 32064 | Prochlorperazine 5mg tablets (Actavis UK Ltd) | Prochlorperazine maleate |
| 68942 | Prochlorperazine 5mg tablets (DE Pharmaceuticals) | Prochlorperazine maleate |
| 49170 | Stemetil 5mg tablets (Lexon (UK) Ltd) | Prochlorperazine maleate |
| 43420 | Prochlorperazine 5mg tablets (Dr Reddy's Laboratories (UK) Ltd) | Prochlorperazine maleate |
| 4401 | Prochlorperazine maleate 10mg modified release capsule | Prochlorperazine Maleate |
| 85 | Prochlorperazine 5mg tablets | Prochlorperazine maleate |
| 8689 | Vertigon spansule 10 10mg Spansule (GlaxoSmithKline Consumer Healthcare) | Prochlorperazine Maleate |
| 40001 | Stemetil 5mg/5ml syrup (Sanofi) | Prochlorperazine mesilate |
| 14356 | Stemetil 12.5mg/1ml solution for injection ampoules (Sanofi) | Prochlorperazine mesilate |
| 32122 | Prochlorperazine 12.5mg/1ml solution for injection ampoules (AMCo) | Prochlorperazine mesilate |
| 6036 | Prochlorperazine 5mg/5ml oral solution | Prochlorperazine mesilate |
| 5510 | Prochlorperazine mesilate 12.5mg/ml injection | Prochlorperazine Mesilate |
| 7593 | Stemetil 5mg/5ml Oral solution (Castlemead Healthcare Ltd) | Prochlorperazine mesilate |
| 14364 | Prochlorperazine 12.5mg/1ml solution for injection ampoules | Prochlorperazine mesilate |
| 3246 | Stemetil 12.5mg/ml Injection (Castlemead Healthcare Ltd) | Prochlorperazine Mesilate |
| 6443 | Promazine 25mg/5ml oral solution | Promazine hydrochloride |
| 33493 | Sparine 100mg Tablet (Wyeth Pharmaceuticals) | Promazine Hydrochloride |
| 17634 | Promazine 50mg/5ml oral solution | Promazine hydrochloride |
| 46945 | Promazine 25mg Tablet (Biorex Laboratories Ltd) | Promazine hydrochloride |
| 13311 | Sparine 50mg/ml Injection (Wyeth Pharmaceuticals) | Promazine Hydrochloride |
| 43654 | Promazine 50mg/ml injection | Promazine Hydrochloride |
| 10780 | Promazine 50mg/5ml oral solution | Promazine Hydrochloride |
| 38089 | Promazine 50mg/5ml syrup (Rosemont Pharmaceuticals Ltd) | Promazine hydrochloride |
| 41995 | Promazine 50mg/ml Injection (Genus Pharmaceuticals Ltd) | Promazine Hydrochloride |
| 67799 | Promazine 50mg/5ml oral suspension | Promazine hydrochloride |
| 15395 | Promazine 12.5mg/5ml oral solution | Promazine Hydrochloride |
| 55890 | Promazine 50mg/5ml Liquid (Rosemont Pharmaceuticals Ltd) | Promazine Hydrochloride |
| 3226 | Sparine 50mg Tablet (Wyeth Pharmaceuticals) | Promazine hydrochloride |
| 40390 | Promazine 25mg/5ml syrup (Rosemont Pharmaceuticals Ltd) | Promazine hydrochloride |
| 71382 | Promazine 50mg/5ml oral solution (A A H Pharmaceuticals Ltd) | Promazine hydrochloride |
| 15161 | Promazine 50mg/ml injection | Promazine Hydrochloride |
| 2972 | Promazine 25mg tablets | Promazine hydrochloride |
| 12193 | Sparine 25mg Tablet (Wyeth Pharmaceuticals) | Promazine hydrochloride |
| 3228 | Promazine 50mg tablets | Promazine hydrochloride |
| 41732 | Promazine 50mg tablets (Teva UK Ltd) | Promazine hydrochloride |
| 65449 | Promazine 25mg tablets (Teva UK Ltd) | Promazine hydrochloride |
| 3197 | Promazine 100mg tablet | Promazine Hydrochloride |
| 60450 | Promazine 25mg tablets (A A H Pharmaceuticals Ltd) | Promazine hydrochloride |
| 63601 | Promazine 25mg/5ml oral solution (A A H Pharmaceuticals Ltd) | Promazine hydrochloride |
| 14610 | Promazine 50mg/5ml oral solution sugar free | Promazine Hydrochloride |
| 3227 | Sparine 50mg/5ml Liquid (Wyeth Pharmaceuticals) | Promazine Hydrochloride |
| 21709 | Seroquel 300mg tablets (AstraZeneca UK Ltd) | Quetiapine fumarate |
| 9794 | Quetiapine 200mg tablets | Quetiapine fumarate |
| 65938 | Quetiapine 25mg tablets (Ranbaxy (UK) Ltd) | Quetiapine fumarate |
| 66427 | Quetiapine 25mg tablets (Accord Healthcare Ltd) | Quetiapine fumarate |
| 7039 | Quetiapine 300mg tablets | Quetiapine fumarate |
| 53552 | Quetiapine 25mg tablets (Zentiva) | Quetiapine fumarate |
| 62531 | Atrolak XL 400mg tablets (Accord Healthcare Ltd) | Quetiapine fumarate |
| 60842 | Quetiapine 100mg tablets (Alliance Healthcare (Distribution) Ltd) | Quetiapine fumarate |
| 61748 | Ebesque XL 200mg tablets (Ethypharm UK Ltd) | Quetiapine fumarate |
| 57412 | Sondate XL 400mg tablets (Teva UK Ltd) | Quetiapine fumarate |
| 5283 | Quetiapine 25mg tablets | Quetiapine fumarate |
| 39237 | Seroquel XL 400mg tablets (AstraZeneca UK Ltd) | Quetiapine fumarate |
| 69637 | Mintreleq XL 200mg tablets (Aristo Pharma Ltd) | Quetiapine fumarate |
| 59215 | Quetiapine 150mg tablets (Ranbaxy (UK) Ltd) | Quetiapine fumarate |
| 38912 | Quetiapine 200mg modified-release tablets | Quetiapine fumarate |
| 40932 | Quetiapine 100mg/5ml oral suspension | Quetiapine fumarate |
| 70992 | Quetiapine 100mg tablets (Ranbaxy (UK) Ltd) | Quetiapine fumarate |
| 51178 | Quetiapine 50mg/5ml oral suspension | Quetiapine fumarate |
| 62943 | Biquelle XL 400mg tablets (Aspire Pharma Ltd) | Quetiapine fumarate |
| 18013 | Seroquel 100mg tablets (AstraZeneca UK Ltd) | Quetiapine fumarate |
| 57034 | Sondate XL 200mg tablets (Teva UK Ltd) | Quetiapine fumarate |
| 38840 | Quetiapine 400mg modified-release tablets | Quetiapine fumarate |
| 14859 | Seroquel 25mg tablets (AstraZeneca UK Ltd) | Quetiapine fumarate |
| 65174 | Quetiapine 300mg tablets (A A H Pharmaceuticals Ltd) | Quetiapine fumarate |
| 70192 | Quetiapine 20mg/ml oral suspension sugar free | Quetiapine fumarate |
| 61575 | Ebesque XL 50mg tablets (Ethypharm UK Ltd) | Quetiapine fumarate |
| 70684 | Quetiapine 20mg/ml oral suspension sugar free (Rosemont Pharmaceuticals Ltd) | Quetiapine fumarate |
| 44326 | Seroquel XL 150mg tablets (AstraZeneca UK Ltd) | Quetiapine fumarate |
| 63359 | Zaluron XL 300mg tablets (Fontus Health Ltd) | Quetiapine fumarate |
| 70312 | Quetiapine 200mg tablets (A A H Pharmaceuticals Ltd) | Quetiapine fumarate |
| 38885 | Quetiapine 50mg modified-release tablets | Quetiapine fumarate |
| 63364 | Zaluron XL 200mg tablets (Fontus Health Ltd) | Quetiapine fumarate |
| 66255 | Atrolak XL 200mg tablets (Accord Healthcare Ltd) | Quetiapine fumarate |
| 70313 | Quetiapine 100mg tablets (DE Pharmaceuticals) | Quetiapine fumarate |
| 56215 | Quetiapine 50mg/5ml oral solution | Quetiapine fumarate |
| 52940 | Sondate XL 50mg tablets (Teva UK Ltd) | Quetiapine fumarate |
| 68375 | Quetiapine 25mg tablets (Teva UK Ltd) | Quetiapine fumarate |
| 14813 | Seroquel 150mg tablets (AstraZeneca UK Ltd) | Quetiapine fumarate |
| 5040 | Quetiapine 150mg tablets | Quetiapine fumarate |
| 63797 | Zaluron XL 150mg tablets (Fontus Health Ltd) | Quetiapine fumarate |
| 63818 | Mintreleq XL 150mg tablets (Aristo Pharma Ltd) | Quetiapine fumarate |
| 55870 | Quetiapine oral liquid | Quetiapine Fumarate |
| 63389 | Zaluron XL 50mg tablets (Fontus Health Ltd) | Quetiapine fumarate |
| 58067 | Quetiapine 125mg/5ml oral suspension | Quetiapine fumarate |
| 56647 | Quetiapine 300mg tablets (Arrow Generics Ltd) | Quetiapine fumarate |
| 45839 | Quetiapine 25mg/5ml oral suspension | Quetiapine fumarate |
| 57612 | Seroquel XL 400mg tablets (Lexon (UK) Ltd) | Quetiapine fumarate |
| 63363 | Zaluron XL 400mg tablets (Fontus Health Ltd) | Quetiapine fumarate |
| 63351 | Biquelle XL 200mg tablets (Aspire Pharma Ltd) | Quetiapine fumarate |
| 49696 | Quetiapine 25mg/5ml oral solution | Quetiapine fumarate |
| 46764 | Quetiapine 12.5mg/5ml oral solution | Quetiapine fumarate |
| 58935 | Tenprolide XL 400mg tablets (Actavis UK Ltd) | Quetiapine fumarate |
| 40779 | Quetiapine 100mg/5ml oral solution | Quetiapine fumarate |
| 5039 | Quetiapine 100mg tablets | Quetiapine fumarate |
| 64639 | Mintreleq XL 300mg tablets (Aristo Pharma Ltd) | Quetiapine fumarate |
| 58821 | Quetiapine 25mg tablets (Dr Reddy's Laboratories (UK) Ltd) | Quetiapine fumarate |
| 38913 | Seroquel XL 50mg tablets (AstraZeneca UK Ltd) | Quetiapine fumarate |
| 69544 | Atrolak XL 50mg tablets (Accord Healthcare Ltd) | Quetiapine fumarate |
| 57613 | Seroquel XL 50mg tablets (Sigma Pharmaceuticals Plc) | Quetiapine fumarate |
| 10107 | Quetiapine Starter Pack | Quetiapine Fumarate |
| 58936 | Tenprolide XL 50mg tablets (Actavis UK Ltd) | Quetiapine fumarate |
| 58425 | Seroquel XL 50mg tablets (DE Pharmaceuticals) | Quetiapine fumarate |
| 54483 | Sondate XL 300mg tablets (Teva UK Ltd) | Quetiapine fumarate |
| 63049 | Biquelle XL 50mg tablets (Aspire Pharma Ltd) | Quetiapine fumarate |
| 64484 | Quetiapine 200mg/5ml oral suspension | Quetiapine fumarate |
| 63925 | Mintreleq XL 50mg tablets (Aristo Pharma Ltd) | Quetiapine fumarate |
| 69836 | Quetiapine 400mg/5ml oral suspension | Quetiapine fumarate |
| 63133 | Biquelle XL 150mg tablets (Aspire Pharma Ltd) | Quetiapine fumarate |
| 38914 | Seroquel XL 200mg tablets (AstraZeneca UK Ltd) | Quetiapine fumarate |
| 69787 | Tenprolide XL 300mg tablets (Actavis UK Ltd) | Quetiapine fumarate |
| 44024 | Quetiapine 150mg modified-release tablets | Quetiapine fumarate |
| 61747 | Ebesque XL 300mg tablets (Ethypharm UK Ltd) | Quetiapine fumarate |
| 46871 | Quetiapine 12.5mg/5ml oral suspension | Quetiapine fumarate |
| 63087 | Biquelle XL 300mg tablets (Aspire Pharma Ltd) | Quetiapine fumarate |
| 6864 | Seroquel 200mg tablets (AstraZeneca UK Ltd) | Quetiapine fumarate |
| 64778 | Sondate XL 150mg tablets (Teva UK Ltd) | Quetiapine fumarate |
| 61746 | Ebesque XL 400mg tablets (Ethypharm UK Ltd) | Quetiapine fumarate |
| 67334 | Seroquel XL 50mg tablets (Waymade Healthcare Plc) | Quetiapine fumarate |
| 38937 | Seroquel XL 300mg tablets (AstraZeneca UK Ltd) | Quetiapine fumarate |
| 69635 | Mintreleq XL 400mg tablets (Aristo Pharma Ltd) | Quetiapine fumarate |
| 70907 | Seroquel 25mg tablets (Mawdsley-Brooks & Company Ltd) | Quetiapine fumarate |
| 38906 | Quetiapine 300mg modified-release tablets | Quetiapine fumarate |
| 16223 | Roxiam 300mg Capsule (AstraZeneca UK Ltd) | Remoxipride Hydrochloride Monohydrate |
| 48077 | Roxiam ir 75mg Capsule (AstraZeneca UK Ltd) | Remoxipride Hydrochloride Monohydrate |
| 23034 | Remoxipride 75mg capsule | Remoxipride Hydrochloride Monohydrate |
| 12445 | Remoxipride 300mg capsule | Remoxipride Hydrochloride Monohydrate |
| 19016 | Roxiam 150mg Capsule (AstraZeneca UK Ltd) | Remoxipride Hydrochloride Monohydrate |
| 8881 | Remoxipride 150mg capsule | Remoxipride Hydrochloride Monohydrate |
| 16434 | Risperidone 25mg powder and solvent for suspension for injection vials | Risperidone |
| 65321 | Risperidone 500microgram tablets (Teva UK Ltd) | Risperidone |
| 4820 | Risperdal 1mg tablets (Janssen-Cilag Ltd) | Risperidone |
| 63494 | Risperidone 500microgram orodispersible tablets sugar free (A A H Pharmaceuticals Ltd) | Risperidone |
| 9340 | Risperdal 3mg tablets (Janssen-Cilag Ltd) | Risperidone |
| 667 | Risperidone 500microgram tablets | Risperidone |
| 65472 | Risperidone 3mg orodispersible tablets sugar free (A A H Pharmaceuticals Ltd) | Risperidone |
| 11799 | Risperdal 6mg tablets (Janssen-Cilag Ltd) | Risperidone |
| 35141 | Risperidone 3mg orodispersible tablets sugar free | Risperidone |
| 63511 | Risperidone 1mg tablets (Actavis UK Ltd) | Risperidone |
| 9475 | Risperdal 4mg tablets (Janssen-Cilag Ltd) | Risperidone |
| 6373 | Risperidone 1mg orodispersible tablets sugar free | Risperidone |
| 1320 | Risperidone 1mg tablets | Risperidone |
| 63255 | Risperidone 1mg tablets (Accord Healthcare Ltd) | Risperidone |
| 35548 | Risperdal Quicklet 3mg orodispersible tablets (Janssen-Cilag Ltd) | Risperidone |
| 302 | Risperidone 1mg/ml oral solution sugar free | Risperidone |
| 1321 | Risperidone 2mg tablets | Risperidone |
| 64493 | Risperidone 1mg tablets (A A H Pharmaceuticals Ltd) | Risperidone |
| 11821 | Risperdal Quicklet 500microgram orodispersible tablets (Janssen-Cilag Ltd) | Risperidone |
| 16425 | Risperidone 37.5mg powder and solvent for suspension for injection vials | Risperidone |
| 51444 | Risperdal Consta 50mg powder and solvent for suspension for injection vials (Waymade Healthcare Plc) | Risperidone |
| 58822 | Risperidone 4mg tablets (Almus Pharmaceuticals Ltd) | Risperidone |
| 59548 | Risperidone 2mg tablets (Alliance Healthcare (Distribution) Ltd) | Risperidone |
| 16908 | Risperdal Consta 25mg powder and solvent for suspension for injection vials (Janssen-Cilag Ltd) | Risperidone |
| 14789 | Risperdal Consta 37.5mg powder and solvent for suspension for injection vials (Janssen-Cilag Ltd) | Risperidone |
| 2787 | Risperidone 4mg tablets | Risperidone |
| 51240 | Risperidone 125micrograms/5ml oral solution | Risperidone |
| 47832 | Risperidone 500microgram tablets (A A H Pharmaceuticals Ltd) | Risperidone |
| 2786 | Risperidone 6mg tablets | Risperidone |
| 46677 | Risperidone 500microgram tablets (Actavis UK Ltd) | Risperidone |
| 16986 | Risperdal Quicklet 1mg orodispersible tablets (Janssen-Cilag Ltd) | Risperidone |
| 65137 | Risperidone 500microgram tablets (Dexcel-Pharma Ltd) | Risperidone |
| 5262 | Risperdal 1mg/ml oral solution (Janssen-Cilag Ltd) | Risperidone |
| 35589 | Risperidone 4mg orodispersible tablets sugar free | Risperidone |
| 5219 | Risperidone 3mg tablets | Risperidone |
| 59829 | Risperidone 3mg tablets (A A H Pharmaceuticals Ltd) | Risperidone |
| 69328 | Risperidone 500microgram tablets (Wockhardt UK Ltd) | Risperidone |
| 14767 | Risperdal Consta 50mg powder and solvent for suspension for injection vials (Janssen-Cilag Ltd) | Risperidone |
| 65493 | Risperidone 1mg tablets (Wockhardt UK Ltd) | Risperidone |
| 16489 | Risperidone 50mg powder and solvent for suspension for injection vials | Risperidone |
| 35953 | Risperdal Quicklet 4mg orodispersible tablets (Janssen-Cilag Ltd) | Risperidone |
| 9659 | Risperdal 2mg tablets (Janssen-Cilag Ltd) | Risperidone |
| 62916 | Risperidone 500microgram orodispersible tablets sugar free (Teva UK Ltd) | Risperidone |
| 11828 | Risperidone 2mg orodispersible tablets sugar free | Risperidone |
| 54346 | Risperidone 1mg tablets (Teva UK Ltd) | Risperidone |
| 7382 | Risperidone 500microgram orodispersible tablets sugar free | Risperidone |
| 64957 | Risperidone 1mg/ml oral solution sugar free (Rosemont Pharmaceuticals Ltd) | Risperidone |
| 55661 | Risperidone 1mg tablets (Kent Pharmaceuticals Ltd) | Risperidone |
| 57217 | Risperidone 1mg tablets (Mylan) | Risperidone |
| 16006 | Risperdal Quicklet 2mg orodispersible tablets (Janssen-Cilag Ltd) | Risperidone |
| 631 | Risperdal 500microgram tablets (Janssen-Cilag Ltd) | Risperidone |
| 56387 | Risperidone 1mg/ml oral solution sugar free (Alliance Healthcare (Distribution) Ltd) | Risperidone |
| 25966 | Serdolect 4mg tablets (Lundbeck Ltd) | Sertindole |
| 31063 | Serdolect 12mg tablets (Lundbeck Ltd) | Sertindole |
| 71389 | Serdolect 20mg tablets (Lundbeck Ltd) | Sertindole |
| 12666 | Sertindole 4mg tablets | Sertindole |
| 16998 | Sertindole 12mg tablets | Sertindole |
| 23162 | Serdolect 16mg tablets (Lundbeck Ltd) | Sertindole |
| 17050 | Sertindole 20mg tablets | Sertindole |
| 19900 | Sertindole 16mg tablets | Sertindole |
| 24069 | Dolmatil 400mg tablets (Sanofi) | Sulpiride |
| 2135 | Sulpiride 200mg tablets | Sulpiride |
| 8903 | Sulpiride 200mg/5ml oral solution sugar free | Sulpiride |
| 70833 | Sulpiride 400mg tablets (Wockhardt UK Ltd) | Sulpiride |
| 9247 | Sulpiride 400mg tablets | Sulpiride |
| 43522 | Sulpiride 200mg tablets (Teva UK Ltd) | Sulpiride |
| 18181 | Sulpor 200mg/5ml oral solution (Rosemont Pharmaceuticals Ltd) | Sulpiride |
| 24053 | Sulparex 200mg Tablet (E R Squibb and Sons Ltd) | Sulpiride |
| 43423 | Sulpiride 200mg tablets (A A H Pharmaceuticals Ltd) | Sulpiride |
| 18352 | Sulpitil 200mg tablets (Pfizer Ltd) | Sulpiride |
| 34810 | Sulpiride 200mg tablets (Wockhardt UK Ltd) | Sulpiride |
| 10666 | Dolmatil 200mg tablets (Sanofi) | Sulpiride |
| 41675 | Sulpiride 200mg tablets (IVAX Pharmaceuticals UK Ltd) | Sulpiride |
| 71421 | Thioridazine 50mg/5ml oral suspension | Thioridazine |
| 1192 | Thioridazine 10mg tablets | Thioridazine hydrochloride |
| 3605 | Thioridazine 25mg/5ml oral solution | Thioridazine hydrochloride |
| 34905 | Thioridazine 25mg Tablet (IVAX Pharmaceuticals UK Ltd) | Thioridazine hydrochloride |
| 35787 | Thioridazine 50mg Tablet (IVAX Pharmaceuticals UK Ltd) | Thioridazine hydrochloride |
| 17399 | Thioridazine 50mg/5ml Oral solution | Thioridazine Hydrochloride |
| 10405 | Thioridazine 25mg/5ml sugar free Oral solution | Thioridazine Hydrochloride |
| 1218 | Thioridazine 25mg tablets | Thioridazine hydrochloride |
| 47361 | Thioridazine 10mg/5ml Oral solution (Rosemont Pharmaceuticals Ltd) | Thioridazine Hydrochloride |
| 15598 | Thioridazine 100mg/5ml sugar free Oral solution | Thioridazine Hydrochloride |
| 3021 | Thioridazine 100mg tablets | Thioridazine hydrochloride |
| 1314 | Thioridazine 50mg tablets | Thioridazine hydrochloride |
| 42816 | Thioridazine 50mg/5ml Oral solution (Rosemont Pharmaceuticals Ltd) | Thioridazine Hydrochloride |
| 2801 | Thioridazine 10mg/5ml Oral solution | Thioridazine Hydrochloride |
| 45860 | Thioridazine 100mg Tablet (IVAX Pharmaceuticals UK Ltd) | Thioridazine hydrochloride |
| 23541 | Ipecacuanha mixture paediatric | Tolu syrup/Ipecacuanha tincture/Sodium bicarbonate/Chloroform water double strength |
| 41663 | Trifluoperazine 5mg tablets (A A H Pharmaceuticals Ltd) | Trifluoperazine hydrochloride |
| 13145 | Trifluoperazine 1mg/5ml oral solution sugar free | Trifluoperazine hydrochloride |
| 1318 | Stelazine 1mg tablets (Mercury Pharma Group Ltd) | Trifluoperazine hydrochloride |
| 18289 | Stelazine 10mg/ml Concentrate (Goldshield Pharmaceuticals Ltd) | Trifluoperazine Hydrochloride |
| 11531 | Trifluoperazine 5mg/5ml oral solution sugar free | Trifluoperazine hydrochloride |
| 29948 | Stelazine Forte 1mg/ml oral solution (Mercury Pharma Group Ltd) | Trifluoperazine hydrochloride |
| 7479 | Stelazine 1mg/ml Injection (Goldshield Pharmaceuticals Ltd) | Trifluoperazine Hydrochloride |
| 1857 | Trifluoperazine 1mg tablets | Trifluoperazine hydrochloride |
| 55382 | Trifluoperazine 1mg/5ml oral solution sugar free (AMCo) | Trifluoperazine hydrochloride |
| 8537 | Trifluoperazine 1mg/ml Injection | Trifluoperazine Hydrochloride |
| 8985 | Stelazine 1mg/5ml syrup (Mercury Pharma Group Ltd) | Trifluoperazine hydrochloride |
| 1316 | Stelazine 5mg tablets (Mercury Pharma Group Ltd) | Trifluoperazine hydrochloride |
| 18668 | Trifluoperazine 10mg/ml concentrate | Trifluoperazine Hydrochloride |
| 1245 | Trifluoperazine 5mg tablets | Trifluoperazine hydrochloride |
| 57605 | Stelazine 1mg tablets (Lexon (UK) Ltd) | Trifluoperazine hydrochloride |
| 71387 | Stelazine 5mg tablets (Lexon (UK) Ltd) | Trifluoperazine hydrochloride |
| 67805 | Stelazine 1mg tablets (Imported (South Africa)) | Trifluoperazine hydrochloride |
| 64216 | Stelazine 5mg tablets (Imported (South Africa)) | Trifluoperazine hydrochloride |
| 40162 | Trifluoperazine 1mg tablets (A A H Pharmaceuticals Ltd) | Trifluoperazine hydrochloride |
| 3955 | Tranylcypromine with trifluoperazine Tablet | Trifluoperazine Hydrochloride/Tranylcypromine Sulphate |
| 24890 | Trifluoperazine with tranylcypromine 1mg + 10mg Tablet | Trifluoperazine Hydrochloride/Tranylcypromine Sulphate |
| 3356 | Parstelin Tablet (GlaxoSmithKline Consumer Healthcare) | Trifluoperazine Hydrochloride/Tranylcypromine Sulphate |
| 21027 | Triperidol 1mg Tablet (Lagap) | Trifluperidol |
| 22814 | Trifluperidol 1mg Tablet | Trifluperidol |
| 23659 | Trifluperidol 0.5mg Tablet | Trifluperidol |
| 21047 | Triperidol 0.5mg Tablet (Lagap) | Trifluperidol |
| 25336 | Zotepine 50mg tablets | Zotepine |
| 30088 | Zoleptil 25 tablets (Movianto UK Ltd) | Zotepine |
| 28759 | Zotepine 100mg tablets | Zotepine |
| 9515 | Zoleptil 50 tablets (Movianto UK Ltd) | Zotepine |
| 17504 | Zotepine 25mg tablets | Zotepine |
| 31537 | Zuclopenthixol acetate 100mg/2ml solution for injection ampoules | Zuclopenthixol acetate |
| 31538 | Clopixol Acuphase 100mg/2ml solution for injection ampoules (Lundbeck Ltd) | Zuclopenthixol acetate |
| 24270 | Zuclopenthixol acetate 50mg/1ml solution for injection ampoules | Zuclopenthixol acetate |
| 14576 | Zuclopenthixol acetate 50mg/ml oily injection | Zuclopenthixol Acetate |
| 36101 | Clopixol Acuphase 50mg/1ml solution for injection ampoules (Lundbeck Ltd) | Zuclopenthixol acetate |
| 5762 | Clopixol acuphase 50mg/ml Oily injection (Lundbeck Ltd) | Zuclopenthixol Acetate |
| 22049 | Clopixol 200mg/1ml solution for injection ampoules (Lundbeck Ltd) | Zuclopenthixol decanoate |
| 3775 | Zuclopenthixol decanoate 200mg/ml oily injection | Zuclopenthixol Decanoate |
| 28355 | Zuclopenthixol decanoate 200mg/1ml solution for injection ampoules | Zuclopenthixol decanoate |
| 3774 | Clopixol 200mg/ml Oily injection (Lundbeck Ltd) | Zuclopenthixol Decanoate |
| 12224 | Zuclopenthixol decanoate 500mg/1ml solution for injection ampoules | Zuclopenthixol decanoate |
| 12073 | Clopixol Conc 500mg/1ml solution for injection ampoules (Lundbeck Ltd) | Zuclopenthixol decanoate |
| 1319 | Clopixol 10mg tablets (Lundbeck Ltd) | Zuclopenthixol dihydrochloride |
| 13600 | Zuclopenthixol 25mg tablets | Zuclopenthixol dihydrochloride |
| 9347 | Clopixol 25mg tablets (Lundbeck Ltd) | Zuclopenthixol dihydrochloride |
| 13368 | Clopixol 2mg tablets (Lundbeck Ltd) | Zuclopenthixol dihydrochloride |
| 12707 | Zuclopenthixol 2mg tablets | Zuclopenthixol dihydrochloride |
| 9686 | Zuclopenthixol 10mg tablets | Zuclopenthixol dihydrochloride |
| **Antimania Medication** | | |
| **Product Code** | **Product Name** | **Drug substance name** |
| 47280 | Asenapine 5mg sublingual tablets sugar free | Asenapine maleate |
| 47167 | Asenapine 10mg sublingual tablets sugar free | Asenapine maleate |
| 59345 | Sycrest 5mg sublingual tablets (Lundbeck Ltd) | Asenapine maleate |
| 40403 | Carbamazepine 400mg Modified-release tablet (Lagap) | Carbamazepine |
| 41726 | Carbamazepine 100mg Tablet (IVAX Pharmaceuticals UK Ltd) | Carbamazepine |
| 53188 | Carbamazepine 500mg/5ml Oral suspension (Martindale Pharmaceuticals Ltd) | Carbamazepine |
| 37800 | Carbamazepine 200mg Modified-release tablet (Lagap) | Carbamazepine |
| 2824 | Tegretol retard 400mg Modified-release tablet (Novartis Pharmaceuticals UK Ltd) | Carbamazepine |
| 47294 | Carbamazepine sr 400mg Tablet (IVAX Pharmaceuticals UK Ltd) | Carbamazepine |
| 46888 | Carbamazepine 200mg Modified-release tablet (Generics (UK) Ltd) | Carbamazepine |
| 32900 | Carbamazepine sr 200mg Tablet (IVAX Pharmaceuticals UK Ltd) | Carbamazepine |
| 34958 | Carbamazepine 100mg Tablet (Berk Pharmaceuticals Ltd) | Carbamazepine |
| 46972 | Carbamazepine 200mg Tablet (IVAX Pharmaceuticals UK Ltd) | Carbamazepine |
| 43451 | Carbamazepine 400mg Modified-release tablet (Generics (UK) Ltd) | Carbamazepine |
| 2823 | Tegretol retard 200mg Modified-release tablet (Novartis Pharmaceuticals UK Ltd) | Carbamazepine |
| 30509 | Timonil retard 400mg Modified-release tablet (C P Pharmaceuticals Ltd) | Carbamazepine |
| 3359 | Priadel 200mg modified-release tablets (Sanofi) | Lithium carbonate |
| 12402 | Camcolit 250 tablets (Essential Pharma Ltd) | Lithium carbonate |
| 760 | Lithium carbonate 200mg modified-release tablets | Lithium carbonate |
| 8041 | Lithium carbonate 250mg tablets | Lithium carbonate |
| 1447 | Lithium carbonate 400mg modified-release tablets | Lithium carbonate |
| 65301 | Lithium carbonate 250mg tablets (Essential Pharma Ltd) | Lithium carbonate |
| 51401 | Priadel 400mg modified-release tablets (Necessity Supplies Ltd) | Lithium carbonate |
| 14954 | Lithium carbonate 450mg modified-release tablets | Lithium carbonate |
| 65296 | Lithium carbonate 250mg tablets (A A H Pharmaceuticals Ltd) | Lithium carbonate |
| 872 | Priadel 400mg modified-release tablets (Sanofi) | Lithium carbonate |
| 65838 | Lithium carbonate 400mg modified-release tablets (DE Pharmaceuticals) | Lithium carbonate |
| 56435 | Lithium carbonate 200mg/5ml oral suspension | Lithium carbonate |
| 8827 | Lithium carbonate 300mg Modified-release tablet | Lithium Carbonate |
| 66248 | Lithium carbonate 400mg modified-release tablets (Ennogen Healthcare Ltd) | Lithium carbonate |
| 12403 | Camcolit 400 modified-release tablets (Essential Pharma Ltd) | Lithium carbonate |
| 25344 | Lithium carbonate 400mg Modified-release tablet (Approved Prescription Services Ltd) | Lithium carbonate |
| 53459 | Priadel 400mg modified-release tablets (DE Pharmaceuticals) | Lithium carbonate |
| 63989 | Lithium carbonate 400mg modified-release tablets (Niche Pharma Ltd) | Lithium carbonate |
| 3352 | Liskonum 450mg modified-release tablets (Teofarma) | Lithium carbonate |
| 68292 | Lithium carbonate 400mg modified-release tablets (AM Distributions (Yorkshire) Ltd) | Lithium carbonate |
| 15388 | Phasal 300mg Tablet (Lagap) | Lithium Carbonate |
| 22018 | Li-Liquid 509mg/5ml oral solution (Rosemont Pharmaceuticals Ltd) | Lithium citrate |
| 56427 | Lithium citrate 509mg/5ml oral solution (Cubic Pharmaceuticals Ltd) | Lithium citrate |
| 10937 | Lithium citrate 509mg/5ml oral solution | Lithium citrate |
| 10809 | Priadel 520mg/5ml liquid (Sanofi) | Lithium citrate |
| 12648 | Lithium citrate 1.018g/5ml oral solution | Lithium citrate |
| 11491 | Lithium citrate 520mg/5ml oral solution sugar free | Lithium citrate |
| 25345 | Li-Liquid 1.018g/5ml oral solution (Rosemont Pharmaceuticals Ltd) | Lithium citrate |
| 56072 | Olanzapine 20mg orodispersible tablets | Olanzapine |
| 5653 | Olanzapine 7.5mg tablets | Olanzapine |
| 16407 | Zyprexa 15mg Velotabs (Eli Lilly and Company Ltd) | Olanzapine |
| 36163 | Zyprexa 20mg tablets (Eli Lilly and Company Ltd) | Olanzapine |
| 33883 | Zyprexa 20mg Velotabs (Eli Lilly and Company Ltd) | Olanzapine |
| 18453 | Zyprexa 2.5mg tablets (Eli Lilly and Company Ltd) | Olanzapine |
| 13820 | Zyprexa 10mg tablets (Eli Lilly and Company Ltd) | Olanzapine |
| 47098 | Olanzapine 5mg orodispersible tablets sugar free | Olanzapine |
| 63833 | Olanzapine 2.5mg tablets (Zentiva) | Olanzapine |
| 70219 | Olanzapine 5mg orodispersible tablets (Teva UK Ltd) | Olanzapine |
| 56143 | Olanzapine 15mg orodispersible tablets | Olanzapine |
| 65707 | Olanzapine 20mg orodispersible tablets sugar free (Actavis UK Ltd) | Olanzapine |
| 71367 | Olanzapine 5mg orodispersible tablets sugar free (Actavis UK Ltd) | Olanzapine |
| 14717 | Zyprexa 5mg Velotabs (Eli Lilly and Company Ltd) | Olanzapine |
| 29540 | Olanzapine 20mg tablets | Olanzapine |
| 55622 | Olanzapine 10mg orodispersible tablets | Olanzapine |
| 18024 | Zyprexa 5mg tablets (Eli Lilly and Company Ltd) | Olanzapine |
| 57270 | Olanzapine 2.5mg/5ml oral suspension | Olanzapine |
| 69674 | Olanzapine 2.5mg/5ml oral solution | Olanzapine |
| 23431 | Olanzapine 10mg powder for solution for injection vials | Olanzapine |
| 47103 | Olanzapine 15mg orodispersible tablets sugar free | Olanzapine |
| 53848 | Zalasta 5mg orodispersible tablets (Consilient Health Ltd) | Olanzapine |
| 63615 | Olanzapine 2.5mg tablets (Dr Reddy's Laboratories (UK) Ltd) | Olanzapine |
| 61103 | Olanzapine 15mg oral lyophilisates sugar free | Olanzapine |
| 53556 | Olanzapine 10mg oral lyophilisates sugar free | Olanzapine |
| 13888 | Zyprexa 10mg Velotabs (Eli Lilly and Company Ltd) | Olanzapine |
| 1249 | Olanzapine 10mg tablets | Olanzapine |
| 56265 | Olanzapine 20mg oral lyophilisates sugar free | Olanzapine |
| 68346 | Zalasta 20mg tablets (Consilient Health Ltd) | Olanzapine |
| 70640 | Olanzapine 12.5mg/5ml oral suspension | Olanzapine |
| 47093 | Olanzapine 20mg orodispersible tablets sugar free | Olanzapine |
| 71478 | Olanzapine 2.5mg tablets (Alliance Healthcare (Distribution) Ltd) | Olanzapine |
| 45953 | Zyprexa 10mg powder for solution for injection vials (Eli Lilly and Company Ltd) | Olanzapine |
| 69511 | Olanzapine 10mg tablets (Teva UK Ltd) | Olanzapine |
| 57616 | Olanzapine 20mg tablets (Teva UK Ltd) | Olanzapine |
| 59143 | Olanzapine 2.5mg tablets (Teva UK Ltd) | Olanzapine |
| 70367 | Olanzapine 10mg tablets (Alliance Healthcare (Distribution) Ltd) | Olanzapine |
| 58854 | Olanzapine 10mg tablets (Actavis UK Ltd) | Olanzapine |
| 55667 | Olanzapine 15mg tablets (Actavis UK Ltd) | Olanzapine |
| 70415 | Olanzapine 15mg tablets (Teva UK Ltd) | Olanzapine |
| 57160 | Olanzapine 5mg oral lyophilisates sugar free | Olanzapine |
| 6850 | Olanzapine 15mg tablets | Olanzapine |
| 65944 | Olanzapine 2.5mg tablets (Mylan) | Olanzapine |
| 70542 | Olanzapine 5mg orodispersible tablets sugar free (Alliance Healthcare (Distribution) Ltd) | Olanzapine |
| 58147 | Olanzapine 10mg tablets (Zentiva) | Olanzapine |
| 68347 | Zalasta 5mg tablets (Consilient Health Ltd) | Olanzapine |
| 2656 | Olanzapine 2.5mg tablets | Olanzapine |
| 52001 | Olanzapine 2.5mg tablets (Aspire Pharma Ltd) | Olanzapine |
| 50214 | Olanzapine 5mg orodispersible tablets | Olanzapine |
| 47063 | Olanzapine 10mg orodispersible tablets sugar free | Olanzapine |
| 21964 | Zyprexa 7.5mg tablets (Eli Lilly and Company Ltd) | Olanzapine |
| 63155 | Olanzapine 7.5mg tablets (Actavis UK Ltd) | Olanzapine |
| 19976 | Zyprexa 15mg tablets (Eli Lilly and Company Ltd) | Olanzapine |
| 3281 | Olanzapine 5mg tablets | Olanzapine |
| 71670 | Olanzapine 5mg tablets (Alliance Healthcare (Distribution) Ltd) | Olanzapine |
| 25902 | Pentamidine 300mg powder for solution for injection vials | Pentamidine isethionate |
| 36229 | Pentacarinat 300mg powder for solution for injection vials (Sanofi) | Pentamidine isethionate |
| 37504 | Sodium stibogluconate 10g/100ml solution for injection vials | Sodium stibogluconate |
| 36398 | Pentostam 10g/100ml solution for injection vials (GlaxoSmithKline UK Ltd) | Sodium stibogluconate |
| 36634 | Episenta 1000mg modified-release granules sachets (Desitin Pharma Ltd) | Sodium valproate |
| 37584 | Episenta 500mg modified-release granules sachets (Desitin Pharma Ltd) | Sodium valproate |
| 7064 | Depakote 250mg gastro-resistant tablets (Sanofi) | Valproate semisodium |
| 53211 | Valproic acid 500mg/5ml oral solution | Valproate semisodium |
| 9759 | Depakote 500mg gastro-resistant tablets (Sanofi) | Valproate semisodium |
| 6305 | Valproic acid 500mg gastro-resistant tablets | Valproate semisodium |
| 5848 | Valproic acid 250mg gastro-resistant tablets | Valproate semisodium |
