# Supplementary material for: Safety of antidepressants in a primary care cohort of adults with obesity and depression
Source: PLoS One. 2021 Jan 29;16(1):e0245722. doi: 10.1371/journal.pone.0245722 (PMC7846000; doi:10.1371/journal.pone.0245722)
Supplement: S5 Table — (DOCX) [file pone.0245722.s008.docx]

**Table S5. Read code diagnoses of bipolar disorders, psychotic disorder or dementia**

| **Bipolar disorder** | |
| --- | --- |
| **Read Code** | **Read Code Description** |
| Eu31.11 | [X]Manic-depressive illness |
| Eu30000 | [X]Hypomania |
| E114.00 | Bipolar affective disorder, currently manic |
| E115.00 | Bipolar affective disorder, currently depressed |
| Eu30z11 | [X]Mania NOS |
| Eu31500 | [X]Bipolar affect dis cur epi severe depres with psyc symp |
| Eu3..00 | [X]Mood - affective disorders |
| Eu31.12 | [X]Manic-depressive psychosis |
| Eu31.00 | [X]Bipolar affective disorder |
| E11..11 | Bipolar psychoses |
| Eu30.11 | [X]Bipolar disorder, single manic episode |
| 146D.00 | H/O: manic depressive disorder |
| E11y000 | Unspecified manic-depressive psychoses |
| Eu30.00 | [X]Manic episode |
| E115.11 | Manic-depressive - now depressed |
| Eu30100 | [X]Mania without psychotic symptoms |
| E11..00 | Affective psychoses |
| E110100 | Single manic episode, mild |
| E117.00 | Unspecified bipolar affective disorder |
| E115000 | Bipolar affective disorder, currently depressed, unspecified |
| E114300 | Bipolar affect disord, currently manic, severe, no psychosis |
| Eu31300 | [X]Bipolar affect disorder cur epi mild or moderate depressn |
| Eu31000 | [X]Bipolar affective disorder, current episode hypomanic |
| E114.11 | Manic-depressive - now manic |
| E110.11 | Hypomanic psychoses |
| E111000 | Recurrent manic episodes, unspecified |
| E110000 | Single manic episode, unspecified |
| Eu30200 | [X]Mania with psychotic symptoms |
| Eu34000 | [X]Cyclothymia |
| ZV11112 | [V]Personal history of manic-depressive psychosis |
| 1S42.00 | Manic mood |
| Eu31400 | [X]Bipol aff disord, curr epis sev depress, no psychot symp |
| ZV11111 | [V]Personal history of manic-depressive psychosis |
| E117600 | Unspecified bipolar affective disorder, in full remission |
| E110200 | Single manic episode, moderate |
| E116100 | Mixed bipolar affective disorder, mild |
| E11..13 | Manic psychoses |
| E111.00 | Recurrent manic episodes |
| Eu31100 | [X]Bipolar affect disorder cur epi manic wout psychotic symp |
| Eu31700 | [X]Bipolar affective disorder, currently in remission |
| E111200 | Recurrent manic episodes, moderate |
| E115200 | Bipolar affective disorder, currently depressed, moderate |
| E117z00 | Unspecified bipolar affective disorder, NOS |
| Eu3y.00 | [X]Other mood affective disorders |
| Eu31200 | [X]Bipolar affect disorder cur epi manic with psychotic symp |
| Eu33312 | [X]Manic-depress psychosis,depressed type+psychotic symptoms |
| Eu33213 | [X]Manic-depress psychosis,depressd,no psychotic symptoms |
| Eu3yy00 | [X]Other specified mood affective disorders |
| Eu3y100 | [X]Other recurrent mood affective disorders |
| Eu3y011 | [X]Mixed affective episode |
| E116.00 | Mixed bipolar affective disorder |
| E116000 | Mixed bipolar affective disorder, unspecified |
| Eu3z.11 | [X]Affective psychosis NOS |
| Eu30y00 | [X]Other manic episodes |
| E111400 | Recurrent manic episodes, severe, with psychosis |
| E11yz00 | Other and unspecified manic-depressive psychoses NOS |
| Eu31z00 | [X]Bipolar affective disorder, unspecified |
| E115300 | Bipolar affect disord, now depressed, severe, no psychosis |
| E115100 | Bipolar affective disorder, currently depressed, mild |
| E114000 | Bipolar affective disorder, currently manic, unspecified |
| E114100 | Bipolar affective disorder, currently manic, mild |
| E110z00 | Manic disorder, single episode NOS |
| E110.00 | Manic disorder, single episode |
| Eu3z.00 | [X]Unspecified mood affective disorder |
| Eu30211 | [X]Mania with mood-congruent psychotic symptoms |
| E111600 | Recurrent manic episodes, in full remission |
| E115z00 | Bipolar affective disorder, currently depressed, NOS |
| Eu34z00 | [X]Persistent mood affective disorder, unspecified |
| Eu34.00 | [X]Persistent mood affective disorders |
| Eu30z00 | [X]Manic episode, unspecified |
| Eu31600 | [X]Bipolar affective disorder, current episode mixed |
| E111z00 | Recurrent manic episode NOS |
| E111100 | Recurrent manic episodes, mild |
| E114200 | Bipolar affective disorder, currently manic, moderate |
| Eu30212 | [X]Mania with mood-incongruent psychotic symptoms |
| E117000 | Unspecified bipolar affective disorder, unspecified |
| Eu34y00 | [X]Other persistent mood affective disorders |
| Eu3y000 | [X]Other single mood affective disorders |
| Eu31y12 | [X]Recurrent manic episodes |
| Eu31y00 | [X]Other bipolar affective disorders |
| E116400 | Mixed bipolar affective disorder, severe, with psychosis |
| E116600 | Mixed bipolar affective disorder, in full remission |
| E114400 | Bipolar affect disord, currently manic,severe with psychosis |
| E115600 | Bipolar affective disorder, now depressed, in full remission |
| E114z00 | Bipolar affective disorder, currently manic, NOS |
| E111500 | Recurrent manic episodes, partial or unspecified remission |
| E114500 | Bipolar affect disord,currently manic, part/unspec remission |
| E11y.00 | Other and unspecified manic-depressive psychoses |
| E116200 | Mixed bipolar affective disorder, moderate |
| E116300 | Mixed bipolar affective disorder, severe, without psychosis |
| E116z00 | Mixed bipolar affective disorder, NOS |
| E116500 | Mixed bipolar affective disorder, partial/unspec remission |
| E117100 | Unspecified bipolar affective disorder, mild |
| E115400 | Bipolar affect disord, now depressed, severe with psychosis |
| E114600 | Bipolar affective disorder, currently manic, full remission |
| E111300 | Recurrent manic episodes, severe without mention psychosis |
| Eu31.13 | [X]Manic-depressive reaction |
| E117400 | Unspecified bipolar affective disorder,severe with psychosis |
| E117200 | Unspecified bipolar affective disorder, moderate |
| E110600 | Single manic episode in full remission |
| E11y300 | Other mixed manic-depressive psychoses |
| E117500 | Unspecified bipolar affect disord, partial/unspec remission |
| E11y100 | Atypical manic disorder |
| E115500 | Bipolar affect disord, now depressed, part/unspec remission |
| E117300 | Unspecified bipolar affective disorder, severe, no psychosis |
| Eu31y11 | [X]Bipolar II disorder |
| **Psychotic disorders** | |
| **Read Code** | **Read Code Description** |
| **readcode** | **readterm** |
| E104.00 | Acute schizophrenic episode |
| Eu2z.11 | [X]Psychosis NOS |
| E10..00 | Schizophrenic disorders |
| E103.00 | Paranoid schizophrenia |
| 1BH..00 | Delusions |
| Eu22011 | [X]Paranoid psychosis |
| E107.00 | Schizo-affective schizophrenia |
| R001.00 | [D]Hallucinations |
| E212.00 | Schizoid personality disorder |
| E13z.11 | Psychotic episode NOS |
| E121.00 | Chronic paranoid psychosis |
| E100200 | Chronic schizophrenic |
| 285..11 | Psychotic condition, insight present |
| Eu22015 | [X]Paranoia |
| 1464 | H/O: schizophrenia |
| E10z.00 | Schizophrenia NOS |
| E130.00 | Reactive depressive psychosis |
| Eu0z.12 | [X]Symptomatic psychosis NOS |
| E103z00 | Paranoid schizophrenia NOS |
| Eu25.00 | [X]Schizoaffective disorders |
| E107z00 | Schizo-affective schizophrenia NOS |
| Eu25100 | [X]Schizoaffective disorder, depressive type |
| Eu22012 | [X]Paranoid state |
| Eu23200 | [X]Acute schizophrenia-like psychotic disorder |
| R001400 | [D]Visual hallucinations |
| Eu32300 | [X]Severe depressive episode with psychotic symptoms |
| R001000 | [D]Hallucinations, auditory |
| E12z.00 | Paranoid psychosis NOS |
| 146H.00 | H/O: psychosis |
| E212z00 | Schizoid personality disorder NOS |
| E13z.00 | Nonorganic psychosis NOS |
| E133.00 | Acute paranoid reaction |
| E100000 | Unspecified schizophrenia |
| E1...00 | Non-organic psychoses |
| E13y.00 | Other reactive psychoses |
| E1y..00 | Other specified non-organic psychoses |
| Eu20000 | [X]Paranoid schizophrenia |
| Eu33315 | [X]Recurrent severe episodes of psychotic depression |
| Eu25011 | [X]Schizoaffective psychosis, manic type |
| Eu2..00 | [X]Schizophrenia, schizotypal and delusional disorders |
| E130.11 | Psychotic reactive depression |
| 1BH..11 | Delusion |
| Eu20y13 | [X]Schizophrenifrm psychos NOS |
| R001z00 | [D]Hallucinations NOS |
| E13..11 | Reactive psychoses |
| Eu20211 | [X]Catatonic stupor |
| Eu23012 | [X]Cycloid psychosis |
| Eu23100 | [X]Acute polymorphic psychot disord with symp of schizophren |
| ZV11000 | [V]Personal history of schizophrenia |
| E1z..00 | Non-organic psychosis NOS |
| 1BH3.00 | Paranoid ideation |
| 286..11 | Poor insight into psychotic condition |
| E13y100 | Brief reactive psychosis |
| E100100 | Subchronic schizophrenia |
| Eu33311 | [X]Endogenous depression with psychotic symptoms |
| Eu20511 | [X]Chronic undifferentiated schizophrenia |
| Eu32313 | [X]Single episode of psychotic depression |
| Eu32311 | [X]Single episode of major depression and psychotic symptoms |
| E134.00 | Psychogenic paranoid psychosis |
| Eu23.00 | [X]Acute and transient psychotic disorders |
| R001200 | [D]Hallucinations, olfactory |
| E102.00 | Catatonic schizophrenia |
| E13yz00 | Other reactive psychoses NOS |
| Eu23112 | [X]Cycloid psychosis with symptoms of schizophrenia |
| 8HHs.00 | Referral to psychosis early intervention service |
| Eu21.18 | [X]Schizotypal personality disorder |
| Eu23312 | [X]Psychogenic paranoid psychosis |
| Eu44.14 | [X]Hysterical psychosis |
| Eu22.00 | [X]Persistent delusional disorders |
| Eu32314 | [X]Single episode of reactive depressive psychosis |
| Eu23z12 | [X]Reactive psychosis |
| E131.00 | Acute hysterical psychosis |
| E101.00 | Hebephrenic schizophrenia |
| Eu2y.00 | [X]Other nonorganic psychotic disorders |
| E103200 | Chronic paranoid schizophrenia |
| Eu20214 | [X]Schizophrenic flexibilatis cerea |
| Eu23z11 | [X]Brief reactive psychosis NOS |
| Eu2y.11 | [X]Chronic hallucinatory psychosis |
| Eu33314 | [X]Recurr severe episodes/psychogenic depressive psychosis |
| E13..00 | Other nonorganic psychoses |
| E100.00 | Simple schizophrenia |
| 1BH1.00 | Grandiose delusions |
| Eu33313 | [X]Recurr severe episodes/major depression+psychotic symptom |
| E10y000 | Atypical schizophrenia |
| E103000 | Unspecified paranoid schizophrenia |
| Eu25z11 | [X]Schizoaffective psychosis NOS |
| E11zz00 | Other affective psychosis NOS |
| Eu25200 | [X]Schizoaffective disorder, mixed type |
| Eu25000 | [X]Schizoaffective disorder, manic type |
| Eu23z00 | [X]Acute and transient psychotic disorder, unspecified |
| Eu20.00 | [X]Schizophrenia |
| Eu22000 | [X]Delusional disorder |
| Eu20z00 | [X]Schizophrenia, unspecified |
| Eu25111 | [X]Schizoaffective psychosis, depressive type |
| Eu20600 | [X]Simple schizophrenia |
| Eu20213 | [X]Schizophrenic catatonia |
| E103500 | Paranoid schizophrenia in remission |
| Eu23000 | [X]Acute polymorphic psychot disord without symp of schizoph |
| Eu25212 | [X]Mixed schizophrenic and affective psychosis |
| Eu25z00 | [X]Schizoaffective disorder, unspecified |
| Eu33316 | [X]Recurrent severe episodes/reactive depressive psychosis |
| E106.00 | Residual schizophrenia |
| Eu60100 | [X]Schizoid personality disorder |
| E10y.00 | Other schizophrenia |
| Eu21.00 | [X]Schizotypal disorder |
| Eu21.15 | [X]Prodromal schizophrenia |
| Eu22y11 | [X]Delusional dysmorphophobia |
| Eu25112 | [X]Schizophreniform psychosis, depressive type |
| E141100 | Residual disintegrative psychoses |
| E11z.00 | Other and unspecified affective psychoses |
| E14..00 | Psychoses with origin in childhood |
| Eu20100 | [X]Hebephrenic schizophrenia |
| 1BH0.00 | Delusion of persecution |
| E107200 | Chronic schizo-affective schizophrenia |
| Eu23300 | [X]Other acute predominantly delusional psychotic disorders |
| E100400 | Acute exacerbation of chronic schizophrenia |
| Eu23y00 | [X]Other acute and transient psychotic disorders |
| Eu33300 | [X]Recurrent depress disorder cur epi severe with psyc symp |
| Eu02z12 | [X] Presenile psychosis NOS |
| Eu22013 | [X]Paraphrenia - late |
| E101z00 | Hebephrenic schizophrenia NOS |
| Eu22z00 | [X]Persistent delusional disorder, unspecified |
| Eu20y00 | [X]Other schizophrenia |
| E10yz00 | Other schizophrenia NOS |
| Eu21.16 | [X]Pseudoneurotic schizophrenia |
| Eu20011 | [X]Paraphrenic schizophrenia |
| Eu22y12 | [X]Involutional paranoid state |
| E103300 | Acute exacerbation of subchronic paranoid schizophrenia |
| Eu25012 | [X]Schizophreniform psychosis, manic type |
| Eu32312 | [X]Single episode of psychogenic depressive psychosis |
| E103400 | Acute exacerbation of chronic paranoid schizophrenia |
| E100z00 | Simple schizophrenia NOS |
| Ryu5300 | [X]Other hallucinations |
| Eu84314 | [X]Symbiotic psychosis |
| Eu20111 | [X]Disorganised schizophrenia |
| R001100 | [D]Hallucinations, gustatory |
| Eu21.12 | [X]Borderline schizophrenia |
| Eu22111 | [X]Capgras syndrome |
| Eu22y13 | [X]Paranoia querulans |
| 1BH2.00 | Ideas of reference |
| E141.00 | Disintegrative psychosis |
| E107500 | Schizo-affective schizophrenia in remission |
| ZS7C611 | Schizophrenic language |
| E100300 | Acute exacerbation of subchronic schizophrenia |
| Eu25y00 | [X]Other schizoaffective disorders |
| E100500 | Schizophrenia in remission |
| E102000 | Unspecified catatonic schizophrenia |
| E107000 | Unspecified schizo-affective schizophrenia |
| E107300 | Acute exacerbation subchronic schizo-affective schizophrenia |
| Eu23211 | [X]Brief schizophreniform disorder |
| Eu20300 | [X]Undifferentiated schizophrenia |
| E107100 | Subchronic schizo-affective schizophrenia |
| Eu20200 | [X]Catatonic schizophrenia |
| E212200 | Schizotypal personality |
| Eu84312 | [X]Disintegrative psychosis |
| Eu22100 | [X]Delusional misidentification syndrome |
| Eu21.14 | [X]Prepsychotic schizophrenia |
| E107400 | Acute exacerbation of chronic schizo-affective schizophrenia |
| E102z00 | Catatonic schizophrenia NOS |
| R001300 | [D]Hallucinations, tactile |
| Eu20500 | [X]Residual schizophrenia |
| Eu20212 | [X]Schizophrenic catalepsy |
| Eu21.13 | [X]Latent schizophrenia |
| Eu22014 | [X]Sensitiver Beziehungswahn |
| Eu22y00 | [X]Other persistent delusional disorders |
| E105.00 | Latent schizophrenia |
| E101000 | Unspecified hebephrenic schizophrenia |
| E212000 | Unspecified schizoid personality disorder |
| E101500 | Hebephrenic schizophrenia in remission |
| Eu23212 | [X]Brief schizophrenifrm psych |
| E100.11 | Schizophrenia simplex |
| Eu21.11 | [X]Latent schizophrenic reaction |
| Eu20311 | [X]Atypical schizophrenia |
| E10y.11 | Cenesthopathic schizophrenia |
| Eu20y12 | [X]Schizophreniform disord NOS |
| E105200 | Chronic latent schizophrenia |
| Eu23214 | [X]Schizophrenic reaction |
| E105500 | Latent schizophrenia in remission |
| E101400 | Acute exacerbation of chronic hebephrenic schizophrenia |
| Eu32800 | [X]Major depression, severe with psychotic symptoms |
| E107.11 | Cyclic schizophrenia |
| E10y100 | Coenesthopathic schizophrenia |
| E102100 | Subchronic catatonic schizophrenia |
| Eu32900 | [X]Single major depr ep, severe with psych, psych in remiss |
| Eu32A00 | [X]Recurr major depr ep, severe with psych, psych in remiss |
| E105000 | Unspecified latent schizophrenia |
| E102500 | Catatonic schizophrenia in remission |
| E105z00 | Latent schizophrenia NOS |
| 38C1400 | Assessment of cause of psychotic and behavioural symptoms |
| **Dementia** | |
| **Read Code** | **Read Code Description** |
| E00..12 | Senile/presenile dementia |
| E00..11 | Senile dementia |
| F110.00 | Alzheimer's disease |
| E00z.00 | Senile or presenile psychoses NOS |
| Eu02z14 | [X] Senile dementia NOS |
| Eu02z00 | [X] Unspecified dementia |
| Eu01.00 | [X]Vascular dementia |
| E000.00 | Uncomplicated senile dementia |
| Eu00.00 | [X]Dementia in Alzheimer's disease |
| Eu00z11 | [X]Alzheimer's dementia unspec |
| E004.11 | Multi infarct dementia |
| Eu01200 | [X]Subcortical vascular dementia |
| Eu02300 | [X]Dementia in Parkinson's disease |
| Eu01.11 | [X]Arteriosclerotic dementia |
| Eu01100 | [X]Multi-infarct dementia |
| Eu00112 | [X]Senile dementia,Alzheimer's type |
| Eu02.00 | [X]Dementia in other diseases classified elsewhere |
| 6AB..00 | Dementia annual review |
| E001.00 | Presenile dementia |
| E00y.00 | Other senile and presenile organic psychoses |
| F110000 | Alzheimer's disease with early onset |
| E002000 | Senile dementia with paranoia |
| Eu01z00 | [X]Vascular dementia, unspecified |
| E004.00 | Arteriosclerotic dementia |
| E004.00 | Arteriosclerotic dementia |
| E002100 | Senile dementia with depression |
| E041.00 | Dementia in conditions EC |
| Eu00011 | [X]Presenile dementia,Alzheimer's type |
| Eu02500 | [X]Lewy body dementia |
| E012.11 | Alcoholic dementia NOS |
| E001300 | Presenile dementia with depression |
| Eu02z16 | [X] Senile dementia, depressed or paranoid type |
| Eu02z15 | [X] Senile psychosis NOS |
| Eu02000 | [X]Dementia in Pick's disease |
| Eu00z00 | [X]Dementia in Alzheimer's disease, unspecified |
| F112.00 | Senile degeneration of brain |
| E001200 | Presenile dementia with paranoia |
| Eu00200 | [X]Dementia in Alzheimer's dis, atypical or mixed type |
| Eu01300 | [X]Mixed cortical and subcortical vascular dementia |
| F110100 | Alzheimer's disease with late onset |
| E00..00 | Senile and presenile organic psychotic conditions |
| Eu02z13 | [X] Primary degenerative dementia NOS |
| Eu02200 | [X]Dementia in Huntington's disease |
| E003.00 | Senile dementia with delirium |
| E003.00 | Senile dementia with delirium |
| E012000 | Chronic alcoholic brain syndrome |
| A411.00 | Jakob-Creutzfeldt disease |
| E001z00 | Presenile dementia NOS |
| Eu00100 | [X]Dementia in Alzheimer's disease with late onset |
| E002z00 | Senile dementia with depressive or paranoid features |
| E004z00 | Arteriosclerotic dementia NOS |
| E001000 | Uncomplicated presenile dementia |
| E004000 | Uncomplicated arteriosclerotic dementia |
| E004300 | Arteriosclerotic dementia with depression |
| Eu00113 | [X]Primary degen dementia of Alzheimer's type, senile onset |
| E002.00 | Senile dementia with depressive or paranoid features |
| Eu01000 | [X]Vascular dementia of acute onset |
| Eu00111 | [X]Alzheimer's disease type 1 |
| F11x000 | Cerebral degeneration due to alcoholism |
| Eu02z12 | [X] Presenile psychosis NOS |
| Eu02z11 | [X] Presenile dementia NOS |
| F11x700 | Cerebral degeneration due to Jakob - Creutzfeldt disease |
| Eu00000 | [X]Dementia in Alzheimer's disease with early onset |
| E001100 | Presenile dementia with delirium |
| 9Ou1.00 | Dementia monitoring first letter |
| E00y.11 | Presbyophrenic psychosis |
| Eu04100 | [X]Delirium superimposed on dementia |
| Eu02100 | [X]Dementia in Creutzfeldt-Jakob disease |
| E012.00 | Other alcoholic dementia |
| 66h..00 | Dementia monitoring |
| Eu01y00 | [X]Other vascular dementia |
| E004200 | Arteriosclerotic dementia with paranoia |
| Eu01111 | [X]Predominantly cortical dementia |
| E004100 | Arteriosclerotic dementia with delirium |
| Fyu3000 | [X]Other Alzheimer's disease |
| Eu00012 | [X]Primary degen dementia, Alzheimer's type, presenile onset |
| Eu00013 | [X]Alzheimer's disease type 2 |
| E02y100 | Drug-induced dementia |
| Eu02y00 | [X]Dementia in other specified diseases classif elsewhere |
| 9Ou2.00 | Dementia monitoring second letter |
| 9Ou..00 | Dementia monitoring administration |
| 9Ou3.00 | Dementia monitoring third letter |
| 9Ou4.00 | Dementia monitoring verbal invite |
| 8Hla.00 | Referral to dementia care advisor |
| 8CSA.00 | Dementia advance care plan agreed |
| 8IAe000 | Dementia advance care plan declined |
| 8CMe000 | Dementia advance care plan |
| 8T05100 | Referral to dementia support organisation declined |
| 8IAe200 | Dementia advance care plan review declined |
