# Supplementary material for: Safety of antidepressants in a primary care cohort of adults with obesity and depression
Source: PLoS One. 2021 Jan 29;16(1):e0245722. doi: 10.1371/journal.pone.0245722 (PMC7846000; doi:10.1371/journal.pone.0245722)
Supplement: S6 Table — (DOCX) [file pone.0245722.s009.docx]

**Table S6. Product codes for anticholinesterase medication**

| **Product Code** | **Product Name** | **Drug substance name** |
| --- | --- | --- |
| 2930 | Donepezil 5mg tablets | Donepezil hydrochloride |
| 2931 | Donepezil 10mg tablets | Donepezil hydrochloride |
| 4597 | Rivastigmine 1.5mg capsules | Rivastigmine hydrogen tartrate |
| 5247 | Aricept 10mg tablets (Eisai Ltd) | Donepezil hydrochloride |
| 5334 | Reminyl 12mg tablets (Shire Pharmaceuticals Ltd) | Galantamine hydrobromide |
| 5400 | Aricept 5mg tablets (Eisai Ltd) | Donepezil hydrochloride |
| 5616 | Exelon 6mg capsules (Novartis Pharmaceuticals UK Ltd) | Rivastigmine hydrogen tartrate |
| 7329 | Galantamine 20mg/5ml oral solution sugar free | Galantamine hydrobromide |
| 7361 | Galantamine 24mg modified-release capsules | Galantamine hydrobromide |
| 9854 | Reminyl 4mg tablets (Shire Pharmaceuticals Ltd) | Galantamine hydrobromide |
| 9966 | Ebixa 5mg/0.5ml pump actuation oral solution (Lundbeck Ltd) | Memantine hydrochloride |
| 10187 | Galantamine 4mg tablets | Galantamine hydrobromide |
| 10255 | Galantamine 8mg modified-release capsules | Galantamine hydrobromide |
| 11546 | Exelon 1.5mg capsules (Novartis Pharmaceuticals UK Ltd) | Rivastigmine hydrogen tartrate |
| 11635 | Galantamine 12mg tablets | Galantamine hydrobromide |
| 11654 | Galantamine 8mg tablets | Galantamine hydrobromide |
| 11716 | Exelon 3mg capsules (Novartis Pharmaceuticals UK Ltd) | Rivastigmine hydrogen tartrate |
| 11751 | Rivastigmine 3mg capsules | Rivastigmine hydrogen tartrate |
| 11752 | Rivastigmine 4.5mg capsules | Rivastigmine hydrogen tartrate |
| 11827 | Rivastigmine 2mg/ml oral solution sugar free | Rivastigmine hydrogen tartrate |
| 14309 | Galantamine 16mg modified-release capsules | Galantamine hydrobromide |
| 18587 | Reminyl XL 8mg capsules (Shire Pharmaceuticals Ltd) | Galantamine hydrobromide |
| 18800 | Ebixa 10mg tablets (Lundbeck Ltd) | Memantine hydrochloride |
| 20140 | Reminyl XL 16mg capsules (Shire Pharmaceuticals Ltd) | Galantamine hydrobromide |
| 24088 | Reminyl XL 24mg capsules (Shire Pharmaceuticals Ltd) | Galantamine hydrobromide |
| 29288 | Reminyl 4mg/ml oral solution (Shire Pharmaceuticals Ltd) | Galantamine hydrobromide |
| 35088 | Donepezil 10mg orodispersible tablets sugar free | Donepezil hydrochloride |
| 35179 | Donepezil 5mg orodispersible tablets sugar free | Donepezil hydrochloride |
| 36848 | Aricept Evess 5mg orodispersible tablets (Eisai Ltd) | Donepezil hydrochloride |
| 36976 | Rivastigmine 4.6mg/24hours transdermal patches | Rivastigmine |
| 37188 | Aricept Evess 10mg orodispersible tablets (Eisai Ltd) | Donepezil hydrochloride |
| 37444 | Exelon 4.6mg/24hours transdermal patches (Novartis Pharmaceuticals UK Ltd) | Rivastigmine |
| 37957 | Exelon 9.5mg/24hours transdermal patches (Novartis Pharmaceuticals UK Ltd) | Rivastigmine |
| 38976 | Memantine 5mg+10mg+15mg+20mg Tablet | Memantine Hydrochloride |
| 39240 | Memantine 20mg tablets | Memantine hydrochloride |
| 39362 | Ebixa tablets treatment initiation pack (Lundbeck Ltd) | Memantine Hydrochloride |
| 39363 | Ebixa 20mg tablets (Lundbeck Ltd) | Memantine hydrochloride |
| 48015 | Galsya XL 24mg capsules (Consilient Health Ltd) | Galantamine hydrobromide |
| 48443 | Donepezil 10mg orodispersible tablets | Donepezil hydrochloride |
| 48482 | Galsya XL 8mg capsules (Consilient Health Ltd) | Galantamine hydrobromide |
| 53842 | Aricept 5mg tablets (Waymade Healthcare Plc) | Donepezil hydrochloride |
| 53882 | Rivastigmine 2mg/ml oral solution | Rivastigmine hydrogen tartrate |
| 53922 | Donepezil 10mg orodispersible tablets (Consilient Health Ltd) | Donepezil hydrochloride |
| 55720 | Gatalin XL 24mg capsules (Aspire Pharma Ltd) | Galantamine hydrobromide |
| 55928 | Exelon 4.5mg capsules (Waymade Healthcare Plc) | Rivastigmine hydrogen tartrate |
| 56421 | Gatalin XL 8mg capsules (Aspire Pharma Ltd) | Galantamine hydrobromide |
| 56600 | Donepezil 5mg tablets (Zentiva) | Donepezil hydrochloride |
| 56631 | Rivastigmine 13.3mg/24hours transdermal patches | Rivastigmine |
| 56709 | Gatalin XL 16mg capsules (Aspire Pharma Ltd) | Galantamine hydrobromide |
| 56771 | Rivastigmine 3mg capsules (Dr Reddy's Laboratories (UK) Ltd) | Rivastigmine hydrogen tartrate |
| 57139 | Ebixa 10mg tablets (DE Pharmaceuticals) | Memantine hydrochloride |
| 57171 | Erastig 9.5mg/24hours transdermal patches (Teva UK Ltd) | Rivastigmine |
| 58709 | Donepezil 10mg tablets (A A H Pharmaceuticals Ltd) | Donepezil hydrochloride |
| 58780 | Voleze 9.5mg/24hours transdermal patches (Focus Pharmaceuticals Ltd) | Rivastigmine |
| 58937 | Exelon 13.3mg/24hours transdermal patches (Novartis Pharmaceuticals UK Ltd) | Rivastigmine |
| 58947 | Donepezil 10mg tablets (Accord Healthcare Ltd) | Donepezil hydrochloride |
| 58969 | Rivastigmine 4.6mg/24hours transdermal patches (A A H Pharmaceuticals Ltd) | Rivastigmine |
| 59330 | Voleze 4.6mg/24hours transdermal patches (Focus Pharmaceuticals Ltd) | Rivastigmine |
| 59871 | Donepezil 10mg/5ml oral suspension | Donepezil hydrochloride |
| 59993 | Galzemic XL 16mg capsules (Creo Pharma Ltd) | Galantamine hydrobromide |
| 60107 | Donepezil 5mg tablets (Alliance Healthcare (Distribution) Ltd) | Donepezil hydrochloride |
| 60192 | Galzemic XL 8mg capsules (Creo Pharma Ltd) | Galantamine hydrobromide |
| 60723 | Rivastigmine 6mg capsules (Waymade Healthcare Plc) | Rivastigmine hydrogen tartrate |
| 61385 | Nemdatine 10mg tablets (Actavis UK Ltd) | Memantine hydrochloride |
| 61476 | Acumor XL 24mg capsules (Mylan) | Galantamine hydrobromide |
| 61618 | Nemdatine 20mg tablets (Actavis UK Ltd) | Memantine hydrochloride |
| 61676 | Donepezil 1mg/ml oral solution sugar free | Donepezil hydrochloride |
| 61920 | Luventa XL 8mg capsules (Fontus Health Ltd) | Galantamine hydrobromide |
| 61921 | Luventa XL 24mg capsules (Fontus Health Ltd) | Galantamine hydrobromide |
| 62164 | Alzest 9.5mg/24hours transdermal patches (Dr Reddy's Laboratories (UK) Ltd) | Rivastigmine |
| 62780 | Alzest 4.6mg/24hours transdermal patches (Dr Reddy's Laboratories (UK) Ltd) | Rivastigmine |
| 62867 | Gazylan XL 16mg capsules (Teva UK Ltd) | Galantamine hydrobromide |
| 62868 | Gazylan XL 24mg capsules (Teva UK Ltd) | Galantamine hydrobromide |
| 62925 | Acumor XL 16mg capsules (Mylan) | Galantamine hydrobromide |
| 63217 | Donepezil 5mg tablets (A A H Pharmaceuticals Ltd) | Donepezil hydrochloride |
| 63226 | Prometax 9.5mg/24hours transdermal patches (Novartis Pharmaceuticals UK Ltd) | Rivastigmine |
| 63360 | Luventa XL 16mg capsules (Fontus Health Ltd) | Galantamine hydrobromide |
| 63405 | Galsya XL 16mg capsules (Consilient Health Ltd) | Galantamine hydrobromide |
| 63951 | Rivastigmine 9.5mg/24hours transdermal patches (Actavis UK Ltd) | Rivastigmine |
| 65333 | Memantine 10mg/ml oral solution sugar free (Alliance Healthcare (Distribution) Ltd) | Memantine hydrochloride |
| 65501 | Eluden 4.6mg/24hours transdermal patches (Mylan) | Rivastigmine |
| 65534 | Donepezil 5mg orodispersible tablets sugar free (A A H Pharmaceuticals Ltd) | Donepezil hydrochloride |
| 65573 | Gazylan XL 8mg capsules (Teva UK Ltd) | Galantamine hydrobromide |
| 65761 | Eluden 9.5mg/24hours transdermal patches (Mylan) | Rivastigmine |
| 66899 | Memantine 20mg orodispersible tablets sugar free | Memantine hydrochloride |
| 66934 | Memantine 10mg orodispersible tablets sugar free | Memantine hydrochloride |
| 67593 | Donepezil 10mg tablets (Zentiva) | Donepezil hydrochloride |
| 68493 | Nemdatine tablets treatment initiation pack (Actavis UK Ltd) |  |
| 68494 | Rivastigmine 6mg capsules (A A H Pharmaceuticals Ltd) | Rivastigmine hydrogen tartrate |
| 68792 | Memantine 10mg/ml oral solution sugar free (Chanelle Medical UK Ltd) | Memantine hydrochloride |
| 68802 | Donepezil 5mg tablets (Waymade Healthcare Plc) | Donepezil hydrochloride |
| 68845 | Memantine 10mg/ml oral solution sugar free (A A H Pharmaceuticals Ltd) | Memantine hydrochloride |
| 69564 | Prometax 4.6mg/24hours transdermal patches (Novartis Pharmaceuticals UK Ltd) | Rivastigmine |
| 69595 | Marixino 20mg tablets (Consilient Health Ltd) |  |
| 69638 | Memantine 5mg/10mg/15mg/20mg 4 week treatment initiation pack (Lupin (Europe) Ltd) | Memantine hydrochloride |
| 69859 | Rivastigmine 4.6mg/24hours transdermal patches (Actavis UK Ltd) | Rivastigmine |
| 69971 | Memantine 10mg/ml oral solution sugar free (Zentiva) | Memantine hydrochloride |
| 70069 | Rivastigmine 9.5mg/24hours transdermal patches (A A H Pharmaceuticals Ltd) | Rivastigmine |
| 70496 | Memantine 10mg soluble tablets sugar free | Memantine hydrochloride |
| 70503 | Memantine 20mg soluble tablets sugar free | Memantine hydrochloride |
| 70826 | Rivastigmine 9.5mg/24hours transdermal patches (DE Pharmaceuticals) | Rivastigmine |
| 71187 | Memantine 10mg tablets (A A H Pharmaceuticals Ltd) | Memantine hydrochloride |
| 71960 | Donepezil 5mg tablets (Accord Healthcare Ltd) | Donepezil hydrochloride |
