# Supplementary material for: Safety of antidepressants in a primary care cohort of adults with obesity and depression
Source: PLoS One. 2021 Jan 29;16(1):e0245722. doi: 10.1371/journal.pone.0245722 (PMC7846000; doi:10.1371/journal.pone.0245722)
Supplement: S7 Table — (DOCX) [file pone.0245722.s010.docx]

**Table S7. Product codes for antidepressant medication**

| **Product Code** | **Product Name** | **British National Formulary Header** |
| --- | --- | --- |
| 22 | Fluoxetine 20mg capsules | Selective Serotonin Re-uptake Inhibitors |
| 49 | Amitriptyline 25mg tablets | Tricyclic And Related Antidepressant Drugs/Neuropathic Pain/Prophylaxis Of Migraine |
| 50 | Paroxetine 20mg tablets | Selective Serotonin Re-uptake Inhibitors |
| 67 | Citalopram 20mg tablets | Selective Serotonin Re-uptake Inhibitors |
| 74 | Dosulepin 75mg tablets | Tricyclic And Related Antidepressant Drugs |
| 83 | Amitriptyline 10mg tablets | Tricyclic And Related Antidepressant Drugs/Neuropathic Pain/Prophylaxis Of Migraine |
| 84 | Dosulepin 25mg capsules | Tricyclic And Related Antidepressant Drugs |
| 114 | Lofepramine 70mg tablets | Tricyclic And Related Antidepressant Drugs |
| 182 | Tryptizol 10mg/ml Injection (Merck Sharp & Dohme Ltd) | Tricyclic And Related Antidepressant Drugs |
| 252 | Prozac 20mg/5ml liquid (Eli Lilly and Company Ltd) | Selective Serotonin Re-uptake Inhibitors |
| 301 | Venlafaxine 37.5mg tablets | Other Antidepressant Drugs |
| 418 | Prozac 20mg capsules (Eli Lilly and Company Ltd) | Selective Serotonin Re-uptake Inhibitors |
| 470 | Venlafaxine 75mg modified-release capsules | Other Antidepressant Drugs |
| 476 | Citalopram 10mg tablets | Selective Serotonin Re-uptake Inhibitors |
| 488 | Sertraline 50mg tablets | Selective Serotonin Re-uptake Inhibitors |
| 513 | Citalopram 40mg/ml oral drops sugar free | Selective Serotonin Re-uptake Inhibitors |
| 527 | Paroxetine 10mg/5ml oral suspension sugar free | Selective Serotonin Re-uptake Inhibitors |
| 595 | Amitriptyline 25mg / Perphenazine 2mg tablets | Tricyclic And Related Antidepressant Drugs |
| 603 | Escitalopram 10mg tablets | Selective Serotonin Re-uptake Inhibitors |
| 623 | Efexor 37.5mg tablets (Wyeth Pharmaceuticals) | Other Antidepressant Drugs |
| 648 | Cipralex 10mg tablets (Lundbeck Ltd) | Selective Serotonin Re-uptake Inhibitors |
| 727 | Sertraline 100mg tablets | Selective Serotonin Re-uptake Inhibitors |
| 742 | Mirtazapine 30mg tablets | Other Antidepressant Drugs |
| 785 | Cipralex 5mg tablets (Lundbeck Ltd) | Selective Serotonin Re-uptake Inhibitors |
| 815 | Cipramil 40mg/ml drops (Lundbeck Ltd) | Selective Serotonin Re-uptake Inhibitors |
| 841 | Seroxat 20mg tablets (GlaxoSmithKline UK Ltd) | Selective Serotonin Re-uptake Inhibitors |
| 1169 | Prothiaden 25mg capsules (Teofarma) | Tricyclic And Related Antidepressant Drugs |
| 1208 | Triptafen tablets (AMCo) | Tricyclic And Related Antidepressant Drugs |
| 1222 | Venlafaxine 75mg tablets | Other Antidepressant Drugs |
| 1310 | Imipramine 10mg tablets | Tricyclic And Related Antidepressant Drugs/Nocturnal Enuresis |
| 1397 | Paroxetine 30mg tablets | Selective Serotonin Re-uptake Inhibitors |
| 1453 | Triptafen m 2mg+10mg Tablet (Goldshield Pharmaceuticals Ltd) | Antipsychotic Drugs/Tricyclic And Related Antidepressant Drugs |
| 1474 | Efexor XL 75mg capsules (Pfizer Ltd) | Other Antidepressant Drugs |
| 1575 | Seroxat 30mg tablets (GlaxoSmithKline UK Ltd) | Selective Serotonin Re-uptake Inhibitors |
| 1612 | Lustral 50mg tablets (Pfizer Ltd) | Selective Serotonin Re-uptake Inhibitors |
| 1712 | Cipramil 20mg tablets (Lundbeck Ltd) | Selective Serotonin Re-uptake Inhibitors |
| 1730 | Trazodone 100mg capsules | Tricyclic And Related Antidepressant Drugs |
| 1809 | Imipramine 25mg tablets | Tricyclic And Related Antidepressant Drugs/Nocturnal Enuresis |
| 1888 | Amitriptyline 50mg tablets | Tricyclic And Related Antidepressant Drugs/Neuropathic Pain/Prophylaxis Of Migraine |
| 1940 | Dothapax 25 capsules (Ashbourne Pharmaceuticals Ltd) | Tricyclic And Related Antidepressant Drugs |
| 2039 | Trimipramine 25mg tablets | Tricyclic And Related Antidepressant Drugs |
| 2093 | Gamanil 70mg tablets (Merck Serono Ltd) | Tricyclic And Related Antidepressant Drugs |
| 2290 | Fluvoxamine 100mg tablets | Selective Serotonin Re-uptake Inhibitors |
| 2320 | Prothiaden 75mg tablets (Teofarma) | Tricyclic And Related Antidepressant Drugs |
| 2356 | Reboxetine 4mg tablets | Other Antidepressant Drugs |
| 2408 | Cipramil 40mg tablets (Lundbeck Ltd) | Selective Serotonin Re-uptake Inhibitors |
| 2525 | Amitriptyline 75mg modified-release capsules | Tricyclic And Related Antidepressant Drugs/Neuropathic Pain |
| 2531 | Surmontil 50mg capsules (Sanofi) | Tricyclic And Related Antidepressant Drugs |
| 2532 | Surmontil 25mg tablets (Sanofi) | Tricyclic And Related Antidepressant Drugs |
| 2548 | Fluoxetine 20mg/5ml oral solution | Selective Serotonin Re-uptake Inhibitors |
| 2579 | Tofranil 10mg Tablet (Novartis Pharmaceuticals UK Ltd) | Tricyclic And Related Antidepressant Drugs/Drugs For Urinary Frequency, Enuresis, And Incontinence |
| 2654 | Venlafaxine 150mg modified-release capsules | Other Antidepressant Drugs |
| 2880 | Fluvoxamine 50mg tablets | Selective Serotonin Re-uptake Inhibitors |
| 2897 | Faverin 50mg tablets (Mylan) | Selective Serotonin Re-uptake Inhibitors |
| 3083 | Mianserin 10mg tablets | Tricyclic And Related Antidepressant Drugs |
| 3183 | Nortriptyline 10mg tablets | Tricyclic And Related Antidepressant Drugs/Nocturnal Enuresis |
| 3194 | Clomipramine 10mg capsules | Tricyclic And Related Antidepressant Drugs |
| 3196 | Trimipramine 50mg capsules | Tricyclic And Related Antidepressant Drugs |
| 3355 | Trazodone 50mg capsules | Tricyclic And Related Antidepressant Drugs |
| 3391 | Dutonin 100mg tablets (Bristol-Myers Squibb Pharmaceuticals Ltd) | Selective Serotonin Re-uptake Inhibitors |
| 3554 | Doxepin 25mg capsules | Tricyclic And Related Antidepressant Drugs |
| 3601 | Seroxat 20mg/10ml liquid (GlaxoSmithKline UK Ltd) | Selective Serotonin Re-uptake Inhibitors |
| 3652 | Amoxapine 100mg tablets | Tricyclic And Related Antidepressant Drugs |
| 3657 | Anafranil 25mg capsules (Novartis Pharmaceuticals UK Ltd) | Tricyclic And Related Antidepressant Drugs |
| 3670 | Clomipramine 25mg capsules | Tricyclic And Related Antidepressant Drugs |
| 3777 | Amitriptyline 10mg/5ml sugar free oral solution | Tricyclic And Related Antidepressant Drugs/Neuropathic Pain |
| 3842 | Doxepin 10mg capsules | Tricyclic And Related Antidepressant Drugs |
| 3861 | Cipramil 10mg tablets (Lundbeck Ltd) | Selective Serotonin Re-uptake Inhibitors |
| 3903 | Nortriptyline 25mg tablets | Tricyclic And Related Antidepressant Drugs/Nocturnal Enuresis |
| 3925 | Clomipramine 50mg capsules | Tricyclic And Related Antidepressant Drugs |
| 4003 | Molipaxin 150mg tablets (Zentiva) | Tricyclic And Related Antidepressant Drugs |
| 4011 | Nefazodone 200mg tablets | Selective Serotonin Re-uptake Inhibitors |
| 4020 | Trazodone 150mg tablets | Tricyclic And Related Antidepressant Drugs |
| 4075 | Fluoxetine 60mg capsules | Selective Serotonin Re-uptake Inhibitors |
| 4118 | Nortriptyline 10mg Capsule | Tricyclic And Related Antidepressant Drugs/Neuropathic Pain |
| 4194 | Molipaxin 100mg capsules (Zentiva) | Tricyclic And Related Antidepressant Drugs |
| 4218 | Lofepramine 70mg/5ml oral suspension sugar free | Tricyclic And Related Antidepressant Drugs |
| 4297 | Dutonin 200mg tablets (Bristol-Myers Squibb Pharmaceuticals Ltd) | Selective Serotonin Re-uptake Inhibitors |
| 4310 | Trimipramine 10mg tablets | Tricyclic And Related Antidepressant Drugs |
| 4352 | Lustral 100mg tablets (Pfizer Ltd) | Selective Serotonin Re-uptake Inhibitors |
| 4411 | Amoxapine 150mg tablets | Tricyclic And Related Antidepressant Drugs |
| 4554 | Nefazodone 100mg tablets | Selective Serotonin Re-uptake Inhibitors |
| 4690 | Amitriptyline 50mg/5ml oral solution sugar free | Tricyclic And Related Antidepressant Drugs/Neuropathic Pain/Prophylaxis Of Migraine |
| 4726 | Zispin 30mg tablets (Organon Laboratories Ltd) | Other Antidepressant Drugs |
| 4770 | Citalopram 40mg tablets | Selective Serotonin Re-uptake Inhibitors |
| 4874 | Molipaxin 50mg capsules (Zentiva) | Tricyclic And Related Antidepressant Drugs |
| 4907 | Prozac 60mg capsules (Eli Lilly and Company Ltd) | Selective Serotonin Re-uptake Inhibitors |
| 5073 | Doxepin 50mg capsules | Tricyclic And Related Antidepressant Drugs |
| 5710 | Efexor XL 150mg capsules (Pfizer Ltd) | Other Antidepressant Drugs |
| 6054 | Dosulepin 25mg/5ml oral solution sugar free | Tricyclic And Related Antidepressant Drugs |
| 6218 | Escitalopram 20mg tablets | Selective Serotonin Re-uptake Inhibitors |
| 6255 | Mianserin 30mg tablets | Tricyclic And Related Antidepressant Drugs |
| 6312 | Amitriptyline 25mg/5ml oral solution sugar free | Tricyclic And Related Antidepressant Drugs/Neuropathic Pain/Prophylaxis Of Migraine |
| 6360 | Cipralex 20mg tablets (Lundbeck Ltd) | Selective Serotonin Re-uptake Inhibitors |
| 6405 | Escitalopram 5mg tablets | Selective Serotonin Re-uptake Inhibitors |
| 6421 | Mirtazapine 15mg orodispersible tablets | Other Antidepressant Drugs |
| 6442 | Trazodone 50mg/5ml oral solution sugar free | Tricyclic And Related Antidepressant Drugs |
| 6481 | Mirtazapine 45mg orodispersible tablets | Other Antidepressant Drugs |
| 6488 | Mirtazapine 30mg orodispersible tablets | Other Antidepressant Drugs |
| 6795 | Mirtazapine 15mg tablets | Other Antidepressant Drugs |
| 6846 | Zispin SolTab 15mg orodispersible tablets (Merck Sharp & Dohme Ltd) | Other Antidepressant Drugs |
| 6854 | Mirtazapine 45mg tablets | Other Antidepressant Drugs |
| 6894 | Perphenazine 2mg with Amitriptyline 25mg tablet | Antipsychotic Drugs/Tricyclic And Related Antidepressant Drugs |
| 6895 | Duloxetine 60mg gastro-resistant capsules | Other Antidepressant Drugs/Treatment Of Diabetic Nephropathy And Neuropathy |
| 7059 | Doxepin 75mg capsules | Tricyclic And Related Antidepressant Drugs |
| 7122 | Duloxetine 30mg gastro-resistant capsules | Other Antidepressant Drugs/Treatment Of Diabetic Nephropathy And Neuropathy |
| 7328 | Sertraline 50mg/5ml oral suspension | Selective Serotonin Re-uptake Inhibitors |
| 7468 | Bolvidon 10mg Tablet (Organon Laboratories Ltd) | Tricyclic And Related Antidepressant Drugs |
| 7515 | Anafranil 10mg capsules (Novartis Pharmaceuticals UK Ltd) | Tricyclic And Related Antidepressant Drugs |
| 7677 | Allegron 10mg tablets (King Pharmaceuticals Ltd) | Tricyclic And Related Antidepressant Drugs |
| 7678 | Nortriptyline 25mg Capsule | Tricyclic And Related Antidepressant Drugs/Neuropathic Pain |
| 7693 | Anafranil 50mg capsules (Novartis Pharmaceuticals UK Ltd) | Tricyclic And Related Antidepressant Drugs |
| 7751 | Tryptizol 25mg Tablet (Merck Sharp & Dohme Ltd) | Tricyclic And Related Antidepressant Drugs/Neuropathic Pain |
| 7755 | Concordin 10 Tablet (Merck Sharp & Dohme Ltd) | Tricyclic And Related Antidepressant Drugs/Neuropathic Pain |
| 7756 | Protriptyline 5mg tablet | Tricyclic And Related Antidepressant Drugs/Neuropathic Pain |
| 7816 | Concordin 5 Tablet (Merck Sharp & Dohme Ltd) | Tricyclic And Related Antidepressant Drugs/Neuropathic Pain |
| 7894 | Anafranil SR 75mg tablets (Novartis Pharmaceuticals UK Ltd) | Tricyclic And Related Antidepressant Drugs |
| 7910 | Tofranil 25mg tablets (Novartis Pharmaceuticals UK Ltd) | Tricyclic And Related Antidepressant Drugs |
| 7979 | Pertofran 25mg Tablet (Novartis Pharmaceuticals UK Ltd) | Tricyclic And Related Antidepressant Drugs/Neuropathic Pain |
| 7981 | Desipramine 25mg tablets | Tricyclic And Related Antidepressant Drugs/Neuropathic Pain |
| 8144 | Bolvidon 20mg Tablet (Organon Laboratories Ltd) | Tricyclic And Related Antidepressant Drugs |
| 8174 | Molipaxin 50mg/5ml oral liquid (Sanofi) | Tricyclic And Related Antidepressant Drugs |
| 8332 | Tryptizol 50mg Tablet (Merck Sharp & Dohme Ltd) | Tricyclic And Related Antidepressant Drugs/Neuropathic Pain |
| 8585 | Bolvidon 30mg Tablet (Organon Laboratories Ltd) | Tricyclic And Related Antidepressant Drugs |
| 8640 | Allegron 25mg tablets (King Pharmaceuticals Ltd) | Tricyclic And Related Antidepressant Drugs |
| 8661 | Clomipramine 75mg modified-release tablets | Tricyclic And Related Antidepressant Drugs |
| 8719 | Anafranil 25mg/5ml syrup (Novartis Pharmaceuticals UK Ltd) | Tricyclic And Related Antidepressant Drugs |
| 8720 | Clomipramine 25mg/5ml oral solution | Tricyclic And Related Antidepressant Drugs |
| 8726 | Tryptizol 10mg Tablet (Merck Sharp & Dohme Ltd) | Tricyclic And Related Antidepressant Drugs/Neuropathic Pain |
| 8831 | Tryptizol mr 75mg Modified-release capsule (Merck Sharp & Dohme Ltd) | Tricyclic And Related Antidepressant Drugs/Neuropathic Pain |
| 8878 | Tryptizol 10mg/5ml sugar free Oral solution (Merck Sharp and Dohme Ltd) | Tricyclic And Related Antidepressant Drugs/Neuropathic Pain |
| 8928 | Surmontil 10mg tablets (Sanofi) | Tricyclic And Related Antidepressant Drugs |
| 9182 | Efexor 75mg tablets (Wyeth Pharmaceuticals) | Other Antidepressant Drugs |
| 9534 | Nefazodone Starter pack | Other Antidepressant Drugs |
| 10083 | Zispin SolTab 30mg orodispersible tablets (Merck Sharp & Dohme Ltd) | Other Antidepressant Drugs |
| 10413 | Sinequan 10mg capsules (Pfizer Ltd) | Tricyclic And Related Antidepressant Drugs |
| 10948 | Dosulepin 75mg/5ml oral solution sugar free | Tricyclic And Related Antidepressant Drugs |
| 11187 | Protriptyline 10mg tablet | Tricyclic And Related Antidepressant Drugs/Neuropathic Pain |
| 11956 | Norval 20mg Tablet (Bencard) | Tricyclic And Related Antidepressant Drugs |
| 11963 | Limbitrol 10 Capsule (Roche Products Ltd) | Tricyclic And Related Antidepressant Drugs |
| 12111 | Vivalan 50mg Tablet (AstraZeneca UK Ltd) | Tricyclic And Related Antidepressant Drugs |
| 12123 | Faverin 100mg tablets (Mylan) | Selective Serotonin Re-uptake Inhibitors |
| 12125 | Sinequan 50mg capsules (Pfizer Ltd) | Tricyclic And Related Antidepressant Drugs |
| 12129 | Sinequan 25mg capsules (Pfizer Ltd) | Tricyclic And Related Antidepressant Drugs |
| 12192 | Norval 30mg Tablet (Bencard) | Tricyclic And Related Antidepressant Drugs |
| 12227 | Butriptyline 25mg tablets | Tricyclic And Related Antidepressant Drugs |
| 12309 | Viloxazine hcl 50mg tablets | Tricyclic And Related Antidepressant Drugs |
| 12353 | Aventyl 25mg Capsule (Eli Lilly and Company Ltd) | Tricyclic And Related Antidepressant Drugs/Neuropathic Pain |
| 12368 | Norval 10mg Tablet (Bencard) | Tricyclic And Related Antidepressant Drugs |
| 12549 | Aventyl 10mg/5ml Liquid (Eli Lilly and Company Ltd) | Tricyclic And Related Antidepressant Drugs/Neuropathic Pain |
| 13151 | Cymbalta 30mg gastro-resistant capsules (Eli Lilly and Company Ltd) | Other Antidepressant Drugs/Treatment Of Diabetic Nephropathy And Neuropathy |
| 13237 | Venlafaxine 37.5mg/5ml oral suspension | Other Antidepressant Drugs |
| 14398 | Asendis 50mg Tablet (Wyeth Pharmaceuticals) | Tricyclic And Related Antidepressant Drugs |
| 14519 | Sinequan 75mg capsules (Pfizer Ltd) | Tricyclic And Related Antidepressant Drugs |
| 14534 | Limbitrol 5 Capsule (Roche Products Ltd) | Tricyclic And Related Antidepressant Drugs |
| 14740 | Oxactin 20mg capsules (Discovery Pharmaceuticals) | Selective Serotonin Re-uptake Inhibitors |
| 14849 | Cymbalta 60mg gastro-resistant capsules (Eli Lilly and Company Ltd) | Other Antidepressant Drugs/Treatment Of Diabetic Nephropathy And Neuropathy |
| 15163 | Edronax 4mg tablets (Pfizer Ltd) | Other Antidepressant Drugs |
| 15268 | Zispin SolTab 45mg orodispersible tablets (Merck Sharp & Dohme Ltd) | Other Antidepressant Drugs |
| 15380 | Asendis 25mg Tablet (Wyeth Pharmaceuticals) | Tricyclic And Related Antidepressant Drugs |
| 15632 | Dothapax 75 tablets (Ashbourne Pharmaceuticals Ltd) | Tricyclic And Related Antidepressant Drugs |
| 16154 | Mirtazapine 15mg/ml oral solution sugar free | Other Antidepressant Drugs |
| 16323 | Perphenazine 2mg with Amitriptyline 10mg tablet | Antipsychotic Drugs/Tricyclic And Related Antidepressant Drugs |
| 17183 | Aventyl 10mg Capsule (Eli Lilly and Company Ltd) | Tricyclic And Related Antidepressant Drugs/Neuropathic Pain |
| 17319 | Amoxapine 25mg tablets | Tricyclic And Related Antidepressant Drugs |
| 18342 | Amitriptyline 25mg / Chlordiazepoxide 10mg capsules | Tricyclic And Related Antidepressant Drugs |
| 18932 | Evadyne 25mg Tablet (Wyeth Pharmaceuticals) | Tricyclic And Related Antidepressant Drugs |
| 19168 | Dosulepin 25mg/5ml mixture | Tricyclic And Related Antidepressant Drugs |
| 19181 | Trazodone 100mg capsules (Mylan) | Tricyclic And Related Antidepressant Drugs |
| 19183 | Fluoxetine 20mg capsules (A A H Pharmaceuticals Ltd) | Selective Serotonin Re-uptake Inhibitors |
| 19186 | Dosulepin 75mg tablets (Actavis UK Ltd) | Tricyclic And Related Antidepressant Drugs |
| 19470 | Fluoxetine 20mg capsules (Ranbaxy (UK) Ltd) | Selective Serotonin Re-uptake Inhibitors |
| 19779 | Amitriptyline 10mg/ml injection | Tricyclic And Related Antidepressant Drugs |
| 20026 | Domical 25mg Tablet (Berk Pharmaceuticals Ltd) | Tricyclic And Related Antidepressant Drugs/Neuropathic Pain |
| 20152 | Escitalopram 10mg/ml oral drops sugar free | Selective Serotonin Re-uptake Inhibitors |
| 20571 | Fluphenazine with nortriptyline 500microgramswith10mg Tablet | Tricyclic And Related Antidepressant Drugs |
| 21081 | Amitriptyline 12.5mg / Chlordiazepoxide 5mg capsules | Tricyclic And Related Antidepressant Drugs |
| 21157 | Thaden 75mg tablets (Opus Pharmaceuticals Ltd) | Tricyclic And Related Antidepressant Drugs |
| 21357 | Asendis 100mg Tablet (Wyeth Pharmaceuticals) | Tricyclic And Related Antidepressant Drugs |
| 21819 | Prepadine 75mg tablets (Teva UK Ltd) | Tricyclic And Related Antidepressant Drugs |
| 21820 | Prepadine 25mg capsules (Teva UK Ltd) | Tricyclic And Related Antidepressant Drugs |
| 22070 | Amitriptyline 10mg/5ml Oral solution (Rosemont Pharmaceuticals Ltd) | Tricyclic And Related Antidepressant Drugs/Neuropathic Pain |
| 23426 | Dosulepin 25mg capsules (A A H Pharmaceuticals Ltd) | Tricyclic And Related Antidepressant Drugs |
| 24134 | Amitriptyline 25mg tablets (Kent Pharmaceuticals Ltd) | Tricyclic And Related Antidepressant Drugs/Neuropathic Pain/Prophylaxis Of Migraine |
| 24141 | Amitriptyline 10mg tablets (Actavis UK Ltd) | Tricyclic And Related Antidepressant Drugs/Neuropathic Pain/Prophylaxis Of Migraine |
| 24145 | Amitriptyline 25mg tablets (Actavis UK Ltd) | Tricyclic And Related Antidepressant Drugs/Neuropathic Pain/Prophylaxis Of Migraine |
| 24147 | Amitriptyline 25mg tablets (Teva UK Ltd) | Tricyclic And Related Antidepressant Drugs/Neuropathic Pain/Prophylaxis Of Migraine |
| 24152 | Amitriptyline 10mg tablets (Teva UK Ltd) | Tricyclic And Related Antidepressant Drugs/Neuropathic Pain/Prophylaxis Of Migraine |
| 24680 | Elavil 10mg Tablet (DDSA Pharmaceuticals Ltd) | Tricyclic And Related Antidepressant Drugs/Neuropathic Pain |
| 24700 | Prondol 15mg Tablet (Wyeth Pharmaceuticals) | Tricyclic And Related Antidepressant Drugs |
| 24723 | Asendis 150mg Tablet (Wyeth Pharmaceuticals) | Tricyclic And Related Antidepressant Drugs |
| 25444 | Lomont 70mg/5ml oral suspension (Rosemont Pharmaceuticals Ltd) | Tricyclic And Related Antidepressant Drugs |
| 26016 | Citalopram 20mg tablets (Sandoz Ltd) | Selective Serotonin Re-uptake Inhibitors |
| 26056 | Cipralex 10mg/ml oral drops (Lundbeck Ltd) | Selective Serotonin Re-uptake Inhibitors |
| 26213 | Domical 10mg Tablet (Berk Pharmaceuticals Ltd) | Tricyclic And Related Antidepressant Drugs/Neuropathic Pain |
| 27008 | Domical 50mg Tablet (Berk Pharmaceuticals Ltd) | Tricyclic And Related Antidepressant Drugs/Neuropathic Pain |
| 27476 | Iprindole hc 15mg | Tricyclic And Related Antidepressant Drugs |
| 27733 | Iprindole hc 30mg | Tricyclic And Related Antidepressant Drugs |
| 29339 | Trazodone 50mg capsules (Mylan) | Tricyclic And Related Antidepressant Drugs |
| 29756 | Paxoran 20mg Tablet (Ranbaxy (UK) Ltd) | Selective Serotonin Re-uptake Inhibitors |
| 29786 | Ranflutin 20mg capsules (Ranbaxy (UK) Ltd) | Selective Serotonin Re-uptake Inhibitors |
| 29857 | Trazodone 150mg tablets (Teva UK Ltd) | Tricyclic And Related Antidepressant Drugs |
| 29875 | Dosulepin 25mg capsules (Mylan) | Tricyclic And Related Antidepressant Drugs |
| 30258 | Fluoxetine 20mg/5ml oral solution (Teva UK Ltd) | Selective Serotonin Re-uptake Inhibitors |
| 30376 | Thaden 25mg capsules (Opus Pharmaceuticals Ltd) | Tricyclic And Related Antidepressant Drugs |
| 30983 | Trazodone 150mg tablets (Mylan) | Tricyclic And Related Antidepressant Drugs |
| 31672 | Prondol 30mg Tablet (Wyeth Pharmaceuticals) | Tricyclic And Related Antidepressant Drugs |
| 31824 | Dosulepin 25mg capsules (IVAX Pharmaceuticals UK Ltd) | Tricyclic And Related Antidepressant Drugs |
| 31826 | Dosulepin 75mg tablets (IVAX Pharmaceuticals UK Ltd) | Tricyclic And Related Antidepressant Drugs |
| 32121 | Dosulepin 75mg tablets (A A H Pharmaceuticals Ltd) | Tricyclic And Related Antidepressant Drugs |
| 32401 | Sertraline 50mg tablets (A A H Pharmaceuticals Ltd) | Selective Serotonin Re-uptake Inhibitors |
| 32439 | Amitriptyline 25mg Tablet (Sussex Pharmaceutical Ltd) | Tricyclic And Related Antidepressant Drugs/Neuropathic Pain |
| 32457 | Butriptyline 50mg tablets | Tricyclic And Related Antidepressant Drugs |
| 32546 | Paxoran 10mg Tablet (Ranbaxy (UK) Ltd) | Selective Serotonin Re-uptake Inhibitors |
| 32848 | Citalopram 10mg tablets (Actavis UK Ltd) | Selective Serotonin Re-uptake Inhibitors |
| 32863 | Imipramine 10mg tablets (Teva UK Ltd) | Tricyclic And Related Antidepressant Drugs/Nocturnal Enuresis |
| 32899 | Paroxetine 20mg tablets (Actavis UK Ltd) | Selective Serotonin Re-uptake Inhibitors |
| 33071 | Felicium 20mg capsules (Opus Pharmaceuticals Ltd) | Selective Serotonin Re-uptake Inhibitors |
| 33074 | Praminil 10mg Tablet (DDSA Pharmaceuticals Ltd) | Tricyclic And Related Antidepressant Drugs/Drugs For Urinary Frequency, Enuresis, And Incontinence |
| 33090 | Amitriptyline 10mg tablets (A A H Pharmaceuticals Ltd) | Tricyclic And Related Antidepressant Drugs/Neuropathic Pain/Prophylaxis Of Migraine |
| 33164 | Dosulepin 25mg capsules (Sandoz Ltd) | Tricyclic And Related Antidepressant Drugs |
| 33337 | Mirtazapine 45mg tablets (A A H Pharmaceuticals Ltd) | Other Antidepressant Drugs |
| 33410 | Fluoxetine 20mg capsules (Zentiva) | Selective Serotonin Re-uptake Inhibitors |
| 33624 | Amitriptyline 50mg tablets (Teva UK Ltd) | Tricyclic And Related Antidepressant Drugs/Neuropathic Pain/Prophylaxis Of Migraine |
| 33720 | Citalopram 10mg tablets (IVAX Pharmaceuticals UK Ltd) | Selective Serotonin Re-uptake Inhibitors |
| 33779 | Prozit 20mg/5ml oral solution (Pinewood Healthcare) | Selective Serotonin Re-uptake Inhibitors |
| 33978 | Paroxetine 20mg tablets (Mylan) | Selective Serotonin Re-uptake Inhibitors |
| 34003 | Trazodone 50mg capsules (A A H Pharmaceuticals Ltd) | Tricyclic And Related Antidepressant Drugs |
| 34046 | Lofepramine 70mg tablets (A A H Pharmaceuticals Ltd) | Tricyclic And Related Antidepressant Drugs |
| 34058 | Dosulepin 75mg tablets (Teva UK Ltd) | Tricyclic And Related Antidepressant Drugs |
| 34107 | Amitriptyline 50mg tablets (Wockhardt UK Ltd) | Tricyclic And Related Antidepressant Drugs/Neuropathic Pain/Prophylaxis Of Migraine |
| 34129 | Amitriptyline 25mg tablets (Wockhardt UK Ltd) | Tricyclic And Related Antidepressant Drugs/Neuropathic Pain/Prophylaxis Of Migraine |
| 34182 | Amitriptyline 50mg tablets (Kent Pharmaceuticals Ltd) | Tricyclic And Related Antidepressant Drugs/Neuropathic Pain/Prophylaxis Of Migraine |
| 34197 | Amitriptyline 25mg Tablet (Berk Pharmaceuticals Ltd) | Tricyclic And Related Antidepressant Drugs/Neuropathic Pain |
| 34202 | Fluoxetine 20mg capsules (Genus Pharmaceuticals Ltd) | Selective Serotonin Re-uptake Inhibitors |
| 34216 | Fluoxetine 20mg/5ml oral solution (A A H Pharmaceuticals Ltd) | Selective Serotonin Re-uptake Inhibitors |
| 34222 | Imipramine 10mg tablets (Actavis UK Ltd) | Tricyclic And Related Antidepressant Drugs/Nocturnal Enuresis |
| 34223 | Dosulepin 25mg capsules (Teva UK Ltd) | Tricyclic And Related Antidepressant Drugs |
| 34224 | Amitriptyline 25mg/5ml oral solution sugar free (Rosemont Pharmaceuticals Ltd) | Tricyclic And Related Antidepressant Drugs/Neuropathic Pain/Prophylaxis Of Migraine |
| 34245 | Clomipramine 25mg capsules (A A H Pharmaceuticals Ltd) | Tricyclic And Related Antidepressant Drugs |
| 34251 | Amitriptyline 50mg/5ml oral solution sugar free (Rosemont Pharmaceuticals Ltd) | Tricyclic And Related Antidepressant Drugs/Neuropathic Pain/Prophylaxis Of Migraine |
| 34274 | Amitriptyline 50mg tablets (A A H Pharmaceuticals Ltd) | Tricyclic And Related Antidepressant Drugs/Neuropathic Pain/Prophylaxis Of Migraine |
| 34288 | Fluoxetine 20mg capsules (Mylan) | Selective Serotonin Re-uptake Inhibitors |
| 34294 | Fluoxetine 20mg capsules (IVAX Pharmaceuticals UK Ltd) | Selective Serotonin Re-uptake Inhibitors |
| 34351 | Paroxetine 20mg tablets (IVAX Pharmaceuticals UK Ltd) | Selective Serotonin Re-uptake Inhibitors |
| 34355 | Imipramine 25mg tablets (Actavis UK Ltd) | Tricyclic And Related Antidepressant Drugs/Nocturnal Enuresis |
| 34356 | Citalopram 20mg tablets (A A H Pharmaceuticals Ltd) | Selective Serotonin Re-uptake Inhibitors |
| 34401 | Amitriptyline 10mg tablets (Wockhardt UK Ltd) | Tricyclic And Related Antidepressant Drugs/Neuropathic Pain/Prophylaxis Of Migraine |
| 34413 | Citalopram 10mg tablets (Zentiva) | Selective Serotonin Re-uptake Inhibitors |
| 34415 | Citalopram 20mg tablets (Mylan) | Selective Serotonin Re-uptake Inhibitors |
| 34419 | Paroxetine 20mg tablets (A A H Pharmaceuticals Ltd) | Selective Serotonin Re-uptake Inhibitors |
| 34421 | Trazodone 50mg capsules (Zentiva) | Tricyclic And Related Antidepressant Drugs |
| 34436 | Citalopram 10mg tablets (Mylan) | Selective Serotonin Re-uptake Inhibitors |
| 34456 | Fluoxetine 20mg capsules (Teva UK Ltd) | Selective Serotonin Re-uptake Inhibitors |
| 34466 | Citalopram 40mg tablets (Sandoz Ltd) | Selective Serotonin Re-uptake Inhibitors |
| 34470 | Trazodone 150mg tablets (Zentiva) | Tricyclic And Related Antidepressant Drugs |
| 34474 | Amitriptyline 25mg Tablet (Regent Laboratories Ltd) | Tricyclic And Related Antidepressant Drugs/Neuropathic Pain |
| 34498 | Citalopram 10mg Tablet (Neo Laboratories Ltd) | Selective Serotonin Re-uptake Inhibitors |
| 34499 | Citalopram 10mg tablets (Sandoz Ltd) | Selective Serotonin Re-uptake Inhibitors |
| 34503 | Amitriptyline 25mg tablets (IVAX Pharmaceuticals UK Ltd) | Tricyclic And Related Antidepressant Drugs |
| 34525 | Dosulepin 75mg tablets (Mylan) | Tricyclic And Related Antidepressant Drugs |
| 34578 | Lofepramine 70mg tablets (IVAX Pharmaceuticals UK Ltd) | Tricyclic And Related Antidepressant Drugs |
| 34580 | Trazodone 100mg capsules (A A H Pharmaceuticals Ltd) | Tricyclic And Related Antidepressant Drugs |
| 34586 | Citalopram 10mg tablets (A A H Pharmaceuticals Ltd) | Selective Serotonin Re-uptake Inhibitors |
| 34587 | Paroxetine 30mg tablets (A A H Pharmaceuticals Ltd) | Selective Serotonin Re-uptake Inhibitors |
| 34603 | Citalopram 40mg tablets (Mylan) | Selective Serotonin Re-uptake Inhibitors |
| 34634 | Amitriptyline 50mg tablets (Actavis UK Ltd) | Tricyclic And Related Antidepressant Drugs/Neuropathic Pain/Prophylaxis Of Migraine |
| 34641 | Dosulepin 25mg capsules (Sovereign Medical Ltd) | Tricyclic And Related Antidepressant Drugs |
| 34643 | Dosulepin 25mg capsules (Almus Pharmaceuticals Ltd) | Tricyclic And Related Antidepressant Drugs |
| 34672 | Lofepramine 70mg tablets (Sterwin Medicines) | Tricyclic And Related Antidepressant Drugs |
| 34722 | Citalopram 20mg Tablet (Neo Laboratories Ltd) | Selective Serotonin Re-uptake Inhibitors |
| 34731 | Amitriptyline 10mg tablets (Kent Pharmaceuticals Ltd) | Tricyclic And Related Antidepressant Drugs/Neuropathic Pain/Prophylaxis Of Migraine |
| 34745 | Dosulepin 25mg capsules (Actavis UK Ltd) | Tricyclic And Related Antidepressant Drugs |
| 34782 | Amitriptyline 25mg tablets (A A H Pharmaceuticals Ltd) | Tricyclic And Related Antidepressant Drugs/Neuropathic Pain/Prophylaxis Of Migraine |
| 34813 | Imipramine 25mg tablets (A A H Pharmaceuticals Ltd) | Tricyclic And Related Antidepressant Drugs/Nocturnal Enuresis |
| 34822 | Citalopram 20mg tablets (Zentiva) | Selective Serotonin Re-uptake Inhibitors |
| 34849 | Fluoxetine 20mg capsules (Tillomed Laboratories Ltd) | Selective Serotonin Re-uptake Inhibitors |
| 34856 | Fluoxetine 60mg capsules (Mylan) | Selective Serotonin Re-uptake Inhibitors |
| 34866 | Clomipramine 10mg capsules (A A H Pharmaceuticals Ltd) | Tricyclic And Related Antidepressant Drugs |
| 34871 | Citalopram 20mg tablets (Actavis UK Ltd) | Selective Serotonin Re-uptake Inhibitors |
| 34872 | Imipramine 25mg Tablet (C P Pharmaceuticals Ltd) | Tricyclic And Related Antidepressant Drugs/Drugs For Urinary Frequency, Enuresis, And Incontinence |
| 34916 | Amitriptyline 10mg Tablet (Berk Pharmaceuticals Ltd) | Tricyclic And Related Antidepressant Drugs/Neuropathic Pain |
| 34950 | Lofepramine 70mg tablets (Actavis UK Ltd) | Tricyclic And Related Antidepressant Drugs |
| 34966 | Citalopram 20mg tablets (Teva UK Ltd) | Selective Serotonin Re-uptake Inhibitors |
| 34970 | Citalopram 20mg tablets (Niche Generics Ltd) | Selective Serotonin Re-uptake Inhibitors |
| 35021 | Paroxetine 10mg tablets | Selective Serotonin Re-uptake Inhibitors |
| 35112 | Seroxat 10mg tablets (GlaxoSmithKline UK Ltd) | Selective Serotonin Re-uptake Inhibitors |
| 35258 | Sinepin 25mg capsules (Marlborough Pharmaceuticals Ltd) | Tricyclic And Related Antidepressant Drugs |
| 35493 | Sinepin 50mg capsules (Marlborough Pharmaceuticals Ltd) | Tricyclic And Related Antidepressant Drugs |
| 36746 | Citalopram 40mg tablets (A A H Pharmaceuticals Ltd) | Selective Serotonin Re-uptake Inhibitors |
| 36893 | Fluoxetine 20mg/5ml oral solution sugar free | Selective Serotonin Re-uptake Inhibitors |
| 37256 | Prozep 20mg/5ml oral solution (Chemidex Pharma Ltd) | Selective Serotonin Re-uptake Inhibitors |
| 38274 | Clomipramine 50mg/5ml oral suspension | Tricyclic And Related Antidepressant Drugs |
| 38890 | Fluoxetine 20mg Capsule (Milpharm Ltd) | Selective Serotonin Re-uptake Inhibitors |
| 39145 | Nortriptyline 10mg/5ml Liquid | Tricyclic And Related Antidepressant Drugs/Neuropathic Pain |
| 39359 | Venlafaxine 75mg modified-release tablets | Other Antidepressant Drugs |
| 39360 | Venlafaxine 150mg modified-release tablets | Other Antidepressant Drugs |
| 39770 | Tifaxin XL 75mg capsules (Genus Pharmaceuticals Ltd) | Other Antidepressant Drugs |
| 39809 | Tifaxin XL 150mg capsules (Genus Pharmaceuticals Ltd) | Other Antidepressant Drugs |
| 40048 | ViePax XL 75mg tablets (Dexcel-Pharma Ltd) | Other Antidepressant Drugs |
| 40049 | ViePax XL 150mg tablets (Dexcel-Pharma Ltd) | Other Antidepressant Drugs |
| 40054 | Venlafaxine 225mg modified-release tablets | Other Antidepressant Drugs |
| 40059 | Venlalic XL 75mg tablets (Ethypharm UK Ltd) | Other Antidepressant Drugs |
| 40062 | Venlalic XL 150mg tablets (Ethypharm UK Ltd) | Other Antidepressant Drugs |
| 40092 | Vensir XL 150mg capsules (Morningside Healthcare Ltd) | Other Antidepressant Drugs |
| 40160 | Mirtazapine 30mg tablets (Actavis UK Ltd) | Other Antidepressant Drugs |
| 40165 | Paroxetine 30mg tablets (Actavis UK Ltd) | Selective Serotonin Re-uptake Inhibitors |
| 40277 | Vensir XL 75mg capsules (Morningside Healthcare Ltd) | Other Antidepressant Drugs |
| 40295 | Valdoxan 25mg tablets (Servier Laboratories Ltd) | Other Antidepressant Drugs |
| 40396 | Amitriptyline 50mg Tablet (Berk Pharmaceuticals Ltd) | Tricyclic And Related Antidepressant Drugs/Neuropathic Pain |
| 40407 | Venlalic XL 225mg tablets (Ethypharm UK Ltd) | Other Antidepressant Drugs |
| 40494 | Agomelatine 25mg tablets | Other Antidepressant Drugs |
| 40514 | Venaxx XL 150mg capsules (AMCo) | Other Antidepressant Drugs |
| 40515 | Venaxx XL 75mg capsules (AMCo) | Other Antidepressant Drugs |
| 40517 | Vexarin XL 150mg capsules (Mylan) | Other Antidepressant Drugs |
| 40726 | Escitalopram 20mg/ml oral drops sugar free | Selective Serotonin Re-uptake Inhibitors |
| 40764 | ViePax 37.5mg tablets (Dexcel-Pharma Ltd) | Other Antidepressant Drugs |
| 40777 | Doxepin 25mg/5ml oral suspension | Tricyclic And Related Antidepressant Drugs |
| 40815 | Tardcaps XL 75mg capsules (IXL Pharma Ltd) | Other Antidepressant Drugs |
| 40817 | Tardcaps XL 150mg capsules (IXL Pharma Ltd) | Other Antidepressant Drugs |
| 40892 | Paroxetine 20mg tablets (Genus Pharmaceuticals Ltd) | Selective Serotonin Re-uptake Inhibitors |
| 40917 | ViePax 75mg tablets (Dexcel-Pharma Ltd) | Other Antidepressant Drugs |
| 41033 | Rodomel XL 75mg capsules (Teva UK Ltd) | Other Antidepressant Drugs |
| 41062 | Cipralex 20mg/ml oral drops (Lundbeck Ltd) | Selective Serotonin Re-uptake Inhibitors |
| 41299 | Politid XL 75mg capsules (Actavis UK Ltd) | Other Antidepressant Drugs |
| 41314 | Rodomel XL 150mg capsules (Teva UK Ltd) | Other Antidepressant Drugs |
| 41408 | Imipramine 25mg tablets (Teva UK Ltd) | Tricyclic And Related Antidepressant Drugs/Nocturnal Enuresis |
| 41528 | Citalopram 10mg tablets (Teva UK Ltd) | Selective Serotonin Re-uptake Inhibitors |
| 41563 | Clomipramine 25mg capsules (IVAX Pharmaceuticals UK Ltd) | Tricyclic And Related Antidepressant Drugs |
| 41597 | Clomipramine 50mg capsules (IVAX Pharmaceuticals UK Ltd) | Tricyclic And Related Antidepressant Drugs |
| 41609 | Trazodone 50mg capsules (Teva UK Ltd) | Tricyclic And Related Antidepressant Drugs |
| 41627 | Lofepramine 70mg Tablet (Teva UK Ltd) | Tricyclic And Related Antidepressant Drugs |
| 41628 | Clomipramine 10mg capsules (IVAX Pharmaceuticals UK Ltd) | Tricyclic And Related Antidepressant Drugs |
| 41681 | Imipramine 10mg tablets (A A H Pharmaceuticals Ltd) | Tricyclic And Related Antidepressant Drugs/Nocturnal Enuresis |
| 41709 | Trazodone 100mg capsules (Teva UK Ltd) | Tricyclic And Related Antidepressant Drugs |
| 41710 | Trazodone 100mg capsules (Zentiva) | Tricyclic And Related Antidepressant Drugs |
| 41729 | Amitriptyline 25mg Tablet (Celltech Pharma Europe Ltd) | Tricyclic And Related Antidepressant Drugs/Neuropathic Pain |
| 42078 | Amitriptyline 25mg tablets (Almus Pharmaceuticals Ltd) | Tricyclic And Related Antidepressant Drugs/Neuropathic Pain/Prophylaxis Of Migraine |
| 42107 | Fluoxetine 20mg capsules (Niche Generics Ltd) | Selective Serotonin Re-uptake Inhibitors |
| 42228 | Trimipramine 10mg tablets (A A H Pharmaceuticals Ltd) | Tricyclic And Related Antidepressant Drugs |
| 42247 | Imipramine 25mg/5ml oral solution sugar free | Tricyclic And Related Antidepressant Drugs/Nocturnal Enuresis |
| 42387 | Sertraline 50mg tablets (Actavis UK Ltd) | Selective Serotonin Re-uptake Inhibitors |
| 42394 | Amitriptyline 25mg Tablet (Crosspharma Ltd) | Tricyclic And Related Antidepressant Drugs/Neuropathic Pain |
| 42499 | Fluoxetine 10mg tablets | Selective Serotonin Re-uptake Inhibitors |
| 42600 | Vexarin XL 75mg capsules (Mylan) | Other Antidepressant Drugs |
| 42660 | Citalopram 10mg tablets (Almus Pharmaceuticals Ltd) | Selective Serotonin Re-uptake Inhibitors |
| 42734 | Dosulepin 75mg tablets (Almus Pharmaceuticals Ltd) | Tricyclic And Related Antidepressant Drugs |
| 42803 | Fluoxetine 20mg/5ml oral solution (IVAX Pharmaceuticals UK Ltd) | Selective Serotonin Re-uptake Inhibitors |
| 43024 | Dosulepin 100mg/5ml oral solution | Tricyclic And Related Antidepressant Drugs |
| 43203 | Venlafaxine 75mg modified-release capsules (Sandoz Ltd) | Other Antidepressant Drugs |
| 43234 | Mirtazapine 45mg orodispersible tablets (Teva UK Ltd) | Other Antidepressant Drugs |
| 43235 | Mirtazapine 45mg orodispersible tablets (A A H Pharmaceuticals Ltd) | Other Antidepressant Drugs |
| 43236 | Mirtazapine 45mg orodispersible tablets (Actavis UK Ltd) | Other Antidepressant Drugs |
| 43237 | Mirtazapine 15mg orodispersible tablets (Teva UK Ltd) | Other Antidepressant Drugs |
| 43239 | Mirtazapine 15mg tablets (A A H Pharmaceuticals Ltd) | Other Antidepressant Drugs |
| 43241 | Mirtazapine 15mg orodispersible tablets (Aurobindo Pharma Ltd) | Other Antidepressant Drugs |
| 43242 | Mirtazapine 15mg tablets (Genus Pharmaceuticals Ltd) | Other Antidepressant Drugs |
| 43246 | Mirtazapine 15mg orodispersible tablets (Genus Pharmaceuticals Ltd) | Other Antidepressant Drugs |
| 43247 | Mirtazapine 45mg orodispersible tablets (Genus Pharmaceuticals Ltd) | Other Antidepressant Drugs |
| 43248 | Mirtazapine 15mg orodispersible tablets (Focus Pharmaceuticals Ltd) | Other Antidepressant Drugs |
| 43250 | Mirtazapine 30mg orodispersible tablets (A A H Pharmaceuticals Ltd) | Other Antidepressant Drugs |
| 43253 | Mirtazapine 15mg orodispersible tablets (A A H Pharmaceuticals Ltd) | Other Antidepressant Drugs |
| 43256 | Mirtazapine 45mg orodispersible tablets (Focus Pharmaceuticals Ltd) | Other Antidepressant Drugs |
| 43257 | Mirtazapine 15mg tablets (Teva UK Ltd) | Other Antidepressant Drugs |
| 43334 | Venlafaxine 150mg modified-release capsules (Sandoz Ltd) | Other Antidepressant Drugs |
| 43518 | Fluvoxamine 100mg tablets (IVAX Pharmaceuticals UK Ltd) | Selective Serotonin Re-uptake Inhibitors |
| 43519 | Citalopram 40mg Tablet (Neo Laboratories Ltd) | Selective Serotonin Re-uptake Inhibitors |
| 43534 | Lofepramine 70mg/5ml Oral suspension (Rosemont Pharmaceuticals Ltd) | Tricyclic And Related Antidepressant Drugs |
| 43561 | Clomipramine 10mg capsules (Teva UK Ltd) | Tricyclic And Related Antidepressant Drugs |
| 43673 | Politid XL 150mg capsules (Actavis UK Ltd) | Other Antidepressant Drugs |
| 43968 | Foraven XL 75mg capsules (Forum Products Ltd) | Other Antidepressant Drugs |
| 44853 | Dosulepin 25mg capsules (Kent Pharmaceuticals Ltd) | Tricyclic And Related Antidepressant Drugs |
| 44861 | Fluvoxamine 100mg tablets (Actavis UK Ltd) | Selective Serotonin Re-uptake Inhibitors |
| 44936 | Venlaneo XL 150mg capsules (Kent Pharmaceuticals Ltd) | Other Antidepressant Drugs |
| 44937 | Venlaneo XL 75mg capsules (Kent Pharmaceuticals Ltd) | Other Antidepressant Drugs |
| 44944 | Sertraline 100mg tablets (Teva UK Ltd) | Selective Serotonin Re-uptake Inhibitors |
| 45223 | Citalopram 40mg tablets (Niche Generics Ltd) | Selective Serotonin Re-uptake Inhibitors |
| 45224 | Fluoxetine 20mg capsules (Sandoz Ltd) | Selective Serotonin Re-uptake Inhibitors |
| 45226 | Trimipramine 25mg tablets (A A H Pharmaceuticals Ltd) | Tricyclic And Related Antidepressant Drugs |
| 45233 | Amitriptyline 10mg tablets (IVAX Pharmaceuticals UK Ltd) | Tricyclic And Related Antidepressant Drugs |
| 45242 | Amitriptyline 10mg Tablet (Sussex Pharmaceutical Ltd) | Tricyclic And Related Antidepressant Drugs/Neuropathic Pain |
| 45247 | Fluoxetine 20mg capsules (Fannin UK Ltd) | Selective Serotonin Re-uptake Inhibitors |
| 45286 | Citalopram 10mg tablets (Niche Generics Ltd) | Selective Serotonin Re-uptake Inhibitors |
| 45304 | Citalopram 40mg tablets (Teva UK Ltd) | Selective Serotonin Re-uptake Inhibitors |
| 45316 | Fluoxetine 20mg capsules (Wockhardt UK Ltd) | Selective Serotonin Re-uptake Inhibitors |
| 45318 | Clomipramine 50mg capsules (A A H Pharmaceuticals Ltd) | Tricyclic And Related Antidepressant Drugs |
| 45329 | Fluoxetine 20mg capsules (Actavis UK Ltd) | Selective Serotonin Re-uptake Inhibitors |
| 45350 | Clomipramine 25mg capsules (Teva UK Ltd) | Tricyclic And Related Antidepressant Drugs |
| 45664 | Depefex XL 150mg capsules (Chiesi Ltd) | Other Antidepressant Drugs |
| 45737 | Dosulepin 25mg/5ml Oral solution (Rosemont Pharmaceuticals Ltd) | Tricyclic And Related Antidepressant Drugs |
| 45806 | Venlafaxine 37.5mg modified-release tablets | Other Antidepressant Drugs |
| 45818 | Venlalic XL 37.5mg tablets (Ethypharm UK Ltd) | Other Antidepressant Drugs |
| 45915 | Sertraline 50mg tablets (Almus Pharmaceuticals Ltd) | Selective Serotonin Re-uptake Inhibitors |
| 45959 | Depefex XL 75mg capsules (Chiesi Ltd) | Other Antidepressant Drugs |
| 46668 | Mirtazapine 15mg tablets (Arrow Generics Ltd) | Other Antidepressant Drugs |
| 46801 | Amitriptyline 10mg/5ml oral solution | Tricyclic And Related Antidepressant Drugs/Neuropathic Pain/Prophylaxis Of Migraine |
| 46818 | Amitriptyline 10mg/5ml oral suspension | Tricyclic And Related Antidepressant Drugs/Neuropathic Pain/Prophylaxis Of Migraine |
| 46926 | Citalopram 40mg tablets (Zentiva) | Selective Serotonin Re-uptake Inhibitors |
| 46970 | Amitriptyline 50mg tablets (IVAX Pharmaceuticals UK Ltd) | Tricyclic And Related Antidepressant Drugs |
| 46977 | Citalopram 40mg tablets (Actavis UK Ltd) | Selective Serotonin Re-uptake Inhibitors |
| 47363 | Mianserin 20mg Tablet (Berk Pharmaceuticals Ltd) | Tricyclic And Related Antidepressant Drugs |
| 47945 | Mirtazapine 30mg tablets (A A H Pharmaceuticals Ltd) | Other Antidepressant Drugs |
| 47966 | Mirtazapine 15mg/ml oral solution sugar free (Rosemont Pharmaceuticals Ltd) | Other Antidepressant Drugs |
| 48026 | Citalopram 20mg tablets (Almus Pharmaceuticals Ltd) | Selective Serotonin Re-uptake Inhibitors |
| 48045 | Fluvoxamine 100mg tablets (A A H Pharmaceuticals Ltd) | Selective Serotonin Re-uptake Inhibitors |
| 48065 | Amitriptyline oral solution | Tricyclic And Related Antidepressant Drugs/Neuropathic Pain |
| 48199 | Ranfaxine XL 75mg capsules (Ranbaxy (UK) Ltd) | Other Antidepressant Drugs |
| 48216 | Nortriptyline 25mg tablets (A A H Pharmaceuticals Ltd) | Tricyclic And Related Antidepressant Drugs/Nocturnal Enuresis |
| 48220 | Prozac 20mg capsules (Lexon (UK) Ltd) | Selective Serotonin Re-uptake Inhibitors |
| 48698 | Mirtazapine 15mg orodispersible tablets sugar free | Other Antidepressant Drugs |
| 49165 | Citalopram 10mg tablets (Alliance Healthcare (Distribution) Ltd) | Selective Serotonin Re-uptake Inhibitors |
| 49511 | Venlablue XL 75mg capsules (Bluefish Pharmaceuticals AB) | Other Antidepressant Drugs |
| 49519 | Sertraline 100mg/5ml oral suspension | Selective Serotonin Re-uptake Inhibitors |
| 49820 | Mirtazapine 45mg orodispersible tablets sugar free | Other Antidepressant Drugs |
| 50081 | Venlablue XL 150mg capsules (Bluefish Pharmaceuticals AB) | Other Antidepressant Drugs |
| 50722 | Dosulepin 25mg/5ml oral solution | Tricyclic And Related Antidepressant Drugs |
| 50892 | Zispin SolTab 15mg orodispersible tablets (Necessity Supplies Ltd) | Other Antidepressant Drugs |
| 50934 | Venlafaxine 150mg/5ml oral solution | Other Antidepressant Drugs |
| 51280 | Efexor XL 150mg capsules (Waymade Healthcare Plc) | Other Antidepressant Drugs |
| 51361 | Venlafaxine 37.5mg tablets (Ranbaxy (UK) Ltd) | Other Antidepressant Drugs |
| 51383 | Duloxetine 60mg gastro-resistant capsules (Sigma Pharmaceuticals Plc) | Other Antidepressant Drugs/Treatment Of Diabetic Nephropathy And Neuropathy |
| 51699 | Venlafaxine 37.5mg/5ml oral solution | Other Antidepressant Drugs |
| 51758 | Prothiaden 25mg capsules (Stephar (U.K.) Ltd) | Tricyclic And Related Antidepressant Drugs |
| 52074 | Alventa XL 75mg capsules (Consilient Health Ltd) | Other Antidepressant Drugs |
| 52100 | Citalopram 10mg tablets (Arrow Generics Ltd) | Selective Serotonin Re-uptake Inhibitors |
| 52354 | Citalopram 20mg tablets (DE Pharmaceuticals) | Selective Serotonin Re-uptake Inhibitors |
| 52408 | Citalopram 10mg tablets (Kent Pharmaceuticals Ltd) | Selective Serotonin Re-uptake Inhibitors |
| 52516 | Alventa XL 150mg capsules (Consilient Health Ltd) | Other Antidepressant Drugs |
| 52607 | Citalopram 20mg tablets (Bristol Laboratories Ltd) | Selective Serotonin Re-uptake Inhibitors |
| 52716 | Tonpular XL 75mg capsules (Wockhardt UK Ltd) | Other Antidepressant Drugs |
| 52824 | Citalopram 10mg tablets (PLIVA Pharma Ltd) | Selective Serotonin Re-uptake Inhibitors |
| 52867 | Amitriptyline 10mg tablets (Accord Healthcare Ltd) | Tricyclic And Related Antidepressant Drugs/Neuropathic Pain/Prophylaxis Of Migraine |
| 53161 | Clomipramine 50mg/5ml oral solution | Tricyclic And Related Antidepressant Drugs |
| 53187 | Clomipramine 50mg capsules (Kent Pharmaceuticals Ltd) | Tricyclic And Related Antidepressant Drugs |
| 53321 | Mirtazapine 15mg/ml oral solution sugar free (A A H Pharmaceuticals Ltd) | Other Antidepressant Drugs |
| 53326 | Venlafaxine 75mg/5ml oral solution | Other Antidepressant Drugs |
| 53394 | Citalopram 20mg tablets (Alliance Healthcare (Distribution) Ltd) | Selective Serotonin Re-uptake Inhibitors |
| 53543 | Zispin SolTab 30mg orodispersible tablets (Necessity Supplies Ltd) | Other Antidepressant Drugs |
| 53648 | Mirtazapine 30mg orodispersible tablets (Actavis UK Ltd) | Other Antidepressant Drugs |
| 53699 | Mirtazapine 15mg tablets (Actavis UK Ltd) | Other Antidepressant Drugs |
| 53787 | Citalopram 10mg tablets (Bristol Laboratories Ltd) | Selective Serotonin Re-uptake Inhibitors |
| 53808 | Trimipramine 10mg tablets (Phoenix Healthcare Distribution Ltd) | Tricyclic And Related Antidepressant Drugs |
| 54012 | Mirtazapine 15mg orodispersible tablets sugar free (Sandoz Ltd) | Other Antidepressant Drugs |
| 54081 | Sertraline 25mg/5ml oral suspension | Selective Serotonin Re-uptake Inhibitors |
| 54342 | Mirtazapine 15mg tablets (Medreich Plc) | Other Antidepressant Drugs |
| 54644 | Mirtazapine 15mg tablets (Pfizer Ltd) | Other Antidepressant Drugs |
| 54792 | Mirtazapine 30mg tablets (Alliance Healthcare (Distribution) Ltd) | Other Antidepressant Drugs |
| 54826 | Sertraline 150mg/5ml oral suspension | Selective Serotonin Re-uptake Inhibitors |
| 54827 | Citalopram 10mg/5ml oral suspension | Selective Serotonin Re-uptake Inhibitors |
| 54877 | Amitriptyline 25mg tablets (Accord Healthcare Ltd) | Tricyclic And Related Antidepressant Drugs/Neuropathic Pain/Prophylaxis Of Migraine |
| 54933 | Sertraline 100mg tablets (PLIVA Pharma Ltd) | Selective Serotonin Re-uptake Inhibitors |
| 55023 | Paroxetine 20mg tablets (Medreich Plc) | Selective Serotonin Re-uptake Inhibitors |
| 55033 | Citalopram 40mg tablets (DE Pharmaceuticals) | Selective Serotonin Re-uptake Inhibitors |
| 55137 | Trazodone 150mg/5ml oral suspension | Tricyclic And Related Antidepressant Drugs |
| 55138 | Trazodone 250mg/5ml oral solution | Tricyclic And Related Antidepressant Drugs |
| 55139 | Amitriptyline 25mg tablets (Alliance Healthcare (Distribution) Ltd) | Tricyclic And Related Antidepressant Drugs/Neuropathic Pain/Prophylaxis Of Migraine |
| 55146 | Sertraline 100mg tablets (A A H Pharmaceuticals Ltd) | Selective Serotonin Re-uptake Inhibitors |
| 55424 | Venlafaxine | Other Antidepressant Drugs |
| 55482 | Mirtazapine 15mg orodispersible tablets (Mylan) | Other Antidepressant Drugs |
| 55488 | Sertraline 50mg tablets (Teva UK Ltd) | Selective Serotonin Re-uptake Inhibitors |
| 55491 | Amitriptyline 10mg tablets (Almus Pharmaceuticals Ltd) | Tricyclic And Related Antidepressant Drugs/Neuropathic Pain/Prophylaxis Of Migraine |
| 55501 | Venlafaxine 150mg Modified-release capsule (Hillcross Pharmaceuticals Ltd) | Other Antidepressant Drugs |
| 55537 | Seroxat 30mg tablets (Lexon (UK) Ltd) | Selective Serotonin Re-uptake Inhibitors |
| 55970 | Nortriptyline 10mg tablets (King Pharmaceuticals Ltd) | Tricyclic And Related Antidepressant Drugs/Nocturnal Enuresis |
| 56009 | Citalopram 20mg tablets (Arrow Generics Ltd) | Selective Serotonin Re-uptake Inhibitors |
| 56209 | Mirtazapine 30mg tablets (Phoenix Healthcare Distribution Ltd) | Other Antidepressant Drugs |
| 56229 | Lofepramine 70mg/5ml oral solution | Tricyclic And Related Antidepressant Drugs |
| 56292 | Citalopram 40mg/ml oral drops sugar free (Actavis UK Ltd) | Selective Serotonin Re-uptake Inhibitors |
| 56355 | Citalopram 10mg tablets (Waymade Healthcare Plc) | Selective Serotonin Re-uptake Inhibitors |
| 56457 | Venlafaxine 75mg tablets (Teva UK Ltd) | Other Antidepressant Drugs |
| 56501 | Tofranil 25mg tablets (Lexon (UK) Ltd) | Tricyclic And Related Antidepressant Drugs/Nocturnal Enuresis |
| 56662 | Venlafaxine 37.5mg tablets (A A H Pharmaceuticals Ltd) | Other Antidepressant Drugs |
| 56703 | Lofepramine 70mg tablets (Sandoz Ltd) | Tricyclic And Related Antidepressant Drugs |
| 57107 | Amitriptyline 10mg tablets (Phoenix Healthcare Distribution Ltd) | Tricyclic And Related Antidepressant Drugs/Neuropathic Pain/Prophylaxis Of Migraine |
| 57226 | Trazodone 25mg/5ml oral suspension | Tricyclic And Related Antidepressant Drugs |
| 57532 | Prozac 20mg capsules (Waymade Healthcare Plc) | Selective Serotonin Re-uptake Inhibitors |
| 57751 | Tonpular XL 150mg capsules (Wockhardt UK Ltd) | Other Antidepressant Drugs |
| 57926 | Dosulepin 75mg/5ml oral solution | Tricyclic And Related Antidepressant Drugs |
| 57936 | Citalopram 40mg/ml oral drops sugar free (A A H Pharmaceuticals Ltd) | Selective Serotonin Re-uptake Inhibitors |
| 57972 | Amitriptyline 10mg tablets (Alliance Healthcare (Distribution) Ltd) | Tricyclic And Related Antidepressant Drugs/Neuropathic Pain/Prophylaxis Of Migraine |
| 57978 | Trimipramine 25mg tablets (Waymade Healthcare Plc) | Tricyclic And Related Antidepressant Drugs |
| 58291 | Mirtazapine 15mg orodispersible tablets (Pfizer Ltd) | Other Antidepressant Drugs |
| 58450 | Feprapax 70mg tablets (Ashbourne Pharmaceuticals Ltd) | Tricyclic And Related Antidepressant Drugs |
| 58476 | Citalopram 20mg tablets (Aurobindo Pharma Ltd) | Selective Serotonin Re-uptake Inhibitors |
| 58625 | Mirtazapine 45mg tablets (Actavis UK Ltd) | Other Antidepressant Drugs |
| 58664 | Sertraline 50mg tablets (Mylan) | Selective Serotonin Re-uptake Inhibitors |
| 58681 | Venladex XL 75mg tablets (Dexcel-Pharma Ltd) | Other Antidepressant Drugs |
| 58723 | Sertraline 50mg tablets (Accord Healthcare Ltd) | Selective Serotonin Re-uptake Inhibitors |
| 58726 | Venladex XL 150mg tablets (Dexcel-Pharma Ltd) | Other Antidepressant Drugs |
| 58837 | Venlafaxine 37.5mg modified-release capsules | Other Antidepressant Drugs |
| 59035 | Venlablue XL 37.5mg capsules (Bluefish Pharmaceuticals AB) | Other Antidepressant Drugs |
| 59161 | Amitriptyline 10mg tablets (Waymade Healthcare Plc) | Tricyclic And Related Antidepressant Drugs/Neuropathic Pain/Prophylaxis Of Migraine |
| 59193 | Citalopram 10mg tablets (Ranbaxy (UK) Ltd) | Selective Serotonin Re-uptake Inhibitors |
| 59288 | Paroxetine 10mg tablets (Actavis UK Ltd) | Selective Serotonin Re-uptake Inhibitors |
| 59358 | Fluoxetine 20mg capsules (Milpharm Ltd) | Selective Serotonin Re-uptake Inhibitors |
| 59563 | Venlafaxine 75mg modified-release capsules (Kent Pharmaceuticals Ltd) | Other Antidepressant Drugs |
| 59600 | Sertraline 100mg tablets (Almus Pharmaceuticals Ltd) | Selective Serotonin Re-uptake Inhibitors |
| 59650 | Citalopram 10mg tablets (Aurobindo Pharma Ltd) | Selective Serotonin Re-uptake Inhibitors |
| 59694 | Mirtazapine 30mg orodispersible tablets (Phoenix Healthcare Distribution Ltd) | Other Antidepressant Drugs |
| 59753 | Sunveniz XL 150mg tablets (Sun Pharmaceuticals UK Ltd) | Other Antidepressant Drugs |
| 59820 | Amitriptyline 50mg/5ml oral solution sugar free (Wockhardt UK Ltd) | Tricyclic And Related Antidepressant Drugs/Neuropathic Pain/Prophylaxis Of Migraine |
| 59923 | Venlafaxine 37.5mg tablets (Bristol Laboratories Ltd) | Other Antidepressant Drugs |
| 59931 | Trazodone 50mg/5ml oral solution sugar free (A A H Pharmaceuticals Ltd) | Tricyclic And Related Antidepressant Drugs |
| 59953 | Mirtazapine 15mg tablets (Almus Pharmaceuticals Ltd) | Other Antidepressant Drugs |
| 59954 | Mirtazapine 45mg tablets (Almus Pharmaceuticals Ltd) | Other Antidepressant Drugs |
| 60138 | Fluoxetine 20mg orodispersible tablets sugar free | Selective Serotonin Re-uptake Inhibitors |
| 60355 | Amitriptyline 25mg tablets (Phoenix Healthcare Distribution Ltd) | Tricyclic And Related Antidepressant Drugs/Neuropathic Pain/Prophylaxis Of Migraine |
| 60370 | Zispin SolTab 15mg orodispersible tablets (Mawdsley-Brooks & Company Ltd) | Other Antidepressant Drugs |
| 60410 | Amitriptyline 25mg/5ml oral solution sugar free (Wockhardt UK Ltd) | Tricyclic And Related Antidepressant Drugs/Neuropathic Pain/Prophylaxis Of Migraine |
| 60449 | Venlafaxine 75mg tablets (A A H Pharmaceuticals Ltd) | Other Antidepressant Drugs |
| 60534 | Fluoxetine 20mg dispersible tablets sugar free | Selective Serotonin Re-uptake Inhibitors |
| 60538 | Mirtazapine 30mg tablets (DE Pharmaceuticals) | Other Antidepressant Drugs |
| 60549 | Venlafaxine 150mg modified-release capsules (Kent Pharmaceuticals Ltd) | Other Antidepressant Drugs |
| 60568 | Citalopram 20mg tablets (Waymade Healthcare Plc) | Selective Serotonin Re-uptake Inhibitors |
| 60591 | Lofepramine 70mg tablets (Teva UK Ltd) | Tricyclic And Related Antidepressant Drugs |
| 60619 | Fluoxetine 20mg/5ml oral solution (Kent Pharmaceuticals Ltd) | Selective Serotonin Re-uptake Inhibitors |
| 60839 | Citalopram 40mg tablets (Almus Pharmaceuticals Ltd) | Selective Serotonin Re-uptake Inhibitors |
| 60843 | Sunveniz XL 75mg tablets (Sun Pharmaceuticals UK Ltd) | Other Antidepressant Drugs |
| 60888 | Citalopram 10mg tablets (Sigma Pharmaceuticals Plc) | Selective Serotonin Re-uptake Inhibitors |
| 60895 | Venlafaxine 37.5mg tablets (Teva UK Ltd) | Other Antidepressant Drugs |
| 60929 | Protriptyline 5mg tablets | Tricyclic And Related Antidepressant Drugs |
| 60962 | Fluoxetine 20mg capsules (Alliance Healthcare (Distribution) Ltd) | Selective Serotonin Re-uptake Inhibitors |
| 61236 | Bonilux XL 150mg capsules (Sandoz Ltd) | Other Antidepressant Drugs |
| 61335 | Prozac 20mg capsules (Mawdsley-Brooks & Company Ltd) | Selective Serotonin Re-uptake Inhibitors |
| 61503 | Sertraline 100mg tablets (Actavis UK Ltd) | Selective Serotonin Re-uptake Inhibitors |
| 61547 | Mirtazapine 15mg/ml oral solution sugar free (DE Pharmaceuticals) | Other Antidepressant Drugs |
| 61657 | Trazodone 75mg/5ml oral solution | Tricyclic And Related Antidepressant Drugs |
| 61835 | Amitriptyline 10mg tablets (DE Pharmaceuticals) | Tricyclic And Related Antidepressant Drugs/Neuropathic Pain/Prophylaxis Of Migraine |
| 61842 | Trazodone 50mg/5ml oral solution | Tricyclic And Related Antidepressant Drugs |
| 61856 | Mirtazapine 15mg orodispersible tablets (Consilient Health Ltd) | Other Antidepressant Drugs |
| 62155 | Fluoxetine 20mg capsules (Phoenix Healthcare Distribution Ltd) | Selective Serotonin Re-uptake Inhibitors |
| 62335 | Olena 20mg dispersible tablets (AMCo) | Selective Serotonin Re-uptake Inhibitors |
| 62620 | Clomipramine 10mg capsules (Mylan) | Tricyclic And Related Antidepressant Drugs |
| 62681 | Dosulepin 75mg tablets (Sandoz Ltd) | Tricyclic And Related Antidepressant Drugs |
| 62688 | Duloxetine 30mg gastro-resistant capsules (Sigma Pharmaceuticals Plc) | Other Antidepressant Drugs/Treatment Of Diabetic Nephropathy And Neuropathy |
| 62692 | Sertraline 100mg tablets (Bristol Laboratories Ltd) | Selective Serotonin Re-uptake Inhibitors |
| 62693 | Sertraline 50mg tablets (Bristol Laboratories Ltd) | Selective Serotonin Re-uptake Inhibitors |
| 62734 | Venlafaxine 150mg/5ml oral suspension | Other Antidepressant Drugs |
| 62819 | Sertraline 12.5mg/5ml oral suspension | Selective Serotonin Re-uptake Inhibitors |
| 62927 | Sertraline 50mg tablets (Wockhardt UK Ltd) | Selective Serotonin Re-uptake Inhibitors |
| 62950 | Sertraline 100mg tablets (Accord Healthcare Ltd) | Selective Serotonin Re-uptake Inhibitors |
| 63216 | Cymbalta 60mg gastro-resistant capsules (Mawdsley-Brooks & Company Ltd) | Other Antidepressant Drugs/Treatment Of Diabetic Nephropathy And Neuropathy |
| 63268 | Venlafaxine 75mg/5ml oral suspension | Other Antidepressant Drugs |
| 63276 | Nortriptyline 25mg tablets (Alliance Healthcare (Distribution) Ltd) | Tricyclic And Related Antidepressant Drugs/Nocturnal Enuresis |
| 63370 | Duloxetine 30mg gastro-resistant capsules (Mawdsley-Brooks & Company Ltd) | Other Antidepressant Drugs/Treatment Of Diabetic Nephropathy And Neuropathy |
| 63403 | Mirtazapine 30mg tablets (Teva UK Ltd) | Other Antidepressant Drugs |
| 63441 | Citalopram 10mg tablets (Rivopharm (UK) Ltd) | Selective Serotonin Re-uptake Inhibitors |
| 63481 | Sertraline 50mg tablets (Aurobindo Pharma Ltd) | Selective Serotonin Re-uptake Inhibitors |
| 63763 | Duloxetine 60mg gastro-resistant capsules (A A H Pharmaceuticals Ltd) | Other Antidepressant Drugs/Treatment Of Diabetic Nephropathy And Neuropathy |
| 63859 | Venlafaxine 75mg tablets (Waymade Healthcare Plc) | Other Antidepressant Drugs |
| 63916 | Escitalopram 10mg tablets (Actavis UK Ltd) | Selective Serotonin Re-uptake Inhibitors |
| 63953 | Cipramil 20mg tablets (DE Pharmaceuticals) | Selective Serotonin Re-uptake Inhibitors |
| 64000 | Amitriptyline 10mg/5ml oral solution sugar free | Tricyclic And Related Antidepressant Drugs/Neuropathic Pain/Prophylaxis Of Migraine |
| 64101 | Mirtazapine 15mg orodispersible tablets (Actavis UK Ltd) | Other Antidepressant Drugs |
| 64139 | Mirtazapine 45mg orodispersible tablets (Mylan) | Other Antidepressant Drugs |
| 64141 | Amitriptyline 5mg/5ml oral solution | Tricyclic And Related Antidepressant Drugs/Neuropathic Pain/Prophylaxis Of Migraine |
| 64223 | Mirtazapine 45mg tablets (Teva UK Ltd) | Other Antidepressant Drugs |
| 64330 | Amitriptyline 50mg tablets (Almus Pharmaceuticals Ltd) | Tricyclic And Related Antidepressant Drugs/Neuropathic Pain/Prophylaxis Of Migraine |
| 64423 | Citalopram 10mg tablets (Accord Healthcare Ltd) | Selective Serotonin Re-uptake Inhibitors |
| 64442 | Duloxetine 60mg gastro-resistant capsules (Teva UK Ltd) | Other Antidepressant Drugs/Treatment Of Diabetic Nephropathy And Neuropathy |
| 64458 | Clomipramine 25mg/5ml oral suspension | Tricyclic And Related Antidepressant Drugs |
| 64647 | Amitriptyline 25mg tablets (DE Pharmaceuticals) | Tricyclic And Related Antidepressant Drugs/Neuropathic Pain/Prophylaxis Of Migraine |
| 64785 | Paroxetine 30mg tablets (Alliance Healthcare (Distribution) Ltd) | Selective Serotonin Re-uptake Inhibitors |
| 65152 | Trazodone 100mg/5ml oral solution | Tricyclic And Related Antidepressant Drugs |
| 65213 | Trimipramine 50mg/5ml oral solution | Tricyclic And Related Antidepressant Drugs |
| 65237 | Nortriptyline 10mg tablets (A A H Pharmaceuticals Ltd) | Tricyclic And Related Antidepressant Drugs/Nocturnal Enuresis |
| 65439 | Amitriptyline 25mg tablets (Sandoz Ltd) | Tricyclic And Related Antidepressant Drugs |
| 65445 | Trimipramine 50mg capsules (A A H Pharmaceuticals Ltd) | Tricyclic And Related Antidepressant Drugs |
| 65482 | Vortioxetine 5mg tablets | Other Antidepressant Drugs |
| 65483 | Vortioxetine 10mg tablets | Other Antidepressant Drugs |
| 65555 | Mirtazapine 15mg orodispersible tablets (Sigma Pharmaceuticals Plc) | Other Antidepressant Drugs |
| 65618 | Duloxetine 30mg gastro-resistant capsules (A A H Pharmaceuticals Ltd) | Other Antidepressant Drugs/Treatment Of Diabetic Nephropathy And Neuropathy |
| 65666 | Venlafaxine 225mg modified-release capsules | Other Antidepressant Drugs |
| 65738 | Efexor 37.5mg tablets (Sigma Pharmaceuticals Plc) | Other Antidepressant Drugs |
| 65762 | Clomipramine 25mg capsules (Waymade Healthcare Plc) | Tricyclic And Related Antidepressant Drugs |
| 65771 | Sertraline 200mg/5ml oral suspension (Special Order) | Selective Serotonin Re-uptake Inhibitors |
| 65804 | Clomipramine 50mg capsules (Teva UK Ltd) | Tricyclic And Related Antidepressant Drugs |
| 65809 | Duloxetine 30mg gastro-resistant capsules (Actavis UK Ltd) | Other Antidepressant Drugs/Treatment Of Diabetic Nephropathy And Neuropathy |
| 65879 | Amitriptyline 10mg tablets (Sigma Pharmaceuticals Plc) | Tricyclic And Related Antidepressant Drugs/Neuropathic Pain/Prophylaxis Of Migraine |
| 65888 | Duloxetine 60mg gastro-resistant capsules (Actavis UK Ltd) | Other Antidepressant Drugs/Treatment Of Diabetic Nephropathy And Neuropathy |
| 65892 | Duloxetine 60mg gastro-resistant capsules (Mawdsley-Brooks & Company Ltd) | Other Antidepressant Drugs/Treatment Of Diabetic Nephropathy And Neuropathy/Urinary Incontinence |
| 65899 | Efexor XL 225mg capsules (Pfizer Ltd) | Other Antidepressant Drugs |
| 65987 | Amitriptyline 25mg tablets (Crescent Pharma Ltd) | Tricyclic And Related Antidepressant Drugs/Neuropathic Pain/Prophylaxis Of Migraine |
| 66100 | Lofepramine 70mg tablets (DE Pharmaceuticals) | Tricyclic And Related Antidepressant Drugs |
| 66183 | Mirtazapine 15mg tablets (Alliance Healthcare (Distribution) Ltd) | Other Antidepressant Drugs |
| 66201 | Nortriptyline 25mg tablets (Sigma Pharmaceuticals Plc) | Tricyclic And Related Antidepressant Drugs/Nocturnal Enuresis |
| 66292 | Seroxat 10mg tablets (Waymade Healthcare Plc) | Selective Serotonin Re-uptake Inhibitors |
| 66405 | Duloxetine 60mg gastro-resistant capsules (DE Pharmaceuticals) | Other Antidepressant Drugs/Treatment Of Diabetic Nephropathy And Neuropathy |
| 66412 | Duloxetine 30mg gastro-resistant capsules (DE Pharmaceuticals) | Other Antidepressant Drugs/Treatment Of Diabetic Nephropathy And Neuropathy |
| 66413 | Sertraline 100mg tablets (Ranbaxy (UK) Ltd) | Selective Serotonin Re-uptake Inhibitors |
| 66437 | Venlafaxine 75mg tablets (DE Pharmaceuticals) | Other Antidepressant Drugs |
| 66493 | Trimipramine 25mg/5ml oral suspension | Tricyclic And Related Antidepressant Drugs |
| 66560 | Sertraline 100mg tablets (Mylan) | Selective Serotonin Re-uptake Inhibitors |
| 66572 | Amitriptyline 25mg tablets (Sigma Pharmaceuticals Plc) | Tricyclic And Related Antidepressant Drugs/Neuropathic Pain/Prophylaxis Of Migraine |
| 66578 | Amitriptyline 10mg tablets (Mawdsley-Brooks & Company Ltd) | Tricyclic And Related Antidepressant Drugs/Neuropathic Pain/Prophylaxis Of Migraine |
| 66579 | Amitriptyline 25mg tablets (Mawdsley-Brooks & Company Ltd) | Tricyclic And Related Antidepressant Drugs/Neuropathic Pain/Prophylaxis Of Migraine |
| 66580 | Mirtazapine 15mg orodispersible tablets (Bluefish Pharmaceuticals AB) | Other Antidepressant Drugs |
| 66744 | Fluoxetine 20mg capsules (Morningside Healthcare Ltd) | Selective Serotonin Re-uptake Inhibitors |
| 66749 | Trazodone 10mg/5ml oral solution | Tricyclic And Related Antidepressant Drugs |
| 66752 | Mirtazapine 15mg tablets (Aurobindo Pharma Ltd) | Other Antidepressant Drugs |
| 66890 | Vortioxetine 20mg tablets | Other Antidepressant Drugs |
| 66919 | Trimipramine 50mg capsules (Waymade Healthcare Plc) | Tricyclic And Related Antidepressant Drugs |
| 67092 | Fluoxetine 20mg capsules (Waymade Healthcare Plc) | Selective Serotonin Re-uptake Inhibitors |
| 67097 | Citalopram 20mg tablets (Accord Healthcare Ltd) | Selective Serotonin Re-uptake Inhibitors |
| 67127 | Amitriptyline 25mg/5ml oral solution sugar free (DE Pharmaceuticals) | Tricyclic And Related Antidepressant Drugs/Neuropathic Pain/Prophylaxis Of Migraine |
| 67259 | Paroxetine 10mg/5ml oral solution | Selective Serotonin Re-uptake Inhibitors |
| 67271 | Efexor 37.5mg tablets (Waymade Healthcare Plc) | Other Antidepressant Drugs |
| 67272 | Zispin 30mg tablets (Waymade Healthcare Plc) | Other Antidepressant Drugs |
| 67288 | Efexor 75mg tablets (Dowelhurst Ltd) | Other Antidepressant Drugs |
| 67431 | Fluoxetine 10mg capsules | Selective Serotonin Re-uptake Inhibitors |
| 67496 | Fluoxetine 30mg capsules | Selective Serotonin Re-uptake Inhibitors |
| 67562 | Fluoxetine 40mg capsules | Selective Serotonin Re-uptake Inhibitors |
| 67563 | Vensir XL 225mg capsules (Morningside Healthcare Ltd) | Other Antidepressant Drugs |
| 67728 | Dosulepin 75mg tablets (Alliance Healthcare (Distribution) Ltd) | Tricyclic And Related Antidepressant Drugs |
| 67730 | Sertraline 50mg tablets (Ranbaxy (UK) Ltd) | Selective Serotonin Re-uptake Inhibitors |
| 67736 | Fluoxetine 20mg capsules (Dr Reddy's Laboratories (UK) Ltd) | Selective Serotonin Re-uptake Inhibitors |
| 67742 | Lofepramine 70mg tablets (Mylan) | Tricyclic And Related Antidepressant Drugs |
| 67758 | Prozac 20mg capsules (DE Pharmaceuticals) | Selective Serotonin Re-uptake Inhibitors |
| 67769 | Fluoxetine 20mg capsules (Strides Shasun (UK) Ltd) | Selective Serotonin Re-uptake Inhibitors |
| 67874 | Brintellix 10mg tablets (Lundbeck Ltd) | Other Antidepressant Drugs |
| 67888 | Fluoxetine 60mg capsules (Kent Pharmaceuticals Ltd) | Selective Serotonin Re-uptake Inhibitors |
| 67928 | Sertraline 100mg tablets (Aurobindo Pharma Ltd) | Selective Serotonin Re-uptake Inhibitors |
| 67935 | Imipramine 10mg tablets (Almus Pharmaceuticals Ltd) | Tricyclic And Related Antidepressant Drugs/Nocturnal Enuresis |
| 67990 | Prothiaden 25mg capsules (Sigma Pharmaceuticals Plc) | Tricyclic And Related Antidepressant Drugs |
| 68050 | Venlafaxine 37.5mg tablets (Alliance Healthcare (Distribution) Ltd) | Other Antidepressant Drugs |
| 68052 | Mirtazapine 30mg orodispersible tablets (Almus Pharmaceuticals Ltd) | Other Antidepressant Drugs |
| 68096 | Duloxetine 60mg gastro-resistant capsules (Zentiva) | Other Antidepressant Drugs/Treatment Of Diabetic Nephropathy And Neuropathy |
| 68228 | Nortriptyline 10mg/5ml oral suspension | Tricyclic And Related Antidepressant Drugs/Nocturnal Enuresis |
| 68266 | Fluoxetine 20mg/5ml oral solution sugar free (Actavis UK Ltd) | Selective Serotonin Re-uptake Inhibitors |
| 68325 | Paroxetine 40mg tablets | Selective Serotonin Re-uptake Inhibitors |
| 68544 | Mirtazapine 2mg capsules | Other Antidepressant Drugs |
| 68657 | Lofepramine 70mg tablets (Kent Pharmaceuticals Ltd) | Tricyclic And Related Antidepressant Drugs |
| 68665 | Clomipramine 10mg capsules (Almus Pharmaceuticals Ltd) | Tricyclic And Related Antidepressant Drugs |
| 68680 | Mirtazapine 15mg orodispersible tablets (Mawdsley-Brooks & Company Ltd) | Other Antidepressant Drugs |
| 68756 | Sertraline 100mg tablets (Sandoz Ltd) | Selective Serotonin Re-uptake Inhibitors |
| 68876 | Venlafaxine 75mg modified-release capsules (Mawdsley-Brooks & Company Ltd) | Other Antidepressant Drugs |
| 68933 | Mirtazapine 30mg tablets (PLIVA Pharma Ltd) | Other Antidepressant Drugs |
| 69005 | Mirtazapine 30mg tablets (Almus Pharmaceuticals Ltd) | Other Antidepressant Drugs |
| 69317 | Nortriptyline 50mg tablets | Tricyclic And Related Antidepressant Drugs/Nocturnal Enuresis |
| 69355 | Trazodone Oral solution | Tricyclic And Related Antidepressant Drugs |
| 69420 | Mirtazapine 30mg orodispersible tablets (Aurobindo Pharma Ltd) | Other Antidepressant Drugs |
| 69428 | Duloxetine 60mg gastro-resistant capsules (Alliance Healthcare (Distribution) Ltd) | Other Antidepressant Drugs/Treatment Of Diabetic Nephropathy And Neuropathy |
| 69525 | Fluoxetine 20mg capsules (Medreich Plc) | Selective Serotonin Re-uptake Inhibitors |
| 69542 | Prozac 20mg capsules (Necessity Supplies Ltd) | Selective Serotonin Re-uptake Inhibitors |
| 69571 | Citalopram 40mg tablets (Accord Healthcare Ltd) | Selective Serotonin Re-uptake Inhibitors |
| 69685 | Fluoxetine 20mg/5ml oral solution sugar free (Morningside Healthcare Ltd) | Selective Serotonin Re-uptake Inhibitors |
| 69712 | Amitriptyline 50mg tablets (Sigma Pharmaceuticals Plc) | Tricyclic And Related Antidepressant Drugs/Neuropathic Pain/Prophylaxis Of Migraine |
| 69725 | Sertraline 50mg tablets (Crescent Pharma Ltd) | Selective Serotonin Re-uptake Inhibitors |
| 69726 | Sertraline 100mg tablets (Crescent Pharma Ltd) | Selective Serotonin Re-uptake Inhibitors |
| 69752 | Duloxetine 60mg gastro-resistant capsules (Creo Pharma Ltd) | Other Antidepressant Drugs/Treatment Of Diabetic Nephropathy And Neuropathy |
| 69819 | Vencarm XL 37.5mg capsules (Aspire Pharma Ltd) | Other Antidepressant Drugs |
| 69898 | Sertraline 50mg tablets (Sandoz Ltd) | Selective Serotonin Re-uptake Inhibitors |
| 69941 | Fluoxetine 10mg capsules (A A H Pharmaceuticals Ltd) | Selective Serotonin Re-uptake Inhibitors |
| 69965 | Duloxetine 60mg gastro-resistant capsules (Consilient Health Ltd) | Other Antidepressant Drugs/Treatment Of Diabetic Nephropathy And Neuropathy |
| 69991 | Brintellix 20mg tablets (Lundbeck Ltd) | Other Antidepressant Drugs |
| 69992 | Brintellix 5mg tablets (Lundbeck Ltd) | Other Antidepressant Drugs |
| 70287 | Imipramine 10mg tablets (Sigma Pharmaceuticals Plc) | Tricyclic And Related Antidepressant Drugs/Nocturnal Enuresis |
| 70300 | Amitriptyline 10mg/5ml oral solution sugar free (Alliance Healthcare (Distribution) Ltd) | Tricyclic And Related Antidepressant Drugs/Neuropathic Pain/Prophylaxis Of Migraine |
| 70315 | Vencarm XL 75mg capsules (Aspire Pharma Ltd) | Other Antidepressant Drugs |
| 70353 | Venlafaxine 37.5mg tablets (DE Pharmaceuticals) | Other Antidepressant Drugs |
| 70405 | Duloxetine 30mg gastro-resistant capsules (Teva UK Ltd) | Other Antidepressant Drugs/Treatment Of Diabetic Nephropathy And Neuropathy |
| 70420 | Vencarm XL 150mg capsules (Aspire Pharma Ltd) | Other Antidepressant Drugs |
| 70495 | Vencarm XL 225mg capsules (Aspire Pharma Ltd) | Other Antidepressant Drugs |
| 70521 | Trazodone 50mg/5ml oral solution sugar free (AMCo) | Tricyclic And Related Antidepressant Drugs |
| 70593 | Dosulepin 25mg/5ml oral suspension | Tricyclic And Related Antidepressant Drugs |
| 70728 | Duloxetine 30mg gastro-resistant capsules (Alliance Healthcare (Distribution) Ltd) | Other Antidepressant Drugs/Treatment Of Diabetic Nephropathy And Neuropathy |
| 70790 | Citalopram 40mg tablets (Aurobindo Pharma Ltd) | Selective Serotonin Re-uptake Inhibitors |
| 70806 | Venlafaxine 150mg modified-release capsules (DE Pharmaceuticals) | Other Antidepressant Drugs |
| 70838 | Dosulepin 25mg Capsule (Celltech Pharma Europe Ltd) | Tricyclic And Related Antidepressant Drugs |
| 70931 | Venlasov XL 75mg capsules (Sovereign Medical Ltd) | Other Antidepressant Drugs |
| 70991 | Amitriptyline 10mg tablets (Arrow Generics Ltd) | Tricyclic And Related Antidepressant Drugs/Neuropathic Pain/Prophylaxis Of Migraine |
| 71005 | Citalopram 10mg tablets (DE Pharmaceuticals) | Selective Serotonin Re-uptake Inhibitors |
| 71023 | Dosulepin 25mg/5ml oral solution sugar free (Special Order) | Tricyclic And Related Antidepressant Drugs |
| 71031 | Trazodone 50mg capsules (Actavis UK Ltd) | Tricyclic And Related Antidepressant Drugs |
| 71042 | Amitriptyline 25mg tablets (Arrow Generics Ltd) | Tricyclic And Related Antidepressant Drugs/Neuropathic Pain/Prophylaxis Of Migraine |
| 71059 | Dosulepin 75mg tablets (Sovereign Medical Ltd) | Tricyclic And Related Antidepressant Drugs |
| 71253 | Imipramine 10mg tablets (DE Pharmaceuticals) | Tricyclic And Related Antidepressant Drugs/Nocturnal Enuresis |
| 71257 | Venlafaxine 75mg modified-release capsules (DE Pharmaceuticals) | Other Antidepressant Drugs |
| 71543 | Mirtazapine 30mg tablets (Sigma Pharmaceuticals Plc) | Other Antidepressant Drugs |
| 71782 | Venlafaxine 150mg modified-release capsules (Mawdsley-Brooks & Company Ltd) | Other Antidepressant Drugs |
| 71806 | Amphero XL 75mg capsules (Mylan) | Other Antidepressant Drugs |
| 71848 | Citalopram 20mg/5ml oral suspension | Selective Serotonin Re-uptake Inhibitors |
| 71852 | Fluoxetine 20mg capsules (Accord Healthcare Ltd) | Selective Serotonin Re-uptake Inhibitors |
| 71932 | Venlasov XL 150mg capsules (Sovereign Medical Ltd) | Other Antidepressant Drugs |
