# Supplementary material for: Safety of antidepressants in a primary care cohort of adults with obesity and depression
Source: PLoS One. 2021 Jan 29;16(1):e0245722. doi: 10.1371/journal.pone.0245722 (PMC7846000; doi:10.1371/journal.pone.0245722)
Supplement: S8 Table — (DOCX) [file pone.0245722.s011.docx]

**Table S8. Outcome measures (Read codes and ICD-10 codes from secondary care/mortality data)**

| **Cardiovascular disease (primary care and secondary care/mortality)** | | |
| --- | --- | --- |
| **PRIMARY CARE** | | |
| **Read Code** | **Read Code Description** | |
| G66..00 | Stroke and cerebrovascular accident unspecified | |
| G66..11 | CVA unspecified | |
| 662e.00 | Stroke/CVA annual review | |
| G60..00 | Subarachnoid haemorrhage | |
| G64z.00 | Cerebral infarction NOS | |
| G64..11 | CVA - cerebral artery occlusion | |
| G64..00 | Cerebral arterial occlusion | |
| G66..13 | CVA - Cerebrovascular accident unspecified | |
| G61..00 | Intracerebral haemorrhage | |
| G61..11 | CVA - cerebrovascular accid due to intracerebral haemorrhage | |
| 14A7.11 | H/O: CVA | |
| 14A7.12 | H/O: stroke | |
| 14A7.00 | H/O: CVA/stroke | |
| G64z.12 | Cerebellar infarction | |
| G66..12 | Stroke unspecified | |
| G667.00 | Left sided CVA | |
| G64..13 | Stroke due to cerebral arterial occlusion | |
| G668.00 | Right sided CVA | |
| G64..12 | Infarction - cerebral | |
| G623.00 | Subdural haemorrhage NOS | |
| G640.00 | Cerebral thrombosis | |
| G622.00 | Subdural haematoma - nontraumatic | |
| G621.00 | Subdural haemorrhage - nontraumatic | |
| G6X..00 | Cerebrl infarctn due/unspcf occlusn or sten/cerebrl artrs | |
| G61..12 | Stroke due to intracerebral haemorrhage | |
| G64z200 | Left sided cerebral infarction | |
| G64z300 | Right sided cerebral infarction | |
| G61z.00 | Intracerebral haemorrhage NOS | |
| G613.00 | Cerebellar haemorrhage | |
| G664.00 | Cerebellar stroke syndrome | |
| G63y000 | Cerebral infarct due to thrombosis of precerebral arteries | |
| G663.00 | Brain stem stroke syndrome | |
| G641.00 | Cerebral embolism | |
| G64z400 | Infarction of basal ganglia | |
| G640000 | Cerebral infarction due to thrombosis of cerebral arteries | |
| G60z.00 | Subarachnoid haemorrhage NOS | |
| G62z.00 | Intracranial haemorrhage NOS | |
| G64z000 | Brainstem infarction | |
| G617.00 | Intracerebral haemorrhage, intraventricular | |
| ZV12511 | [V]Personal history of stroke | |
| ZV12512 | [V]Personal history of cerebrovascular accident (CVA) | |
| Gyu6400 | [X]Other cerebral infarction | |
| G665.00 | Pure motor lacunar syndrome | |
| G676.00 | Nonpyogenic venous sinus thrombosis | |
| G68X.00 | Sequelae of stroke,not specfd as h'morrhage or infarction | |
| G6W..00 | Cereb infarct due unsp occlus/stenos precerebr arteries | |
| G64z.11 | Brainstem infarction NOS | |
| G600.00 | Ruptured berry aneurysm | |
| G63y100 | Cerebral infarction due to embolism of precerebral arteries | |
| G614.00 | Pontine haemorrhage | |
| G602.00 | Subarachnoid haemorrhage from middle cerebral artery | |
| G64z111 | Lateral medullary syndrome | |
| G676000 | Cereb infarct due cerebral venous thrombosis, nonpyogenic | |
| G611.00 | Internal capsule haemorrhage | |
| G61X000 | Left sided intracerebral haemorrhage, unspecified | |
| G60X.00 | Subarachnoid haemorrh from intracranial artery, unspecif | |
| G620.00 | Extradural haemorrhage - nontraumatic | |
| G61X100 | Right sided intracerebral haemorrhage, unspecified | |
| G641000 | Cerebral infarction due to embolism of cerebral arteries | |
| G62..00 | Other and unspecified intracranial haemorrhage | |
| G673300 | Vertebral artery dissection | |
| G682.00 | Sequelae of other nontraumatic intracranial haemorrhage | |
| 7P24200 | Delivery of rehabilitation for stroke | |
| G603.00 | Subarachnoid haemorrhage from anterior communicating artery | |
| G666.00 | Pure sensory lacunar syndrome | |
| G67A.00 | Cerebral vein thrombosis | |
| G683.00 | Sequelae of cerebral infarction | |
| G612.00 | Basal nucleus haemorrhage | |
| G610.00 | Cortical haemorrhage | |
| G61X.00 | Intracerebral haemorrhage in hemisphere, unspecified | |
| Gyu6300 | [X]Cerebrl infarctn due/unspcf occlusn or sten/cerebrl artrs | |
| L440.11 | CVA - cerebrovascular accident in the puerperium | |
| G604.00 | Subarachnoid haemorrhage from posterior communicating artery | |
| G619.00 | Lobar cerebral haemorrhage | |
| 14AK.00 | H/O: Stroke in last year | |
| G641.11 | Cerebral embolus | |
| 1M4..00 | Central post-stroke pain | |
| G605.00 | Subarachnoid haemorrhage from basilar artery | |
| Gyu6.00 | [X]Cerebrovascular diseases | |
| Gyu6G00 | [X]Cereb infarct due unsp occlus/stenos precerebr arteries | |
| G63..11 | Infarction - precerebral | |
| G64z100 | Wallenberg syndrome | |
| G680.00 | Sequelae of subarachnoid haemorrhage | |
| G616.00 | External capsule haemorrhage | |
| G63y.00 | Other precerebral artery occlusion | |
| G681.00 | Sequelae of intracerebral haemorrhage | |
| G618.00 | Intracerebral haemorrhage, multiple localized | |
| Gyu6600 | [X]Occlusion and stenosis of other cerebral arteries | |
| 662M100 | Stroke 6 month review | |
| Gyu6200 | [X]Other intracerebral haemorrhage | |
| 662e.11 | Stroke annual review | |
| 662M200 | Stroke initial post discharge review | |
| Gyu6500 | [X]Occlusion and stenosis of other precerebral arteries | |
| G615.00 | Bulbar haemorrhage | |
| Gyu6100 | [X]Other subarachnoid haemorrhage | |
| Gyu6F00 | [X]Intracerebral haemorrhage in hemisphere, unspecified | |
| G606.00 | Subarachnoid haemorrhage from vertebral artery | |
| L440.12 | Stroke in the puerperium | |
| G601.00 | Subarachnoid haemorrhage from carotid siphon and bifurcation | |
| 661M700 | Stroke self-management plan agreed | |
| Gyu6C00 | [X]Sequelae of stroke,not specfd as h'morrhage or infarction | |
| Gyu6000 | [X]Subarachnoid haemorrhage from other intracranial arteries | |
| Gyu6E00 | [X]Subarachnoid haemorrh from intracranial artery, unspecif | |
| Gyu6700 | [X]Other specified cerebrovascular diseases | |
| Gyu6A00 | [X]Other cerebrovascular disorders in diseases CE | |
| 661N700 | Stroke self-management plan review | |
| G363.00 | Ruptur cardiac wall w'out haemopericard/cur comp fol ac MI | |
| G3...00 | Ischaemic heart disease | |
| G3...11 | Arteriosclerotic heart disease | |
| G3...12 | Atherosclerotic heart disease | |
| G3...13 | IHD - Ischaemic heart disease | |
| G310.00 | Postmyocardial infarction syndrome | |
| G31..00 | Other acute and subacute ischaemic heart disease | |
| G30z.00 | Acute myocardial infarction NOS | |
| G30yz00 | Other acute myocardial infarction NOS | |
| G30y200 | Acute septal infarction | |
| G30y100 | Acute papillary muscle infarction | |
| G30y000 | Acute atrial infarction | |
| G30y.00 | Other acute myocardial infarction | |
| G30X000 | Acute ST segment elevation myocardial infarction | |
| G30X.00 | Acute transmural myocardial infarction of unspecif site | |
| G30B.00 | Acute posterolateral myocardial infarction | |
| G30A.00 | Mural thrombosis | |
| G308.00 | Inferior myocardial infarction NOS | |
| G309.00 | Acute Q-wave infarct | |
| G307100 | Acute non-ST segment elevation myocardial infarction | |
| G307000 | Acute non-Q wave infarction | |
| G307.00 | Acute subendocardial infarction | |
| G306.00 | True posterior myocardial infarction | |
| G305.00 | Lateral myocardial infarction NOS | |
| G304.00 | Posterior myocardial infarction NOS | |
| G303.00 | Acute inferoposterior infarction | |
| G302.00 | Acute inferolateral infarction | |
| G301z00 | Anterior myocardial infarction NOS | |
| G301100 | Acute anteroseptal infarction | |
| G301000 | Acute anteroapical infarction | |
| G301.00 | Other specified anterior myocardial infarction | |
| G300.00 | Acute anterolateral infarction | |
| G30..17 | Silent myocardial infarction | |
| G30..16 | Thrombosis - coronary | |
| G30..15 | MI - acute myocardial infarction | |
| G30..14 | Heart attack | |
| G30..13 | Cardiac rupture following myocardial infarction (MI) | |
| G30..12 | Coronary thrombosis | |
| G30..11 | Attack - heart | |
| G30..00 | Acute myocardial infarction | |
| G312.00 | Coronary thrombosis not resulting in myocardial infarction | |
| G31y200 | Subendocardial ischaemia | |
| G3z..00 | Ischaemic heart disease NOS | |
| G311011 | MI - myocardial infarction aborted | |
| G33zz00 | Angina pectoris NOS | |
| G33z000 | Status anginosus | |
| G362.00 | Ventric septal defect/curr comp fol acut myocardal infarctn | |
| G380.00 | Postoperative transmural myocardial infarction anterior wall | |
| G39..00 | Coronary microvascular disease | |
| G384.00 | Postoperative subendocardial myocardial infarction | |
| G33z700 | Stable angina | |
| G32..00 | Old myocardial infarction | |
| G31y300 | Transient myocardial ischaemia | |
| G361.00 | Atrial septal defect/curr comp folow acut myocardal infarct | |
| G311.14 | Angina at rest | |
| G311.12 | Impending infarction | |
| G311.13 | Unstable angina | |
| G311.11 | Crescendo angina | |
| G34yz00 | Other specified chronic ischaemic heart disease NOS | |
| G311200 | Angina at rest | |
| G311000 | Myocardial infarction aborted | |
| G341111 | Mural cardiac aneurysm | |
| G340000 | Single coronary vessel disease | |
| G353.00 | Subsequent myocardial infarction of other sites | |
| G33..00 | Angina pectoris | |
| G31yz00 | Other acute and subacute ischaemic heart disease NOS | |
| G330.00 | Angina decubitus | |
| G366.00 | Thrombosis atrium,auric append&vent/curr comp foll acute MI | |
| G340100 | Double coronary vessel disease | |
| G311100 | Unstable angina | |
| G311.00 | Preinfarction syndrome | |
| G365.00 | Rupture papillary muscle/curr comp fol acute myocard infarct | |
| G33z400 | Ischaemic chest pain | |
| G310.11 | Dressler's syndrome | |
| G34y.00 | Other specified chronic ischaemic heart disease | |
| G341100 | Other cardiac wall aneurysm | |
| G3y..00 | Other specified ischaemic heart disease | |
| G360.00 | Haemopericardium/current comp folow acut myocard infarct | |
| G34y100 | Chronic myocardial ischaemia | |
| G31y.00 | Other acute and subacute ischaemic heart disease | |
| G364.00 | Ruptur chordae tendinae/curr comp fol acute myocard infarct | |
| G341000 | Ventricular cardiac aneurysm | |
| G37..00 | Cardiac syndrome X | |
| G311300 | Refractory angina | |
| G381.00 | Postoperative transmural myocardial infarction inferior wall | |
| G383.00 | Postoperative transmural myocardial infarction unspec site | |
| G38..00 | Postoperative myocardial infarction | |
| G311z00 | Preinfarction syndrome NOS | |
| G36..00 | Certain current complication follow acute myocardial infarct | |
| G330000 | Nocturnal angina | |
| G34y000 | Chronic coronary insufficiency | |
| G35..00 | Subsequent myocardial infarction | |
| G33z.00 | Angina pectoris NOS | |
| G341z00 | Aneurysm of heart NOS | |
| G31y000 | Acute coronary insufficiency | |
| G33z300 | Angina on effort | |
| G34z000 | Asymptomatic coronary heart disease | |
| G34..00 | Other chronic ischaemic heart disease | |
| G341300 | Acquired atrioventricular fistula of heart | |
| G341.00 | Aneurysm of heart | |
| G343.00 | Ischaemic cardiomyopathy | |
| G344.00 | Silent myocardial ischaemia | |
| G311400 | Worsening angina | |
| G33z200 | Syncope anginosa | |
| G340.11 | Triple vessel disease of the heart | |
| G35X.00 | Subsequent myocardial infarction of unspecified site | |
| G331.00 | Prinzmetal's angina | |
| G341200 | Aneurysm of coronary vessels | |
| G33z500 | Post infarct angina | |
| G340.12 | Coronary artery disease | |
| G38z.00 | Postoperative myocardial infarction, unspecified | |
| G31y100 | Microinfarction of heart | |
| G332.00 | Coronary artery spasm | |
| G32..11 | Healed myocardial infarction | |
| G32..12 | Personal history of myocardial infarction | |
| G351.00 | Subsequent myocardial infarction of inferior wall | |
| G33z600 | New onset angina | |
| G350.00 | Subsequent myocardial infarction of anterior wall | |
| G311500 | Acute coronary syndrome | |
| G33z100 | Stenocardia | |
| G331.11 | Variant angina pectoris | |
| G341.11 | Cardiac aneurysm | |
| G34z.00 | Other chronic ischaemic heart disease NOS | |
| G330z00 | Angina decubitus NOS | |
| G340.00 | Coronary atherosclerosis | |
| G342.00 | Atherosclerotic cardiovascular disease | |
| Gyu3000 | [X]Other forms of angina pectoris | |
| Gyu3200 | [X]Other forms of acute ischaemic heart disease | |
| Gyu3300 | [X]Other forms of chronic ischaemic heart disease | |
| Gyu3400 | [X]Acute transmural myocardial infarction of unspecif site | |
| Gyu3600 | [X]Subsequent myocardial infarction of unspecified site | |
| Gyu3.00 | [X]Ischaemic heart diseases | |
| Gyu3500 | [X]Subsequent myocardial infarction of other sites | |
| 1O1..00 | Heart failure confirmed | |
| 8H2S.00 | Admit heart failure emergency | |
| G1yz100 | Rheumatic left ventricular failure | |
| G232.00 | Hypertensive heart&renal dis wth (congestive) heart failure | |
| G234.00 | Hyperten heart&renal dis+both(congestv)heart and renal fail | |
| G58..00 | Heart failure | |
| G58..11 | Cardiac failure | |
| G580.00 | Congestive heart failure | |
| G580.11 | Congestive cardiac failure | |
| G580.12 | Right heart failure | |
| G580.13 | Right ventricular failure | |
| G580.14 | Biventricular failure | |
| G580000 | Acute congestive heart failure | |
| G580100 | Chronic congestive heart failure | |
| G580200 | Decompensated cardiac failure | |
| G580300 | Compensated cardiac failure | |
| G580400 | Congestive heart failure due to valvular disease | |
| G581.00 | Left ventricular failure | |
| G581.11 | Asthma - cardiac | |
| G581.12 | Pulmonary oedema - acute | |
| G581.13 | Impaired left ventricular function | |
| G581000 | Acute left ventricular failure | |
| G582.00 | Acute heart failure | |
| G583.00 | Heart failure with normal ejection fraction | |
| G583.11 | HFNEF - heart failure with normal ejection fraction | |
| G583.12 | Heart failure with preserved ejection fraction | |
| G584.00 | Right ventricular failure | |
| G58z.00 | Heart failure NOS | |
| G58z.11 | Weak heart | |
| G58z.12 | Cardiac failure NOS | |
| - | Peripheral angiopathic disease EC NOS | |
| - | Peripheral arterial disease | |
| - | Peripheral ischaemia | |
| - | Peripheral ischaemic vascular disease | |
| - | Peripheral vascular complications of care | |
| - | Peripheral vascular complications of care NOS | |
| - | Peripheral vascular disease monitoring | |
| - | Peripheral vascular disease monitoring first letter | |
| - | Peripheral vascular disease monitoring invitation | |
| - | Peripheral vascular disease monitoring second letter | |
| - | Peripheral vascular disease monitoring third letter | |
| - | Peripheral vascular disease NOS | |
| - | Peripheral vascular disease NOS | |
| - | Claudication | |
| **SECONDARY CARE (HOSPITAL EPISODES AND MORTALITY)** | | |
| **ICD-10 code** | **ICD-10 description** | |
| I60 | Subarachnoid haemorrhage | |
| I61 | Intracerebral haemorrhage | |
| I62 | Other nontraumatic intracranial haemorrhage | |
| I63 | Cerebral infarction | |
| I64 | Stroke, not specified as haemorrhage or infarction | |
| I69 | Sequelae of cerebrovascular disease (EXCEPT I69.8) | |
| I20 | Angina pectoris | |
| I21 | Acute myocardial infarction | |
| I22 | Subsequent myocardial infarction | |
| I23 | Certain current complications following acute myocardial infarction | |
| I24 | Other acute ischaemic heart diseases | |
| I25 | Chronic ischaemic heart disease | |
| I50 | Heart failure | |
| 150.0 | Congestive heart failure | |
| I50.9 | Heart failure, unspecified | |
| I11.0 | Hypertensive heart disease with (congestive) heart failure | |
| I13.2 | Hypertensive heart and renal disease with both (congestive) heart failure and renal failure | |
| I13.0 | Hypertensive heart and renal disease with (congestive) heart failure | |
| I73.9 | Peripheral arterial disease | |
| **Diabetes (primary care and secondary care/mortality)** | | |
| **PRIMARY CARE** | | |
| **Read Code** | **Read Code Description** | |
| 66A..00 | Diabetic monitoring | |
| 66A2.00 | Follow-up diabetic assessment | |
| 66A3.00 | Diabetic on diet only | |
| 66A4.00 | Diabetic on oral treatment | |
| 66A5.00 | Diabetic on insulin | |
| 66A6.00 | Last hypo. attack | |
| 66A7.00 | Frequency of hypo. attacks | |
| 66A7000 | Frequency of hospital treated hypoglycaemia | |
| 66A7100 | Frequency of GP or paramedic treated hypoglycaemia | |
| 66A8.00 | Has seen dietician - diabetes | |
| 66A9.00 | Understands diet - diabetes | |
| 66AA.00 | Injection sites | |
| 66Aa.00 | Diabetic diet - poor compliance | |
| 66AA.11 | Injection sites – diabetic | |
| 66Ab.00 | Diabetic foot examination | |
| 66AB.00 | Urine sugar charts | |
| 66AC.00 | Blood sugar charts | |
| 66Ac.00 | Diabetic peripheral neuropathy screening | |
| 66Ad.00 | Hypoglycaemic attack requiring 3rd party assistance | |
| 66AD.00 | Fundoscopy - diabetic check | |
| 66Ae.00 | HbA1c target | |
| 66AE.00 | Feet examination | |
| 66Ae000 | HbA1c target level - IFCC standardised | |
| 66Af.00 | Patient diabetes education review | |
| 66AF.00 | Attends out-patients | |
| 66Ag.00 | Insulin needles changed daily | |
| 66AG.00 | Diabetic drug side effects | |
| 66Ah.00 | Insulin needles changed for each injection | |
| 66AH.00 | Diabetic treatment changed | |
| 66AH000 | Conversion to insulin | |
| 66AH100 | Conversion to insulin in secondary care | |
| 66AH200 | Conversion to insulin by diabetes specialist nurse | |
| 66AH300 | Conversion to non-insulin injectable medication | |
| 66Ai.00 | Diabetic 6 month review | |
| 66AI.00 | Diabetic - good control | |
| 66Aj.00 | Insulin needles changed less than once a day | |
| 66AJ.00 | Diabetic - poor control | |
| 66AJ.11 | Unstable diabetes | |
| 66AJ000 | Chronic hyperglycaemia | |
| 66AJ100 | Brittle diabetes | |
| 66AJ200 | Loss of hypoglycaemic warning | |
| 66AJ300 | Recurrent severe hypos | |
| 66AJ400 | Hypoglycaemic warning absent | |
| 66AJz00 | Diabetic - poor control NOS | |
| 66AK.00 | Diabetic - cooperative patient | |
| 66Ak.00 | Diabetic monitoring - lower risk albumin excretion | |
| 66Al.00 | Diabetic monitoring - higher risk albumin excretion | |
| 66AL.00 | Diabetic-uncooperative patient | |
| 66Am.00 | Insulin dose changed | |
| 66AM.00 | Diabetic - follow-up default | |
| 66An.00 | Diabetes type 1 review | |
| 66AN.00 | Date diabetic treatment start | |
| 66Ao.00 | Diabetes type 2 review | |
| 66Ap.00 | Insulin treatment initiated | |
| 66AP.00 | Diabetes: practice programme | |
| 66AQ.00 | Diabetes: shared care programme | |
| 66Aq.00 | Diabetic foot screen | |
| 66AQ000 | Unsuitable for diabetes year of care programme | |
| 66AQ100 | Declined consent for diabetes year of care programme | |
| 66AR.00 | Diabetes management plan given | |
| 66Ar.00 | Insulin treatment stopped | |
| 66AS.00 | Diabetic annual review | |
| 66As.00 | Diabetic on subcutaneous treatment | |
| 66AS000 | Diabetes Year of Care annual review | |
| 66At.00 | Diabetic dietary review | |
| 66At000 | Type I diabetic dietary review | |
| 66At011 | Type 1 diabetic dietary review | |
| 66At100 | Type II diabetic dietary review | |
| 66At111 | Type 2 diabetic dietary review | |
| 66AU.00 | Diabetes care by hospital only | |
| 66Au.00 | Diabetic erectile dysfunction review | |
| 66AV.00 | Diabetic on insulin and oral treatment | |
| 66Av.00 | Diabetic assessment of erectile dysfunction | |
| 66AW.00 | Diabetic foot risk assessment | |
| 66Aw.00 | Insulin dose | |
| 66AX.00 | Diabetes: shared care in pregnancy - diabetol and obstet | |
| 66Ax.00 | Checking accuracy of blood glucose meter | |
| 66Ay.00 | Gestational diabetes mellitus annual review | |
| 66AY.00 | Diabetic diet - good compliance | |
| 66AZ.00 | Diabetic monitoring NOS | |
| 9OL..00 | Diabetes monitoring admin. | |
| 9OL..11 | Diabetes clinic administration | |
| 9OL1.00 | Attends diabetes monitoring | |
| 9OL2.00 | Refuses diabetes monitoring | |
| 9OL3.00 | Diabetes monitoring default | |
| 9OL4.00 | Diabetes monitoring 1st letter | |
| 9OL5.00 | Diabetes monitoring 2nd letter | |
| 9OL6.00 | Diabetes monitoring 3rd letter | |
| 9OL7.00 | Diabetes monitor.verbal invite | |
| 9OL8.00 | Diabetes monitor.phone invite | |
| 9OLA.00 | Diabetes monitor. check done | |
| 9OLA.11 | Diabetes monitored | |
| 9OLB.00 | Attended diabetes structured education programme | |
| 9OLC.00 | Family/carer attended diabetes structured education prog | |
| 9OLD.00 | Diabetic patient unsuitable for digital retinal photography | |
| 9OLE.00 | Attended DESMOND structured programme | |
| 9OLF.00 | Diabetes structured education programme completed | |
| 9OLG.00 | Attended XPERT diabetes structured education programme | |
| 9OLH.00 | Attended DAFNE diabetes structured education programme | |
| 9OLJ.00 | DAFNE diabetes structured education programme completed | |
| 9OLK.00 | DESMOND diabetes structured education programme completed | |
| 9OLL.00 | XPERT diabetes structured education programme completed | |
| 9OLM.00 | Diabetes structured education programme declined | |
| 9OLN.00 | Diabetes monitor invitation by SMS (short message service) | |
| 9OLP.00 |  | |
| 9OLZ.00 | Diabetes monitoring admin.NOS | |
| C10..00 | Diabetes mellitus | |
| C100.00 | Diabetes mellitus with no mention of complication | |
| C100000 | Diabetes mellitus, juvenile type, no mention of complication | |
| C100011 | Insulin dependent diabetes mellitus | |
| C100100 | Diabetes mellitus, adult onset, no mention of complication | |
| C100111 | Maturity onset diabetes | |
| C100112 | Non-insulin dependent diabetes mellitus | |
| C100z00 | Diabetes mellitus NOS with no mention of complication | |
| C101.00 | Diabetes mellitus with ketoacidosis | |
| C101000 | Diabetes mellitus, juvenile type, with ketoacidosis | |
| C101100 | Diabetes mellitus, adult onset, with ketoacidosis | |
| C101y00 | Other specified diabetes mellitus with ketoacidosis | |
| C101z00 | Diabetes mellitus NOS with ketoacidosis | |
| C102.00 | Diabetes mellitus with hyperosmolar coma | |
| C102000 | Diabetes mellitus, juvenile type, with hyperosmolar coma | |
| C102100 | Diabetes mellitus, adult onset, with hyperosmolar coma | |
| C102z00 | Diabetes mellitus NOS with hyperosmolar coma | |
| C103.00 | Diabetes mellitus with ketoacidotic coma | |
| C103000 | Diabetes mellitus, juvenile type, with ketoacidotic coma | |
| C103100 | Diabetes mellitus, adult onset, with ketoacidotic coma | |
| C103y00 | Other specified diabetes mellitus with coma | |
| C103z00 | Diabetes mellitus NOS with ketoacidotic coma | |
| C104.00 | Diabetes mellitus with renal manifestation | |
| C104.11 | Diabetic nephropathy | |
| C104000 | Diabetes mellitus, juvenile type, with renal manifestation | |
| C104100 | Diabetes mellitus, adult onset, with renal manifestation | |
| C104y00 | Other specified diabetes mellitus with renal complications | |
| C104z00 | Diabetes mellitus with nephropathy NOS | |
| C105.00 | Diabetes mellitus with ophthalmic manifestation | |
| C105000 | Diabetes mellitus, juvenile type, + ophthalmic manifestation | |
| C105100 | Diabetes mellitus, adult onset, + ophthalmic manifestation | |
| C105y00 | Other specified diabetes mellitus with ophthalmic complicatn | |
| C105z00 | Diabetes mellitus NOS with ophthalmic manifestation | |
| C106.00 | Diabetes mellitus with neurological manifestation | |
| C106.11 | Diabetic amyotrophy | |
| C106.12 | Diabetes mellitus with neuropathy | |
| C106.13 | Diabetes mellitus with polyneuropathy | |
| C106000 | Diabetes mellitus, juvenile, + neurological manifestation | |
| C106100 | Diabetes mellitus, adult onset, + neurological manifestation | |
| C106y00 | Other specified diabetes mellitus with neurological comps | |
| C106z00 | Diabetes mellitus NOS with neurological manifestation | |
| C107.00 | Diabetes mellitus with peripheral circulatory disorder | |
| C107.11 | Diabetes mellitus with gangrene | |
| C107.12 | Diabetes with gangrene | |
| C107000 | Diabetes mellitus, juvenile +peripheral circulatory disorder | |
| C107100 | Diabetes mellitus, adult, + peripheral circulatory disorder | |
| C107200 | Diabetes mellitus, adult with gangrene | |
| C107300 | IDDM with peripheral circulatory disorder | |
| C107400 | NIDDM with peripheral circulatory disorder | |
| C107z00 | Diabetes mellitus NOS with peripheral circulatory disorder | |
| C108.00 | Insulin dependent diabetes mellitus | |
| C108.11 | IDDM-Insulin dependent diabetes mellitus | |
| C108.12 | Type 1 diabetes mellitus | |
| C108.13 | Type I diabetes mellitus | |
| C108000 | Insulin-dependent diabetes mellitus with renal complications | |
| C108011 | Type I diabetes mellitus with renal complications | |
| C108012 | Type 1 diabetes mellitus with renal complications | |
| C108100 | Insulin-dependent diabetes mellitus with ophthalmic comps | |
| C108112 | Type 1 diabetes mellitus with ophthalmic complications | |
| C108200 | Insulin-dependent diabetes mellitus with neurological comps | |
| C108211 | Type I diabetes mellitus with neurological complications | |
| C108212 | Type 1 diabetes mellitus with neurological complications | |
| C108300 | Insulin dependent diabetes mellitus with multiple complicatn | |
| C108311 | Type I diabetes mellitus with multiple complications | |
| C108400 | Unstable insulin dependent diabetes mellitus | |
| C108411 | Unstable type I diabetes mellitus | |
| C108412 | Unstable type 1 diabetes mellitus | |
| C108500 | Insulin dependent diabetes mellitus with ulcer | |
| C108511 | Type I diabetes mellitus with ulcer | |
| C108512 | Type 1 diabetes mellitus with ulcer | |
| C108600 | Insulin dependent diabetes mellitus with gangrene | |
| C108700 | Insulin dependent diabetes mellitus with retinopathy | |
| C108711 | Type I diabetes mellitus with retinopathy | |
| C108712 | Type 1 diabetes mellitus with retinopathy | |
| C108800 | Insulin dependent diabetes mellitus - poor control | |
| C108811 | Type I diabetes mellitus - poor control | |
| C108812 | Type 1 diabetes mellitus - poor control | |
| C108900 | Insulin dependent diabetes maturity onset | |
| C108911 | Type I diabetes mellitus maturity onset | |
| C108912 | Type 1 diabetes mellitus maturity onset | |
| C108A00 | Insulin-dependent diabetes without complication | |
| C108A11 | Type I diabetes mellitus without complication | |
| C108A12 | Type 1 diabetes mellitus without complication | |
| C108B00 | Insulin dependent diabetes mellitus with mononeuropathy | |
| C108B11 | Type I diabetes mellitus with mononeuropathy | |
| C108C00 | Insulin dependent diabetes mellitus with polyneuropathy | |
| C108D00 | Insulin dependent diabetes mellitus with nephropathy | |
| C108D11 | Type I diabetes mellitus with nephropathy | |
| C108E00 | Insulin dependent diabetes mellitus with hypoglycaemic coma | |
| C108E11 | Type I diabetes mellitus with hypoglycaemic coma | |
| C108E12 | Type 1 diabetes mellitus with hypoglycaemic coma | |
| C108F00 | Insulin dependent diabetes mellitus with diabetic cataract | |
| C108F11 | Type I diabetes mellitus with diabetic cataract | |
| C108F12 | Type 1 diabetes mellitus with diabetic cataract | |
| C108G00 | Insulin dependent diab mell with peripheral angiopathy | |
| C108H00 | Insulin dependent diabetes mellitus with arthropathy | |
| C108H11 | Type I diabetes mellitus with arthropathy | |
| C108J00 | Insulin dependent diab mell with neuropathic arthropathy | |
| C108J11 | Type I diabetes mellitus with neuropathic arthropathy | |
| C108J12 | Type 1 diabetes mellitus with neuropathic arthropathy | |
| C108y00 | Other specified diabetes mellitus with multiple comps | |
| C108z00 | Unspecified diabetes mellitus with multiple complications | |
| C109.00 | Non-insulin dependent diabetes mellitus | |
| C109.11 | NIDDM - Non-insulin dependent diabetes mellitus | |
| C109.12 | Type 2 diabetes mellitus | |
| C109.13 | Type II diabetes mellitus | |
| C109000 | Non-insulin-dependent diabetes mellitus with renal comps | |
| C109011 | Type II diabetes mellitus with renal complications | |
| C109012 | Type 2 diabetes mellitus with renal complications | |
| C109100 | Non-insulin-dependent diabetes mellitus with ophthalm comps | |
| C109111 | Type II diabetes mellitus with ophthalmic complications | |
| C109112 | Type 2 diabetes mellitus with ophthalmic complications | |
| C109200 | Non-insulin-dependent diabetes mellitus with neuro comps | |
| C109211 | Type II diabetes mellitus with neurological complications | |
| C109212 | Type 2 diabetes mellitus with neurological complications | |
| C109300 | Non-insulin-dependent diabetes mellitus with multiple comps | |
| C109312 | Type 2 diabetes mellitus with multiple complications | |
| C109400 | Non-insulin dependent diabetes mellitus with ulcer | |
| C109411 | Type II diabetes mellitus with ulcer | |
| C109412 | Type 2 diabetes mellitus with ulcer | |
| C109500 | Non-insulin dependent diabetes mellitus with gangrene | |
| C109511 | Type II diabetes mellitus with gangrene | |
| C109512 | Type 2 diabetes mellitus with gangrene | |
| C109600 | Non-insulin-dependent diabetes mellitus with retinopathy | |
| C109611 | Type II diabetes mellitus with retinopathy | |
| C109612 | Type 2 diabetes mellitus with retinopathy | |
| C109700 | Non-insulin dependent diabetes mellitus - poor control | |
| C109711 | Type II diabetes mellitus - poor control | |
| C109712 | Type 2 diabetes mellitus - poor control | |
| C109900 | Non-insulin-dependent diabetes mellitus without complication | |
| C109911 | Type II diabetes mellitus without complication | |
| C109912 | Type 2 diabetes mellitus without complication | |
| C109A00 | Non-insulin dependent diabetes mellitus with mononeuropathy | |
| C109A11 | Type II diabetes mellitus with mononeuropathy | |
| C109B00 | Non-insulin dependent diabetes mellitus with polyneuropathy | |
| C109B11 | Type II diabetes mellitus with polyneuropathy | |
| C109B12 | Type 2 diabetes mellitus with polyneuropathy | |
| C109C00 | Non-insulin dependent diabetes mellitus with nephropathy | |
| C109C11 | Type II diabetes mellitus with nephropathy | |
| C109C12 | Type 2 diabetes mellitus with nephropathy | |
| C109D00 | Non-insulin dependent diabetes mellitus with hypoglyca coma | |
| C109D11 | Type II diabetes mellitus with hypoglycaemic coma | |
| C109D12 | Type 2 diabetes mellitus with hypoglycaemic coma | |
| C109E00 | Non-insulin depend diabetes mellitus with diabetic cataract | |
| C109E11 | Type II diabetes mellitus with diabetic cataract | |
| C109E12 | Type 2 diabetes mellitus with diabetic cataract | |
| C109F00 | Non-insulin-dependent d m with peripheral angiopath | |
| C109F11 | Type II diabetes mellitus with peripheral angiopathy | |
| C109F12 | Type 2 diabetes mellitus with peripheral angiopathy | |
| C109G00 | Non-insulin dependent diabetes mellitus with arthropathy | |
| C109G11 | Type II diabetes mellitus with arthropathy | |
| C109G12 | Type 2 diabetes mellitus with arthropathy | |
| C109H00 | Non-insulin dependent d m with neuropathic arthropathy | |
| C109H11 | Type II diabetes mellitus with neuropathic arthropathy | |
| C109H12 | Type 2 diabetes mellitus with neuropathic arthropathy | |
| C109J00 | Insulin treated Type 2 diabetes mellitus | |
| C109J11 | Insulin treated non-insulin dependent diabetes mellitus | |
| C109J12 | Insulin treated Type II diabetes mellitus | |
| C109K00 | Hyperosmolar non-ketotic state in type 2 diabetes mellitus | |
| C10A.00 | Malnutrition-related diabetes mellitus | |
| C10A000 | Malnutrition-related diabetes mellitus with coma | |
| C10A100 | Malnutrition-related diabetes mellitus with ketoacidosis | |
| C10A500 | Malnutritn-relat diabetes melitus wth periph circul complctn | |
| C10B.00 | Diabetes mellitus induced by steroids | |
| C10B000 | Steroid induced diabetes mellitus without complication | |
| C10C.00 | Diabetes mellitus autosomal dominant | |
| C10C.11 | Maturity onset diabetes in youth | |
| C10C.12 | Maturity onset diabetes in youth type 1 | |
| C10D.00 | Diabetes mellitus autosomal dominant type 2 | |
| C10D.11 | Maturity onset diabetes in youth type 2 | |
| C10E.00 | Type 1 diabetes mellitus | |
| C10E.11 | Type I diabetes mellitus | |
| C10E.12 | Insulin dependent diabetes mellitus | |
| C10E000 | Type 1 diabetes mellitus with renal complications | |
| C10E011 | Type I diabetes mellitus with renal complications | |
| C10E012 | Insulin-dependent diabetes mellitus with renal complications | |
| C10E100 | Type 1 diabetes mellitus with ophthalmic complications | |
| C10E111 | Type I diabetes mellitus with ophthalmic complications | |
| C10E112 | Insulin-dependent diabetes mellitus with ophthalmic comps | |
| C10E200 | Type 1 diabetes mellitus with neurological complications | |
| C10E212 | Insulin-dependent diabetes mellitus with neurological comps | |
| C10E300 | Type 1 diabetes mellitus with multiple complications | |
| C10E311 | Type I diabetes mellitus with multiple complications | |
| C10E312 | Insulin dependent diabetes mellitus with multiple complicat | |
| C10E400 | Unstable type 1 diabetes mellitus | |
| C10E411 | Unstable type I diabetes mellitus | |
| C10E412 | Unstable insulin dependent diabetes mellitus | |
| C10E500 | Type 1 diabetes mellitus with ulcer | |
| C10E511 | Type I diabetes mellitus with ulcer | |
| C10E512 | Insulin dependent diabetes mellitus with ulcer | |
| C10E600 | Type 1 diabetes mellitus with gangrene | |
| C10E611 | Type I diabetes mellitus with gangrene | |
| C10E612 | Insulin dependent diabetes mellitus with gangrene | |
| C10E700 | Type 1 diabetes mellitus with retinopathy | |
| C10E711 | Type I diabetes mellitus with retinopathy | |
| C10E712 | Insulin dependent diabetes mellitus with retinopathy | |
| C10E800 | Type 1 diabetes mellitus - poor control | |
| C10E811 | Type I diabetes mellitus - poor control | |
| C10E812 | Insulin dependent diabetes mellitus - poor control | |
| C10E900 | Type 1 diabetes mellitus maturity onset | |
| C10E911 | Type I diabetes mellitus maturity onset | |
| C10E912 | Insulin dependent diabetes maturity onset | |
| C10EA00 | Type 1 diabetes mellitus without complication | |
| C10EA11 | Type I diabetes mellitus without complication | |
| C10EA12 | Insulin-dependent diabetes without complication | |
| C10EB00 | Type 1 diabetes mellitus with mononeuropathy | |
| C10EC00 | Type 1 diabetes mellitus with polyneuropathy | |
| C10EC11 | Type I diabetes mellitus with polyneuropathy | |
| C10EC12 | Insulin dependent diabetes mellitus with polyneuropathy | |
| C10ED00 | Type 1 diabetes mellitus with nephropathy | |
| C10ED12 | Insulin dependent diabetes mellitus with nephropathy | |
| C10EE00 | Type 1 diabetes mellitus with hypoglycaemic coma | |
| C10EE12 | Insulin dependent diabetes mellitus with hypoglycaemic coma | |
| C10EF00 | Type 1 diabetes mellitus with diabetic cataract | |
| C10EF12 | Insulin dependent diabetes mellitus with diabetic cataract | |
| C10EG00 | Type 1 diabetes mellitus with peripheral angiopathy | |
| C10EH00 | Type 1 diabetes mellitus with arthropathy | |
| C10EJ00 | Type 1 diabetes mellitus with neuropathic arthropathy | |
| C10EK00 | Type 1 diabetes mellitus with persistent proteinuria | |
| C10EL00 | Type 1 diabetes mellitus with persistent microalbuminuria | |
| C10EL11 | Type I diabetes mellitus with persistent microalbuminuria | |
| C10EM00 | Type 1 diabetes mellitus with ketoacidosis | |
| C10EM11 | Type I diabetes mellitus with ketoacidosis | |
| C10EN00 | Type 1 diabetes mellitus with ketoacidotic coma | |
| C10EN11 | Type I diabetes mellitus with ketoacidotic coma | |
| C10EP00 | Type 1 diabetes mellitus with exudative maculopathy | |
| C10EP11 | Type I diabetes mellitus with exudative maculopathy | |
| C10EQ00 | Type 1 diabetes mellitus with gastroparesis | |
| C10EQ11 | Type I diabetes mellitus with gastroparesis | |
| C10ER00 | Latent autoimmune diabetes mellitus in adult | |
| C10F.00 | Type 2 diabetes mellitus | |
| C10F.11 | Type II diabetes mellitus | |
| C10F000 | Type 2 diabetes mellitus with renal complications | |
| C10F011 | Type II diabetes mellitus with renal complications | |
| C10F100 | Type 2 diabetes mellitus with ophthalmic complications | |
| C10F111 | Type II diabetes mellitus with ophthalmic complications | |
| C10F200 | Type 2 diabetes mellitus with neurological complications | |
| C10F211 | Type II diabetes mellitus with neurological complications | |
| C10F300 | Type 2 diabetes mellitus with multiple complications | |
| C10F311 | Type II diabetes mellitus with multiple complications | |
| C10F400 | Type 2 diabetes mellitus with ulcer | |
| C10F411 | Type II diabetes mellitus with ulcer | |
| C10F500 | Type 2 diabetes mellitus with gangrene | |
| C10F511 | Type II diabetes mellitus with gangrene | |
| C10F600 | Type 2 diabetes mellitus with retinopathy | |
| C10F611 | Type II diabetes mellitus with retinopathy | |
| C10F700 | Type 2 diabetes mellitus - poor control | |
| C10F711 | Type II diabetes mellitus - poor control | |
| C10F811 | Metabolic syndrome X | |
| C10F900 | Type 2 diabetes mellitus without complication | |
| C10F911 | Type II diabetes mellitus without complication | |
| C10FA00 | Type 2 diabetes mellitus with mononeuropathy | |
| C10FA11 | Type II diabetes mellitus with mononeuropathy | |
| C10FB00 | Type 2 diabetes mellitus with polyneuropathy | |
| C10FB11 | Type II diabetes mellitus with polyneuropathy | |
| C10FC00 | Type 2 diabetes mellitus with nephropathy | |
| C10FC11 | Type II diabetes mellitus with nephropathy | |
| C10FD00 | Type 2 diabetes mellitus with hypoglycaemic coma | |
| C10FD11 | Type II diabetes mellitus with hypoglycaemic coma | |
| C10FE00 | Type 2 diabetes mellitus with diabetic cataract | |
| C10FE11 | Type II diabetes mellitus with diabetic cataract | |
| C10FF00 | Type 2 diabetes mellitus with peripheral angiopathy | |
| C10FF11 | Type II diabetes mellitus with peripheral angiopathy | |
| C10FG00 | Type 2 diabetes mellitus with arthropathy | |
| C10FG11 | Type II diabetes mellitus with arthropathy | |
| C10FH00 | Type 2 diabetes mellitus with neuropathic arthropathy | |
| C10FH11 | Type II diabetes mellitus with neuropathic arthropathy | |
| C10FJ00 | Insulin treated Type 2 diabetes mellitus | |
| C10FJ11 | Insulin treated Type II diabetes mellitus | |
| C10FK00 | Hyperosmolar non-ketotic state in type 2 diabetes mellitus | |
| C10FK11 | Hyperosmolar non-ketotic state in type II diabetes mellitus | |
| C10FL00 | Type 2 diabetes mellitus with persistent proteinuria | |
| C10FL11 | Type II diabetes mellitus with persistent proteinuria | |
| C10FM00 | Type 2 diabetes mellitus with persistent microalbuminuria | |
| C10FM11 | Type II diabetes mellitus with persistent microalbuminuria | |
| C10FN00 | Type 2 diabetes mellitus with ketoacidosis | |
| C10FN11 | Type II diabetes mellitus with ketoacidosis | |
| C10FP00 | Type 2 diabetes mellitus with ketoacidotic coma | |
| C10FP11 | Type II diabetes mellitus with ketoacidotic coma | |
| C10FQ00 | Type 2 diabetes mellitus with exudative maculopathy | |
| C10FQ11 | Type II diabetes mellitus with exudative maculopathy | |
| C10FR00 | Type 2 diabetes mellitus with gastroparesis | |
| C10FS00 | Maternally inherited diabetes mellitus | |
| C10G.00 | Secondary pancreatic diabetes mellitus | |
| C10G000 | Secondary pancreatic diabetes mellitus without complication | |
| C10H.00 | Diabetes mellitus induced by non-steroid drugs | |
| C10H000 | DM induced by non-steroid drugs without complication | |
| C10K.00 | Type A insulin resistance | |
| C10K000 | Type A insulin resistance without complication | |
| C10M.00 | Lipoatrophic diabetes mellitus | |
| C10N.00 | Secondary diabetes mellitus | |
| C10N000 | Secondary diabetes mellitus without complication | |
| C10N100 | Cystic fibrosis related diabetes mellitus | |
| C10P.00 | Diabetes mellitus in remission | |
| C10P000 | Type I diabetes mellitus in remission | |
| C10P011 | Type 1 diabetes mellitus in remission | |
| C10P100 | Type II diabetes mellitus in remission | |
| C10P111 | Type 2 diabetes mellitus in remission | |
| C10y.00 | Diabetes mellitus with other specified manifestation | |
| C10y000 | Diabetes mellitus, juvenile, + other specified manifestation | |
| C10y100 | Diabetes mellitus, adult, + other specified manifestation | |
| C10yy00 | Other specified diabetes mellitus with other spec comps | |
| C10yz00 | Diabetes mellitus NOS with other specified manifestation | |
| C10z.00 | Diabetes mellitus with unspecified complication | |
| C10z000 | Diabetes mellitus, juvenile type, + unspecified complication | |
| C10z100 | Diabetes mellitus, adult onset, + unspecified complication | |
| C10zy00 | Other specified diabetes mellitus with unspecified comps | |
| C10zz00 | Diabetes mellitus NOS with unspecified complication | |
| C350011 | Bronzed diabetes | |
| Cyu2.00 | [X]Diabetes mellitus | |
| Cyu2000 | [X]Other specified diabetes mellitus | |
| Cyu2300 | [X]Unspecified diabetes mellitus with renal complications | |
| F171100 | Autonomic neuropathy due to diabetes | |
| F345000 | Diabetic mononeuritis multiplex | |
| F35z000 | Diabetic mononeuritis NOS | |
| F372.00 | Polyneuropathy in diabetes | |
| F372.11 | Diabetic polyneuropathy | |
| F372.12 | Diabetic neuropathy | |
| F372000 | Acute painful diabetic neuropathy | |
| F372100 | Chronic painful diabetic neuropathy | |
| F372200 | Asymptomatic diabetic neuropathy | |
| F381300 | Myasthenic syndrome due to diabetic amyotrophy | |
| F381311 | Diabetic amyotrophy | |
| F3y0.00 | Diabetic mononeuropathy | |
| F420.00 | Diabetic retinopathy | |
| F420000 | Background diabetic retinopathy | |
| F420100 | Proliferative diabetic retinopathy | |
| F420200 | Preproliferative diabetic retinopathy | |
| F420300 | Advanced diabetic maculopathy | |
| F420400 | Diabetic maculopathy | |
| F420500 | Advanced diabetic retinal disease | |
| F420600 | Non proliferative diabetic retinopathy | |
| F420700 | High risk proliferative diabetic retinopathy | |
| F420800 | High risk non proliferative diabetic retinopathy | |
| F420z00 | Diabetic retinopathy NOS | |
| F440700 | Diabetic iritis | |
| F464000 | Diabetic cataract | |
| G73y000 | Diabetic peripheral angiopathy | |
| K01x100 | Nephrotic syndrome in diabetes mellitus | |
| K081.00 | Nephrogenic diabetes insipidus | |
| K08yA00 | Proteinuric diabetic nephropathy | |
| K08yA11 | Clinical diabetic nephropathy | |
| K27y700 | Erectile dysfunction due to diabetes mellitus | |
| Kyu0300 | [X]Glomerular disorders in diabetes mellitus | |
| L180500 | Pre-existing diabetes mellitus, insulin-dependent | |
| L180600 | Pre-existing diabetes mellitus, non-insulin-dependent | |
| L180700 | Pre-existing malnutrition-related diabetes mellitus | |
| L180X00 | Pre-existing diabetes mellitus, unspecified | |
| M037200 | Cellulitis in diabetic foot | |
| M271000 | Ischaemic ulcer diabetic foot | |
| M271100 | Neuropathic diabetic ulcer - foot | |
| M271200 | Mixed diabetic ulcer - foot | |
| N030000 | Diabetic cheiroarthropathy | |
| N030011 | Diabetic cheiropathy | |
| N030100 | Diabetic Charcot arthropathy | |
| R054200 | [D]Gangrene of toe in diabetic | |
| R054300 | [D]Widespread diabetic foot gangrene | |
| ZC2C800 | Dietary advice for diabetes mellitus | |
| ZC2C900 | Dietary advice for type I diabetes | |
| ZC2C911 | Diet advice for insulin-dependent diabetes | |
| ZC2CA00 | Dietary advice for type II diabetes | |
| ZRbH.00 | Perceived control of insulin-dependent diabetes | |
| ZV65312 | [V]Dietary counselling in diabetes mellitus | |
| **SECONDARY CARE (HOSPITAL EPISODES AND MORTALITY)** | | |
| **ICD-10 code** | **ICD-10 description** | |
| E10 | Type 1 diabetes mellitus | |
| E11 | Type 2 diabetes mellitus | |
| E12 | Malnutrition-related diabetes mellitus | |
| E13 | Other specified diabetes mellitus | |
| E14 | Unspecified diabetes mellitus | |
| **Falls/Fractures (Hospital episodes and mortality only)** | |  |
| **SECONDARY CARE (HOSPITAL EPISODES AND MORTALITY)** | |  |
| **ICD-10 code** | **ICD-10 description** |  |
| W00 - W19 | Falls |  |
| S02.X | Fractures of skull and facial bones |  |
| S12.X | Fracture of neck |  |
| S22.X | Fracture of rib(s), sternum and thoracic spine |  |
| S32.X | Fracture of lumbar spine and pelvis |  |
| S42.X | Fracture of shoulder and upper arm |  |
| S52.X | Fracture of forearm |  |
| S62.X | Fracture of wrist and hand |  |
| S72.X | Fracture of hip and thigh |  |
| S82.X | Fracture of knee and lower leg |  |
| S92.X | Fracture of ankle and foot |  |

**Table S8. Read codes and ICD-10 codes (where relevant) for Covariates**

| **Smoking** | | | |  |
| --- | --- | --- | --- | --- |
| **Read Code** | | **Read Code Description** | **Category** |  |
| 9S10.00 | | White British | White |  |
| 9S13.00 | | White Scottish | White |  |
| 9S14.00 | | Other white British ethnic group | White |  |
| 9i00.00 | | White British - ethnic category 2001 census | White |  |
| 9i20.00 | | English - ethnic category 2001 census | White |  |
| 9i21.00 | | Scottish - ethnic category 2001 census | White |  |
| 9i22.00 | | Welsh - ethnic category 2001 census | White |  |
| 9i23.00 | | Cornish - ethnic category 2001 census | White |  |
| 9S11.00 | | White Irish | White |  |
| 9SA9.00 | | Irish (NMO) | White |  |
| 9SI..00 | | Irish traveller | White |  |
| 9i1..00 | | Irish - ethnic category 2001 census | White |  |
| 9i10.00 | | White Irish - ethnic category 2001 census | White |  |
| 9i24.00 | | Northern Irish - ethnic category 2001 census | White |  |
| 9i2C.00 | | Irish Traveller - ethnic category 2001 census | White |  |
| 9S12.00 | | Other white ethnic group | White |  |
| 9SAA.00 | | Greek/Greek Cypriot (NMO) | White |  |
| 9SAA.11 | | Greek (NMO) | White |  |
| 9SAA.12 | | Greek Cypriot (NMO) | White |  |
| 9SAB.00 | | Turkish/Turkish Cypriot (NMO) | White |  |
| 9SAB.11 | | Turkish (NMO) | White |  |
| 9SAB.12 | | Turkish Cypriot (NMO) | White |  |
| 9SAC.00 | | Other European (NMO) | White |  |
| 9T1..00 | | New Zealand ethnic groups | White |  |
| 9T1Y.00 | | Other New Zealand ethnic group | White |  |
| 9T1Z.00 | | New Zealand ethnic group NOS | White |  |
| 9i2..00 | | Other White background - ethnic category 2001 census | White |  |
| 9i26.00 | | Cypriot (part not stated) - ethnic category 2001 census | White |  |
| 9i27.00 | | Greek - ethnic category 2001 census | White |  |
| 9i28.00 | | Greek Cypriot - ethnic category 2001 census | White |  |
| 9i29.00 | | Turkish - ethnic category 2001 census | White |  |
| 9i2A.00 | | Turkish Cypriot - ethnic category 2001 census | White |  |
| 9i2B.00 | | Italian - ethnic category 2001 census | White |  |
| 9i2D.00 | | Traveller - ethnic category 2001 census | White |  |
| 9i2E.00 | | Gypsy/Romany - ethnic category 2001 census | White |  |
| 9i2F.00 | | Polish - ethnic category 2001 census | White |  |
| 9i2G.00 | | Baltic Estonian/Latvian/Lithuanian - ethn categ 2001 census | White |  |
| 9i2H.00 | | Commonwealth (Russian) Indep States - ethn categ 2001 census | White |  |
| 9i2J.00 | | Kosovan - ethnic category 2001 census | White |  |
| 9i2K.00 | | Albanian - ethnic category 2001 census | White |  |
| 9i2L.00 | | Bosnian - ethnic category 2001 census | White |  |
| 9i2M.00 | | Croatian - ethnic category 2001 census | White |  |
| 9i2N.00 | | Serbian - ethnic category 2001 census | White |  |
| 9i2P.00 | | Other republics former Yugoslavia - ethnic categ 2001 census | White |  |
| 9i2T.00 | | Other White or White unspecified ethnic category 2001 census | White |  |
| 9S1..00 | | White | White |  |
| 9SB3.00 | | Other ethnic, mixed white orig | White |  |
| 9i25.00 | | Ulster Scots - ethnic category 2001 census | White |  |
| 9i2Q.00 | | Mixed Irish and other White - ethnic category 2001 census | White |  |
| 9i2R.00 | | Oth White European/European unsp/Mixed European 2001 census | White |  |
| 9i2S.00 | | Other mixed White - ethnic category 2001 census | White |  |
| 9SB5.00 | | Black Caribbean and White | Mixed |  |
| 9SB6.00 | | Black African and White | Mixed |  |
| 9i3..00 | | White and Black Caribbean - ethnic category 2001 census | Mixed |  |
| 9i4..00 | | White and Black African - ethnic category 2001 census | Mixed |  |
| 9SB2.00 | | Other ethnic, Asian/White orig | Mixed |  |
| 9i5..00 | | White and Asian - ethnic category 2001 census | Mixed |  |
| 9S45.00 | | Black E Afric Asia/Indo-Caribb | Mixed |  |
| 9S45.11 | | Black East African Asian | Mixed |  |
| 9S45.12 | | Black Indo-Caribbean | Mixed |  |
| 9S46.00 | | Black Indian sub-continent | Mixed |  |
| 9S47.00 | | Black - other Asian | Mixed |  |
| 9S52.00 | | Other Black - Black/Asian orig | Mixed |  |
| 9SA6.00 | | E Afric Asian/Indo-Carib (NMO) | Mixed |  |
| 9i60.00 | | Black and Asian - ethnic category 2001 census | Mixed |  |
| 9i61.00 | | Black and Chinese - ethnic category 2001 census | Mixed |  |
| 9iA7.00 | | Caribbean Asian - ethnic category 2001 census | Mixed |  |
| 9SB..00 | | Other ethnic, mixed origin | Mixed |  |
| 9SB4.00 | | Other ethnic, other mixed orig | Mixed |  |
| 9i6..00 | | Other Mixed background - ethnic category 2001 census | Mixed |  |
| 9i63.00 | | Chinese and White - ethnic category 2001 census | Mixed |  |
| 9i64.00 | | Asian and Chinese - ethnic category 2001 census | Mixed |  |
| 9i65.00 | | Other Mixed or Mixed unspecified ethnic category 2001 census | Mixed |  |
| 9S51.00 | | Other Black - Black/White orig | Mixed |  |
| 9SB1.00 | | Other ethnic, Black/White orig | Mixed |  |
| 9i62.00 | | Black and White - ethnic category 2001 census | Mixed |  |
| 9S6..00 | | Indian | S. Asian |  |
| 9SA7.00 | | Indian sub-continent (NMO) | S. Asian |  |
| 9i7..00 | | Indian or British Indian - ethnic category 2001 census | S. Asian |  |
| 9iA1.00 | | Punjabi - ethnic category 2001 census | S. Asian |  |
| 9S7..00 | | Pakistani | S. Asian |  |
| 9i8..00 | | Pakistani or British Pakistani - ethnic category 2001 census | S. Asian |  |
| 9iA2.00 | | Kashmiri - ethnic category 2001 census | S. Asian |  |
| 9S8..00 | | Bangladeshi | S. Asian |  |
| 9i9..00 | | Bangladeshi or British Bangladeshi - ethn categ 2001 census | S. Asian |  |
| 9SA6.11 | | East African Asian (NMO) | S. Asian |  |
| 9SA8.00 | | Other Asian (NMO) | S. Asian |  |
| 9SH..00 | | Other Asian ethnic group | S. Asian |  |
| 9iA..00 | | Other Asian background - ethnic category 2001 census | S. Asian |  |
| 9iA3.00 | | East African Asian - ethnic category 2001 census | S. Asian |  |
| 9iA8.00 | | British Asian - ethnic category 2001 census | S. Asian |  |
| 9iA9.00 | | Mixed Asian - ethnic category 2001 census | S. Asian |  |
| 9iAA.00 | | Other Asian or Asian unspecified ethnic category 2001 census | S. Asian |  |
| 9S2..00 | | Black Caribbean | Black |  |
| 9S42.00 | | Black Caribbean/W.I./Guyana | Black |  |
| 9S42.11 | | Black Caribbean | Black |  |
| 9S42.12 | | Black West Indian | Black |  |
| 9S42.13 | | Black Guyana | Black |  |
| 9SA3.00 | | Caribbean I./W.I./Guyana (NMO) | Black |  |
| 9SA3.11 | | Caribbean Island (NMO) | Black |  |
| 9SA3.12 | | West Indian (NMO) | Black |  |
| 9SA3.13 | | Guyana (NMO) | Black |  |
| 9iB..00 | | Caribbean - ethnic category 2001 census | Black |  |
| 9S3..00 | | Black African | Black |  |
| 9S44.00 | | Black - other African country | Black |  |
| 9SA5.00 | | Other African countries (NMO) | Black |  |
| 9iC..00 | | African - ethnic category 2001 census | Black |  |
| 9iD0.00 | | Somali - ethnic category 2001 census | Black |  |
| 9iD1.00 | | Nigerian - ethnic category 2001 census | Black |  |
| 9S4..00 | | Black, other, non-mixed origin | Black |  |
| 9S41.00 | | Black British | Black |  |
| 9S43.00 | | Black N African/Arab/Iranian | Black |  |
| 9S43.11 | | Black North African | Black |  |
| 9S43.12 | | Black Arab | Black |  |
| 9S43.13 | | Black Iranian | Black |  |
| 9S48.00 | | Black Black - other | Black |  |
| 9S5..00 | | Black - other, mixed | Black |  |
| 9SG..00 | | Other black ethnic group | Black |  |
| 9iD..00 | | Other Black background - ethnic category 2001 census | Black |  |
| 9iD2.00 | | Black British - ethnic category 2001 census | Black |  |
| 9iD3.00 | | Mixed Black - ethnic category 2001 census | Black |  |
| 9iD4.00 | | Other Black or Black unspecified ethnic category 2001 census | Black |  |
| 9S9..00 | | Chinese | Other |  |
| 9iE..00 | | Chinese - ethnic category 2001 census | Other |  |
| 9SA..00 | | Other ethnic non-mixed (NMO) | Other |  |
| 9SA1.00 | | Brit. ethnic minor. spec.(NMO) | Other |  |
| 9SA2.00 | | Brit. ethnic minor. unsp (NMO) | Other |  |
| 9SA4.00 | | N African Arab/Iranian (NMO) | Other |  |
| 9SA4.11 | | North African Arab (NMO) | Other |  |
| 9SA4.12 | | Iranian (NMO) | Other |  |
| 9SAD.00 | | Other ethnic NEC (NMO) | Other |  |
| 9SC..00 | | Vietnamese | Other |  |
| 9SJ..00 | | Other ethnic group | Other |  |
| 9T1A.00 | | Other Pacific ethnic group | Other |  |
| 9iA4.00 | | Sri Lankan - ethnic category 2001 census | Other |  |
| 9iA5.00 | | Tamil - ethnic category 2001 census | Other |  |
| 9iA6.00 | | Sinhalese - ethnic category 2001 census | Other |  |
| 9iF..00 | | Other - ethnic category 2001 census | Other |  |
| 9iF0.00 | | Vietnamese - ethnic category 2001 census | Other |  |
| 9iF1.00 | | Japanese - ethnic category 2001 census | Other |  |
| 9iF2.00 | | Filipino - ethnic category 2001 census | Other |  |
| 9iF3.00 | | Malaysian - ethnic category 2001 census | Other |  |
| 9iF4.00 | | Buddhist - ethnic category 2001 census | Other |  |
| 9iF5.00 | | Hindu - ethnic category 2001 census | Other |  |
| 9iF6.00 | | Jewish - ethnic category 2001 census | Other |  |
| 9iF7.00 | | Muslim - ethnic category 2001 census | Other |  |
| 9iF8.00 | | Sikh - ethnic category 2001 census | Other |  |
| 9iF9.00 | | Arab - ethnic category 2001 census | Other |  |
| 9iFA.00 | | North African - ethnic category 2001 census | Other |  |
| 9iFB.00 | | Mid East (excl Israeli, Iranian & Arab) - eth cat 2001 cens | Other |  |
| 9iFC.00 | | Israeli - ethnic category 2001 census | Other |  |
| 9iFD.00 | | Iranian - ethnic category 2001 census | Other |  |
| 9iFE.00 | | Kurdish - ethnic category 2001 census | Other |  |
| 9iFF.00 | | Moroccan - ethnic category 2001 census | Other |  |
| 9iFG.00 | | Latin American - ethnic category 2001 census | Other |  |
| 9iFH.00 | | South and Central American - ethnic category 2001 census | Other |  |
| 9iFJ.00 | | Mauritian/Seychellois/Maldivian/St Helena eth cat 2001census | Other |  |
| 9iFK.00 | | Any other group - ethnic category 2001 census | Other |  |
| 916E.00 | | Patient ethnicity unknown | Not known |  |
| 9S...00 | | Ethnic groups (1991 census) | Not known |  |
| 9SD..00 | | Ethnic group not given - patient refused | Not known |  |
| 9SE..00 | | Ethnic group not recorded | Not known |  |
| 9SZ..00 | | Ethnic groups (census) NOS | Not known |  |
| 9T...00 | | Ethnicity and other related nationality data | Not known |  |
| 9i...00 | | Ethnic category - 2001 census | Not known |  |
| 9i0..00 | | British or mixed British - ethnic category 2001 census | Not known |  |
| 9iG..00 | | Ethnic category not stated - 2001 census | Not known |  |
| 9t0..00 | | Ethnic category - 2011 census England and Wales | Not known |  |
| 9t00.00 | | White:Eng/Welsh/Scot/NI/Brit - England and Wales 2011 census | White |  |
| 9t01.00 | | White: Irish - England and Wales ethnic category 2011 census | White |  |
| 9t02.00 | | White: Gypsy/Irish Traveller - Eng+Wales eth cat 2011 census | White |  |
| 9t03.00 | | White: other White backgrd- Eng+Wales ethnic cat 2011 census | White |  |
| 9t04.00 | | Mixed: White+Black Caribbean - Eng+Wales eth cat 2011 census | Mixed |  |
| 9t05.00 | | Mixed: White+Black African - Eng+Wales eth cat 2011 census | Mixed |  |
| 9t06.00 | | Mixed: White+Asian - Eng+Wales ethnic category 2011 census | Mixed |  |
| 9t08.00 | | Asian/Asian Brit: Indian - Eng+Wales ethnic cat 2011 census | S. Asian |  |
| 9t09.00 | | Asian/Asian British:Pakistani- Eng+Wales eth cat 2011 census | S. Asian |  |
| 9t0A.00 | | Asian/Asian Brit: Bangladeshi- Eng+Wales eth cat 2011 census | S. Asian |  |
| 9t0B.00 | | Asian/Asian Brit: Chinese - Eng+Wales ethnic cat 2011 census | Other |  |
| 9t0C.00 | | Asian/Asian Brit: other Asian- Eng+Wales eth cat 2011 census | S. Asian |  |
| 9t0D.00 | | Black/African/Carib/Black Brit: African- Eng+Wales 2011 cens | Black |  |
| 9t0E.00 | | Black/African/Caribbn/Black Brit: Caribbean - Eng+Wales 2011 | Black |  |
| 9t0F.00 | | Black/Afr/Carib/Black Brit: other Black- Eng+Wales 2011 cens | Black |  |
| 9t0G.00 | | Other ethnic group: Arab - Eng+Wales ethnic cat 2011 census | Other |  |
| 9t0H.00 | | Other ethnic: any other grp - Eng+Wales eth cat 2011 census | Other |  |
| 9t12.00 | | Mixed: White and Black Caribbean - NI ethnic cat 2011 census | Mixed |  |
| 9t13.00 | | Mixed: White and Black African - NI ethnic cat 2011 census | Mixed |  |
| 9t14.00 | | Mixed: White and Asian - NI ethnic category 2011 census | Mixed |  |
| 9t15.00 | | Mixed: other Mixed/multiple ethnic backgrd - NI 2011 census | Mixed |  |
| 9t16.00 | | Asian or Asian British: Indian - NI ethnic cat 2011 census | S. Asian |  |
| 9t17.00 | | Asian/Asian British: Pakistani - NI ethnic cat 2011 census | S. Asian |  |
| 9t18.00 | | Asian/Asian British: Bangladeshi - NI ethnic cat 2011 census | S. Asian |  |
| 9t19.00 | | Asian/Asian British: Chinese - NI ethnic cat 2011 census | Other |  |
| 9t1E.00 | | Other ethnic group: Arab - NI ethnic category 2011 census | Other |  |
| 9t1F.00 | | Other ethnic group: any other grp- NI ethnic cat 2011 census | Other |  |
| 9t2..00 | | Ethnic category - 2011 census Scotland | Not specified |  |
| 9t20.00 | | White: Scottish - Scotland ethnic category 2011 census | White |  |
| 9t21.00 | | White: other British - Scotland ethnic category 2011 census | White |  |
| 9t22.00 | | White: Irish - Scotland ethnic category 2011 census | White |  |
| 9t24.00 | | White: Polish - Scotland ethnic category 2011 census | White |  |
| 9t25.00 | | White: other White ethnic grp- Scotland ethnic cat 2011 cens | White |  |
| 9t26.00 | | Mixed/multiple ethnic grps: any- Scot ethnic cat 2011 census | Mixed |  |
| 9t27.00 | | Asian: Pakistani/Pakistani Scot/Pakistani Brit- Scot 2011 | S.Asian |  |
| 9t28.00 | | Asian: Indian, Indian Scot/Indian Brit- Scotland 2011 census | S.Asian |  |
| 9t29.00 | | Bangladeshi, Bangladeshi Scot or Bangladeshi Brit- Scot 2011 | S.Asian |  |
| 9t2A.00 | | Asian: Chinese - Scotland ethnic category 2011 census | Other |  |
| 9t2B.00 | | Asian: other Asian group - Scotland ethnic cat 2011 census | S.Asian |  |
| 9t2C.00 | | African: African/African Scot/African Brit - Scotland 2011 | Black |  |
| 9t2D.00 | | African: any other African - Scotland ethnic cat 2011 census | Black |  |
| 9t2G.00 | | Carib/Black: any other Black/Caribbean grp - Scotland 2011 | Black |  |
| 9t2H.00 | | Other ethnic grp: Arab/Arab Scot/Arab British- Scotland 2011 | Other |  |
| 9t2J.00 | | Other ethnic grp: any other ethnic grp- Scotland 2011 census | Other |  |
| **Smoking** | | | |  |
| **Read Code** | | **Read Code Description** | **Category** |  |
| 8CdB.00 | | Stop smoking service opportunity signposted | current smoker |  |
| 13p0.00 | | Negotiated date for cessation of smoking | current smoker |  |
| 8B31G00 | | Varenicline smoking cessation therapy offered | current smoker |  |
| ZV6D800 | | [V]Tobacco abuse counselling | current smoker |  |
| 1V08.00 | | Smokes drugs in cigarette form | current smoker |  |
| ZV4K000 | | [V]Tobacco use | current smoker |  |
| 137P.11 | | Smoker | current smoker |  |
| 137C.00 | | Keeps trying to stop smoking | current smoker |  |
| 137g.00 | | Cigarette pack-years | current smoker |  |
| 137Q.11 | | Smoking restarted | current smoker |  |
| 137..00 | | Tobacco consumption | current smoker |  |
| 137M.00 | | Rolls own cigarettes | current smoker |  |
| 8CAL.00 | | Smoking cessation advice | current smoker |  |
| 137G.00 | | Trying to give up smoking | current smoker |  |
| 137Z.00 | | Tobacco consumption NOS | current smoker |  |
| 9NS0200 | | Referral for smoking cessation service offered | current smoker |  |
| E251200 | | Tobacco dependence, episodic | current smoker |  |
| 137f.00 | | Reason for restarting smoking | current smoker |  |
| 1376 | | Very heavy smoker - 40+cigs/d | current smoker |  |
| 137h.00 | | Minutes from waking to first tobacco consumption | current smoker |  |
| E251z00 | | Tobacco dependence NOS | current smoker |  |
| 1372.11 | | Occasional smoker | current smoker |  |
| 8HkQ.00 | | Referral to NHS stop smoking service | current smoker |  |
| 8IEo.00 | | Referral to smoking cessation service declined | current smoker |  |
| 137a.00 | | Pipe tobacco consumption | current smoker |  |
| 8HTK.00 | | Referral to stop-smoking clinic | current smoker |  |
| 137W.00 | | Chews tobacco | current smoker |  |
| E251000 | | Tobacco dependence, unspecified | current smoker |  |
| 137V.00 | | Smoking reduced | current smoker |  |
| 137m.00 | | Failed attempt to stop smoking | current smoker |  |
| 1375 | | Heavy smoker - 20-39 cigs/day | current smoker |  |
| 9ko..11 | | Current smoker annual review | current smoker |  |
| 137H.00 | | Pipe smoker | current smoker |  |
| 137o.00 | | Waterpipe tobacco consumption | current smoker |  |
| 137Y.00 | | Cigar consumption | current smoker |  |
| 9ko..00 | | Current smoker annual review - enhanced services admin | current smoker |  |
| 1373 | | Light smoker - 1-9 cigs/day | current smoker |  |
| 137E.00 | | Tobacco consumption unknown | current smoker |  |
| 137c.00 | | Thinking about stopping smoking | current smoker |  |
| 137Q.00 | | Smoking started | current smoker |  |
| 1374 | | Moderate smoker - 10-19 cigs/d | current smoker |  |
| 137X.00 | | Cigarette consumption | current smoker |  |
| 137..11 | | Smoker - amount smoked | current smoker |  |
| 137J.00 | | Cigar smoker | current smoker |  |
| 137R.00 | | Current smoker | current smoker |  |
| E251.00 | | Tobacco dependence | current smoker |  |
| 137d.00 | | Not interested in stopping smoking | current smoker |  |
| 8T08.00 | | Referral to smoking cessation service | current smoker |  |
| 137D.00 | | Admitted tobacco cons untrue ? | current smoker |  |
| 137b.00 | | Ready to stop smoking | current smoker |  |
| 8IAj.00 | | Smoking cessation advice declined | current smoker |  |
| 1372 | | Trivial smoker - < 1 cig/day | current smoker |  |
| 137P.00 | | Cigarette smoker | current smoker |  |
| 8H7i.00 | | Referral to smoking cessation advisor | current smoker |  |
| 137e.00 | | Smoking restarted | current smoker |  |
| E251100 | | Tobacco dependence, continuous | current smoker |  |
| 137K.00 | | Stopped smoking | ex smoker |  |
| 137K000 | | Recently stopped smoking | ex smoker |  |
| 1379 | | Ex-moderate smoker (10-19/day) | ex smoker |  |
| 137T.00 | | Date ceased smoking | ex smoker |  |
| 137j.00 | | Ex-cigarette smoker | ex smoker |  |
| 9km..11 | | Ex-smoker annual review | ex smoker |  |
| 1378 | | Ex-light smoker (1-9/day) | ex smoker |  |
| 137l.00 | | Ex roll-up cigarette smoker | ex smoker |  |
| 137B.00 | | Ex-very heavy smoker (40+/day) | ex smoker |  |
| 137O.00 | | Ex cigar smoker | ex smoker |  |
| 137S.00 | | Ex smoker | ex smoker |  |
| 137N.00 | | Ex pipe smoker | ex smoker |  |
| 1377 | | Ex-trivial smoker (<1/day) | ex smoker |  |
| 137F.00 | | Ex-smoker - amount unknown | ex smoker |  |
| 9km..00 | | Ex-smoker annual review - enhanced services administration | ex smoker |  |
| 137A.00 | | Ex-heavy smoker (20-39/day) | ex smoker |  |
| 137i.00 | | Ex-tobacco chewer | ex smoker |  |
| 137U.00 | | Not a passive smoker | never smoker |  |
| 1371.11 | | Non-smoker | never smoker |  |
| 9kn..00 | | Non-smoker annual review - enhanced services administration | never smoker |  |
| 137L.00 | | Current non-smoker | never smoker |  |
| 9kn..11 | | Non-smoker annual review | never smoker |  |
| 1371 | | Never smoked tobacco | never smoker |  |
| 137I.00 | | Passive smoker | never smoker |  |
| **Alcohol** | | | |  |
| **Read Code** | | **Read Code Description** | **Category** |  |
| 1361 | | Teetotaller | No |  |
| 1361.11 | | Non drinker alcohol | No |  |
| 1361.12 | | Non-drinker alcohol | No |  |
| 1362 | | Trivial drinker - <1u/day | Yes |  |
| 1362.11 | | Drinks rarely | Yes |  |
| 1362.12 | | Drinks occasionally | Yes |  |
| 1363 | | Light drinker - 1-2u/day | Yes |  |
| 1364 | | Moderate drinker - 3-6u/day | Yes |  |
| 1365 | | Heavy drinker - 7-9u/day | Yes |  |
| 1366 | | Very heavy drinker - >9u/day | Yes |  |
| 1367 | | Stopped drinking alcohol | Ex |  |
| 1369 | | Suspect alcohol abuse - denied | Yes |  |
| 136A.00 | | Ex-trivial drinker (<1u/day) | Ex |  |
| 136B.00 | | Ex-light drinker - (1-2u/day) | Ex |  |
| 136C.00 | | Ex-moderate drinker - (3-6u/d) | Ex |  |
| 136D.00 | | Ex-heavy drinker - (7-9u/day) | Ex |  |
| 136E.00 | | Ex-very heavy drinker-(>9u/d) | Ex |  |
| 136F.00 | | Spirit drinker | Yes |  |
| 136G.00 | | Beer drinker | Yes |  |
| 136H.00 | | Drinks beer and spirits | Yes |  |
| 136I.00 | | Drinks wine | Yes |  |
| 136J.00 | | Social drinker | Yes |  |
| 136K.00 | | Alcohol intake above recommended sensible limits | Yes |  |
| 136L.00 | | Alcohol intake within recommended sensible limits | Yes |  |
| 136M.00 | | Current non drinker | No |  |
| 136N.00 | | Light drinker | Yes |  |
| 136O.00 | | Moderate drinker | Yes |  |
| 136P.00 | | Heavy drinker | Yes |  |
| 136Q.00 | | Very heavy drinker | Yes |  |
| 136R.00 | | Binge drinker | Yes |  |
| 136S.00 | | Hazardous alcohol use | Yes |  |
| 136T.00 | | Harmful alcohol use | Yes |  |
| 136W.00 | | Alcohol misuse | Yes |  |
| 136Z.00 | | Alcohol consumption NOS | Yes |  |
| 136a.00 | | Increasing risk drinking | Yes |  |
| 136c.00 | | Higher risk drinking | Yes |  |
| 136d.00 | | Lower risk drinking | Yes |  |
| 13ZY.00 | | Disqualified from driving due to excess alcohol | Yes |  |
| 1B1c.00 | | Alcohol induced hallucinations | Yes |  |
| 1D19.00 | | Pain in lymph nodes after alcohol consumption | Yes |  |
| 2577 | | O/E - breath - alcohol smell | Yes |  |
| 2577.11 | | O/E - alcoholic breath | Yes |  |
| 38Dz.00 | | Severity of alcohol dependence questionnaire | Yes |  |
| 38Dz.11 | | SADQ - Severity of alcohol dependence questionnaire | Yes |  |
| 66e0.00 | | alcohol abuse monitoring | Yes |  |
| 7P22100 | | Delivery of rehabilitation for alcohol addiction | Yes |  |
| 8BA8.00 | | alcohol detoxification | Yes |  |
| 8BAu.00 | | Alcohol harm reduction programme | Yes |  |
| 8CAv.00 | | advised to contact primary care alcohol worker | Yes |  |
| 8CE1.00 | | Alcohol leaflet given | Yes |  |
| 8G32.00 | | Aversion therapy - alcoholism | Yes |  |
| 8H35.00 | | Admitted to alcohol detoxification centre | Yes |  |
| 8H7p.00 | | Referral to community alcohol team | Yes |  |
| 8HkG.00 | | Referral to specialist alcohol treatment service | Yes |  |
| 8HkJ.00 | | Referral to alcohol brief intervention service | Yes |  |
| 8IAF.00 | | brief intervention for excessive alcohol consumptn declined | Yes |  |
| 8IAJ.00 | | declined referral to specialist alcohol treatment service | Yes |  |
| 8IAt.00 | | Extended intervention for excessive alcohol consumption declined | Yes |  |
| 8W2..00 | | Refer to MH services deferred until alcohol misuse resolved | Yes |  |
| 9NJz.00 | | In-house alcohol detoxification | Yes |  |
| 9NN2.00 | | Under care of community alcohol team | Yes |  |
| 9Nz9.00 | | Emergency dept attendanc related to personl alcohl consumptn | Yes |  |
| 9NzA.00 | | Hospital attendance related to personal alcohol consumption | Yes |  |
| 9k1..00 | | Alcohol misuse - enhanced services administration | Yes |  |
| 9k11.00 | | Alcohol consumption counselling | Yes |  |
| 9k12.00 | | Alcohol misuse - enhanced service completed | Yes |  |
| 9k14.00 | | Alcohol counselling by other agencies | Yes |  |
| 9k15.00 | | Alcohol screen - AUDIT completed | Yes |  |
| 9k18.00 | | Alcohol screen - AUDIT PC completed | Yes |  |
| 9k1B.00 | | Extended intervention for excessive alcohol consumption completed | Yes |  |
| C150500 | | Alcohol-induced pseudo-Cushing's syndrome | Yes |  |
| E01..00 | | Alcoholic psychoses | Yes |  |
| E010.00 | | Alcohol withdrawal delirium | Yes |  |
| E011.00 | | Alcohol amnestic syndrome | Yes |  |
| E011000 | | Korsakov's alcoholic psychosis | Yes |  |
| E011100 | | Korsakov's alcoholic psychosis with peripheral neuritis | Yes |  |
| E011200 | | Wernicke-Korsakov syndrome | Yes |  |
| E011z00 | | Alcohol amnestic syndrome NOS | Yes |  |
| E012.00 | | Other alcoholic dementia | Yes |  |
| E012.11 | | Alcoholic dementia NOS | Yes |  |
| E012000 | | Chronic alcoholic brain syndrome | Yes |  |
| E013.00 | | Alcohol withdrawal hallucinosis | Yes |  |
| E014.00 | | Pathological alcohol intoxication | Yes |  |
| E014.11 | | Drunkenness - pathological | Yes |  |
| E015.00 | | Alcoholic paranoia | Yes |  |
| E01y.00 | | Other alcoholic psychosis | Yes |  |
| E01y000 | | Alcohol withdrawal syndrome | Yes |  |
| E01yz00 | | Other alcoholic psychosis NOS | Yes |  |
| E01z.00 | | Alcoholic psychosis NOS | Yes |  |
| E23..00 | | Alcohol dependence syndrome | Yes |  |
| E23..11 | | Alcoholism | Yes |  |
| E23..12 | | Alcohol problem drinking | Yes |  |
| E230.00 | | Acute alcoholic intoxication in alcoholism | Yes |  |
| E230.11 | | Alcohol dependence with acute alcoholic intoxication | Yes |  |
| E230000 | | Acute alcoholic intoxication, unspecified, in alcoholism | Yes |  |
| E230100 | | Continuous acute alcoholic intoxication in alcoholism | Yes |  |
| E230200 | | Episodic acute alcoholic intoxication in alcoholism | Yes |  |
| E230300 | | Acute alcoholic intoxication in remission, in alcoholism | Ex |  |
| E230z00 | | Acute alcoholic intoxication in alcoholism NOS | Yes |  |
| E231.00 | | Chronic alcoholism | Yes |  |
| E231000 | | Unspecified chronic alcoholism | Yes |  |
| E231100 | | Continuous chronic alcoholism | Yes |  |
| E231200 | | Episodic chronic alcoholism | Yes |  |
| E231300 | | Chronic alcoholism in remission | Ex |  |
| E231z00 | | Chronic alcoholism NOS | Yes |  |
| E23z.00 | | Alcohol dependence syndrome NOS | Yes |  |
| E247.11 | | Absinthe addiction | Yes |  |
| E250.00 | | Nondependent alcohol abuse | Yes |  |
| E250.11 | | Drunkenness NOS | Yes |  |
| E250.12 | | Hangover (alcohol) | Yes |  |
| E250.13 | | Inebriety NOS | Yes |  |
| E250.14 | | Intoxication - alcohol | Yes |  |
| E250000 | | Nondependent alcohol abuse, unspecified | Yes |  |
| E250100 | | Nondependent alcohol abuse, continuous | Yes |  |
| E250200 | | Nondependent alcohol abuse, episodic | Yes |  |
| E250300 | | Nondependent alcohol abuse in remission | Ex |  |
| E250z00 | | Nondependent alcohol abuse NOS | Yes |  |
| Eu10.00 | | [X]Mental and behavioural disorders due to use of alcohol | Yes |  |
| Eu10000 | | [X]Mental & behav dis due to use alcohol: acute intoxication | Yes |  |
| Eu10011 | | [X]Acute alcoholic drunkenness | Yes |  |
| Eu10100 | | [X]Mental and behav dis due to use of alcohol: harmful use | Yes |  |
| Eu10200 | | [X]Mental and behav dis due to use alcohol: dependence syndr | Yes |  |
| Eu10211 | | [X]Alcohol addiction | Yes |  |
| Eu10212 | | [X]Chronic alcoholism | Yes |  |
| Eu10213 | | [X]Dipsomania | Yes |  |
| Eu10300 | | [X]Mental and behav dis due to use alcohol: withdrawal state | Yes |  |
| Eu10400 | | [X]Men & behav dis due alcohl: withdrawl state with delirium | Yes |  |
| Eu10411 | | [X]Delirium tremens, alcohol induced | Yes |  |
| Eu10500 | | [X]Mental & behav dis due to use alcohol: psychotic disorder | Yes |  |
| Eu10511 | | [X]Alcoholic hallucinosis | Yes |  |
| Eu10512 | | [X]Alcoholic jealousy | Yes |  |
| Eu10513 | | [X]Alcoholic paranoia | Yes |  |
| Eu10514 | | [X]Alcoholic psychosis NOS | Yes |  |
| Eu10600 | | [X]Mental and behav dis due to use alcohol: amnesic syndrome | Yes |  |
| Eu10611 | | [X]Korsakov's psychosis, alcohol induced | Yes |  |
| Eu10700 | | [X]Mental and behavioural disorders due to use of alcohol: residual and late-onset psychotic disorde | Yes |  |
| Eu10711 | | [X]Alcoholic dementia NOS | Yes |  |
| Eu10712 | | [X]Chronic alcoholic brain syndrome | Yes |  |
| Eu10800 | | [X]Alcohol withdrawal-induced seizure | Yes |  |
| Eu10y00 | | [X]Men & behav dis due to use alcohol: oth men & behav dis | Yes |  |
| Eu10z00 | | [X]Ment & behav dis due use alcohol: unsp ment & behav dis | Yes |  |
| F11x000 | | Cerebral degeneration due to alcoholism | Yes |  |
| F11x011 | | Alcoholic encephalopathy | Yes |  |
| F144000 | | Cerebellar ataxia due to alcoholism | Yes |  |
| F25B.00 | | Alcohol-induced epilepsy | Yes |  |
| F375.00 | | Alcoholic polyneuropathy | Yes |  |
| F394100 | | Alcoholic myopathy | Yes |  |
| G555.00 | | Alcoholic cardiomyopathy | Yes |  |
| G852300 | | Oesophageal varices in alcoholic cirrhosis of the liver | Yes |  |
| J153.00 | | Alcoholic gastritis | Yes |  |
| J610.00 | | Alcoholic fatty liver | Yes |  |
| J611.00 | | Acute alcoholic hepatitis | Yes |  |
| J612.00 | | Alcoholic cirrhosis of liver | Yes |  |
| J612000 | | Alcoholic fibrosis and sclerosis of liver | Yes |  |
| J613.00 | | Alcoholic liver damage unspecified | Yes |  |
| J613000 | | Alcoholic hepatic failure | Yes |  |
| J617.00 | | Alcoholic hepatitis | Yes |  |
| J617000 | | Chronic alcoholic hepatitis | Yes |  |
| J670800 | | Alcohol-induced acute pancreatitis | Yes |  |
| J671000 | | Alcohol-induced chronic pancreatitis | Yes |  |
| L255300 | | Maternal care for (suspected) damage to fetus from alcohol | Yes |  |
| R103.00 | | [D]Alcohol blood level excessive | Yes |  |
| SM0..00 | | Alcohol causing toxic effect | Yes |  |
| SM00.00 | | Ethyl alcohol causing toxic effect | Yes |  |
| SM00100 | | denatured alcohol causing toxic effect | Yes |  |
| SM00z00 | | Ethyl alcohol causing toxic effect NOS | Yes |  |
| SM01.00 | | Methyl alcohol causing toxic effect | Yes |  |
| SM0z.00 | | Alcohol causing toxic effect NOS | Yes |  |
| T900.00 | | Accidental poisoning by alcoholic beverages | Yes |  |
| U60H311 | | [X] Adverse reaction to alcohol deterrents | Yes |  |
| U80..00 | | [X]Evidence of alcohl involv determin by blood alcohl level | Yes |  |
| U800.00 | | [X]Eviden of alcohl involv blood alcohl level <20 mg/100 ml | Yes |  |
| U801.00 | | [X]Eviden of alcohl involv blood alcohl level 20-39mg/100ml | Yes |  |
| U802.00 | | [X]Eviden of alcohl involv blood alcohl level 40-59mg/100ml | Yes |  |
| U803.00 | | [X]Eviden of alcohl involv blood alcohl level 60-79mg/100ml | Yes |  |
| U804.00 | | [X]Eviden of alcohl involv blood alcohl level 80-99mg/100ml | Yes |  |
| U805.00 | | [X]Eviden of alcoh involv blood alcoh level 100-119mg/100ml | Yes |  |
| U806.00 | | [X]Eviden of alcoh involv blood alcoh level 120-199mg/100ml | Yes |  |
| U807.00 | | [X]Eviden of alcoh involv blood alcoh level 200-239mg/100ml | Yes |  |
| U808.00 | | [X]Eviden alcoh involv blood alcoh level 240mg/100ml or more | Yes |  |
| U80z.00 | | [X]Evid alcoh invol detrm by pres alcoh in bld levl not spec | Yes |  |
| U81..00 | | [X]Evid of alcohol involv determind by level of intoxication | Yes |  |
| U810.00 | | [X]Evid alcoh invol determ by levl of intox mild alcoh intox | Yes |  |
| U811.00 | | [X]Evid alcoh invol determ by level of intox mod alcoh intox | Yes |  |
| U812.00 | | [X]Evid alcoh invol determ by level of intox sev alcoh intox | Yes |  |
| U813.00 | | [X]Evid alcoh invl determ by levl intox very sev alcoh intox | Yes |  |
| U814.00 | | [X]Evid alch invl detrm by levl intox alch invl not oth spec | Yes |  |
| Z191.00 | | Alcohol detoxification | Yes |  |
| Z191100 | | alcohol withdrawal regime | Yes |  |
| Z191200 | | Planned reduction of alcohol consumption | Yes |  |
| Z191211 | | Alcohol reduction programme | Yes |  |
| Z191400 | | Self-monitoring of alcohol intake | Yes |  |
| Z4B1.00 | | Alcoholism counselling | Yes |  |
| ZC22200 | | Advice to change alcoholic drink intake | Yes |  |
| ZC2H.00 | | Advice to change alcohol intake | Yes |  |
| ZRk6.00 | | Severity of alcohol dependence questionnaire | Yes |  |
| ZRk6.11 | | SADQ - Severity of alcohol dependence questionnaire | Yes |  |
| ZRk9.00 | | Short alcohol dependence data | Yes |  |
| ZRk9.11 | | SADD - Short alcohol dependence data | Yes |  |
| ZV11311 | | [V]Problems related to lifestyle alcohol use | Yes |  |
| ZV4KC00 | | [V] Alcohol use | Yes |  |
| ZV57A00 | | [V]Alcohol rehabilitation | Yes |  |
| ZV6D600 | | [V]Alcohol abuse counselling and surveillance | Yes |  |
| ZV79100 | | [V]Screening for alcoholism | Yes |  |
| **Chronic Kidney Disease** | | | |  |
| **PRIMARY CARE** | | | |  |
| **Read Code** | | **Read Code Description** | |  |
| 7L1A011 | | Thomas intravascular shunt for dialysis | |  |
| 7L1A000 | | Renal dialysis | |  |
| 7L1A100 | | Peritoneal dialysis | |  |
| 7L1A200 | | Haemodialysis NEC | |  |
| 7L1A300 | | Haemofiltration | |  |
| 7L1A400 | | Automated peritoneal dialysis | |  |
| 7L1A500 | | Continuous ambulatory peritoneal dialysis | |  |
| 7L1A600 | | Peritoneal dialysis NEC | |  |
| 7B00111 | | Allotransplantation of kidney from live donor | |  |
| 7B00y00 | | Other specified transplantation of kidney | |  |
| 7B00300 | | Allotransplantation of kidney from cadaver, heart-beating | |  |
| 7B00400 | | Allotransplantation kidney from cadaver, heart non-beating | |  |
| 7B00211 | | Allotransplantation of kidney from cadaver | |  |
| 7B00212 | | Cadaveric renal transplant | |  |
| 7B00600 | | Xenograft renal transplant | |  |
| 7B00500 | | Allotransplantation of kidney from cadaver NEC | |  |
| 7B00.00 | | Transplantation of kidney | |  |
| 7B00z00 | | Transplantation of kidney NOS | |  |
| 7B00100 | | Transplantation of kidney from live donor | |  |
| 7B00200 | | Transplantation of kidney from cadaver | |  |
| 7B00000 | | Autotransplant of kidney | |  |
| ZV42000 | | [V]Kidney transplanted | |  |
| K05..00 | | Chronic renal failure | |  |
| K011.00 | | Nephrotic syndrome with membranous glomerulonephritis | |  |
| K01x100 | | Nephrotic syndrome in diabetes mellitus | |  |
| K01..00 | | Nephrotic syndrome | |  |
| K100.00 | | Chronic pyelonephritis | |  |
| K02y200 | | Chronic focal glomerulonephritis | |  |
| ZV42000 | | [V]Kidney transplanted | |  |
| K050.00 | | End stage renal failure | |  |
| K02..00 | | Chronic glomerulonephritis | |  |
| K0D..00 | | End-stage renal disease | |  |
| K010.00 | | Nephrotic syndrome with proliferative glomerulonephritis | |  |
| K05..11 | | Chronic uraemia | |  |
| K02..11 | | Nephritis - chronic | |  |
| K021.00 | | Chronic membranous glomerulonephritis | |  |
| SP08300 | | Kidney transplant failure and rejection | |  |
| K02..12 | | Nephropathy - chronic | |  |
| 1Z13.00 | | Chronic kidney disease stage 4 | |  |
| 1Z12.00 | | Chronic kidney disease stage 3 | |  |
| 1Z14.00 | | Chronic kidney disease stage 5 | |  |
| K02z.00 | | Chronic glomerulonephritis NOS | |  |
| 8L50.00 | | Renal transplant planned | |  |
| K01B.00 | | Nephrotic syndrome, diffuse crescentic glomerulonephritis | |  |
| TB00111 | | Renal transplant with complication, without blame | |  |
| K016.00 | | Nephrotic syndrome, diffuse membranous glomerulonephritis | |  |
| K017.00 | | Nephrotic syn difus mesangial prolifertiv glomerulonephritis | |  |
| K019.00 | | Nephrotic syn,diffuse mesangiocapillary glomerulonephritis | |  |
| K01x411 | | Lupus nephritis | |  |
| K015.00 | | Nephrotic syndrome, focal and segmental glomerular lesions | |  |
| K014.00 | | Nephrotic syndrome, minor glomerular abnormality | |  |
| K100300 | | Chronic pyonephrosis | |  |
| 7B06300 | | Exploration of renal transplant | |  |
| K01z.00 | | Nephrotic syndrome NOS | |  |
| K013.00 | | Nephrotic syndrome with minimal change glomerulonephritis | |  |
| K020.00 | | Chronic proliferative glomerulonephritis | |  |
| K01x111 | | Kimmelstiel - Wilson disease | |  |
| K01x400 | | Nephrotic syndrome in systemic lupus erythematosus | |  |
| K01x000 | | Nephrotic syndrome in amyloidosis | |  |
| K100z00 | | Chronic pyelonephritis NOS | |  |
| K100500 | | Chronic obstructive pyelonephritis | |  |
| K018.00 | | Nephrotic syn,difus endocapilary proliftv glomerulonephritis | |  |
| K05..12 | | End stage renal failure | |  |
| TB00100 | | Kidney transplant with complication, without blame | |  |
| K01A.00 | | Nephrotic syndrome, dense deposit disease | |  |
| K100100 | | Chronic pyelonephritis with medullary necrosis | |  |
| K013.12 | | Steroid sensitive nephrotic syndrome | |  |
| K01x300 | | Nephrotic syndrome in polyarteritis nodosa | |  |
| K02y.00 | | Other chronic glomerulonephritis | |  |
| K022.00 | | Chronic membranoproliferative glomerulonephritis | |  |
| K02yz00 | | Other chronic glomerulonephritis NOS | |  |
| K023.00 | | Chronic rapidly progressive glomerulonephritis | |  |
| K02y300 | | Chronic diffuse glomerulonephritis | |  |
| K01w000 | | Finnish nephrosis syndrome | |  |
| K01y.00 | | Nephrotic syndrome with other pathological kidney lesions | |  |
| 1Z1B.00 | | Chronic kidney disease stage 3 with proteinuria | |  |
| 1Z15.00 | | Chronic kidney disease stage 3A | |  |
| 1Z1H.00 | | Chronic kidney disease stage 4 with proteinuria | |  |
| 1Z1C.00 | | Chronic kidney disease stage 3 without proteinuria | |  |
| 1Z1E.00 | | Chronic kidney disease stage 3A without proteinuria | |  |
| 1Z1G.00 | | Chronic kidney disease stage 3B without proteinuria | |  |
| 1Z1F.00 | | Chronic kidney disease stage 3B with proteinuria | |  |
| 1Z16.00 | | Chronic kidney disease stage 3B | |  |
| 1Z1L.00 | | Chronic kidney disease stage 5 without proteinuria | |  |
| 1Z1J.00 | | Chronic kidney disease stage 4 without proteinuria | |  |
| 1Z1D.00 | | Chronic kidney disease stage 3A with proteinuria | |  |
| 1Z1K.00 | | Chronic kidney disease stage 5 with proteinuria | |  |
| K02y000 | | Chronic glomerulonephritis + diseases EC | |  |
| K100000 | | Chronic pyelonephritis without medullary necrosis | |  |
| K012.00 | | Nephrotic syndrome+membranoproliferative glomerulonephritis | |  |
| **SECONDARY CARE (HOSPITAL EPISODES)** | | | |  |
| **ICD-10 code** | | **ICD-10 description** | |  |
| D63.1 | | Anaemia in chronic kidney disease | |  |
| E08.2 | | Diabetes mellitus due to underlying condition with kidney complications | |  |
| E10.2 | | Type 1 diabetes mellitus with kidney complications | |  |
| E11.2 | | Type 2 diabetes mellitus with kidney complications | |  |
| E12.2 | | Malnutrition-related diabetes mellitus with kidney complications | |  |
| E13.2 | | Other specified diabetes mellitus with kidney complications | |  |
| E14.2 | | Unspecified diabetes with kidney complications | |  |
| I12 – I13.9 | | Hypertensive renal disease | |  |
| N02-N08.8 | | Recurrent of persistent haematuria | |  |
| N03.X | | Chronic nephritic syndrome | |  |
| N04.X | | Nephrotic syndrome | |  |
| N05.X | | Unspecified nephritic syndrome | |  |
| N06.X | | Isolated proteinuria with specified morphological lesion | |  |
| N07.X | | Hereditary nephropathy, not elsewhere classified | |  |
| N08.X | | Glomerular disorders in diseases classified elsewhere | |  |
| N15.0 | | Balkan Nephropathy | |  |
| N18.X | | Chronic kidney disease | |  |
| Q61.X | | Cystic kidney disease | |  |
| Q62.X | | Congenital obstructive defects of renal pelvis and congenital malformations of ureter | |  |
| Z94.0 | | Kidney transplant status | |  |
| **Cancer** | | | |  |
| **PRIMARY CARE** | | | |  |
| Read Code | | Read Code Description | |  |
| Read Code | | Read Code Description | |  |
| B0...00 | | Malignant neoplasm of lip, oral cavity and pharynx | |  |
| B0...11 | | Carcinoma of lip, oral cavity and pharynx | |  |
| B00..00 | | Malignant neoplasm of lip | |  |
| B00..11 | | Carcinoma of lip | |  |
| B000.00 | | Malignant neoplasm of upper lip, vermilion border | |  |
| B000000 | | Malignant neoplasm of upper lip, external | |  |
| B000100 | | Malignant neoplasm of upper lip, lipstick area | |  |
| B000z00 | | Malignant neoplasm of upper lip, vermilion border NOS | |  |
| B001.00 | | Malignant neoplasm of lower lip, vermilion border | |  |
| B001000 | | Malignant neoplasm of lower lip, external | |  |
| B001100 | | Malignant neoplasm of lower lip, lipstick area | |  |
| B001z00 | | Malignant neoplasm of lower lip, vermilion border NOS | |  |
| B002.00 | | Malignant neoplasm of upper lip, inner aspect | |  |
| B002000 | | Malignant neoplasm of upper lip, buccal aspect | |  |
| B002100 | | Malignant neoplasm of upper lip, frenulum | |  |
| B002200 | | Malignant neoplasm of upper lip, mucosa | |  |
| B002300 | | Malignant neoplasm of upper lip, oral aspect | |  |
| B002z00 | | Malignant neoplasm of upper lip, inner aspect NOS | |  |
| B003.00 | | Malignant neoplasm of lower lip, inner aspect | |  |
| B003000 | | Malignant neoplasm of lower lip, buccal aspect | |  |
| B003100 | | Malignant neoplasm of lower lip, frenulum | |  |
| B003200 | | Malignant neoplasm of lower lip, mucosa | |  |
| B003300 | | Malignant neoplasm of lower lip, oral aspect | |  |
| B003z00 | | Malignant neoplasm of lower lip, inner aspect NOS | |  |
| B004.00 | | Malignant neoplasm of lip unspecified, inner aspect | |  |
| B004000 | | Malignant neoplasm of lip unspecified, buccal aspect | |  |
| B004200 | | Malignant neoplasm of lip unspecified, mucosa | |  |
| B004300 | | Malignant neoplasm of lip, oral aspect | |  |
| B005.00 | | Malignant neoplasm of commissure of lip | |  |
| B006.00 | | Malignant neoplasm of overlapping lesion of lip | |  |
| B007.00 | | Malignant neoplasm of lip, unspecified | |  |
| B00z000 | | Malignant neoplasm of lip, unspecified, external | |  |
| B00z100 | | Malignant neoplasm of lip, unspecified, lipstick area | |  |
| B00zz00 | | Malignant neoplasm of lip, vermilion border NOS | |  |
| B01..00 | | Malignant neoplasm of tongue | |  |
| B010.00 | | Malignant neoplasm of base of tongue | |  |
| B010.11 | | Malignant neoplasm of posterior third of tongue | |  |
| B010000 | | Malignant neoplasm of base of tongue dorsal surface | |  |
| B010z00 | | Malignant neoplasm of fixed part of tongue NOS | |  |
| B011.00 | | Malignant neoplasm of dorsal surface of tongue | |  |
| B011100 | | Malignant neoplasm of midline of tongue | |  |
| B011z00 | | Malignant neoplasm of dorsum of tongue NOS | |  |
| B012.00 | | Malignant neoplasm of tongue, tip and lateral border | |  |
| B013.00 | | Malignant neoplasm of ventral surface of tongue | |  |
| B013000 | | Malignant neoplasm of anterior 2/3 of tongue ventral surface | |  |
| B013100 | | Malignant neoplasm of frenulum linguae | |  |
| B013z00 | | Malignant neoplasm of ventral tongue surface NOS | |  |
| B014.00 | | Malignant neoplasm of anterior 2/3 of tongue unspecified | |  |
| B015.00 | | Malignant neoplasm of tongue, junctional zone | |  |
| B016.00 | | Malignant neoplasm of lingual tonsil | |  |
| B017.00 | | Malignant overlapping lesion of tongue | |  |
| B01y.00 | | Malignant neoplasm of other sites of tongue | |  |
| B01z.00 | | Malignant neoplasm of tongue NOS | |  |
| B02..00 | | Malignant neoplasm of major salivary glands | |  |
| B020.00 | | Malignant neoplasm of parotid gland | |  |
| B021.00 | | Malignant neoplasm of submandibular gland | |  |
| B022.00 | | Malignant neoplasm of sublingual gland | |  |
| B02y.00 | | Malignant neoplasm of other major salivary glands | |  |
| B02z.00 | | Malignant neoplasm of major salivary gland NOS | |  |
| B03..00 | | Malignant neoplasm of gum | |  |
| B030.00 | | Malignant neoplasm of upper gum | |  |
| B031.00 | | Malignant neoplasm of lower gum | |  |
| B03y.00 | | Malignant neoplasm of other sites of gum | |  |
| B03z.00 | | Malignant neoplasm of gum NOS | |  |
| B04..00 | | Malignant neoplasm of floor of mouth | |  |
| B040.00 | | Malignant neoplasm of anterior portion of floor of mouth | |  |
| B041.00 | | Malignant neoplasm of lateral portion of floor of mouth | |  |
| B042.00 | | Malignant neoplasm, overlapping lesion of floor of mouth | |  |
| B04y.00 | | Malignant neoplasm of other sites of floor of mouth | |  |
| B04z.00 | | Malignant neoplasm of floor of mouth NOS | |  |
| B05..00 | | Malignant neoplasm of other and unspecified parts of mouth | |  |
| B050.00 | | Malignant neoplasm of cheek mucosa | |  |
| B050.11 | | Malignant neoplasm of buccal mucosa | |  |
| B051.00 | | Malignant neoplasm of vestibule of mouth | |  |
| B051000 | | Malignant neoplasm of upper buccal sulcus | |  |
| B051100 | | Malignant neoplasm of lower buccal sulcus | |  |
| B052.00 | | Malignant neoplasm of hard palate | |  |
| B053.00 | | Malignant neoplasm of soft palate | |  |
| B054.00 | | Malignant neoplasm of uvula | |  |
| B055.00 | | Malignant neoplasm of palate unspecified | |  |
| B055000 | | Malignant neoplasm of junction of hard and soft palate | |  |
| B055100 | | Malignant neoplasm of roof of mouth | |  |
| B055z00 | | Malignant neoplasm of palate NOS | |  |
| B056.00 | | Malignant neoplasm of retromolar area | |  |
| B057.00 | | Overlapping lesion of other and unspecified parts of mouth | |  |
| B05y.00 | | Malignant neoplasm of other specified mouth parts | |  |
| B05z.00 | | Malignant neoplasm of mouth NOS | |  |
| B05z000 | | Kaposi's sarcoma of palate | |  |
| B06..00 | | Malignant neoplasm of oropharynx | |  |
| B060.00 | | Malignant neoplasm of tonsil | |  |
| B060000 | | Malignant neoplasm of faucial tonsil | |  |
| B060100 | | Malignant neoplasm of palatine tonsil | |  |
| B060200 | | Malignant neoplasm of overlapping lesion of tonsil | |  |
| B060z00 | | Malignant neoplasm tonsil NOS | |  |
| B061.00 | | Malignant neoplasm of tonsillar fossa | |  |
| B062.00 | | Malignant neoplasm of tonsillar pillar | |  |
| B062000 | | Malignant neoplasm of faucial pillar | |  |
| B062100 | | Malignant neoplasm of glossopalatine fold | |  |
| B062200 | | Malignant neoplasm of palatoglossal arch | |  |
| B062300 | | Malignant neoplasm of palatopharyngeal arch | |  |
| B062z00 | | Malignant neoplasm of tonsillar fossa NOS | |  |
| B063.00 | | Malignant neoplasm of vallecula | |  |
| B064.00 | | Malignant neoplasm of anterior epiglottis | |  |
| B064000 | | Malignant neoplasm of epiglottis, free border | |  |
| B064100 | | Malignant neoplasm of glossoepiglottic fold | |  |
| B064z00 | | Malignant neoplasm of anterior epiglottis NOS | |  |
| B065.00 | | Malignant neoplasm of junctional region of epiglottis | |  |
| B066.00 | | Malignant neoplasm of lateral wall of oropharynx | |  |
| B067.00 | | Malignant neoplasm of posterior wall of oropharynx | |  |
| B06y.00 | | Malignant neoplasm of oropharynx, other specified sites | |  |
| B06yz00 | | Malignant neoplasm of other specified site of oropharynx NOS | |  |
| B06z.00 | | Malignant neoplasm of oropharynx NOS | |  |
| B07..00 | | Malignant neoplasm of nasopharynx | |  |
| B070.00 | | Malignant neoplasm of roof of nasopharynx | |  |
| B071.00 | | Malignant neoplasm of posterior wall of nasopharynx | |  |
| B071000 | | Malignant neoplasm of adenoid | |  |
| B071100 | | Malignant neoplasm of pharyngeal tonsil | |  |
| B071z00 | | Malignant neoplasm of posterior wall of nasopharynx NOS | |  |
| B072.00 | | Malignant neoplasm of lateral wall of nasopharynx | |  |
| B072000 | | Malignant neoplasm of pharyngeal recess | |  |
| B072z00 | | Malignant neoplasm of lateral wall of nasopharynx NOS | |  |
| B073.00 | | Malignant neoplasm of anterior wall of nasopharynx | |  |
| B073100 | | Malignant neoplasm of nasopharyngeal soft palate surface | |  |
| B073200 | | Malignant neoplasm posterior margin nasal septum and choanae | |  |
| B073z00 | | Malignant neoplasm of anterior wall of nasopharynx NOS | |  |
| B074.00 | | Malignant neoplasm, overlapping lesion of nasopharynx | |  |
| B07y.00 | | Malignant neoplasm of other specified site of nasopharynx | |  |
| B07z.00 | | Malignant neoplasm of nasopharynx NOS | |  |
| B08..00 | | Malignant neoplasm of hypopharynx | |  |
| B080.00 | | Malignant neoplasm of postcricoid region | |  |
| B081.00 | | Malignant neoplasm of pyriform sinus | |  |
| B082.00 | | Malignant neoplasm aryepiglottic fold, hypopharyngeal aspect | |  |
| B083.00 | | Malignant neoplasm of posterior pharynx | |  |
| B08y.00 | | Malignant neoplasm of other specified hypopharyngeal site | |  |
| B08z.00 | | Malignant neoplasm of hypopharynx NOS | |  |
| B0z..00 | | Malig neop other/ill-defined sites lip, oral cavity, pharynx | |  |
| B0z0.00 | | Malignant neoplasm of pharynx unspecified | |  |
| B0z1.00 | | Malignant neoplasm of Waldeyer's ring | |  |
| B0z2.00 | | Malignant neoplasm of laryngopharynx | |  |
| B0zy.00 | | Malignant neoplasm of other sites lip, oral cavity, pharynx | |  |
| B0zz.00 | | Malignant neoplasm of lip, oral cavity and pharynx NOS | |  |
| B1...00 | | Malignant neoplasm of digestive organs and peritoneum | |  |
| B1...11 | | Carcinoma of digestive organs and peritoneum | |  |
| B10..00 | | Malignant neoplasm of oesophagus | |  |
| B100.00 | | Malignant neoplasm of cervical oesophagus | |  |
| B101.00 | | Malignant neoplasm of thoracic oesophagus | |  |
| B102.00 | | Malignant neoplasm of abdominal oesophagus | |  |
| B103.00 | | Malignant neoplasm of upper third of oesophagus | |  |
| B104.00 | | Malignant neoplasm of middle third of oesophagus | |  |
| B105.00 | | Malignant neoplasm of lower third of oesophagus | |  |
| B106.00 | | Malignant neoplasm, overlapping lesion of oesophagus | |  |
| B107.00 | | Siewert type I adenocarcinoma | |  |
| B10y.00 | | Malignant neoplasm of other specified part of oesophagus | |  |
| B10z.00 | | Malignant neoplasm of oesophagus NOS | |  |
| B10z.11 | | Oesophageal cancer | |  |
| B11..00 | | Malignant neoplasm of stomach | |  |
| B11..11 | | Gastric neoplasm | |  |
| B110.00 | | Malignant neoplasm of cardia of stomach | |  |
| B110000 | | Malignant neoplasm of cardiac orifice of stomach | |  |
| B110100 | | Malignant neoplasm of cardio-oesophageal junction of stomach | |  |
| B110111 | | Malignant neoplasm of gastro-oesophageal junction | |  |
| B110z00 | | Malignant neoplasm of cardia of stomach NOS | |  |
| B111.00 | | Malignant neoplasm of pylorus of stomach | |  |
| B111000 | | Malignant neoplasm of prepylorus of stomach | |  |
| B111100 | | Malignant neoplasm of pyloric canal of stomach | |  |
| B111z00 | | Malignant neoplasm of pylorus of stomach NOS | |  |
| B112.00 | | Malignant neoplasm of pyloric antrum of stomach | |  |
| B113.00 | | Malignant neoplasm of fundus of stomach | |  |
| B114.00 | | Malignant neoplasm of body of stomach | |  |
| B115.00 | | Malignant neoplasm of lesser curve of stomach unspecified | |  |
| B116.00 | | Malignant neoplasm of greater curve of stomach unspecified | |  |
| B117.00 | | Malignant neoplasm, overlapping lesion of stomach | |  |
| B118.00 | | Siewert type II adenocarcinoma | |  |
| B119.00 | | Siewert type III adenocarcinoma | |  |
| B11y.00 | | Malignant neoplasm of other specified site of stomach | |  |
| B11y000 | | Malignant neoplasm of anterior wall of stomach NEC | |  |
| B11y100 | | Malignant neoplasm of posterior wall of stomach NEC | |  |
| B11yz00 | | Malignant neoplasm of other specified site of stomach NOS | |  |
| B11z.00 | | Malignant neoplasm of stomach NOS | |  |
| B12..00 | | Malignant neoplasm of small intestine and duodenum | |  |
| B120.00 | | Malignant neoplasm of duodenum | |  |
| B121.00 | | Malignant neoplasm of jejunum | |  |
| B122.00 | | Malignant neoplasm of ileum | |  |
| B123.00 | | Malignant neoplasm of Meckel's diverticulum | |  |
| B124.00 | | Malignant neoplasm, overlapping lesion of small intestine | |  |
| B12y.00 | | Malignant neoplasm of other specified site small intestine | |  |
| B12z.00 | | Malignant neoplasm of small intestine NOS | |  |
| B13..00 | | Malignant neoplasm of colon | |  |
| B130.00 | | Malignant neoplasm of hepatic flexure of colon | |  |
| B131.00 | | Malignant neoplasm of transverse colon | |  |
| B132.00 | | Malignant neoplasm of descending colon | |  |
| B133.00 | | Malignant neoplasm of sigmoid colon | |  |
| B134.00 | | Malignant neoplasm of caecum | |  |
| B134.11 | | Carcinoma of caecum | |  |
| B135.00 | | Malignant neoplasm of appendix | |  |
| B136.00 | | Malignant neoplasm of ascending colon | |  |
| B137.00 | | Malignant neoplasm of splenic flexure of colon | |  |
| B138.00 | | Malignant neoplasm, overlapping lesion of colon | |  |
| B139.00 | | Hereditary nonpolyposis colon cancer | |  |
| B13y.00 | | Malignant neoplasm of other specified sites of colon | |  |
| B13z.00 | | Malignant neoplasm of colon NOS | |  |
| B13z.11 | | Colonic cancer | |  |
| B14..00 | | Malignant neoplasm of rectum, rectosigmoid junction and anus | |  |
| B140.00 | | Malignant neoplasm of rectosigmoid junction | |  |
| B141.00 | | Malignant neoplasm of rectum | |  |
| B141.11 | | Carcinoma of rectum | |  |
| B141.12 | | Rectal carcinoma | |  |
| B142.00 | | Malignant neoplasm of anal canal | |  |
| B142.11 | | Anal carcinoma | |  |
| B142000 | | Malignant neoplasm of cloacogenic zone | |  |
| B143.00 | | Malignant neoplasm of anus unspecified | |  |
| B14y.00 | | Malig neop other site rectum, rectosigmoid junction and anus | |  |
| B14z.00 | | Malignant neoplasm rectum,rectosigmoid junction and anus NOS | |  |
| B15..00 | | Malignant neoplasm of liver and intrahepatic bile ducts | |  |
| B150.00 | | Primary malignant neoplasm of liver | |  |
| B150000 | | Primary carcinoma of liver | |  |
| B150100 | | Hepatoblastoma of liver | |  |
| B150200 | | Primary angiosarcoma of liver | |  |
| B150300 | | Hepatocellular carcinoma | |  |
| B150z00 | | Primary malignant neoplasm of liver NOS | |  |
| B151.00 | | Malignant neoplasm of intrahepatic bile ducts | |  |
| B151000 | | Malignant neoplasm of interlobular bile ducts | |  |
| B151100 | | Malignant neoplasm of interlobular biliary canals | |  |
| B151200 | | Malignant neoplasm of intrahepatic biliary passages | |  |
| B151400 | | Malignant neoplasm of intrahepatic gall duct | |  |
| B151z00 | | Malignant neoplasm of intrahepatic bile ducts NOS | |  |
| B152.00 | | Malignant neoplasm of liver unspecified | |  |
| B153.00 | | Secondary malignant neoplasm of liver | |  |
| B15z.00 | | Malignant neoplasm of liver and intrahepatic bile ducts NOS | |  |
| B16..00 | | Malignant neoplasm gallbladder and extrahepatic bile ducts | |  |
| B160.00 | | Malignant neoplasm of gallbladder | |  |
| B160.11 | | Carcinoma gallbladder | |  |
| B161.00 | | Malignant neoplasm of extrahepatic bile ducts | |  |
| B161000 | | Malignant neoplasm of cystic duct | |  |
| B161100 | | Malignant neoplasm of hepatic duct | |  |
| B161200 | | Malignant neoplasm of common bile duct | |  |
| B161211 | | Carcinoma common bile duct | |  |
| B161300 | | Malignant neoplasm of sphincter of Oddi | |  |
| B161z00 | | Malignant neoplasm of extrahepatic bile ducts NOS | |  |
| B162.00 | | Malignant neoplasm of ampulla of Vater | |  |
| B163.00 | | Malignant neoplasm, overlapping lesion of biliary tract | |  |
| B16y.00 | | Malignant neoplasm other gallbladder/extrahepatic bile duct | |  |
| B16z.00 | | Malignant neoplasm gallbladder/extrahepatic bile ducts NOS | |  |
| B17..00 | | Malignant neoplasm of pancreas | |  |
| B170.00 | | Malignant neoplasm of head of pancreas | |  |
| B171.00 | | Malignant neoplasm of body of pancreas | |  |
| B172.00 | | Malignant neoplasm of tail of pancreas | |  |
| B173.00 | | Malignant neoplasm of pancreatic duct | |  |
| B174.00 | | Malignant neoplasm of Islets of Langerhans | |  |
| B175.00 | | Malignant neoplasm, overlapping lesion of pancreas | |  |
| B176.00 | | Somatostatinoma of pancreas | |  |
| B17y.00 | | Malignant neoplasm of other specified sites of pancreas | |  |
| B17y000 | | Malignant neoplasm of ectopic pancreatic tissue | |  |
| B17yz00 | | Malignant neoplasm of specified site of pancreas NOS | |  |
| B17z.00 | | Malignant neoplasm of pancreas NOS | |  |
| B18..00 | | Malignant neoplasm of retroperitoneum and peritoneum | |  |
| B180.00 | | Malignant neoplasm of retroperitoneum | |  |
| B180100 | | Malignant neoplasm of perinephric tissue | |  |
| B180200 | | Malignant neoplasm of retrocaecal tissue | |  |
| B180z00 | | Malignant neoplasm of retroperitoneum NOS | |  |
| B181.00 | | Mesothelioma of peritoneum | |  |
| B182.00 | | Overlapping malign lesion of retroperitoneum and peritoneum | |  |
| B18y.00 | | Malignant neoplasm of specified parts of peritoneum | |  |
| B18y100 | | Malignant neoplasm of mesocaecum | |  |
| B18y200 | | Malignant neoplasm of mesorectum | |  |
| B18y300 | | Malignant neoplasm of omentum | |  |
| B18y400 | | Malignant neoplasm of parietal peritoneum | |  |
| B18y500 | | Malignant neoplasm of pelvic peritoneum | |  |
| B18y600 | | Malignant neoplasm of the pouch of Douglas | |  |
| B18y700 | | Malignant neoplasm of mesentery | |  |
| B18yz00 | | Malignant neoplasm of specified parts of peritoneum NOS | |  |
| B18z.00 | | Malignant neoplasm of retroperitoneum and peritoneum NOS | |  |
| B1z..00 | | Malig neop oth/ill-defined sites digestive tract/peritoneum | |  |
| B1z0.00 | | Malignant neoplasm of intestinal tract, part unspecified | |  |
| B1z0.11 | | Cancer of bowel | |  |
| B1z1.00 | | Malignant neoplasm of spleen NEC | |  |
| B1z1000 | | Angiosarcoma of spleen | |  |
| B1z1100 | | Fibrosarcoma of spleen | |  |
| B1z1z00 | | Malignant neoplasm of spleen NOS | |  |
| B1z2.00 | | Malignant neoplasm, overlapping lesion of digestive system | |  |
| B1zy.00 | | Malignant neoplasm other spec digestive tract and peritoneum | |  |
| B1zz.00 | | Malignant neoplasm of digestive tract and peritoneum NOS | |  |
| B2...00 | | Malig neop of respiratory tract and intrathoracic organs | |  |
| B2...11 | | Carcinoma of respiratory tract and intrathoracic organs | |  |
| B20..00 | | Malig neop nasal cavities, middle ear and accessory sinuses | |  |
| B200.00 | | Malignant neoplasm of nasal cavities | |  |
| B200000 | | Malignant neoplasm of cartilage of nose | |  |
| B200100 | | Malignant neoplasm of nasal conchae | |  |
| B200200 | | Malignant neoplasm of septum of nose | |  |
| B200300 | | Malignant neoplasm of vestibule of nose | |  |
| B200z00 | | Malignant neoplasm of nasal cavities NOS | |  |
| B201.00 | | Malig neop auditory tube, middle ear and mastoid air cells | |  |
| B201000 | | Malignant neoplasm of auditory (Eustachian) tube | |  |
| B201100 | | Malignant neoplasm of tympanic cavity | |  |
| B201200 | | Malignant neoplasm of tympanic antrum | |  |
| B201300 | | Malignant neoplasm of mastoid air cells | |  |
| B201z00 | | Malig neop auditory tube, middle ear, mastoid air cells NOS | |  |
| B202.00 | | Malignant neoplasm of maxillary sinus | |  |
| B203.00 | | Malignant neoplasm of ethmoid sinus | |  |
| B204.00 | | Malignant neoplasm of frontal sinus | |  |
| B205.00 | | Malignant neoplasm of sphenoidal sinus | |  |
| B206.00 | | Malignant neoplasm, overlapping lesion of accessory sinuses | |  |
| B20y.00 | | Malig neop other site nasal cavity, middle ear and sinuses | |  |
| B20z.00 | | Malignant neoplasm of accessory sinus NOS | |  |
| B21..00 | | Malignant neoplasm of larynx | |  |
| B210.00 | | Malignant neoplasm of glottis | |  |
| B211.00 | | Malignant neoplasm of supraglottis | |  |
| B212.00 | | Malignant neoplasm of subglottis | |  |
| B213.00 | | Malignant neoplasm of laryngeal cartilage | |  |
| B213000 | | Malignant neoplasm of arytenoid cartilage | |  |
| B213100 | | Malignant neoplasm of cricoid cartilage | |  |
| B213200 | | Malignant neoplasm of cuneiform cartilage | |  |
| B213300 | | Malignant neoplasm of thyroid cartilage | |  |
| B213z00 | | Malignant neoplasm of laryngeal cartilage NOS | |  |
| B214.00 | | Malignant neoplasm, overlapping lesion of larynx | |  |
| B215.00 | | Malignant neoplasm of epiglottis NOS | |  |
| B21y.00 | | Malignant neoplasm of larynx, other specified site | |  |
| B21z.00 | | Malignant neoplasm of larynx NOS | |  |
| B22..00 | | Malignant neoplasm of trachea, bronchus and lung | |  |
| B220.00 | | Malignant neoplasm of trachea | |  |
| B220100 | | Malignant neoplasm of mucosa of trachea | |  |
| B220z00 | | Malignant neoplasm of trachea NOS | |  |
| B221.00 | | Malignant neoplasm of main bronchus | |  |
| B221000 | | Malignant neoplasm of carina of bronchus | |  |
| B221100 | | Malignant neoplasm of hilus of lung | |  |
| B221z00 | | Malignant neoplasm of main bronchus NOS | |  |
| B222.00 | | Malignant neoplasm of upper lobe, bronchus or lung | |  |
| B222.11 | | Pancoast's syndrome | |  |
| B222000 | | Malignant neoplasm of upper lobe bronchus | |  |
| B222100 | | Malignant neoplasm of upper lobe of lung | |  |
| B222z00 | | Malignant neoplasm of upper lobe, bronchus or lung NOS | |  |
| B223.00 | | Malignant neoplasm of middle lobe, bronchus or lung | |  |
| B223000 | | Malignant neoplasm of middle lobe bronchus | |  |
| B223100 | | Malignant neoplasm of middle lobe of lung | |  |
| B223z00 | | Malignant neoplasm of middle lobe, bronchus or lung NOS | |  |
| B224.00 | | Malignant neoplasm of lower lobe, bronchus or lung | |  |
| B224000 | | Malignant neoplasm of lower lobe bronchus | |  |
| B224100 | | Malignant neoplasm of lower lobe of lung | |  |
| B224z00 | | Malignant neoplasm of lower lobe, bronchus or lung NOS | |  |
| B225.00 | | Malignant neoplasm of overlapping lesion of bronchus & lung | |  |
| B226.00 | | Mesothelioma | |  |
| B22y.00 | | Malignant neoplasm of other sites of bronchus or lung | |  |
| B22z.00 | | Malignant neoplasm of bronchus or lung NOS | |  |
| B22z.11 | | Lung cancer | |  |
| B23..00 | | Malignant neoplasm of pleura | |  |
| B230.00 | | Malignant neoplasm of parietal pleura | |  |
| B231.00 | | Malignant neoplasm of visceral pleura | |  |
| B232.00 | | Mesothelioma of pleura | |  |
| B23y.00 | | Malignant neoplasm of other specified pleura | |  |
| B23z.00 | | Malignant neoplasm of pleura NOS | |  |
| B24..00 | | Malignant neoplasm of thymus, heart and mediastinum | |  |
| B240.00 | | Malignant neoplasm of thymus | |  |
| B241.00 | | Malignant neoplasm of heart | |  |
| B241000 | | Malignant neoplasm of endocardium | |  |
| B241200 | | Malignant neoplasm of myocardium | |  |
| B241300 | | Malignant neoplasm of pericardium | |  |
| B241400 | | Mesothelioma of pericardium | |  |
| B241z00 | | Malignant neoplasm of heart NOS | |  |
| B242.00 | | Malignant neoplasm of anterior mediastinum | |  |
| B243.00 | | Malignant neoplasm of posterior mediastinum | |  |
| B24X.00 | | Malignant neoplasm of mediastinum, part unspecified | |  |
| B24y.00 | | Malig neop of other site of heart, thymus and mediastinum | |  |
| B24z.00 | | Malignant neoplasm of heart, thymus and mediastinum NOS | |  |
| B25..00 | | Malig neo, overlapping lesion of heart, mediastinum & pleura | |  |
| B26..00 | | Malignant neoplasm, overlap lesion of resp & intrathor orgs | |  |
| B2z..00 | | Malig neop other/ill-defined sites resp/intrathoracic organs | |  |
| B2z0.00 | | Malig neop of upper respiratory tract, part unspecified | |  |
| B2zy.00 | | Malignant neoplasm of other site of respiratory tract | |  |
| B2zz.00 | | Malignant neoplasm of respiratory tract NOS | |  |
| B3...00 | | Malig neop of bone, connective tissue, skin and breast | |  |
| B3...11 | | Carcinoma of bone, connective tissue, skin and breast | |  |
| B3...12 | | Sarcoma of bone and connective tissue | |  |
| B30..00 | | Malignant neoplasm of bone and articular cartilage | |  |
| B30..11 | | Chondroma | |  |
| B30..12 | | Osteoma | |  |
| B300.00 | | Malignant neoplasm of bones of skull and face | |  |
| B300000 | | Malignant neoplasm of ethmoid bone | |  |
| B300100 | | Malignant neoplasm of frontal bone | |  |
| B300200 | | Malignant neoplasm of malar bone | |  |
| B300300 | | Malignant neoplasm of nasal bone | |  |
| B300400 | | Malignant neoplasm of occipital bone | |  |
| B300500 | | Malignant neoplasm of orbital bone | |  |
| B300600 | | Malignant neoplasm of parietal bone | |  |
| B300700 | | Malignant neoplasm of sphenoid bone | |  |
| B300800 | | Malignant neoplasm of temporal bone | |  |
| B300900 | | Malignant neoplasm of zygomatic bone | |  |
| B300A00 | | Malignant neoplasm of maxilla | |  |
| B300B00 | | Malignant neoplasm of turbinate | |  |
| B300C00 | | Malignant neoplasm of vomer | |  |
| B300z00 | | Malignant neoplasm of bones of skull and face NOS | |  |
| B301.00 | | Malignant neoplasm of mandible | |  |
| B302.00 | | Malignant neoplasm of vertebral column | |  |
| B302000 | | Malignant neoplasm of cervical vertebra | |  |
| B302100 | | Malignant neoplasm of thoracic vertebra | |  |
| B302200 | | Malignant neoplasm of lumbar vertebra | |  |
| B302z00 | | Malignant neoplasm of vertebral column NOS | |  |
| B303.00 | | Malignant neoplasm of ribs, sternum and clavicle | |  |
| B303000 | | Malignant neoplasm of rib | |  |
| B303100 | | Malignant neoplasm of sternum | |  |
| B303200 | | Malignant neoplasm of clavicle | |  |
| B303300 | | Malignant neoplasm of costal cartilage | |  |
| B303400 | | Malignant neoplasm of costo-vertebral joint | |  |
| B303500 | | Malignant neoplasm of xiphoid process | |  |
| B303z00 | | Malignant neoplasm of rib, sternum and clavicle NOS | |  |
| B304.00 | | Malignant neoplasm of scapula and long bones of upper arm | |  |
| B304000 | | Malignant neoplasm of scapula | |  |
| B304100 | | Malignant neoplasm of acromion | |  |
| B304200 | | Malignant neoplasm of humerus | |  |
| B304300 | | Malignant neoplasm of radius | |  |
| B304400 | | Malignant neoplasm of ulna | |  |
| B304z00 | | Malig neop of scapula and long bones of upper arm NOS | |  |
| B305.00 | | Malignant neoplasm of hand bones | |  |
| B305.11 | | Malignant neoplasm of carpal bones | |  |
| B305.12 | | Malignant neoplasm of metacarpal bones | |  |
| B305000 | | Malignant neoplasm of carpal bone - scaphoid | |  |
| B305100 | | Malignant neoplasm of carpal bone - lunate | |  |
| B305800 | | Malignant neoplasm of first metacarpal bone | |  |
| B305A00 | | Malignant neoplasm of third metacarpal bone | |  |
| B305C00 | | Malignant neoplasm of fifth metacarpal bone | |  |
| B305D00 | | Malignant neoplasm of phalanges of hand | |  |
| B305z00 | | Malignant neoplasm of hand bones NOS | |  |
| B306.00 | | Malignant neoplasm of pelvic bones, sacrum and coccyx | |  |
| B306000 | | Malignant neoplasm of ilium | |  |
| B306100 | | Malignant neoplasm of ischium | |  |
| B306200 | | Malignant neoplasm of pubis | |  |
| B306300 | | Malignant neoplasm of sacral vertebra | |  |
| B306400 | | Malignant neoplasm of coccygeal vertebra | |  |
| B306500 | | Malignant sacral teratoma | |  |
| B306z00 | | Malignant neoplasm of pelvis, sacrum or coccyx NOS | |  |
| B307.00 | | Malignant neoplasm of long bones of leg | |  |
| B307000 | | Malignant neoplasm of femur | |  |
| B307100 | | Malignant neoplasm of fibula | |  |
| B307200 | | Malignant neoplasm of tibia | |  |
| B307z00 | | Malignant neoplasm of long bones of leg NOS | |  |
| B308.00 | | Malignant neoplasm of short bones of leg | |  |
| B308000 | | Malignant neoplasm of patella | |  |
| B308100 | | Malignant neoplasm of talus | |  |
| B308200 | | Malignant neoplasm of calcaneum | |  |
| B308300 | | Malignant neoplasm of medial cuneiform | |  |
| B308800 | | Malignant neoplasm of first metatarsal bone | |  |
| B308900 | | Malignant neoplasm of second metatarsal bone | |  |
| B308B00 | | Malignant neoplasm of fourth metatarsal bone | |  |
| B308D00 | | Malignant neoplasm of phalanges of foot | |  |
| B308z00 | | Malignant neoplasm of short bones of leg NOS | |  |
| B30W.00 | | Malignant neoplasm/overlap lesion/bone+articulr cartilage | |  |
| B30X.00 | | Malignant neoplasm/bones+articular cartilage/limb,unspfd | |  |
| B30z.00 | | Malignant neoplasm of bone and articular cartilage NOS | |  |
| B30z000 | | Osteosarcoma | |  |
| B31..00 | | Malignant neoplasm of connective and other soft tissue | |  |
| B310.00 | | Malig neop of connective and soft tissue head, face and neck | |  |
| B310000 | | Malignant neoplasm of soft tissue of head | |  |
| B310100 | | Malignant neoplasm of soft tissue of face | |  |
| B310200 | | Malignant neoplasm of soft tissue of neck | |  |
| B310300 | | Malignant neoplasm of cartilage of ear | |  |
| B310400 | | Malignant neoplasm of tarsus of eyelid | |  |
| B310500 | | Malignant neoplasm soft tissues of cervical spine | |  |
| B310z00 | | Malig neop connective and soft tissue head, face, neck NOS | |  |
| B311.00 | | Malig neop connective and soft tissue upper limb/shoulder | |  |
| B311000 | | Malignant neoplasm of connective and soft tissue of shoulder | |  |
| B311100 | | Malignant neoplasm of connective and soft tissue, upper arm | |  |
| B311200 | | Malignant neoplasm of connective and soft tissue of fore-arm | |  |
| B311300 | | Malignant neoplasm of connective and soft tissue of hand | |  |
| B311400 | | Malignant neoplasm of connective and soft tissue of finger | |  |
| B311500 | | Malignant neoplasm of connective and soft tissue of thumb | |  |
| B311z00 | | Malig neop connective soft tissue upper limb/shoulder NOS | |  |
| B312.00 | | Malig neop of connective and soft tissue of hip and leg | |  |
| B312000 | | Malignant neoplasm of connective and soft tissue of hip | |  |
| B312100 | | Malig neop of connective and soft tissue thigh and upper leg | |  |
| B312200 | | Malig neop connective and soft tissue of popliteal space | |  |
| B312300 | | Malig neop of connective and soft tissue of lower leg | |  |
| B312400 | | Malignant neoplasm of connective and soft tissue of foot | |  |
| B312500 | | Malignant neoplasm of connective and soft tissue of toe | |  |
| B312z00 | | Malig neop connective and soft tissue hip and leg NOS | |  |
| B313.00 | | Malignant neoplasm of connective and soft tissue of thorax | |  |
| B313000 | | Malignant neoplasm of connective and soft tissue of axilla | |  |
| B313100 | | Malignant neoplasm of diaphragm | |  |
| B313200 | | Malignant neoplasm of great vessels | |  |
| B313300 | | Malig neoplasm of connective and soft tissues of thor spine | |  |
| B313z00 | | Malig neop of connective and soft tissue of thorax NOS | |  |
| B314.00 | | Malignant neoplasm of connective and soft tissue of abdomen | |  |
| B314000 | | Malig neop of connective and soft tissue of abdominal wall | |  |
| B314100 | | Malig neoplasm of connective and soft tissues of lumb spine | |  |
| B314z00 | | Malig neop of connective and soft tissue of abdomen NOS | |  |
| B315.00 | | Malignant neoplasm of connective and soft tissue of pelvis | |  |
| B315000 | | Malignant neoplasm of connective and soft tissue of buttock | |  |
| B315100 | | Malig neop of connective and soft tissue of inguinal region | |  |
| B315200 | | Malignant neoplasm of connective and soft tissue of perineum | |  |
| B315300 | | Malig neopl of connective and soft tissue - sacrum or coccyx | |  |
| B315z00 | | Malig neop of connective and soft tissue of pelvis NOS | |  |
| B316.00 | | Malig neop of connective and soft tissue trunk unspecified | |  |
| B317.00 | | Malignant neoplasm, overlap lesion connective & soft tissue | |  |
| B31y.00 | | Malig neop connective and soft tissue other specified site | |  |
| B31z.00 | | Malignant neoplasm of connective and soft tissue, site NOS | |  |
| B31z000 | | Kaposi's sarcoma of soft tissue | |  |
| B32..00 | | Malignant melanoma of skin | |  |
| B320.00 | | Malignant melanoma of lip | |  |
| B321.00 | | Malignant melanoma of eyelid including canthus | |  |
| B322.00 | | Malignant melanoma of ear and external auricular canal | |  |
| B322000 | | Malignant melanoma of auricle (ear) | |  |
| B322100 | | Malignant melanoma of external auditory meatus | |  |
| B322z00 | | Malignant melanoma of ear and external auricular canal NOS | |  |
| B323.00 | | Malignant melanoma of other and unspecified parts of face | |  |
| B323000 | | Malignant melanoma of external surface of cheek | |  |
| B323100 | | Malignant melanoma of chin | |  |
| B323200 | | Malignant melanoma of eyebrow | |  |
| B323300 | | Malignant melanoma of forehead | |  |
| B323400 | | Malignant melanoma of external surface of nose | |  |
| B323500 | | Malignant melanoma of temple | |  |
| B323z00 | | Malignant melanoma of face NOS | |  |
| B324.00 | | Malignant melanoma of scalp and neck | |  |
| B324000 | | Malignant melanoma of scalp | |  |
| B324100 | | Malignant melanoma of neck | |  |
| B324z00 | | Malignant melanoma of scalp and neck NOS | |  |
| B325.00 | | Malignant melanoma of trunk (excluding scrotum) | |  |
| B325000 | | Malignant melanoma of axilla | |  |
| B325100 | | Malignant melanoma of breast | |  |
| B325200 | | Malignant melanoma of buttock | |  |
| B325300 | | Malignant melanoma of groin | |  |
| B325400 | | Malignant melanoma of perianal skin | |  |
| B325500 | | Malignant melanoma of perineum | |  |
| B325600 | | Malignant melanoma of umbilicus | |  |
| B325700 | | Malignant melanoma of back | |  |
| B325800 | | Malignant melanoma of chest wall | |  |
| B325z00 | | Malignant melanoma of trunk, excluding scrotum, NOS | |  |
| B326.00 | | Malignant melanoma of upper limb and shoulder | |  |
| B326000 | | Malignant melanoma of shoulder | |  |
| B326100 | | Malignant melanoma of upper arm | |  |
| B326200 | | Malignant melanoma of fore-arm | |  |
| B326300 | | Malignant melanoma of hand | |  |
| B326400 | | Malignant melanoma of finger | |  |
| B326500 | | Malignant melanoma of thumb | |  |
| B326z00 | | Malignant melanoma of upper limb or shoulder NOS | |  |
| B327.00 | | Malignant melanoma of lower limb and hip | |  |
| B327000 | | Malignant melanoma of hip | |  |
| B327100 | | Malignant melanoma of thigh | |  |
| B327200 | | Malignant melanoma of knee | |  |
| B327300 | | Malignant melanoma of popliteal fossa area | |  |
| B327400 | | Malignant melanoma of lower leg | |  |
| B327500 | | Malignant melanoma of ankle | |  |
| B327600 | | Malignant melanoma of heel | |  |
| B327700 | | Malignant melanoma of foot | |  |
| B327800 | | Malignant melanoma of toe | |  |
| B327900 | | Malignant melanoma of great toe | |  |
| B327z00 | | Malignant melanoma of lower limb or hip NOS | |  |
| B328.00 | | Malignant melanoma stage IA | |  |
| B329.00 | | Malignant melanoma stage IB | |  |
| B32A.00 | | Malignant melanoma stage IIA | |  |
| B32B.00 | | Malignant melanoma stage IIB | |  |
| B32C.00 | | Malignant melanoma stage IIC | |  |
| B32D.00 | | Malignant melanoma stage IIIA | |  |
| B32E.00 | | Malignant melanoma stage IIIB | |  |
| B32F.00 | | Malignant melanoma stage IIIC | |  |
| B32G.00 | | Malignant melanoma stage IV M1a | |  |
| B32H.00 | | Malignant melanoma stage IV M1b | |  |
| B32J.00 | | Malignant melanoma stage IV M1c | |  |
| B32y.00 | | Malignant melanoma of other specified skin site | |  |
| B32y000 | | Overlapping malignant melanoma of skin | |  |
| B32z.00 | | Malignant melanoma of skin NOS | |  |
| B33..00 | | Other malignant neoplasm of skin | |  |
| B33..11 | | Basal cell carcinoma | |  |
| B33..12 | | Epithelioma | |  |
| B33..13 | | Rodent ulcer | |  |
| B33..14 | | Malignant neoplasm of sebaceous gland | |  |
| B33..15 | | Malignant neoplasm of sweat gland | |  |
| B33..16 | | Epithelioma basal cell | |  |
| B330.00 | | Malignant neoplasm of skin of lip | |  |
| B331.00 | | Malignant neoplasm of eyelid including canthus | |  |
| B331000 | | Malignant neoplasm of canthus | |  |
| B331100 | | Malignant neoplasm of upper eyelid | |  |
| B331200 | | Malignant neoplasm of lower eyelid | |  |
| B332.00 | | Malignant neoplasm skin of ear and external auricular canal | |  |
| B332000 | | Malignant neoplasm of skin of auricle (ear) | |  |
| B332100 | | Malignant neoplasm of skin of external auditory meatus | |  |
| B332200 | | Malignant neoplasm of pinna NEC | |  |
| B332z00 | | Malig neop skin of ear and external auricular canal NOS | |  |
| B333.00 | | Malignant neoplasm skin of other and unspecified parts face | |  |
| B333000 | | Malignant neoplasm of skin of cheek, external | |  |
| B333100 | | Malignant neoplasm of skin of chin | |  |
| B333200 | | Malignant neoplasm of skin of eyebrow | |  |
| B333300 | | Malignant neoplasm of skin of forehead | |  |
| B333400 | | Malignant neoplasm of skin of nose (external) | |  |
| B333500 | | Malignant neoplasm of skin of temple | |  |
| B333z00 | | Malignant neoplasm skin other and unspec part of face NOS | |  |
| B334.00 | | Malignant neoplasm of scalp and skin of neck | |  |
| B334000 | | Malignant neoplasm of scalp | |  |
| B334100 | | Malignant neoplasm of skin of neck | |  |
| B334z00 | | Malignant neoplasm of scalp or skin of neck NOS | |  |
| B335.00 | | Malignant neoplasm of skin of trunk, excluding scrotum | |  |
| B335000 | | Malignant neoplasm of skin of axillary fold | |  |
| B335100 | | Malignant neoplasm of skin of chest, excluding breast | |  |
| B335200 | | Malignant neoplasm of skin of breast | |  |
| B335300 | | Malignant neoplasm of skin of abdominal wall | |  |
| B335400 | | Malignant neoplasm of skin of umbilicus | |  |
| B335500 | | Malignant neoplasm of skin of groin | |  |
| B335600 | | Malignant neoplasm of skin of perineum | |  |
| B335700 | | Malignant neoplasm of skin of back | |  |
| B335800 | | Malignant neoplasm of skin of buttock | |  |
| B335900 | | Malignant neoplasm of perianal skin | |  |
| B335A00 | | Malignant neoplasm of skin of scapular region | |  |
| B335z00 | | Malignant neoplasm of skin of trunk, excluding scrotum, NOS | |  |
| B336.00 | | Malignant neoplasm of skin of upper limb and shoulder | |  |
| B336000 | | Malignant neoplasm of skin of shoulder | |  |
| B336100 | | Malignant neoplasm of skin of upper arm | |  |
| B336200 | | Malignant neoplasm of skin of fore-arm | |  |
| B336300 | | Malignant neoplasm of skin of hand | |  |
| B336400 | | Malignant neoplasm of skin of finger | |  |
| B336500 | | Malignant neoplasm of skin of thumb | |  |
| B336z00 | | Malignant neoplasm of skin of upper limb or shoulder NOS | |  |
| B337.00 | | Malignant neoplasm of skin of lower limb and hip | |  |
| B337000 | | Malignant neoplasm of skin of hip | |  |
| B337100 | | Malignant neoplasm of skin of thigh | |  |
| B337200 | | Malignant neoplasm of skin of knee | |  |
| B337300 | | Malignant neoplasm of skin of popliteal fossa area | |  |
| B337400 | | Malignant neoplasm of skin of lower leg | |  |
| B337500 | | Malignant neoplasm of skin of ankle | |  |
| B337600 | | Malignant neoplasm of skin of heel | |  |
| B337700 | | Malignant neoplasm of skin of foot | |  |
| B337800 | | Malignant neoplasm of skin of toe | |  |
| B337900 | | Malignant neoplasm of skin of great toe | |  |
| B337z00 | | Malignant neoplasm of skin of lower limb or hip NOS | |  |
| B338.00 | | Squamous cell carcinoma of skin | |  |
| B339.00 | | Dermatofibrosarcoma protuberans | |  |
| B33X.00 | | Malignant neoplasm overlapping lesion of skin | |  |
| B33y.00 | | Malignant neoplasm of other specified skin sites | |  |
| B33z.00 | | Malignant neoplasm of skin NOS | |  |
| B33z.11 | | Squamous cell carcinoma of skin NOS | |  |
| B33z000 | | Kaposi's sarcoma of skin | |  |
| B33z100 | | Naevoid basal cell carcinoma syndrome | |  |
| B33z111 | | Basal cell naevus syndrome | |  |
| B34..00 | | Malignant neoplasm of female breast | |  |
| B34..11 | | Ca female breast | |  |
| B340.00 | | Malignant neoplasm of nipple and areola of female breast | |  |
| B340000 | | Malignant neoplasm of nipple of female breast | |  |
| B340100 | | Malignant neoplasm of areola of female breast | |  |
| B340z00 | | Malignant neoplasm of nipple or areola of female breast NOS | |  |
| B341.00 | | Malignant neoplasm of central part of female breast | |  |
| B342.00 | | Malignant neoplasm of upper-inner quadrant of female breast | |  |
| B343.00 | | Malignant neoplasm of lower-inner quadrant of female breast | |  |
| B344.00 | | Malignant neoplasm of upper-outer quadrant of female breast | |  |
| B345.00 | | Malignant neoplasm of lower-outer quadrant of female breast | |  |
| B346.00 | | Malignant neoplasm of axillary tail of female breast | |  |
| B347.00 | | Malignant neoplasm, overlapping lesion of breast | |  |
| B34y.00 | | Malignant neoplasm of other site of female breast | |  |
| B34y000 | | Malignant neoplasm of ectopic site of female breast | |  |
| B34yz00 | | Malignant neoplasm of other site of female breast NOS | |  |
| B34z.00 | | Malignant neoplasm of female breast NOS | |  |
| B35..00 | | Malignant neoplasm of male breast | |  |
| B350.00 | | Malignant neoplasm of nipple and areola of male breast | |  |
| B350000 | | Malignant neoplasm of nipple of male breast | |  |
| B350100 | | Malignant neoplasm of areola of male breast | |  |
| B35z.00 | | Malignant neoplasm of other site of male breast | |  |
| B35z000 | | Malignant neoplasm of ectopic site of male breast | |  |
| B35zz00 | | Malignant neoplasm of male breast NOS | |  |
| B36..00 | | Local recurrence of malignant tumour of breast | |  |
| B3y..00 | | Malig neop of bone, connective tissue, skin and breast OS | |  |
| B3z..00 | | Malig neop of bone, connective tissue, skin and breast NOS | |  |
| B4...00 | | Malignant neoplasm of genitourinary organ | |  |
| B4...11 | | Carcinoma of genitourinary organ | |  |
| B40..00 | | Malignant neoplasm of uterus, part unspecified | |  |
| B41..00 | | Malignant neoplasm of cervix uteri | |  |
| B41..11 | | Cervical carcinoma (uterus) | |  |
| B410.00 | | Malignant neoplasm of endocervix | |  |
| B410000 | | Malignant neoplasm of endocervical canal | |  |
| B410100 | | Malignant neoplasm of endocervical gland | |  |
| B410z00 | | Malignant neoplasm of endocervix NOS | |  |
| B411.00 | | Malignant neoplasm of exocervix | |  |
| B412.00 | | Malignant neoplasm, overlapping lesion of cervix uteri | |  |
| B41y.00 | | Malignant neoplasm of other site of cervix | |  |
| B41y000 | | Malignant neoplasm of cervical stump | |  |
| B41y100 | | Malignant neoplasm of squamocolumnar junction of cervix | |  |
| B41yz00 | | Malignant neoplasm of other site of cervix NOS | |  |
| B41z.00 | | Malignant neoplasm of cervix uteri NOS | |  |
| B42..00 | | Malignant neoplasm of placenta | |  |
| B420.00 | | Choriocarcinoma | |  |
| B43..00 | | Malignant neoplasm of body of uterus | |  |
| B430.00 | | Malignant neoplasm of corpus uteri, excluding isthmus | |  |
| B430000 | | Malignant neoplasm of cornu of corpus uteri | |  |
| B430100 | | Malignant neoplasm of fundus of corpus uteri | |  |
| B430200 | | Malignant neoplasm of endometrium of corpus uteri | |  |
| B430211 | | Malignant neoplasm of endometrium | |  |
| B430300 | | Malignant neoplasm of myometrium of corpus uteri | |  |
| B430z00 | | Malignant neoplasm of corpus uteri NOS | |  |
| B431.00 | | Malignant neoplasm of isthmus of uterine body | |  |
| B431000 | | Malignant neoplasm of lower uterine segment | |  |
| B431z00 | | Malignant neoplasm of isthmus of uterine body NOS | |  |
| B432.00 | | Malignant neoplasm of overlapping lesion of corpus uteri | |  |
| B43y.00 | | Malignant neoplasm of other site of uterine body | |  |
| B43z.00 | | Malignant neoplasm of body of uterus NOS | |  |
| B44..00 | | Malignant neoplasm of ovary and other uterine adnexa | |  |
| B440.00 | | Malignant neoplasm of ovary | |  |
| B440.11 | | Cancer of ovary | |  |
| B441.00 | | Malignant neoplasm of fallopian tube | |  |
| B442.00 | | Malignant neoplasm of broad ligament | |  |
| B443.00 | | Malignant neoplasm of parametrium | |  |
| B44y.00 | | Malignant neoplasm of other site of uterine adnexa | |  |
| B44z.00 | | Malignant neoplasm of uterine adnexa NOS | |  |
| B45..00 | | Malig neop of other and unspecified female genital organs | |  |
| B450.00 | | Malignant neoplasm of vagina | |  |
| B450100 | | Malignant neoplasm of vaginal vault | |  |
| B450z00 | | Malignant neoplasm of vagina NOS | |  |
| B451.00 | | Malignant neoplasm of labia majora | |  |
| B451000 | | Malignant neoplasm of greater vestibular (Bartholin's) gland | |  |
| B451z00 | | Malignant neoplasm of labia majora NOS | |  |
| B452.00 | | Malignant neoplasm of labia minora | |  |
| B453.00 | | Malignant neoplasm of clitoris | |  |
| B454.00 | | Malignant neoplasm of vulva unspecified | |  |
| B454.11 | | Primary vulval cancer | |  |
| B45X.00 | | Malignant neoplasm/overlapping lesion/feml genital organs | |  |
| B45y.00 | | Malignant neoplasm of other specified female genital organ | |  |
| B45y000 | | Malignant neoplasm of overlapping lesion of vulva | |  |
| B45z.00 | | Malignant neoplasm of female genital organ NOS | |  |
| B46..00 | | Malignant neoplasm of prostate | |  |
| B47..00 | | Malignant neoplasm of testis | |  |
| B470.00 | | Malignant neoplasm of undescended testis | |  |
| B470200 | | Seminoma of undescended testis | |  |
| B470300 | | Teratoma of undescended testis | |  |
| B470z00 | | Malignant neoplasm of undescended testis NOS | |  |
| B471.00 | | Malignant neoplasm of descended testis | |  |
| B471000 | | Seminoma of descended testis | |  |
| B471100 | | Teratoma of descended testis | |  |
| B471z00 | | Malignant neoplasm of descended testis NOS | |  |
| B47z.00 | | Malignant neoplasm of testis NOS | |  |
| B47z.11 | | Seminoma of testis | |  |
| B47z.12 | | Teratoma of testis | |  |
| B48..00 | | Malignant neoplasm of penis and other male genital organs | |  |
| B480.00 | | Malignant neoplasm of prepuce (foreskin) | |  |
| B481.00 | | Malignant neoplasm of glans penis | |  |
| B482.00 | | Malignant neoplasm of body of penis | |  |
| B483.00 | | Malignant neoplasm of penis, part unspecified | |  |
| B484.00 | | Malignant neoplasm of epididymis | |  |
| B485.00 | | Malignant neoplasm of spermatic cord | |  |
| B486.00 | | Malignant neoplasm of scrotum | |  |
| B487.00 | | Malignant neoplasm, overlapping lesion of penis | |  |
| B48y.00 | | Malignant neoplasm of other male genital organ | |  |
| B48y000 | | Malignant neoplasm of seminal vesicle | |  |
| B48y100 | | Malignant neoplasm of tunica vaginalis | |  |
| B48y200 | | Malignant neoplasm, overlapping lesion male genital orgs | |  |
| B48yz00 | | Malignant neoplasm of other male genital organ NOS | |  |
| B48z.00 | | Malignant neoplasm of penis and other male genital organ NOS | |  |
| B49..00 | | Malignant neoplasm of urinary bladder | |  |
| B490.00 | | Malignant neoplasm of trigone of urinary bladder | |  |
| B491.00 | | Malignant neoplasm of dome of urinary bladder | |  |
| B492.00 | | Malignant neoplasm of lateral wall of urinary bladder | |  |
| B493.00 | | Malignant neoplasm of anterior wall of urinary bladder | |  |
| B494.00 | | Malignant neoplasm of posterior wall of urinary bladder | |  |
| B495.00 | | Malignant neoplasm of bladder neck | |  |
| B496.00 | | Malignant neoplasm of ureteric orifice | |  |
| B497.00 | | Malignant neoplasm of urachus | |  |
| B498.00 | | Local recurrence of malignant tumour of urinary bladder | |  |
| B49y.00 | | Malignant neoplasm of other site of urinary bladder | |  |
| B49y000 | | Malignant neoplasm, overlapping lesion of bladder | |  |
| B49z.00 | | Malignant neoplasm of urinary bladder NOS | |  |
| B4A..00 | | Malig neop of kidney and other unspecified urinary organs | |  |
| B4A..11 | | Renal malignant neoplasm | |  |
| B4A0.00 | | Malignant neoplasm of kidney parenchyma | |  |
| B4A0000 | | Hypernephroma | |  |
| B4A1.00 | | Malignant neoplasm of renal pelvis | |  |
| B4A1000 | | Malignant neoplasm of renal calyces | |  |
| B4A1100 | | Malignant neoplasm of ureteropelvic junction | |  |
| B4A1z00 | | Malignant neoplasm of renal pelvis NOS | |  |
| B4A2.00 | | Malignant neoplasm of ureter | |  |
| B4A3.00 | | Malignant neoplasm of urethra | |  |
| B4A4.00 | | Malignant neoplasm of paraurethral glands | |  |
| B4Ay.00 | | Malignant neoplasm of other urinary organs | |  |
| B4Ay000 | | Malignant neoplasm of overlapping lesion of urinary organs | |  |
| B4Az.00 | | Malignant neoplasm of kidney or urinary organs NOS | |  |
| B4y..00 | | Malignant neoplasm of genitourinary organ OS | |  |
| B4z..00 | | Malignant neoplasm of genitourinary organ NOS | |  |
| B5...00 | | Malignant neoplasm of other and unspecified sites | |  |
| B5...11 | | Carcinoma of other and unspecified sites | |  |
| B50..00 | | Malignant neoplasm of eye | |  |
| B500.00 | | Malig neop eyeball excl conjunctiva, cornea, retina, choroid | |  |
| B500000 | | Malignant neoplasm of ciliary body | |  |
| B500100 | | Malignant neoplasm of iris | |  |
| B500200 | | Malignant neoplasm of crystalline lens | |  |
| B500z00 | | Malignant neoplasm of eyeball NOS | |  |
| B501.00 | | Malignant neoplasm of orbit | |  |
| B501000 | | Malignant neoplasm of connective tissue of orbit | |  |
| B501z00 | | Malignant neoplasm of orbit NOS | |  |
| B502.00 | | Malignant neoplasm of lacrimal gland | |  |
| B503.00 | | Malignant neoplasm of conjunctiva | |  |
| B504.00 | | Malignant neoplasm of cornea | |  |
| B505.00 | | Malignant neoplasm of retina | |  |
| B506.00 | | Malignant neoplasm of choroid | |  |
| B507.00 | | Malignant neoplasm of lacrimal duct | |  |
| B507000 | | Malignant neoplasm of lacrimal sac | |  |
| B507100 | | Malignant neoplasm of nasolacrimal duct | |  |
| B508.00 | | Malignant neoplasm, overlapping lesion of eye and adnexa | |  |
| B509.00 | | Malignant melanoma of eye | |  |
| B50y.00 | | Malignant neoplasm of other specified site of eye | |  |
| B50z.00 | | Malignant neoplasm of eye NOS | |  |
| B51..00 | | Malignant neoplasm of brain | |  |
| B51..11 | | Cerebral tumour - malignant | |  |
| B510.00 | | Malignant neoplasm cerebrum (excluding lobes and ventricles) | |  |
| B510000 | | Malignant neoplasm of basal ganglia | |  |
| B510100 | | Malignant neoplasm of cerebral cortex | |  |
| B510300 | | Malignant neoplasm of globus pallidus | |  |
| B510400 | | Malignant neoplasm of hypothalamus | |  |
| B510500 | | Malignant neoplasm of thalamus | |  |
| B510z00 | | Malignant neoplasm of cerebrum NOS | |  |
| B511.00 | | Malignant neoplasm of frontal lobe | |  |
| B512.00 | | Malignant neoplasm of temporal lobe | |  |
| B512000 | | Malignant neoplasm of hippocampus | |  |
| B512z00 | | Malignant neoplasm of temporal lobe NOS | |  |
| B513.00 | | Malignant neoplasm of parietal lobe | |  |
| B514.00 | | Malignant neoplasm of occipital lobe | |  |
| B515.00 | | Malignant neoplasm of cerebral ventricles | |  |
| B515000 | | Malignant neoplasm of choroid plexus | |  |
| B516.00 | | Malignant neoplasm of cerebellum | |  |
| B517.00 | | Malignant neoplasm of brain stem | |  |
| B517000 | | Malignant neoplasm of cerebral peduncle | |  |
| B517100 | | Malignant neoplasm of medulla oblongata | |  |
| B517200 | | Malignant neoplasm of midbrain | |  |
| B517300 | | Malignant neoplasm of pons | |  |
| B517z00 | | Malignant neoplasm of brain stem NOS | |  |
| B51y.00 | | Malignant neoplasm of other parts of brain | |  |
| B51y000 | | Malignant neoplasm of corpus callosum | |  |
| B51y200 | | Malignant neoplasm, overlapping lesion of brain | |  |
| B51yz00 | | Malignant neoplasm of other part of brain NOS | |  |
| B51z.00 | | Malignant neoplasm of brain NOS | |  |
| B52..00 | | Malig neop of other and unspecified parts of nervous system | |  |
| B520.00 | | Malignant neoplasm of cranial nerves | |  |
| B520000 | | Malignant neoplasm of olfactory bulb | |  |
| B520100 | | Malignant neoplasm of optic nerve | |  |
| B520200 | | Malignant neoplasm of acoustic nerve | |  |
| B520z00 | | Malignant neoplasm of cranial nerves NOS | |  |
| B521.00 | | Malignant neoplasm of cerebral meninges | |  |
| B521000 | | Malignant neoplasm of cerebral dura mater | |  |
| B521200 | | Malignant neoplasm of cerebral pia mater | |  |
| B521z00 | | Malignant neoplasm of cerebral meninges NOS | |  |
| B522.00 | | Malignant neoplasm of spinal cord | |  |
| B523.00 | | Malignant neoplasm of spinal meninges | |  |
| B523z00 | | Malignant neoplasm of spinal meninges NOS | |  |
| B524.00 | | Malig neopl peripheral nerves and autonomic nervous system | |  |
| B524000 | | Malignant neoplasm of peripheral nerves of head, face & neck | |  |
| B524100 | | Malignant neoplasm of peripheral nerve,upp limb,incl should | |  |
| B524200 | | Malignant neoplasm of peripheral nerve of low limb, incl hip | |  |
| B524300 | | Malignant neoplasm of peripheral nerve of thorax | |  |
| B524400 | | Malignant neoplasm of peripheral nerve of abdomen | |  |
| B524500 | | Malignant neoplasm of peripheral nerve of pelvis | |  |
| B524600 | | Malignant neoplasm,overlap lesion periph nerve & auton ns | |  |
| B524W00 | | Mal neoplasm/periph nerves+autonomic nervous system,unspc | |  |
| B525.00 | | Malignant neoplasm of cauda equina | |  |
| B52W.00 | | Malig neopl, overlap lesion brain & other part of CNS | |  |
| B52X.00 | | Malignant neoplasm of meninges, unspecified | |  |
| B52y.00 | | Malignant neoplasm of other specified part of nervous system | |  |
| B52z.00 | | Malignant neoplasm of nervous system NOS | |  |
| B53..00 | | Malignant neoplasm of thyroid gland | |  |
| B54..00 | | Malig neop of other endocrine glands and related structures | |  |
| B540.00 | | Malignant neoplasm of adrenal gland | |  |
| B540.11 | | Phaeochromocytoma | |  |
| B540000 | | Malignant neoplasm of adrenal cortex | |  |
| B540100 | | Malignant neoplasm of adrenal medulla | |  |
| B540z00 | | Malignant neoplasm of adrenal gland NOS | |  |
| B541.00 | | Malignant neoplasm of parathyroid gland | |  |
| B542.00 | | Malignant neoplasm pituitary gland and craniopharyngeal duct | |  |
| B542000 | | Malignant neoplasm of pituitary gland | |  |
| B542100 | | Malignant neoplasm of craniopharyngeal duct | |  |
| B542z00 | | Malig neop pituitary gland or craniopharyngeal duct NOS | |  |
| B543.00 | | Malignant neoplasm of pineal gland | |  |
| B544.00 | | Malignant neoplasm of carotid body | |  |
| B545.00 | | Malignant neoplasm of aortic body and other paraganglia | |  |
| B545000 | | Malignant neoplasm of glomus jugulare | |  |
| B545100 | | Malignant neoplasm of aortic body | |  |
| B545200 | | Malignant neoplasm of coccygeal body | |  |
| B545z00 | | Malignant neoplasm of aortic body or paraganglia NOS | |  |
| B546.00 | | Neuroblastoma | |  |
| B54X.00 | | Malignant neoplasm-pluriglandular involvement,unspecified | |  |
| B54y.00 | | Malignant neoplasm of other specified endocrine gland | |  |
| B54z.00 | | Malig neop of endocrine gland or related structure NOS | |  |
| B55..00 | | Malignant neoplasm of other and ill-defined sites | |  |
| B550.00 | | Malignant neoplasm of head, neck and face | |  |
| B550000 | | Malignant neoplasm of head NOS | |  |
| B550100 | | Malignant neoplasm of cheek NOS | |  |
| B550200 | | Malignant neoplasm of nose NOS | |  |
| B550300 | | Malignant neoplasm of jaw NOS | |  |
| B550400 | | Malignant neoplasm of neck NOS | |  |
| B550500 | | Malignant neoplasm of supraclavicular fossa NOS | |  |
| B550z00 | | Malignant neoplasm of head, neck and face NOS | |  |
| B551.00 | | Malignant neoplasm of thorax | |  |
| B551000 | | Malignant neoplasm of axilla NOS | |  |
| B551100 | | Malignant neoplasm of chest wall NOS | |  |
| B551200 | | Malignant neoplasm of intrathoracic site NOS | |  |
| B551z00 | | Malignant neoplasm of thorax NOS | |  |
| B552.00 | | Malignant neoplasm of abdomen | |  |
| B553.00 | | Malignant neoplasm of pelvis | |  |
| B553000 | | Malignant neoplasm of inguinal region NOS | |  |
| B553100 | | Malignant neoplasm of presacral region | |  |
| B553200 | | Malignant neoplasm of sacrococcygeal region | |  |
| B553z00 | | Malignant neoplasm of pelvis NOS | |  |
| B554.00 | | Malignant neoplasm of upper limb NOS | |  |
| B555.00 | | Malignant neoplasm of lower limb NOS | |  |
| B55y.00 | | Malignant neoplasm of other specified sites | |  |
| B55y000 | | Malignant neoplasm of back NOS | |  |
| B55y100 | | Malignant neoplasm of trunk NOS | |  |
| B55y200 | | Malignant neoplasm of flank NOS | |  |
| B55yz00 | | Malignant neoplasm of specified site NOS | |  |
| B55z.00 | | Malignant neoplasm of other and ill defined site NOS | |  |
| B56..00 | | Secondary and unspecified malignant neoplasm of lymph nodes | |  |
| B56..11 | | Lymph node metastases | |  |
| B560.00 | | Secondary and unspec malig neop lymph nodes head/face/neck | |  |
| B560000 | | Secondary and unspec malig neop of superficial parotid LN | |  |
| B560100 | | Secondary and unspec malignant neoplasm mastoid lymph nodes | |  |
| B560200 | | Secondary and unspec malig neop superficial cervical LN | |  |
| B560300 | | Secondary and unspec malignant neoplasm occipital lymph node | |  |
| B560400 | | Secondary and unspec malig neop deep parotid lymph nodes | |  |
| B560500 | | Secondary and unspec malig neop submandibular lymph nodes | |  |
| B560600 | | Secondary and unspec malig neop of facial lymph nodes | |  |
| B560700 | | Secondary and unspec malig neop submental lymph nodes | |  |
| B560800 | | Secondary and unspec malig neop anterior cervical LN | |  |
| B560900 | | Secondary and unspec malig neop deep cervical LN | |  |
| B560z00 | | Secondary unspec malig neop lymph nodes head/face/neck NOS | |  |
| B561.00 | | Secondary and unspec malig neop intrathoracic lymph nodes | |  |
| B561000 | | Secondary and unspec malig neop internal mammary lymph nodes | |  |
| B561100 | | Secondary and unspec malig neop intercostal lymph nodes | |  |
| B561200 | | Secondary and unspec malig neop diaphragmatic lymph nodes | |  |
| B561300 | | Secondary and unspec malig neop ant mediastinal lymph nodes | |  |
| B561400 | | Secondary and unspec malig neop post mediastinal lymph nodes | |  |
| B561500 | | Secondary and unspec malig neop paratracheal lymph nodes | |  |
| B561600 | | Secondary and unspec malig neop superfic tracheobronchial LN | |  |
| B561700 | | Secondary and unspec malig neop inferior tracheobronchial LN | |  |
| B561800 | | Secondary and unspec malig neop bronchopulmonary lymph nodes | |  |
| B561900 | | Secondary and unspec malig neop pulmonary lymph nodes | |  |
| B561z00 | | Secondary and unspec malig neop intrathoracic LN NOS | |  |
| B562.00 | | Secondary and unspec malig neop intra-abdominal lymph nodes | |  |
| B562000 | | Secondary and unspec malig neop coeliac lymph nodes | |  |
| B562100 | | Secondary and unspec malig neop superficial mesenteric LN | |  |
| B562200 | | Secondary and unspec malig neop inferior mesenteric LN | |  |
| B562300 | | Secondary and unspec malig neop common iliac lymph nodes | |  |
| B562400 | | Secondary and unspec malig neop external iliac lymph nodes | |  |
| B562z00 | | Secondary and unspec malig neop intra-abdominal LN NOS | |  |
| B563.00 | | Secondary and unspec malig neop axilla and upper limb LN | |  |
| B563000 | | Secondary and unspec malig neop axillary lymph nodes | |  |
| B563100 | | Secondary and unspec malig neop supratrochlear lymph nodes | |  |
| B563200 | | Secondary and unspec malig neop infraclavicular lymph nodes | |  |
| B563300 | | Secondary and unspec malig neop pectoral lymph nodes | |  |
| B563z00 | | Secondary and unspec malig neop axilla and upper limb LN NOS | |  |
| B564.00 | | Secondary and unspec malig neop inguinal and lower limb LN | |  |
| B564000 | | Secondary and unspec malig neop superficial inguinal LN | |  |
| B564100 | | Secondary and unspec malig neop deep inguinal lymph nodes | |  |
| B564z00 | | Secondary and unspec malig neop of inguinal and leg LN NOS | |  |
| B565.00 | | Secondary and unspec malig neop intrapelvic lymph nodes | |  |
| B565000 | | Secondary and unspec malig neop internal iliac lymph nodes | |  |
| B565200 | | Secondary and unspec malig neop circumflex iliac LN | |  |
| B565300 | | Secondary and unspec malig neop sacral lymph nodes | |  |
| B565z00 | | Secondary and unspec malig neop intrapelvic LN NOS | |  |
| B56y.00 | | Secondary and unspec malig neop lymph nodes multiple sites | |  |
| B56z.00 | | Secondary and unspec malig neop lymph nodes NOS | |  |
| B57..00 | | Secondary malig neop of respiratory and digestive systems | |  |
| B57..11 | | Metastases of respiratory and/or digestive systems | |  |
| B57..12 | | Secondary carcinoma of respiratory and/or digestive systems | |  |
| B570.00 | | Secondary malignant neoplasm of lung | |  |
| B571.00 | | Secondary malignant neoplasm of mediastinum | |  |
| B572.00 | | Secondary malignant neoplasm of pleura | |  |
| B573.00 | | Secondary malignant neoplasm of other respiratory organs | |  |
| B574.00 | | Secondary malignant neoplasm of small intestine and duodenum | |  |
| B574000 | | Secondary malignant neoplasm of duodenum | |  |
| B574100 | | Secondary malignant neoplasm of jejunum | |  |
| B574200 | | Secondary malignant neoplasm of ileum | |  |
| B574z00 | | Secondary malig neop of small intestine or duodenum NOS | |  |
| B575.00 | | Secondary malignant neoplasm of large intestine and rectum | |  |
| B575000 | | Secondary malignant neoplasm of colon | |  |
| B575100 | | Secondary malignant neoplasm of rectum | |  |
| B575z00 | | Secondary malig neop of large intestine or rectum NOS | |  |
| B576.00 | | Secondary malig neop of retroperitoneum and peritoneum | |  |
| B576000 | | Secondary malignant neoplasm of retroperitoneum | |  |
| B576100 | | Secondary malignant neoplasm of peritoneum | |  |
| B576200 | | Malignant ascites | |  |
| B576z00 | | Secondary malig neop of retroperitoneum or peritoneum NOS | |  |
| B577.00 | | Secondary malignant neoplasm of liver | |  |
| B577.11 | | Liver metastases | |  |
| B57y.00 | | Secondary malignant neoplasm of other digestive organ | |  |
| B57z.00 | | Secondary malig neop of respiratory or digestive system NOS | |  |
| B58..00 | | Secondary malignant neoplasm of other specified sites | |  |
| B58..11 | | Secondary carcinoma of other specified sites | |  |
| B580.00 | | Secondary malignant neoplasm of kidney | |  |
| B581.00 | | Secondary malignant neoplasm of other urinary organs | |  |
| B581000 | | Secondary malignant neoplasm of ureter | |  |
| B581100 | | Secondary malignant neoplasm of bladder | |  |
| B581200 | | Secondary malignant neoplasm of urethra | |  |
| B581z00 | | Secondary malignant neoplasm of other urinary organ NOS | |  |
| B582.00 | | Secondary malignant neoplasm of skin | |  |
| B582000 | | Secondary malignant neoplasm of skin of head | |  |
| B582100 | | Secondary malignant neoplasm of skin of face | |  |
| B582200 | | Secondary malignant neoplasm of skin of neck | |  |
| B582300 | | Secondary malignant neoplasm of skin of trunk | |  |
| B582400 | | Secondary malignant neoplasm of skin of shoulder and arm | |  |
| B582500 | | Secondary malignant neoplasm of skin of hip and leg | |  |
| B582600 | | Secondary malignant neoplasm of skin of breast | |  |
| B582z00 | | Secondary malignant neoplasm of skin NOS | |  |
| B583.00 | | Secondary malignant neoplasm of brain and spinal cord | |  |
| B583000 | | Secondary malignant neoplasm of brain | |  |
| B583100 | | Secondary malignant neoplasm of spinal cord | |  |
| B583200 | | Cerebral metastasis | |  |
| B583z00 | | Secondary malignant neoplasm of brain or spinal cord NOS | |  |
| B584.00 | | Secondary malignant neoplasm of other part of nervous system | |  |
| B585.00 | | Secondary malignant neoplasm of bone and bone marrow | |  |
| B585000 | | Pathological fracture due to metastatic bone disease | |  |
| B586.00 | | Secondary malignant neoplasm of ovary | |  |
| B587.00 | | Secondary malignant neoplasm of adrenal gland | |  |
| B58y.00 | | Secondary malignant neoplasm of other specified sites | |  |
| B58y000 | | Secondary malignant neoplasm of breast | |  |
| B58y100 | | Secondary malignant neoplasm of uterus | |  |
| B58y200 | | Secondary malignant neoplasm of cervix uteri | |  |
| B58y211 | | Secondary cancer of the cervix | |  |
| B58y300 | | Secondary malignant neoplasm of vagina | |  |
| B58y400 | | Secondary malignant neoplasm of vulva | |  |
| B58y411 | | Secondary cancer of the vulva | |  |
| B58y500 | | Secondary malignant neoplasm of prostate | |  |
| B58y600 | | Secondary malignant neoplasm of testis | |  |
| B58y700 | | Secondary malignant neoplasm of penis | |  |
| B58y800 | | Secondary malignant neoplasm of epididymis and vas deferens | |  |
| B58y900 | | Secondary malignant neoplasm of tongue | |  |
| B58yz00 | | Secondary malignant neoplasm of other specified site NOS | |  |
| B58z.00 | | Secondary malignant neoplasm of other specified site NOS | |  |
| B59..00 | | Malignant neoplasm of unspecified site | |  |
| B590.00 | | Disseminated malignancy NOS | |  |
| B590.11 | | Carcinomatosis | |  |
| B591.00 | | Other malignant neoplasm NOS | |  |
| B592.00 | | Malignant neoplasms of independent (primary) multiple sites | |  |
| B592X00 | | Kaposi's sarcoma of multiple organs | |  |
| B593.00 | | Primary malignant neoplasm of unknown site | |  |
| B594.00 | | Secondary malignant neoplasm of unknown site | |  |
| B595.00 | | Malignant tumour of unknown origin | |  |
| B59z.00 | | Malignant neoplasm of unspecified site NOS | |  |
| B59zX00 | | Kaposi's sarcoma, unspecified | |  |
| B5y..00 | | Malignant neoplasm of other and unspecified site OS | |  |
| B5z..00 | | Malignant neoplasm of other and unspecified site NOS | |  |
| B6...00 | | Malignant neoplasm of lymphatic and haemopoietic tissue | |  |
| B6...11 | | Malignant neoplasm of histiocytic tissue | |  |
| B60..00 | | Lymphosarcoma and reticulosarcoma | |  |
| B600.00 | | Reticulosarcoma | |  |
| B600000 | | Reticulosarcoma of unspecified site | |  |
| B600100 | | Reticulosarcoma of lymph nodes of head, face and neck | |  |
| B600300 | | Reticulosarcoma of intra-abdominal lymph nodes | |  |
| B600700 | | Reticulosarcoma of spleen | |  |
| B600z00 | | Reticulosarcoma NOS | |  |
| B601.00 | | Lymphosarcoma | |  |
| B601000 | | Lymphosarcoma of unspecified site | |  |
| B601100 | | Lymphosarcoma of lymph nodes of head, face and neck | |  |
| B601200 | | Lymphosarcoma of intrathoracic lymph nodes | |  |
| B601300 | | Lymphosarcoma of intra-abdominal lymph nodes | |  |
| B601500 | | Lymphosarcoma of lymph nodes of inguinal region and leg | |  |
| B601700 | | Lymphosarcoma of spleen | |  |
| B601800 | | Lymphosarcoma of lymph nodes of multiple sites | |  |
| B601z00 | | Lymphosarcoma NOS | |  |
| B602.00 | | Burkitt's lymphoma | |  |
| B602100 | | Burkitt's lymphoma of lymph nodes of head, face and neck | |  |
| B602200 | | Burkitt's lymphoma of intrathoracic lymph nodes | |  |
| B602300 | | Burkitt's lymphoma of intra-abdominal lymph nodes | |  |
| B602500 | | Burkitt's lymphoma of lymph nodes of inguinal region and leg | |  |
| B602z00 | | Burkitt's lymphoma NOS | |  |
| B60y.00 | | Other specified reticulosarcoma or lymphosarcoma | |  |
| B60z.00 | | Reticulosarcoma or lymphosarcoma NOS | |  |
| B61..00 | | Hodgkin's disease | |  |
| B61..11 | | Hodgkin lymphoma | |  |
| B610.00 | | Hodgkin's paragranuloma | |  |
| B610100 | | Hodgkin's paragranuloma of lymph nodes of head, face, neck | |  |
| B610300 | | Hodgkin's paragranuloma of intra-abdominal lymph nodes | |  |
| B611.00 | | Hodgkin's granuloma | |  |
| B611100 | | Hodgkin's granuloma of lymph nodes of head, face and neck | |  |
| B612.00 | | Hodgkin's sarcoma | |  |
| B612400 | | Hodgkin's sarcoma of lymph nodes of axilla and upper limb | |  |
| B613.00 | | Hodgkin's disease, lymphocytic-histiocytic predominance | |  |
| B613000 | | Hodgkin's, lymphocytic-histiocytic predominance unspec site | |  |
| B613100 | | Hodgkin's, lymphocytic-histiocytic pred of head, face, neck | |  |
| B613200 | | Hodgkin's, lymphocytic-histiocytic pred intrathoracic nodes | |  |
| B613300 | | Hodgkin's, lymphocytic-histiocytic pred intra-abdominal node | |  |
| B613500 | | Hodgkin's, lymphocytic-histiocytic pred inguinal and leg | |  |
| B613600 | | Hodgkin's, lymphocytic-histiocytic pred intrapelvic nodes | |  |
| B613700 | | Hodgkin's, lymphocytic-histiocytic predominance of spleen | |  |
| B613800 | | Hodgkin's, lymphocytic-histiocytic pred of multiple sites | |  |
| B613z00 | | Hodgkin's, lymphocytic-histiocytic predominance NOS | |  |
| B614.00 | | Hodgkin's disease, nodular sclerosis | |  |
| B614000 | | Hodgkin's disease, nodular sclerosis of unspecified site | |  |
| B614100 | | Hodgkin's nodular sclerosis of head, face and neck | |  |
| B614200 | | Hodgkin's nodular sclerosis of intrathoracic lymph nodes | |  |
| B614300 | | Hodgkin's nodular sclerosis of intra-abdominal lymph nodes | |  |
| B614400 | | Hodgkin's nodular sclerosis of lymph nodes of axilla and arm | |  |
| B614700 | | Hodgkin's disease, nodular sclerosis of spleen | |  |
| B614800 | | Hodgkin's nodular sclerosis of lymph nodes of multiple sites | |  |
| B614z00 | | Hodgkin's disease, nodular sclerosis NOS | |  |
| B615.00 | | Hodgkin's disease, mixed cellularity | |  |
| B615000 | | Hodgkin's disease, mixed cellularity of unspecified site | |  |
| B615100 | | Hodgkin's mixed cellularity of lymph nodes head, face, neck | |  |
| B615200 | | Hodgkin's mixed cellularity of intrathoracic lymph nodes | |  |
| B615500 | | Hodgkin's mixed cellularity of lymph nodes inguinal and leg | |  |
| B615z00 | | Hodgkin's disease, mixed cellularity NOS | |  |
| B616.00 | | Hodgkin's disease, lymphocytic depletion | |  |
| B616000 | | Hodgkin's lymphocytic depletion of unspecified site | |  |
| B616100 | | Hodgkin's lymphocytic depletion of head, face and neck | |  |
| B616400 | | Hodgkin's lymphocytic depletion lymph nodes axilla and arm | |  |
| B616500 | | Hodgkin's lymphocytic depletion lymph nodes inguinal and leg | |  |
| B616700 | | Hodgkin's disease, lymphocytic depletion of spleen | |  |
| B616800 | | Hodgkin's lymphocytic depletion lymph nodes multiple sites | |  |
| B616z00 | | Hodgkin's disease, lymphocytic depletion NOS | |  |
| B617.00 | | Nodular lymphocyte predominant Hodgkin lymphoma | |  |
| B618.00 | | Nodular sclerosis classical Hodgkin lymphoma | |  |
| B619.00 | | Mixed cellularity classical Hodgkin lymphoma | |  |
| B61B.00 | | Lymphocyte-rich classical Hodgkin lymphoma | |  |
| B61C.00 | | Other classical Hodgkin lymphoma | |  |
| B61z.00 | | Hodgkin's disease NOS | |  |
| B61z.11 | | Hodgkin lymphoma NOS | |  |
| B61z000 | | Hodgkin's disease NOS, unspecified site | |  |
| B61z100 | | Hodgkin's disease NOS of lymph nodes of head, face and neck | |  |
| B61z200 | | Hodgkin's disease NOS of intrathoracic lymph nodes | |  |
| B61z300 | | Hodgkin's disease NOS of intra-abdominal lymph nodes | |  |
| B61z400 | | Hodgkin's disease NOS of lymph nodes of axilla and arm | |  |
| B61z500 | | Hodgkin's disease NOS of lymph nodes inguinal region and leg | |  |
| B61z700 | | Hodgkin's disease NOS of spleen | |  |
| B61z800 | | Hodgkin's disease NOS of lymph nodes of multiple sites | |  |
| B61zz00 | | Hodgkin's disease NOS | |  |
| B62..00 | | Other malignant neoplasm of lymphoid and histiocytic tissue | |  |
| B620.00 | | Nodular lymphoma (Brill - Symmers disease) | |  |
| B620000 | | Nodular lymphoma of unspecified site | |  |
| B620100 | | Nodular lymphoma of lymph nodes of head, face and neck | |  |
| B620200 | | Nodular lymphoma of intrathoracic lymph nodes | |  |
| B620300 | | Nodular lymphoma of intra-abdominal lymph nodes | |  |
| B620400 | | Nodular lymphoma of lymph nodes of axilla and upper limb | |  |
| B620500 | | Nodular lymphoma of lymph nodes of inguinal region and leg | |  |
| B620800 | | Nodular lymphoma of lymph nodes of multiple sites | |  |
| B620z00 | | Nodular lymphoma NOS | |  |
| B621.00 | | Mycosis fungoides | |  |
| B621000 | | Mycosis fungoides of unspecified site | |  |
| B621300 | | Mycosis fungoides of intra-abdominal lymph nodes | |  |
| B621400 | | Mycosis fungoides of lymph nodes of axilla and upper limb | |  |
| B621500 | | Mycosis fungoides of lymph nodes of inguinal region and leg | |  |
| B621800 | | Mycosis fungoides of lymph nodes of multiple sites | |  |
| B621z00 | | Mycosis fungoides NOS | |  |
| B622.00 | | Sezary's disease | |  |
| B622z00 | | Sezary's disease NOS | |  |
| B623.00 | | Malignant histiocytosis | |  |
| B623000 | | Malignant histiocytosis of unspecified site | |  |
| B623100 | | Malignant histiocytosis of lymph nodes head, face and neck | |  |
| B623300 | | Malignant histiocytosis of intra-abdominal lymph nodes | |  |
| B623800 | | Malignant histiocytosis of lymph nodes of multiple sites | |  |
| B623z00 | | Malignant histiocytosis NOS | |  |
| B624.00 | | Leukaemic reticuloendotheliosis | |  |
| B624.11 | | Leukaemic reticuloendotheliosis | |  |
| B624.12 | | Hairy cell leukaemia | |  |
| B624000 | | Leukaemic reticuloendotheliosis of unspecified sites | |  |
| B624300 | | Leukaemic reticuloend of intra-abdominal lymph nodes | |  |
| B624z00 | | Leukaemic reticuloendotheliosis NOS | |  |
| B625.00 | | Letterer-Siwe disease | |  |
| B625.11 | | Histiocytosis X (acute, progressive) | |  |
| B625000 | | Letterer-Siwe disease of unspecified sites | |  |
| B625200 | | Letterer-Siwe disease of intrathoracic lymph nodes | |  |
| B625800 | | Letterer-Siwe disease of lymph nodes of multiple sites | |  |
| B625z00 | | Letterer-Siwe disease NOS | |  |
| B626.00 | | Malignant mast cell tumours | |  |
| B626000 | | Mast cell malignancy of unspecified site | |  |
| B626500 | | Mast cell malignancy of lymph nodes inguinal region and leg | |  |
| B626800 | | Mast cell malignancy of lymph nodes of multiple sites | |  |
| B626z00 | | Malignant mast cell tumour NOS | |  |
| B627.00 | | Non - Hodgkin's lymphoma | |  |
| B627.11 | | Non-Hodgkin lymphoma | |  |
| B627000 | | Follicular non-Hodgkin's small cleaved cell lymphoma | |  |
| B627100 | | Follicular non-Hodg mixed sml cleavd & lge cell lymphoma | |  |
| B627200 | | Follicular non-Hodgkin's large cell lymphoma | |  |
| B627300 | | Diffuse non-Hodgkin's small cell (diffuse) lymphoma | |  |
| B627400 | | Diffuse non-Hodgkin's small cleaved cell (diffuse) lymphoma | |  |
| B627500 | | Diffuse non-Hodgkin mixed sml & lge cell (diffuse) lymphoma | |  |
| B627600 | | Diffuse non-Hodgkin's immunoblastic (diffuse) lymphoma | |  |
| B627700 | | Diffuse non-Hodgkin's lymphoblastic (diffuse) lymphoma | |  |
| B627800 | | Diffuse non-Hodgkin's lymphoma undifferentiated (diffuse) | |  |
| B627900 | | Mucosa-associated lymphoma | |  |
| B627911 | | Maltoma | |  |
| B627A00 | | Diffuse non-Hodgkin's large cell lymphoma | |  |
| B627B00 | | Other types of follicular non-Hodgkin's lymphoma | |  |
| B627C00 | | Follicular non-Hodgkin's lymphoma | |  |
| B627C11 | | Follicular lymphoma NOS | |  |
| B627D00 | | Diffuse non-Hodgkin's centroblastic lymphoma | |  |
| B627E00 | | Diffuse large B-cell lymphoma | |  |
| B627F00 | | Extranod marg zone B-cell lymphom mucosa-assoc lymphoid tiss | |  |
| B627G00 | | Mediastinal (thymic) large B-cell lymphoma | |  |
| B627W00 | | Unspecified B-cell non-Hodgkin's lymphoma | |  |
| B627X00 | | Diffuse non-Hodgkin's lymphoma, unspecified | |  |
| B628.00 | | Follicular lymphoma | |  |
| B628000 | | Follicular lymphoma grade 1 | |  |
| B628100 | | Follicular lymphoma grade 2 | |  |
| B628200 | | Follicular lymphoma grade 3 | |  |
| B628300 | | Follicular lymphoma grade 3a | |  |
| B628400 | | Follicular lymphoma grade 3b | |  |
| B628500 | | Diffuse follicle centre lymphoma | |  |
| B628600 | | Cutaneous follicle centre lymphoma | |  |
| B628700 | | Other types of follicular lymphoma | |  |
| B629.00 | | Multifocal multisystemic dissem Langerhans-cell histiocytosi | |  |
| B62A.00 | | Sarcoma of dendritic cells | |  |
| B62B.00 | | Multifocal and unisystemic Langerhans-cell histiocytosis | |  |
| B62C.00 | | Unifocal Langerhans-cell histiocytosis | |  |
| B62D.00 | | Histiocytic sarcoma | |  |
| B62E.00 | | T/NK-cell lymphoma | |  |
| B62E100 | | Anaplastic large cell lymphoma, ALK-positive | |  |
| B62E200 | | Anaplastic large cell lymphoma, ALK-negative | |  |
| B62E300 | | Cutaneous T-cell lymphoma | |  |
| B62E400 | | Extranodal NK/T-cell lymphoma, nasal type | |  |
| B62E500 | | Hepatosplenic T-cell lymphoma | |  |
| B62E600 | | Enteropathy-associated T-cell lymphoma | |  |
| B62E700 | | Subcutaneous panniculitic T-cell lymphoma | |  |
| B62E800 | | Blastic NK-cell lymphoma | |  |
| B62E900 | | Angioimmunoblastic T-cell lymphoma | |  |
| B62EA00 | | Primary cutaneous CD30-positive T-cell proliferations | |  |
| B62Ew00 | | Other mature T/NK-cell lymphoma | |  |
| B62F.00 | | Nonfollicular lymphoma | |  |
| B62F.11 | | Non-follicular lymphoma | |  |
| B62F000 | | Small cell B-cell lymphoma | |  |
| B62F100 | | Mantle cell lymphoma | |  |
| B62F200 | | Lymphoblastic (diffuse) lymphoma | |  |
| B62Fy00 | | Other non-follicular lymphoma | |  |
| B62x.00 | | Malignant lymphoma otherwise specified | |  |
| B62x000 | | T-zone lymphoma | |  |
| B62x100 | | Lymphoepithelioid lymphoma | |  |
| B62x200 | | Peripheral T-cell lymphoma | |  |
| B62x400 | | Malignant reticulosis | |  |
| B62x500 | | Malignant immunoproliferative small intestinal disease | |  |
| B62x600 | | True histiocytic lymphoma | |  |
| B62xX00 | | Oth and unspecif peripheral & cutaneous T-cell lymphomas | |  |
| B62y.00 | | Malignant lymphoma NOS | |  |
| B62y000 | | Malignant lymphoma NOS of unspecified site | |  |
| B62y100 | | Malignant lymphoma NOS of lymph nodes of head, face and neck | |  |
| B62y200 | | Malignant lymphoma NOS of intrathoracic lymph nodes | |  |
| B62y300 | | Malignant lymphoma NOS of intra-abdominal lymph nodes | |  |
| B62y400 | | Malignant lymphoma NOS of lymph nodes of axilla and arm | |  |
| B62y500 | | Malignant lymphoma NOS of lymph node inguinal region and leg | |  |
| B62y600 | | Malignant lymphoma NOS of intrapelvic lymph nodes | |  |
| B62y700 | | Malignant lymphoma NOS of spleen | |  |
| B62y800 | | Malignant lymphoma NOS of lymph nodes of multiple sites | |  |
| B62yz00 | | Malignant lymphoma NOS | |  |
| B62z.00 | | Malignant neoplasms of lymphoid and histiocytic tissue NOS | |  |
| B62z000 | | Unspec malig neop lymphoid/histiocytic of unspecified site | |  |
| B62z100 | | Unspec malig neop lymphoid/histiocytic lymph node head/neck | |  |
| B62z200 | | Unspec malig neop lymphoid/histiocytic of intrathoracic node | |  |
| B62z300 | | Unspec malig neop lymphoid/histiocytic intra-abdominal nodes | |  |
| B62z400 | | Unspec malig neop lymphoid/histiocytic lymph node axilla/arm | |  |
| B62z500 | | Unspec malig neop lymphoid/histiocytic nodes inguinal/leg | |  |
| B62z600 | | Unspec malig neop lymphoid/histiocytic of intrapelvic nodes | |  |
| B62z800 | | Unspec malig neop lymphoid/histiocytic of multiple sites | |  |
| B62zz00 | | Lymphoid and histiocytic malignancy NOS | |  |
| B62zz11 | | Immunoproliferative neoplasm | |  |
| B63..00 | | Multiple myeloma and immunoproliferative neoplasms | |  |
| B630.00 | | Multiple myeloma | |  |
| B630.11 | | Kahler's disease | |  |
| B630.12 | | Myelomatosis | |  |
| B630000 | | Malignant plasma cell neoplasm, extramedullary plasmacytoma | |  |
| B630100 | | Solitary myeloma | |  |
| B630200 | | Plasmacytoma NOS | |  |
| B630300 | | Lambda light chain myeloma | |  |
| B630400 | | Solitary plasmacytoma | |  |
| B631.00 | | Plasma cell leukaemia | |  |
| B63y.00 | | Other immunoproliferative neoplasms | |  |
| B63z.00 | | Immunoproliferative neoplasm or myeloma NOS | |  |
| B64..00 | | Lymphoid leukaemia | |  |
| B64..11 | | Lymphatic leukaemia | |  |
| B640.00 | | Acute lymphoid leukaemia | |  |
| B640000 | | B-cell acute lymphoblastic leukaemia | |  |
| B641.00 | | Chronic lymphoid leukaemia | |  |
| B641.11 | | Chronic lymphatic leukaemia | |  |
| B641000 | | B-cell chronic lymphocytic leukaemia | |  |
| B641011 | | Chronic lymphocytic leukaemia of B-cell type | |  |
| B641100 | | Clinical stage A chronic lymphocytic leukaemia | |  |
| B641200 | | Clinical stage B chronic lymphocytic leukaemia | |  |
| B641300 | | Clinical stage C chronic lymphocytic leukaemia | |  |
| B642.00 | | Subacute lymphoid leukaemia | |  |
| B64y.00 | | Other lymphoid leukaemia | |  |
| B64y100 | | Prolymphocytic leukaemia | |  |
| B64y200 | | Adult T-cell leukaemia | |  |
| B64y300 | | B-cell prolymphocytic leukaemia | |  |
| B64y400 | | T-cell prolymphocytic leukaemia | |  |
| B64y411 | | Prolymphocytic leukaemia of T-cell type | |  |
| B64y500 | | Adult T-cell lymphoma/leukaemia (HTLV-1-associated) | |  |
| B64yz00 | | Other lymphoid leukaemia NOS | |  |
| B64z.00 | | Lymphoid leukaemia NOS | |  |
| B65..00 | | Myeloid leukaemia | |  |
| B650.00 | | Acute myeloid leukaemia | |  |
| B651.00 | | Chronic myeloid leukaemia | |  |
| B651.11 | | Chronic granulocytic leukaemia | |  |
| B651000 | | Chronic eosinophilic leukaemia | |  |
| B651100 | | Chronic myeloid leukaemia, BCR/ABL positive | |  |
| B651200 | | Chronic neutrophilic leukaemia | |  |
| B651300 | | Atypical chronic myeloid leukaemia, BCR/ABL negative | |  |
| B651z00 | | Chronic myeloid leukaemia NOS | |  |
| B652.00 | | Subacute myeloid leukaemia | |  |
| B653.00 | | Myeloid sarcoma | |  |
| B653000 | | Chloroma | |  |
| B653100 | | Granulocytic sarcoma | |  |
| B654.00 | | Acute myeloblastic leukaemia | |  |
| B65y100 | | Acute promyelocytic leukaemia | |  |
| B65yz00 | | Other myeloid leukaemia NOS | |  |
| B65z.00 | | Myeloid leukaemia NOS | |  |
| B66..00 | | Monocytic leukaemia | |  |
| B66..11 | | Histiocytic leukaemia | |  |
| B66..12 | | Monoblastic leukaemia | |  |
| B660.00 | | Acute monocytic leukaemia | |  |
| B661.00 | | Chronic monocytic leukaemia | |  |
| B662.00 | | Subacute monocytic leukaemia | |  |
| B663.00 | | Acute monoblastic leukaemia | |  |
| B66y.00 | | Other monocytic leukaemia | |  |
| B66yz00 | | Other monocytic leukaemia NOS | |  |
| B66z.00 | | Monocytic leukaemia NOS | |  |
| B67..00 | | Other specified leukaemia | |  |
| B670.00 | | Acute erythraemia and erythroleukaemia | |  |
| B670.11 | | Di Guglielmo's disease | |  |
| B671.00 | | Chronic erythraemia | |  |
| B671.11 | | Heilmeyer - Schoner disease | |  |
| B672.00 | | Megakaryocytic leukaemia | |  |
| B672.11 | | Thrombocytic leukaemia | |  |
| B673.00 | | Mast cell leukaemia | |  |
| B674.00 | | Acute panmyelosis | |  |
| B675.00 | | Acute myelofibrosis | |  |
| B676.00 | | Acute erythroid leukaemia | |  |
| B677.00 | | Myelodysplastic and myeloproliferative disease | |  |
| B67y.00 | | Other and unspecified leukaemia | |  |
| B67y000 | | Lymphosarcoma cell leukaemia | |  |
| B67yz00 | | Other and unspecified leukaemia NOS | |  |
| B67z.00 | | Other specified leukaemia NOS | |  |
| B68..00 | | Leukaemia of unspecified cell type | |  |
| B680.00 | | Acute leukaemia NOS | |  |
| B681.00 | | Chronic leukaemia NOS | |  |
| B682.00 | | Subacute leukaemia NOS | |  |
| B68y.00 | | Other leukaemia of unspecified cell type | |  |
| B68z.00 | | Leukaemia NOS | |  |
| B69..00 | | Myelomonocytic leukaemia | |  |
| B690.00 | | Acute myelomonocytic leukaemia | |  |
| B691.00 | | Chronic myelomonocytic leukaemia | |  |
| B692.00 | | Subacute myelomonocytic leukaemia | |  |
| B693.00 | | Juvenile myelomonocytic leukaemia | |  |
| B6y..00 | | Malignant neoplasm lymphatic or haematopoietic tissue OS | |  |
| B6y0.00 | | Myeloproliferative disorder | |  |
| B6y0.11 | | Myeloproliferative disease | |  |
| B6y1.00 | | Myelosclerosis with myeloid metaplasia | |  |
| B6y1.12 | | Osteomyelofibrosis | |  |
| B6z..00 | | Malignant neoplasm lymphatic or haematopoietic tissue NOS | |  |
| B6z0.00 | | Kaposi's sarcoma of lymph nodes | |  |
| B8...00 | | Carcinoma in situ | |  |
| B8...11 | | Bowen's disease | |  |
| B80..00 | | Carcinoma in situ of digestive organs | |  |
| B80..11 | | Ca-in-situ of G.I. tract | |  |
| B800.00 | | Carcinoma in situ of lip, oral cavity and pharynx | |  |
| B800.11 | | Carcinoma in situ of oral cavity | |  |
| B800.12 | | Carcinoma in situ of pharynx | |  |
| B800000 | | Carcinoma in situ of lip | |  |
| B800100 | | Carcinoma in situ of tongue | |  |
| B800200 | | Carcinoma in situ of salivary glands | |  |
| B800300 | | Carcinoma in situ of gums | |  |
| B800400 | | Carcinoma in situ of floor of mouth | |  |
| B800500 | | Carcinoma in situ of cheek | |  |
| B800600 | | Carcinoma in situ of palate | |  |
| B800700 | | Carcinoma in situ of nasopharynx | |  |
| B800800 | | Carcinoma in situ of oropharynx | |  |
| B800900 | | Carcinoma in situ of hypopharynx | |  |
| B800z00 | | Carcinoma in situ of lip, oral cavity and pharynx NOS | |  |
| B801.00 | | Carcinoma in situ of oesophagus | |  |
| B801000 | | Carcinoma in situ of upper 1/3 oesophagus | |  |
| B801100 | | Carcinoma in situ of middle 1/3 oesophagus | |  |
| B801200 | | Carcinoma in situ of lower 1/3 oesophagus | |  |
| B801z00 | | Carcinoma in situ of oesophagus NOS | |  |
| B802.00 | | Carcinoma in situ of stomach | |  |
| B802000 | | Carcinoma in situ of cardia of stomach | |  |
| B802100 | | Carcinoma in situ of fundus of stomach | |  |
| B802200 | | Carcinoma in situ of body of stomach | |  |
| B802300 | | Carcinoma in situ of pyloric antrum | |  |
| B802400 | | Carcinoma in situ of pyloric canal | |  |
| B802z00 | | Carcinoma in situ of stomach NOS | |  |
| B803.00 | | Carcinoma in situ of colon | |  |
| B803000 | | Carcinoma in situ of hepatic flexure of colon | |  |
| B803100 | | Carcinoma in situ of transverse colon | |  |
| B803200 | | Carcinoma in situ of descending colon | |  |
| B803300 | | Carcinoma in situ of sigmoid colon | |  |
| B803400 | | Carcinoma in situ of caecum | |  |
| B803500 | | Carcinoma in situ of appendix | |  |
| B803600 | | Carcinoma in situ of ascending colon | |  |
| B803700 | | Carcinoma in situ of splenic flexure of colon | |  |
| B803z00 | | Carcinoma in situ of colon NOS | |  |
| B804.00 | | Carcinoma in situ of rectum and rectosigmoid junction | |  |
| B804000 | | Carcinoma in situ of rectosigmoid junction | |  |
| B804100 | | Carcinoma in situ of rectum | |  |
| B804z00 | | Carcinoma in situ of rectum or rectosigmoid junction NOS | |  |
| B805.00 | | Carcinoma in situ of anal canal | |  |
| B806.00 | | Carcinoma in situ of anus NOS | |  |
| B807.00 | | Carcinoma in situ of other and unspecified small intestine | |  |
| B807000 | | Carcinoma in situ of duodenum | |  |
| B807100 | | Carcinoma in situ of jejunum | |  |
| B807200 | | Carcinoma in situ of ileum | |  |
| B807300 | | Carcinoma in situ of Meckel's diverticulum | |  |
| B807z00 | | Carcinoma in situ other and unspecified small intestine NOS | |  |
| B808.00 | | Carcinoma in situ of liver and biliary system | |  |
| B808.11 | | Carcinoma in situ of biliary system | |  |
| B808000 | | Carcinoma in situ of liver | |  |
| B808100 | | Carcinoma in situ of intrahepatic bile ducts | |  |
| B808200 | | Carcinoma in situ of hepatic duct | |  |
| B808300 | | Carcinoma in situ of gall bladder | |  |
| B808400 | | Carcinoma in situ of cystic duct | |  |
| B808500 | | Carcinoma in situ of common bile duct | |  |
| B808600 | | Carcinoma in situ of ampulla of Vater | |  |
| B808z00 | | Carcinoma in situ of liver or biliary system NOS | |  |
| B80z.00 | | Carcinoma in situ of other and unspecified digestive organs | |  |
| B80z000 | | Carcinoma in situ of pancreas | |  |
| B80z100 | | Carcinoma in situ of spleen | |  |
| B81..00 | | Carcinoma in situ of respiratory system | |  |
| B810.00 | | Carcinoma in situ of larynx | |  |
| B810000 | | Carcinoma in situ of thyroid cartilage | |  |
| B810100 | | Carcinoma in situ of cricoid cartilage | |  |
| B810200 | | Carcinoma in situ of epiglottis | |  |
| B810300 | | Carcinoma in situ of arytenoid cartilage | |  |
| B810600 | | Carcinoma in situ of aryepiglottic fold | |  |
| B810700 | | Carcinoma in situ of vestibular fold | |  |
| B810800 | | Carcinoma in situ of vocal fold - glottis | |  |
| B810811 | | Carcinoma in situ of glottis | |  |
| B810z00 | | Carcinoma in situ of larynx NOS | |  |
| B811.00 | | Carcinoma in situ of trachea | |  |
| B812.00 | | Carcinoma in situ of bronchus and lung | |  |
| B812000 | | Carcinoma in situ of carina of bronchus | |  |
| B812100 | | Carcinoma in situ of main bronchus | |  |
| B812200 | | Carcinoma in situ of upper lobe bronchus and lung | |  |
| B812300 | | Carcinoma in situ of middle lobe bronchus and lung | |  |
| B812400 | | Carcinoma in situ of lower lobe bronchus and lung | |  |
| B812z00 | | Carcinoma in situ of bronchus or lung NOS | |  |
| B81y.00 | | Carcinoma in situ of other specified part respiratory system | |  |
| B81y.11 | | Carcinoma in situ of nasal sinuses | |  |
| B81y000 | | Carcinoma in situ of pleura | |  |
| B81y100 | | Carcinoma in situ of nasal cavity | |  |
| B81y400 | | Carcinoma in situ of Eustachian tube | |  |
| B81y500 | | Carcinoma in situ of mastoid air cells | |  |
| B81y600 | | Carcinoma in situ of maxillary sinus | |  |
| B81y700 | | Carcinoma in situ of ethmoidal sinus | |  |
| B81y900 | | Carcinoma in situ of sphenoidal sinus | |  |
| B81yz00 | | Carcinoma in situ of specified parts respiratory system NOS | |  |
| B81z.00 | | Carcinoma in situ of respiratory organ NOS | |  |
| B82..00 | | Carcinoma in situ of skin | |  |
| B820.00 | | Carcinoma in situ of skin of lip | |  |
| B821.00 | | Carcinoma in situ of skin of eyelid including canthus | |  |
| B822.00 | | Carcinoma in situ skin of ear and external auricular canal | |  |
| B822.11 | | Carcinoma in situ of ear | |  |
| B822000 | | Carcinoma in situ of skin of auricle | |  |
| B822z00 | | Carcinoma in situ skin of ear/external auricular canal NOS | |  |
| B823.00 | | Carcinoma in situ of skin of other parts of face | |  |
| B823000 | | Carcinoma in situ of skin of forehead skin | |  |
| B823100 | | Carcinoma in situ of skin of eyebrow | |  |
| B823300 | | Carcinoma in situ of skin of cheek | |  |
| B823400 | | Carcinoma in situ of skin of nose | |  |
| B823500 | | Carcinoma in situ of skin of temple | |  |
| B823600 | | Carcinoma in situ of skin of jaw | |  |
| B823z00 | | Carcinoma in situ of skin of other parts of face NOS | |  |
| B824.00 | | Carcinoma in situ of scalp and skin of neck | |  |
| B824000 | | Carcinoma in situ of scalp | |  |
| B824100 | | Carcinoma in situ of skin of neck | |  |
| B825.00 | | Carcinoma in situ of skin of trunk, excluding scrotum | |  |
| B825000 | | Carcinoma in situ of skin of breast | |  |
| B825100 | | Carcinoma in situ of skin of chest wall NOS | |  |
| B825200 | | Carcinoma in situ of skin of axilla | |  |
| B825300 | | Carcinoma in situ of skin of back | |  |
| B825400 | | Carcinoma in situ of skin of abdominal wall | |  |
| B825500 | | Carcinoma in situ of skin of groin | |  |
| B825600 | | Carcinoma in situ of skin of perineum | |  |
| B825700 | | Carcinoma in situ of skin of buttock | |  |
| B825800 | | Carcinoma in situ of perianal skin | |  |
| B825z00 | | Carcinoma in situ of skin of trunk NOS | |  |
| B826.00 | | Carcinoma in situ of skin of upper limb and shoulder | |  |
| B826000 | | Carcinoma in situ of skin of shoulder | |  |
| B826100 | | Carcinoma in situ of skin of upper arm | |  |
| B826200 | | Carcinoma in situ of skin of lower arm | |  |
| B826300 | | Carcinoma in situ of skin of hand | |  |
| B826z00 | | Carcinoma in situ of skin of upper limb or shoulder NOS | |  |
| B827.00 | | Carcinoma in situ of skin of lower limb and hip | |  |
| B827.11 | | Carcinoma in situ of skin of leg | |  |
| B827000 | | Carcinoma in situ of skin of hip | |  |
| B827100 | | Carcinoma in situ of skin of thigh | |  |
| B827200 | | Carcinoma in situ of skin of knee | |  |
| B827300 | | Carcinoma in situ of skin of lower leg | |  |
| B827400 | | Carcinoma in situ of skin of foot | |  |
| B827z00 | | Carcinoma in situ of skin of lower limb or hip NOS | |  |
| B828.00 | | Melanoma in situ of skin | |  |
| B828000 | | Melanoma in situ of lip | |  |
| B828100 | | Melanoma in situ of eyelid, including canthus | |  |
| B828200 | | Melanoma in situ of ear and external auricular canal | |  |
| B828300 | | Melanoma in situ of scalp and neck | |  |
| B828400 | | Melanoma in situ of trunk | |  |
| B828500 | | Melanoma in situ of upper limb, including shoulder | |  |
| B828600 | | Melanoma in situ of lower limb, including hip | |  |
| B828700 | | Melanoma in situ of scalp | |  |
| B828800 | | Melanoma in situ of back of hand | |  |
| B828900 | | Melanoma in situ of back | |  |
| B828W00 | | Melanoma in situ, unspecified | |  |
| B828X00 | | Melanoma in situ of other and unspecified parts of face | |  |
| B82y.00 | | Carcinoma in situ of other specified sites of skin | |  |
| B82z.00 | | Carcinoma in situ of skin NOS | |  |
| B83..00 | | Carcinoma in situ of breast and genitourinary system | |  |
| B830.00 | | Carcinoma in situ of breast | |  |
| B830000 | | Lobular carcinoma in situ of breast | |  |
| B830100 | | Intraductal carcinoma in situ of breast | |  |
| B831.00 | | Carcinoma in situ of cervix uteri | |  |
| B831.11 | | CIN III - carcinoma in situ of cervix | |  |
| B831.12 | | Cervical intraepithelial neoplasia | |  |
| B831.13 | | Cervical intraepithelial neoplasia grade III | |  |
| B831000 | | Carcinoma in situ of endocervix | |  |
| B831100 | | Carcinoma in situ of exocervix | |  |
| B832.00 | | Carcinoma in situ of other and unspecified parts of uterus | |  |
| B832.11 | | Carcinoma in situ of body of uterus | |  |
| B832000 | | Carcinoma in situ of endometrium | |  |
| B833.00 | | Carcinoma in situ other and unspecified female genital organ | |  |
| B833000 | | Carcinoma in situ of ovary | |  |
| B833100 | | Carcinoma in situ of fallopian tube | |  |
| B833200 | | Carcinoma in situ of vagina | |  |
| B833300 | | Carcinoma in situ of vulva | |  |
| B833z00 | | Carcinoma in situ of female genital organs NOS | |  |
| B834.00 | | Carcinoma in situ of prostate | |  |
| B835.00 | | Carcinoma in situ of penis | |  |
| B836.00 | | Carcinoma in situ other and unspecified male genital organs | |  |
| B836000 | | Carcinoma in situ of testis | |  |
| B836300 | | Carcinoma in situ of scrotum | |  |
| B836z00 | | Carcinoma in situ of male genital organs NOS | |  |
| B837.00 | | Carcinoma in situ of bladder | |  |
| B83z.00 | | Carcinoma in situ of urinary organs NOS | |  |
| B8y..00 | | Carcinoma in situ of other and unspecified sites | |  |
| B8y0.00 | | Carcinoma in situ of eye | |  |
| B8yy.00 | | Carcinoma in situ of other specified site | |  |
| B8yy000 | | Carcinoma in situ of thyroid gland | |  |
| B8yy100 | | Carcinoma in situ of adrenal gland | |  |
| B8yy200 | | Carcinoma in situ of parathyroid gland | |  |
| B8yy300 | | Carcinoma in situ of pituitary gland | |  |
| B8yyz00 | | Carcinoma in situ of other specified site NOS | |  |
| B8z..00 | | Carcinoma in situ NOS | |  |
| Byu0.00 | | [X]Malignant neoplasm of lip, oral cavity and pharynx | |  |
| Byu1.00 | | [X]Malignant neoplasm of digestive organs | |  |
| Byu1100 | | [X]Other specified carcinomas of liver | |  |
| Byu1200 | | [X]Malignant neoplasm of intestinal tract, part unspecified | |  |
| Byu1300 | | [X]Malignant neoplsm/ill-defin sites within digestive system | |  |
| Byu2.00 | | [X]Malignant neoplasm of respiratory and intrathoracic orga | |  |
| Byu2000 | | [X]Malignant neoplasm of bronchus or lung, unspecified | |  |
| Byu2100 | | [X]Malignant neoplasm/overlap lesion/heart,mediastinm+pleura | |  |
| Byu2200 | | [X]Malignant neoplasm/upper resp tract, part unspecified | |  |
| Byu2300 | | [X]Malignant neopl/overlapping les/resp+intrathoracic organs | |  |
| Byu2400 | | [X]Malignant neoplasm/ill-defined sites within resp system | |  |
| Byu2500 | | [X]Malignant neoplasm of mediastinum, part unspecified | |  |
| Byu3.00 | | [X]Malignant neoplasm of bone and articular cartilage | |  |
| Byu3100 | | [X]Malignant neoplasm/bones+articular cartilage/limb,unspfd | |  |
| Byu3200 | | [X]Malignant neoplasm/overlap lesion/bone+articulr cartilage | |  |
| Byu3300 | | [X]Malignant neoplasm/bone+articular cartilage, unspecified | |  |
| Byu4.00 | | [X]Melanoma and other malignant neoplasms of skin | |  |
| Byu4000 | | [X]Malignant melanoma of other+unspecified parts of face | |  |
| Byu4100 | | [X]Malignant melanoma of skin, unspecified | |  |
| Byu4200 | | [X]Oth malignant neoplasm/skin of oth+unspecfd parts of face | |  |
| Byu4300 | | [X]Malignant neoplasm of skin, unspecified | |  |
| Byu5.00 | | [X]Malignant neoplasm of mesothelial and soft tissue | |  |
| Byu5000 | | [X]Mesothelioma of other sites | |  |
| Byu5011 | | [X]Mesothelioma of lung | |  |
| Byu5100 | | [X]Mesothelioma, unspecified | |  |
| Byu5300 | | [X]Kaposi's sarcoma, unspecified | |  |
| Byu5400 | | [X]Malignant neoplasm/peripheral nerves of trunk,unspecified | |  |
| Byu5500 | | [X]Mal neoplasm/overlap les/periph nerv+autonomic nerv systm | |  |
| Byu5700 | | [X]Malignant neoplasm of peritoneum, unspecified | |  |
| Byu5800 | | [X]Mal neoplasm/connective+soft tissue of trunk,unspecified | |  |
| Byu5900 | | [X]Malignant neoplasm/connective + soft tissue,unspecified | |  |
| Byu5A00 | | [X]Malignant neoplasm overlapping lesion of skin | |  |
| Byu5B00 | | [X]Kaposi's sarcoma of other sites | |  |
| Byu6.00 | | [X]Malignant neoplasm of breast | |  |
| Byu7.00 | | [X]Malignant neoplasm of female genital organs | |  |
| Byu7000 | | [X]Malignant neoplasm of uterine adnexa, unspecified | |  |
| Byu7100 | | [X]Malignant neoplasm/other specified female genital organs | |  |
| Byu7300 | | [X]Malignant neoplasm of female genital organ, unspecified | |  |
| Byu8.00 | | [X]Malignant neoplasm of male genital organs | |  |
| Byu8000 | | [X]Malignant neoplasm/other specified male genital organs | |  |
| Byu8200 | | [X]Malignant neoplasm of male genital organ, unspecified | |  |
| Byu9.00 | | [X]Malignant neoplasm of urinary tract | |  |
| Byu9000 | | [X]Malignant neoplasm of urinary organ, unspecified | |  |
| ByuA.00 | | [X]Malignant neoplasm of eye, brain and other parts of cent | |  |
| ByuA000 | | [X]Malignant neoplasm/other and unspecified cranial nerves | |  |
| ByuA100 | | [X]Malignant neoplasm/central nervous system, unspecified | |  |
| ByuA200 | | [X]Malignant neoplasm of meninges, unspecified | |  |
| ByuA300 | | [X]Malig neopl, overlap lesion brain & other part of CNS | |  |
| ByuB.00 | | [X]Malignant neoplasm of thyroid and other endocrine glands | |  |
| ByuB100 | | [X]Malignant neoplasm of endocrine gland, unspecified | |  |
| ByuC.00 | | [X]Malignant neoplasm of ill-defined, secondary and unspeci | |  |
| ByuC000 | | [X]Malignant neoplasm of other specified sites | |  |
| ByuC100 | | [X]Malignant neoplasm/overlap lesion/other+ill-defined sites | |  |
| ByuC200 | | [X]2ndry+unspcf malignant neoplasm lymph nodes/multi regions | |  |
| ByuC300 | | [X]Secondary malignant neoplasm/oth+unspc respiratory organs | |  |
| ByuC400 | | [X]Secondary malignant neoplasm/oth+unspcfd digestive organs | |  |
| ByuC500 | | [X]2ndry malignant neoplasm/bladder+oth+unsp urinary organs | |  |
| ByuC600 | | [X]2ndry malignant neoplasm/oth+unspec parts/nervous system | |  |
| ByuC700 | | [X]Secondary malignant neoplasm of other specified sites | |  |
| ByuC800 | | [X]Malignant neoplasm without specification of site | |  |
| ByuD.00 | | [X]Malignant neoplasms of lymphoid, haematopoietic and rela | |  |
| ByuD000 | | [X]Other Hodgkin's disease | |  |
| ByuD100 | | [X]Other types of follicular non-Hodgkin's lymphoma | |  |
| ByuD200 | | [X]Other types of diffuse non-Hodgkin's lymphoma | |  |
| ByuD300 | | [X]Other specified types of non-Hodgkin's lymphoma | |  |
| ByuD400 | | [X]Other malignant immunoproliferative diseases | |  |
| ByuD500 | | [X]Other lymphoid leukaemia | |  |
| ByuD600 | | [X]Other myeloid leukaemia | |  |
| ByuD700 | | [X]Other monocytic leukaemia | |  |
| ByuD800 | | [X]Other specified leukaemias | |  |
| ByuD900 | | [X]Other leukaemia of unspecified cell type | |  |
| ByuDA00 | | [X]Oth spcf mal neoplsm/lymphoid,haematopoietic+rltd tissue | |  |
| ByuDB00 | | [X]Mal neoplasm/lymphoid,haematopoietic+related tissu,unspcf | |  |
| ByuDC00 | | [X]Diffuse non-Hodgkin's lymphoma, unspecified | |  |
| ByuDD00 | | [X]Oth and unspecif peripheral & cutaneous T-cell lymphomas | |  |
| ByuDE00 | | [X]Unspecified B-cell non-Hodgkin's lymphoma | |  |
| ByuDF00 | | [X]Non-Hodgkin's lymphoma, unspecified type | |  |
| ByuDF11 | | [X]Non-Hodgkin's lymphoma NOS | |  |
| ByuE.00 | | [X]Malignant neoplasms/independent (primary) multiple sites | |  |
| ByuE000 | | [X]Malignant neoplasms/independent(primary)multiple sites | |  |
| ByuF100 | | [X]Carcinoma in situ of other specified digestive organs | |  |
| ByuF300 | | [X]Carcinoma in situ of other parts of respiratory system | |  |
| ByuF600 | | [X]Melanoma in situ of other sites | |  |
| ByuF900 | | [X]Carcinoma in situ of skin, unspecified | |  |
| ByuFA00 | | [X]Carcinoma in situ of other parts of cervix | |  |
| ByuFC00 | | [X]Carcinoma in situ of oth+unspecified male genital organs | |  |
| ByuFF00 | | [X]Melanoma in situ, unspecified | |  |
| ByuFG00 | | [X]Other carcinoma in situ of breast | |  |
| 142..11 | | H/O: cancer | |  |
| 1427000 | | H/O: prostate cancer | |  |
| 4M0..00 | | Gleason grading of prostate cancer | |  |
| 1O0..00 | | Cancer confirmed | |  |
| 8BAD000 | | Cancer chemotherapy | |  |
| 8BC6.00 | | Cancer treatment started | |  |
| 8BCF.00 | | Cancer hospital treatment completed | |  |
| 9OkC.00 | | Patient on regional cancer register | |  |
| A788600 | | Human immunodeficiency virus with secondary cancers | |  |
| B....11 | | Cancers | |  |
| 142..00 | | H/O: malignant neoplasm (*) | |  |
| A788W00 | | HIV disease resulting in unspecified malignant neoplasm | |  |
| A789800 | | HIV disease resulting in multiple malignant neoplasms | |  |
| AyuC800 | | [X]HIV disease resulting in other malignant neoplasms | |  |
| AyuC900 | | [X]HIV disease resulting in unspecified malignant neoplasm | |  |
| ZV10.00 | | [V]Personal history of malignant neoplasm | |  |
| ZV10011 | | [V]Personal history of malignant neoplasm of anus | |  |
| ZV10014 | | [V]Personal history of malignant neoplasm of large intestine | |  |
| ZV10015 | | [V]Personal history of malignant neoplasm of liver | |  |
| ZV10016 | | [V]Personal history of malignant neoplasm of oesophagus | |  |
| ZV10017 | | [V]Personal history of malignant neoplasm of rectum | |  |
| ZV10018 | | [V]Personal history of malignant neoplasm of stomach | |  |
| ZV10019 | | [V]Personal history of malignant neoplasm of tongue | |  |
| ZV10111 | | [V]Personal history of malignant neoplasm of bronchus | |  |
| ZV10112 | | [V]Personal history of malignant neoplasm of lung | |  |
| ZV10211 | | [V]Personal history of malignant neoplasm - accessory sinus | |  |
| ZV10212 | | [V]Personal history of malignant neoplasm of larynx | |  |
| ZV10214 | | [V]Personal history of malignant neoplasm of nose | |  |
| ZV10300 | | [V]Personal history of malignant neoplasm of breast | |  |
| ZV10400 | | [V]Personal history of malignant neoplasm of genital organ | |  |
| ZV10411 | | [V]Personal history of malignant neoplasm of cervix uteri | |  |
| ZV10412 | | [V]Personal history of malignant neoplasm of genital organ | |  |
| ZV10414 | | [V]Personal history of malignant neoplasm of ovary | |  |
| ZV10415 | | [V]Personal history of malignant neoplasm of prostate | |  |
| ZV10416 | | [V]Personal history of malignant neoplasm of testis | |  |
| ZV10417 | | [V]Personal history of malignant neoplasm of uterine body | |  |
| ZV10500 | | [V]Personal history of malignant neoplasm of urinary organ | |  |
| ZV10511 | | [V]Personal history of malignant neoplasm of bladder | |  |
| ZV10512 | | [V]Personal history of malignant neoplasm of kidney | |  |
| ZV10513 | | [V]Personal history of malignant neoplasm of kidney | |  |
| ZV10y00 | | [V]Personal history of other specified malignant neoplasm | |  |
| ZV10y11 | | [V]Personal history of malignant neoplasm of bone | |  |
| ZV10y12 | | [V]Personal history of malignant neoplasm of brain | |  |
| ZV10y13 | | [V]Personal history of malignant neoplasm of eye | |  |
| ZV10y14 | | [V]Personal history of malignant neoplasm of skin | |  |
| ZV10y15 | | [V]Personal history of malignant neoplasm of thyroid | |  |
| ZV10y16 | | [V]Personal history of malignant neoplasm of tongue | |  |
| ZV10z00 | | [V]Personal history of unspecified malignant neoplasm | |  |
| ZV67600 | | [V]Follow-up examination aft surgery for malignant neoplasm | |  |
| ZV67700 | | [V]Follow-up exam after radiotherapy for malignant neoplasm | |  |
| ZV67A00 | | [V]Folow-up exam aft other treatment for malignant neoplasm | |  |
| ZV67B00 | | [V]Folow-up exam aft unspec treatment for malignant neoplasm | |  |
| 142..12 | | H/O: carcinoma | |  |
| 44a4.00 | | Squamous cell carcinoma antigen level | |  |
| 4K2M.00 | | Crv smr - hi grade dyskaryosis? invasive squamous carcinoma | |  |
| 7G05D00 | | Excision biopsy of basal cell carcinoma | |  |
| B911013 | | Choriocarcinoma | |  |
| BB11.00 | | [M]Carcinoma in situ NOS | |  |
| BB11.11 | | [M]Intraepithelial carcinoma NOS | |  |
| BB12.00 | | [M]Carcinoma NOS | |  |
| BB13.00 | | [M]Carcinoma, metastatic, NOS | |  |
| BB13.11 | | [M]Secondary carcinoma | |  |
| BB14.00 | | [M]Carcinomatosis | |  |
| BB17.00 | | [M]Large cell carcinoma NOS | |  |
| BB18.00 | | [M]Carcinoma, undifferentiated type, NOS | |  |
| BB19.00 | | [M]Carcinoma, anaplastic type, NOS | |  |
| BB1A.00 | | [M]Pleomorphic carcinoma | |  |
| BB1B.00 | | [M]Giant cell and spindle cell carcinoma | |  |
| BB1C.00 | | [M]Giant cell carcinoma | |  |
| BB1D.00 | | [M]Spindle cell carcinoma | |  |
| BB1E.00 | | [M]Pseudosarcomatous carcinoma | |  |
| BB1F.00 | | [M]Polygonal cell carcinoma | |  |
| BB1G.00 | | [M]Spheroidal cell carcinoma | |  |
| BB1J.00 | | [M]Small cell carcinoma NOS | |  |
| BB1J.12 | | [M]Round cell carcinoma | |  |
| BB1K.00 | | [M]Oat cell carcinoma | |  |
| BB1L.00 | | [M]Small cell carcinoma, fusiform cell type | |  |
| BB1M.00 | | [M]Small cell carcinoma, intermediate cell | |  |
| BB1N.00 | | [M]Small cell-large cell carcinoma | |  |
| BB1P.00 | | [M]Non-small cell carcinoma | |  |
| BB21.00 | | [M]Papillary carcinoma in situ | |  |
| BB22.00 | | [M]Papillary carcinoma NOS | |  |
| BB24.00 | | [M]Verrucous carcinoma NOS | |  |
| BB24.11 | | [M]Verrucous epidermoid carcinoma | |  |
| BB24.12 | | [M]Verrucous squamous cell carcinoma | |  |
| BB26.00 | | [M]Papillary squamous cell carcinoma | |  |
| BB26.11 | | [M]Papillary epidermoid carcinoma | |  |
| BB29.00 | | [M]Squamous cell carcinoma in situ NOS | |  |
| BB29.11 | | [M]Epidermoid carcinoma in situ | |  |
| BB29.12 | | [M]Intraepidermal carcinoma NOS | |  |
| BB29.13 | | [M]Intraepithelial squamous cell carcinoma | |  |
| BB2A.00 | | [M]Squamous cell carcinoma NOS | |  |
| BB2A.11 | | [M]Epidermoid carcinoma NOS | |  |
| BB2A.12 | | [M]Spinous cell carcinoma | |  |
| BB2A.13 | | [M]Squamous cell carcinoma of skin NOS | |  |
| BB2B.00 | | [M]Squamous cell carcinoma, metastatic NOS | |  |
| BB2C.00 | | [M]Squamous cell carcinoma, keratinising type NOS | |  |
| BB2C.11 | | [M]Epidermoid carcinoma, keratinising type | |  |
| BB2D.00 | | [M]Squamous cell carcinoma, large cell, non-keratinising | |  |
| BB2E.00 | | [M]Squamous cell carcinoma, small cell, non-keratinising | |  |
| BB2F.00 | | [M]Squamous cell carcinoma, spindle cell type | |  |
| BB2G.00 | | [M]Adenoid squamous cell carcinoma | |  |
| BB2J.00 | | [M]Squamous cell carcinoma, microinvasive | |  |
| BB2M.00 | | [M]Lymphoepithelial carcinoma | |  |
| BB31.00 | | [M]Basal cell carcinoma NOS | |  |
| BB32.00 | | [M]Multicentric basal cell carcinoma | |  |
| BB33.00 | | [M]Basal cell carcinoma, morphoea type | |  |
| BB34.00 | | [M]Basal cell carcinoma, fibroepithelial type | |  |
| BB35.00 | | [M]Basosquamous carcinoma | |  |
| BB36.00 | | [M]Metatypical carcinoma | |  |
| BB3C.00 | | [M]Superficial basal cell carcinoma | |  |
| BB3D.00 | | [M]Basal cell carcinoma, nodular | |  |
| BB3E.00 | | [M]Basal cell carcinoma, micronodular | |  |
| BB3F.00 | | [M]Basal cell carcinoma, infiltrative | |  |
| BB3G.00 | | [M]Pigmented basal cell carcinoma | |  |
| BB4..00 | | [M]Transitional cell papillomas and carcinomas | |  |
| BB42.00 | | [M]Transitional cell carcinoma in situ | |  |
| BB43.00 | | [M]Transitional cell carcinoma NOS | |  |
| BB43.11 | | [M]Urothelial carcinoma | |  |
| BB46.00 | | [M]Schneiderian carcinoma | |  |
| BB47.00 | | [M]Transitional cell carcinoma, spindle cell type | |  |
| BB48.00 | | [M]Basaloid carcinoma | |  |
| BB49.00 | | [M]Cloacogenic carcinoma | |  |
| BB4A.00 | | [M]Papillary transitional cell carcinoma | |  |
| BB4z.00 | | [M]Transitional cell papilloma or carcinoma NOS | |  |
| BB5..00 | | [M]Adenomas and adenocarcinomas | |  |
| BB5..11 | | [M]Adenocarcinomas | |  |
| BB51.00 | | [M]Adenocarcinoma in situ | |  |
| BB51000 | | [M]Adenocarcinoma in situ in villous adenoma | |  |
| BB51100 | | [M]Adenocarcinoma in situ in tubulovillous adenoma | |  |
| BB52.00 | | [M]Adenocarcinoma NOS | |  |
| BB52000 | | [M]Adenocarcinoma in tubulovillous adenoma | |  |
| BB53.00 | | [M]Adenocarcinoma, metastatic, NOS | |  |
| BB54.00 | | [M]Scirrhous adenocarcinoma | |  |
| BB56.00 | | [M]Superficial spreading adenocarcinoma | |  |
| BB57.00 | | [M]Adenocarcinoma, intestinal type | |  |
| BB58.00 | | [M]Carcinoma, diffuse type | |  |
| BB5a.00 | | [M]Renal adenoma and carcinoma | |  |
| BB5a000 | | [M]Renal cell carcinoma | |  |
| BB5az00 | | [M]Renal adenoma or carcinoma NOS | |  |
| BB5B.00 | | [M]Pancreatic adenomas and carcinomas | |  |
| BB5b.00 | | [M]Granular cell carcinoma | |  |
| BB5B100 | | [M]Islet cell carcinoma | |  |
| BB5B600 | | [M]Mixed islet cell and exocrine adenocarcinoma | |  |
| BB5Bz00 | | [M]Pancreatic adenoma or carcinoma NOS | |  |
| BB5c.00 | | [M]Parathyroid adenomas and adenocarcinomas | |  |
| BB5C.00 | | [M]Gastrinoma and carcinomas | |  |
| BB5Cz00 | | [M]Gastrinoma or carcinoma NOS | |  |
| BB5cz00 | | [M]Parathyroid adenoma or adenocarcinoma NOS | |  |
| BB5D.00 | | [M]Hepatobiliary tract adenomas and carcinomas | |  |
| BB5D.11 | | [M]Biliary tract adenomas and adenocarcinomas | |  |
| BB5D100 | | [M]Cholangiocarcinoma | |  |
| BB5D111 | | [M]Bile duct carcinoma | |  |
| BB5D300 | | [M]Bile duct cystadenocarcinoma | |  |
| BB5D500 | | [M]Hepatocellular carcinoma NOS | |  |
| BB5D513 | | [M]Liver cell carcinoma | |  |
| BB5D700 | | [M]Combined hepatocellular carcinoma and cholangiocarcinoma | |  |
| BB5D711 | | [M]Hepatocholangiocarcinoma | |  |
| BB5D800 | | [M]Hepatocellular carcinoma, fibrolamellar | |  |
| BB5dz00 | | [M]Mixed cell adenoma or adenocarcinoma NOS | |  |
| BB5Dz00 | | [M]Hepatobiliary adenoma or carcinoma NOS | |  |
| BB5f.00 | | [M]Thyroid adenoma and adenocarcinoma | |  |
| BB5F.00 | | [M]Trabecular adenocarcinoma | |  |
| BB5f100 | | [M]Follicular adenocarcinoma NOS | |  |
| BB5f111 | | [M]Follicular carcinoma | |  |
| BB5f200 | | [M]Follicular adenocarcinoma, well differentiated type | |  |
| BB5f300 | | [M]Follicular adenocarcinoma, trabecular type | |  |
| BB5f600 | | [M]Papillary and follicular adenocarcinoma | |  |
| BB5f700 | | [M]Nonencapsulated sclerosing carcinoma | |  |
| BB5fz00 | | [M]Thyroid adenoma or adenocarcinoma NOS | |  |
| BB5h100 | | [M]Adrenal cortical carcinoma | |  |
| BB5j.00 | | [M]Endometrioid adenomas and carcinomas | |  |
| BB5J.00 | | [M]Adenoid cystic carcinoma | |  |
| BB5J.11 | | [M]Cylindroid adenocarcinoma | |  |
| BB5j200 | | [M]Endometrioid carcinoma | |  |
| BB5jz00 | | [M]Endometrioid adenoma or carcinoma NOS | |  |
| BB5K.00 | | [M]Cribriform carcinoma | |  |
| BB5L.00 | | [M]Adenomatous and adenocarcinomatous polyps | |  |
| BB5L100 | | [M]Adenocarcinoma in adenomatous polyp | |  |
| BB5L200 | | [M]Adenocarcinoma in situ in adenomatous polyp | |  |
| BB5L300 | | [M]Adenocarcinoma in multiple adenomatous polyps | |  |
| BB5Lz00 | | [M]Adenomatous or adenocarcinomatous polyp NOS | |  |
| BB5M.00 | | [M]Tubular adenomas and adenocarcinomas | |  |
| BB5M100 | | [M]Tubular adenocarcinoma | |  |
| BB5Mz00 | | [M]Tubular adenoma or adenocarcinoma NOS | |  |
| BB5N.00 | | [M]Adenomatous and adenocarcinomatous polyps of colon | |  |
| BB5N.11 | | [M]Adenoma or or adenocarcinoma in polyposis coli | |  |
| BB5N100 | | [M]Adenocarcinoma in adenomatous polposis coli | |  |
| BB5Nz00 | | [M]Adenomatous or adenocarcinomatous polyps of the colon NOS | |  |
| BB5P.00 | | [M]Solid carcinoma NOS | |  |
| BB5R900 | | [M]Neuroendocrine carcinoma | |  |
| BB5RA00 | | [M]Merkel cell carcinoma | |  |
| BB5S.00 | | [M]Respiratory tract adenomas and adenocarcinomas | |  |
| BB5S200 | | [M]Bronchiolo-alveolar adenocarcinoma | |  |
| BB5S211 | | [M]Alveolar cell carcinoma | |  |
| BB5S212 | | [M]Bronchiolar carcinoma | |  |
| BB5S400 | | [M]Alveolar adenocarcinoma | |  |
| BB5Sz00 | | [M]Respiratory tract adenoma or adenocarcinoma NOS | |  |
| BB5T.00 | | [M]Papillary adenomas and adenocarcinomas | |  |
| BB5T100 | | [M]Papillary adenocarcinoma NOS | |  |
| BB5Tz00 | | [M]Papillary adenoma or adenocarcinoma NOS | |  |
| BB5U.00 | | [M]Villous adenomas and adenocarcinomas | |  |
| BB5U100 | | [M]Adenocarcinoma in villous adenoma | |  |
| BB5U200 | | [M]Villous adenocarcinoma | |  |
| BB5Uz00 | | [M]Villous adenoma or adenocarcinoma NOS | |  |
| BB5V.00 | | [M]Pituitary adenomas and carcinomas | |  |
| BB5V100 | | [M]Chromophobe carcinoma | |  |
| BB5V311 | | [M]Eosinophil carcinoma | |  |
| BB5V700 | | [M]Basophil carcinoma | |  |
| BB5V711 | | [M]Mucoid cell carcinoma | |  |
| BB5Vz00 | | [M]Pituitary adenoma or carcinoma NOS | |  |
| BB5W.00 | | [M]Oxyphilic adenomas and adenocarcinomas | |  |
| BB5W100 | | [M]Oxyphilic adenocarcinoma | |  |
| BB5W111 | | [M]Hurthle cell adenocarcinoma | |  |
| BB5W112 | | [M]Oncytic adenocarcinoma | |  |
| BB5Wz00 | | [M]Oxyphilic adenoma or adenocarcinoma NOS | |  |
| BB5X.00 | | [M]Clear cell adenomas and adenocarcinomas | |  |
| BB5X100 | | [M]Clear cell adenocarcinoma NOS | |  |
| BB5Xz00 | | [M]Clear cell adenoma or adenocarcinoma NOS | |  |
| BB5y000 | | [M]Basal cell adenocarcinoma | |  |
| BB5z.00 | | [M]Adenoma or adenocarcinoma NOS | |  |
| BB60.00 | | [M]Skin appendage adenoma and carcinoma | |  |
| BB60100 | | [M]Skin appendage carcinoma | |  |
| BB61.00 | | [M]Sweat gland adenoma and adenocarcinomas | |  |
| BB61200 | | [M]Sweat gland adenocarcinoma | |  |
| BB62.00 | | [M]Apocrine adenoma and adenocarcinomas | |  |
| BB62100 | | [M]Apocrine adenocarcinoma | |  |
| BB62z00 | | [M]Apocrine adenoma or adenocarcinoma NOS | |  |
| BB69.00 | | [M]Sebaceous adenoma and adenocarcinoma | |  |
| BB69100 | | [M]Sebaceous adenocarcinoma | |  |
| BB69z00 | | [M]Sebaceous adenoma or adenocarcinoma NOS | |  |
| BB6A.00 | | [M]Ceruminous adenoma and adenocarcinoma | |  |
| BB71.00 | | [M]Mucoepidermoid carcinoma | |  |
| BB80.00 | | [M]Cystadenoma and carcinoma | |  |
| BB80100 | | [M]Cystadenocarcinoma NOS | |  |
| BB80z00 | | [M]Cystadenoma or carcinoma NOS | |  |
| BB81.11 | | [M]Ovarian cystadenoma or carcinoma | |  |
| BB81200 | | [M]Serous cystadenocarcinoma, NOS | |  |
| BB81500 | | [M]Papillary cystadenocarcinoma, NOS | |  |
| BB81800 | | [M]Papillary serous cystadenocarcinoma | |  |
| BB81B00 | | [M]Serous surface papillary carcinoma | |  |
| BB81E00 | | [M]Mucinous cystadenocarcinoma NOS | |  |
| BB81E11 | | [M]Pseudomucinous adenocarcinoma | |  |
| BB81H00 | | [M]Papillary mucinous cystadenocarcinoma | |  |
| BB82.00 | | [M]Mucinous adenoma and adenocarcinoma | |  |
| BB82100 | | [M]Mucinous adenocarcinoma | |  |
| BB82111 | | [M]Colloid adenocarcinoma | |  |
| BB82112 | | [M]Gelatinous adenocarcinoma | |  |
| BB82114 | | [M]Mucous adenocarcinoma | |  |
| BB82z00 | | [M]Mucinous adenoma or adenocarcinoma NOS | |  |
| BB84.00 | | [M]Mucin-producing adenocarcinoma | |  |
| BB85.00 | | [M]Signet ring carcinoma | |  |
| BB85000 | | [M]Signet ring cell carcinoma | |  |
| BB85100 | | [M]Metastatic signet ring cell carcinoma | |  |
| BB85z00 | | [M]Signet ring carcinoma NOS | |  |
| BB90.00 | | [M]Intraductal carcinoma, noninfiltrating NOS | |  |
| BB91.00 | | [M]Infiltrating duct carcinoma | |  |
| BB91.11 | | [M]Duct carcinoma NOS | |  |
| BB91000 | | [M]Intraductal papillary adenocarcinoma with invasion | |  |
| BB91100 | | [M]Infiltrating duct and lobular carcinoma | |  |
| BB92.00 | | [M]Comedocarcinoma, noninfiltrating | |  |
| BB93.00 | | [M]Comedocarcinoma NOS | |  |
| BB94.00 | | [M]Juvenile breast carcinoma | |  |
| BB94.11 | | [M]Secretory breast carcinoma | |  |
| BB96.00 | | [M]Noninfiltrating intraductal papillary adenocarcinoma | |  |
| BB9B.00 | | [M]Medullary carcinoma NOS | |  |
| BB9B.11 | | [M]C cell carcinoma | |  |
| BB9C.00 | | [M]Medullary carcinoma with amyloid stroma | |  |
| BB9C.11 | | [M]Solid carcinoma with amyloid stroma | |  |
| BB9D.00 | | [M]Medullary carcinoma with lymphoid stroma | |  |
| BB9E.00 | | [M]Lobular carcinoma in situ | |  |
| BB9E000 | | [M]Intraductal carcinoma and lobular carcinoma in situ | |  |
| BB9F.00 | | [M]Lobular carcinoma NOS | |  |
| BB9G.00 | | [M]Infiltrating ductular carcinoma | |  |
| BB9H.00 | | [M]Inflammatory carcinoma | |  |
| BB9K.00 | | [M]Paget's disease and infiltrating breast duct carcinoma | |  |
| BB9K000 | | [M]Paget's disease and intraductal carcinoma of breast | |  |
| BB9M.00 | | [M]Intracystic carcinoma NOS | |  |
| BBA2.00 | | [M]Acinar cell carcinoma | |  |
| BBB0.00 | | [M]Adenosquamous carcinoma | |  |
| BBB2.00 | | [M]Adenocarcinoma with squamous metaplasia | |  |
| BBB3.00 | | [M]Adenocarcinoma with cartilaginous and osseous metaplasia | |  |
| BBB4.00 | | [M]Adenocarcinoma with spindle cell metaplasia | |  |
| BBB5.00 | | [M]Adenocarcinoma with apocrine metaplasia | |  |
| BBB7.00 | | [M]Epithelial-myoepithelial carcinoma | |  |
| BBCA.00 | | [M]Sertoli cell carcinoma | |  |
| BBE1.11 | | [M]Melanocarcinoma | |  |
| BBE1.14 | | [M]Naevocarcinoma | |  |
| BBLG.00 | | [M]Carcinoma in pleomorphic adenoma | |  |
| BBQ3.00 | | [M]Embryonal carcinoma NOS | |  |
| BBQ4.11 | | [M]Infantile embryonal carcinoma | |  |
| BBQ7300 | | [M]Teratocarcinoma | |  |
| BBR2.00 | | [M]Choriocarcinoma | |  |
| BBR3.00 | | [M]Choriocarcinoma combined with teratoma | |  |
| BBZ2.11 | | [M]Intraosseous carcinoma | |  |
| D212000 | | Anaemia in ovarian carcinoma | |  |
| **SECONDARY CARE (HOSPITAL EPISODES)** | | | | |
| **ICD-10 code** | **ICD-10 description** | | | |
| C00 | Malignant neoplasm of lip | | | |
| C01 | Malignant neoplasm of base of tongue | | | |
| C02 | Malignant neoplasm of other and unspecified parts of tongue | | | |
| C03 | Malignant neoplasm of gum | | | |
| C04 | Malignant neoplasm of floor of mouth | | | |
| C05 | Malignant neoplasm of palate | | | |
| C06 | Malignant neoplasm of other and unspecified parts of mouth | | | |
| C07 | Malignant neoplasm of parotid gland | | | |
| C08 | Malignant neoplasm of other and unspecified major salivary glands | | | |
| C09 | Malignant neoplasm of tonsil | | | |
| C10 | Malignant neoplasm of oropharynx | | | |
| C11 | Malignant neoplasm of nasopharynx | | | |
| C12 | Malignant neoplasm of piriform sinus | | | |
| C13 | Malignant neoplasm of hypopharynx | | | |
| C14 | Malignant neoplasm of other and ill-defined sites in the lip, oral cavity and pharynx | | | |
| C15 | Malignant neoplasm of oesophagus | | | |
| C16 | Malignant neoplasm of stomach | | | |
| C17 | Malignant neoplasm of small intestine | | | |
| C18 | Malignant neoplasm of colon | | | |
| C19 | Malignant neoplasm of rectosigmoid junction | | | |
| C20 | Malignant neoplasm of rectum | | | |
| C21 | Malignant neoplasm of anus and anal canal | | | |
| C22 | Malignant neoplasm of liver and intrahepatic bile ducts | | | |
| C23 | Malignant neoplasm of gallbladder | | | |
| C24 | Malignant neoplasm of other and unspecified parts of biliary tract | | | |
| C25 | Malignant neoplasm of pancreas | | | |
| C26 | Malignant neoplasm of other and ill-defined digestive organs | | | |
| C30 | Malignant neoplasm of nasal cavity and middle ear | | | |
| C31 | Malignant neoplasm of accessory sinuses | | | |
| C32 | Malignant neoplasm of larynx | | | |
| C33 | Malignant neoplasm of trachea | | | |
| C34 | Malignant neoplasm of bronchus and lung | | | |
| C37 | Malignant neoplasm of thymus | | | |
| C38 | Malignant neoplasm of heart, mediastinum and pleura | | | |
| C39 | Malignant neoplasm of other and ill-defined sites in the respiratory system and intrathoracic organs | | | |
| C40 | Malignant neoplasm of bone and articular cartilage of limbs | | | |
| C41 | Malignant neoplasm of bone and articular cartilage of other and unspecified sites | | | |
| C43 | Malignant melanoma of skin | | | |
| C44 | Other malignant neoplasms of skin | | | |
| C45 | Mesothelioma | | | |
| C46 | Kaposi sarcoma | | | |
| C47 | Malignant neoplasm of peripheral nerves and autonomic nervous system | | | |
| C48 | Malignant neoplasm of retroperitoneum and peritoneum | | | |
| C49 | Malignant neoplasm of other connective and soft tissue | | | |
| C50 | Malignant neoplasm of breast | | | |
| C51 | Malignant neoplasm of vulva | | | |
| C52 | Malignant neoplasm of vagina | | | |
| C53 | Malignant neoplasm of cervix uteri | | | |
| C54 | Malignant neoplasm of corpus uteri | | | |
| C55 | Malignant neoplasm of uterus, part unspecified | | | |
| C56 | Malignant neoplasm of ovary | | | |
| C57 | Malignant neoplasm of other and unspecified female genital organs | | | |
| C58 | Malignant neoplasm of placenta | | | |
| C60 | Malignant neoplasm of penis | | | |
| C61 | Malignant neoplasm of prostate | | | |
| C62 | Malignant neoplasm of testis | | | |
| C63 | Malignant neoplasm of other and unspecified male genital organs | | | |
| C64 | Malignant neoplasm of kidney, except renal pelvis | | | |
| C65 | Malignant neoplasm of renal pelvis | | | |
| C66 | Malignant neoplasm of ureter | | | |
| C67 | Malignant neoplasm of bladder | | | |
| C68 | Malignant neoplasm of other and unspecified urinary organs | | | |
| C69 | Malignant neoplasm of eye and adnexa | | | |
| C70 | Malignant neoplasm of meninges | | | |
| C71 | Malignant neoplasm of brain | | | |
| C72 | Malignant neoplasm of spinal cord, cranial nerves and other parts of central nervous system | | | |
| C73 | Malignant neoplasm of thyroid gland | | | |
| C74 | Malignant neoplasm of adrenal gland | | | |
| C75 | Malignant neoplasm of other endocrine glands and related structures | | | |
| C76 | Malignant neoplasm of other and ill-defined sites | | | |
| C77 | Secondary and unspecified malignant neoplasm of lymph nodes | | | |
| C78 | Secondary malignant neoplasm of respiratory and digestive organs | | | |
| C79 | Secondary malignant neoplasm of other and unspecified sites | | | |
| C80 | Malignant neoplasm, without specification of site | | | |
| C81 | Hodgkin lymphoma | | | |
| C82 | Follicular lymphoma | | | |
| C83 | Non-follicular lymphoma | | | |
| C84 | Mature T/NK-cell lymphomas | | | |
| C85 | Other and unspecified types of non-Hodgkin lymphoma | | | |
| C86 | Other specified types of T/NK-cell lymphoma | | | |
| C88 | Malignant immunoproliferative diseases | | | |
| C90 | Multiple myeloma and malignant plasma cell neoplasms | | | |
| C91 | Lymphoid leukaemia | | | |
| C92 | Myeloid leukaemia | | | |
| C93 | Monocytic leukaemia | | | |
| C94 | Other leukaemias of specified cell type | | | |
| C95 | Leukaemia of unspecified cell type | | | |
| C96 | Other and unspecified malignant neoplasms of lymphoid, haematopoietic and related tissue | | | |
| C97 | Malignant neoplasms of independent (primary) multiple sites | | | |
| **Glucose Lowering Therapies** | | | | |
| **Product Code** | **Product name** | | **Drug substance name** | |
| 5174 | Acarbose 100mg tablets | | Acarbose | |
| 479 | Acarbose 50mg tablets | | Acarbose | |
| 71020 | Acarbose 50mg tablets (Actavis UK Ltd) | | Acarbose | |
| 9105 | Glucobay 100mg tablets (Bayer Plc) | | Acarbose | |
| 5621 | Glucobay 50mg tablets (Bayer Plc) | | Acarbose | |
| 60328 | Alogliptin 12.5mg tablets | | Alogliptin benzoate | |
| 59177 | Alogliptin 25mg tablets | | Alogliptin benzoate | |
| 68258 | Alogliptin 25mg tablets (Colorama Pharmaceuticals Ltd) | | Alogliptin benzoate | |
| 69459 | Alogliptin 25mg tablets (Ennogen Healthcare Ltd) | | Alogliptin benzoate | |
| 59809 | Alogliptin 6.25mg tablets | | Alogliptin benzoate | |
| 60681 | Vipidia 12.5mg tablets (Takeda UK Ltd) | | Alogliptin benzoate | |
| 60682 | Vipidia 25mg tablets (Takeda UK Ltd) | | Alogliptin benzoate | |
| 62326 | Vipidia 6.25mg tablets (Takeda UK Ltd) | | Alogliptin benzoate | |
| 46665 | Linagliptin 5mg tablets | | Linagliptin | |
| 46716 | Trajenta 5mg tablets (Boehringer Ingelheim Ltd) | | Linagliptin | |
| 45821 | Onglyza 2.5mg tablets (AstraZeneca UK Ltd) | | Saxagliptin hydrochloride | |
| 41431 | Onglyza 5mg tablets (AstraZeneca UK Ltd) | | Saxagliptin hydrochloride | |
| 45775 | Saxagliptin 2.5mg tablets | | Saxagliptin hydrochloride | |
| 41204 | Saxagliptin 5mg tablets | | Saxagliptin hydrochloride | |
| 35462 | Januvia 100mg tablets (Merck Sharp & Dohme Ltd) | | Sitagliptin phosphate | |
| 50124 | Januvia 25mg tablets (Merck Sharp & Dohme Ltd) | | Sitagliptin phosphate | |
| 50087 | Januvia 50mg tablets (Merck Sharp & Dohme Ltd) | | Sitagliptin phosphate | |
| 35022 | Sitagliptin 100mg tablets | | Sitagliptin phosphate | |
| 48533 | Sitagliptin 25mg tablets | | Sitagliptin phosphate | |
| 48401 | Sitagliptin 50mg tablets | | Sitagliptin phosphate | |
| 69654 | Qtern 5mg/10mg tablets (AstraZeneca UK Ltd) | | Saxagliptin hydrochloride/Dapagliflozin propanediol monohydrate | |
| 69540 | Saxagliptin 5mg / Dapagliflozin 10mg tablets | | Saxagliptin hydrochloride/Dapagliflozin propanediol monohydrate | |
| 5678 | Nateglinide 120mg tablets | | Nateglinide | |
| 5989 | Nateglinide 180mg tablets | | Nateglinide | |
| 11483 | Nateglinide 60mg tablets | | Nateglinide | |
| 15955 | Starlix 120mg tablets (Novartis Pharmaceuticals UK Ltd) | | Nateglinide | |
| 27125 | Starlix 180mg tablets (Novartis Pharmaceuticals UK Ltd) | | Nateglinide | |
| 23945 | Starlix 60mg tablets (Novartis Pharmaceuticals UK Ltd) | | Nateglinide | |
| 52203 | Enyglid 0.5mg tablets (Consilient Health Ltd) | | Repaglinide | |
| 11321 | NovoNorm 1mg tablets (Novo Nordisk Ltd) | | Repaglinide | |
| 11366 | NovoNorm 2mg tablets (Novo Nordisk Ltd) | | Repaglinide | |
| 11316 | NovoNorm 500microgram tablets (Novo Nordisk Ltd) | | Repaglinide | |
| 61925 | NovoNorm 500microgram tablets (Waymade Healthcare Plc) | | Repaglinide | |
| 36948 | Prandin 0.5mg tablets (Novo Nordisk Ltd) | | Repaglinide | |
| 36774 | Prandin 1mg tablets (Novo Nordisk Ltd) | | Repaglinide | |
| 35561 | Prandin 2mg tablets (Novo Nordisk Ltd) | | Repaglinide | |
| 9707 | Repaglinide 1mg tablets | | Repaglinide | |
| 9748 | Repaglinide 2mg tablets | | Repaglinide | |
| 9865 | Repaglinide 500microgram tablets | | Repaglinide | |
| 69947 | Albiglutide 30mg powder and solvent for solution for injection pre-filled disposable devices | | Albiglutide | |
| 63785 | Dulaglutide 0.75mg/0.5ml solution for injection pre-filled disposable devices | | Dulaglutide | |
| 63823 | Dulaglutide 1.5mg/0.5ml solution for injection pre-filled disposable devices | | Dulaglutide | |
| 63401 | Trulicity 0.75mg/0.5ml solution for injection pre-filled pen (Eli Lilly and Company Ltd) | | Dulaglutide | |
| 63336 | Trulicity 1.5mg/0.5ml solution for injection pre-filled pen (Eli Lilly and Company Ltd) | | Dulaglutide | |
| 62661 | Bydureon 2mg powder and solvent for prolonged-release suspension for injection pre-filled pen (AstraZeneca UK Ltd) | | Exenatide | |
| 46469 | Bydureon 2mg powder and solvent for prolonged-release suspension for injection vials (AstraZeneca UK Ltd) | | Exenatide | |
| 64622 | Bydureon 2mg powder and solvent for prolonged-release suspension for injection vials (Lexon (UK) Ltd) | | Exenatide | |
| 35150 | Byetta 10micrograms/0.04ml solution for injection 2.4ml pre-filled disposable devices (AstraZeneca UK Ltd) | | Exenatide | |
| 35144 | Byetta 5micrograms/0.02ml solution for injection 1.2ml pre-filled disposable devices (AstraZeneca UK Ltd) | | Exenatide | |
| 35149 | Exenatide 10micrograms/0.04ml solution for injection 2.4ml pre-filled disposable devices | | Exenatide | |
| 62904 | Exenatide 2mg powder and solvent for prolonged-release suspension for injection pre-filled disposable devices | | Exenatide | |
| 46458 | Exenatide 2mg powder and solvent for prolonged-release suspension for injection vials | | Exenatide | |
| 35251 | Exenatide 5micrograms/0.02ml solution for injection 1.2ml pre-filled disposable devices | | Exenatide | |
| 69548 | Saxenda 6mg/ml solution for injection 3ml pre-filled pen (Novo Nordisk Ltd) | | Liraglutide | |
| 40693 | Liraglutide 6mg/ml solution for injection 3ml pre-filled disposable devices | | Liraglutide | |
| 40642 | Victoza 6mg/ml solution for injection 3ml pre-filled pen (Novo Nordisk Ltd) | | Liraglutide | |
| 55459 | Lixisenatide 10micrograms/0.2ml solution for injection 3ml pre-filled disposable devices | | Lixisenatide | |
| 55413 | Lixisenatide 20micrograms/0.2ml solution for injection 3ml pre-filled disposable devices | | Lixisenatide | |
| 55728 | Lyxumia 10micrograms/0.2ml solution for injection 3ml pre-filled pen (Sanofi) | | Lixisenatide | |
| 55729 | Lyxumia 20micrograms/0.2ml solution for injection 3ml pre-filled pen (Sanofi) | | Lixisenatide | |
| 63562 | Insulin degludec 100units/ml / Liraglutide 3.6mg/ml solution for injection 3ml pre-filled disposable devices | | Insulin degludec/Liraglutide | |
| 62899 | Xultophy 100units/ml / 3.6mg/ml solution for injection 3ml pre-filled pen (Novo Nordisk Ltd) | | Insulin degludec/Liraglutide | |
| 13516 | Hypurin bovine isophane 100unit/ml Injection (C P Pharmaceuticals Ltd) | | Insulin isophane bovine | |
| 71137 | Hypurin Bovine Isophane 100units/ml suspension for injection 10ml vials (Waymade Healthcare Plc) | | Insulin isophane bovine | |
| 14340 | Hypurin Bovine Isophane 100units/ml suspension for injection 10ml vials (Wockhardt UK Ltd) | | Insulin isophane bovine | |
| 28588 | Hypurin Bovine Isophane 100units/ml suspension for injection 3ml cartridges (Wockhardt UK Ltd) | | Insulin isophane bovine | |
| 18590 | Insulin isophane bovine 100units/ml suspension for injection 10ml vials | | Insulin isophane bovine | |
| 36066 | Insulin isophane bovine 100units/ml suspension for injection 3ml cartridges | | Insulin isophane bovine | |
| 38422 | Isophane 100iu/ml Injection (Celltech Pharma Europe Ltd) | | Insulin isophane bovine | |
| 30236 | Isophane insulin 100iu/ml Injection | | Insulin Isophane Bovine | |
| 47856 | Neuphane 100unit/ml Injection (Wellcome Medical Division) | | Insulin isophane bovine | |
| 15484 | Insulin isophane bovine 100units/ml suspension for injection 1.5ml cartridges | | Insulin isophane bovine | |
| 8118 | Humaject i 100iu/ml Pen (Eli Lilly and Company Ltd) | | Insulin Isophane Human | |
| 7772 | Human protaphane 100unit/ml Injection (Novo Nordisk Ltd) | | Insulin isophane human | |
| 7771 | Human protaphane penfill 100 100unit/ml Penfill (Novo Nordisk Ltd) | | Insulin isophane human | |
| 4760 | Humulin i 100unit/ml Injection (Eli Lilly and Company Ltd) | | Insulin Isophane Human | |
| 14918 | Humulin I 100units/ml suspension for injection 10ml vials (Eli Lilly and Company Ltd) | | Insulin isophane human | |
| 14357 | Humulin I 100units/ml suspension for injection 3ml cartridges (Eli Lilly and Company Ltd) | | Insulin isophane human | |
| 71361 | Humulin I 100units/ml suspension for injection 3ml cartridges (Waymade Healthcare Plc) | | Insulin isophane human | |
| 43950 | Humulin I KwikPen 100units/ml suspension for injection 3ml pre-filled pen (Eli Lilly and Company Ltd) | | Insulin isophane human | |
| 10229 | Humulin I Pen 100units/ml suspension for injection 3ml pre-filled pen (Eli Lilly and Company Ltd) | | Insulin isophane human | |
| 1886 | Insulatard 100iu/ml GE injection (Novo Nordisk Ltd) | | Insulin isophane human | |
| 33966 | Insulatard 100unit/ml Injection (Novo Nordisk Ltd) | | Insulin Isophane Human | |
| 14928 | Insulatard 100units/ml suspension for injection 10ml vials (Novo Nordisk Ltd) | | Insulin isophane human | |
| 71118 | Insulatard 100units/ml suspension for injection 10ml vials (Waymade Healthcare Plc) | | Insulin isophane human | |
| 5891 | Insulatard FlexPen 100units/ml suspension for injection (Novo Nordisk Ltd) | | Insulin isophane human | |
| 9737 | Insulatard innolet 100iu/ml Injection (Novo Nordisk Ltd) | | Insulin isophane human | |
| 10208 | Insulatard InnoLet 100units/ml suspension for injection 3ml pre-filled pen (Novo Nordisk Ltd) | | Insulin isophane human | |
| 71428 | Insulatard InnoLet 100units/ml suspension for injection 3ml pre-filled pen (Waymade Healthcare Plc) | | Insulin isophane human | |
| 1595 | Insulatard NovoLet 100units/ml suspension for injection (Novo Nordisk Ltd) | | Insulin isophane human | |
| 1593 | Insulatard penfill 100 100iu/ml Penfill (Novo Nordisk Ltd) | | Insulin isophane human | |
| 14290 | Insulatard Penfill 100units/ml suspension for injection 3ml cartridges (Novo Nordisk Ltd) | | Insulin isophane human | |
| 52748 | Insulatard Penfill 100units/ml suspension for injection 3ml cartridges (Waymade Healthcare Plc) | | Insulin isophane human | |
| 55517 | Insulin isophane human 100units/ml suspension for injection 10ml vials | | Insulin isophane human | |
| 10207 | Insulin isophane human 100units/ml suspension for injection 3ml cartridges | | Insulin isophane human | |
| 25812 | Insulin isophane human 100units/ml suspension for injection 3ml pre-filled disposable devices | | Insulin isophane human | |
| 59500 | Insulin isophane human 100units/ml suspension for injection 5ml vials | | Insulin isophane human | |
| 15961 | Insulin isophane human crb 100iu/ml Injection | | Insulin Isophane Human | |
| 13729 | Insulin isophane human emp 100unit/ml Injection | | Insulin Isophane Human | |
| 11080 | Insulin isophane human prb 100iu/ml Injection | | Insulin Isophane Human | |
| 14925 | Insulin isophane human vial 100unit/ml Sterile suspension injection | | Insulin Isophane Human | |
| 5501 | Insuman basal 100iu/ml Injection (Aventis Pharma) | | Insulin Isophane Human | |
| 27461 | Insuman Basal 100units/ml suspension for injection 3ml cartridges (Sanofi) | | Insulin isophane human | |
| 23992 | Insuman Basal 100units/ml suspension for injection 3ml pre-filled OptiSet pen (Sanofi) | | Insulin isophane human | |
| 46001 | Insuman Basal 100units/ml suspension for injection 3ml pre-filled SoloStar pen (Sanofi) | | Insulin isophane human | |
| 35468 | Insuman Basal 100units/ml suspension for injection 5ml vials (Sanofi) | | Insulin isophane human | |
| 10175 | Insulin isophane human 100units/ml suspension for injection 1.5ml cartridges | | Insulin isophane human | |
| 28183 | Hypurin Porcine Isophane 100units/ml suspension for injection 10ml vials (Wockhardt UK Ltd) | | Insulin isophane porcine | |
| 14933 | Hypurin Porcine Isophane 100units/ml suspension for injection 3ml cartridges (Wockhardt UK Ltd) | | Insulin isophane porcine | |
| 7350 | Insulin isophane porcine 100units/ml suspension for injection 10ml vials | | Insulin isophane porcine | |
| 30686 | Insulin isophane porcine 100units/ml suspension for injection 3ml cartridges | | Insulin isophane porcine | |
| 13819 | Hypurin Porcine Isophane 100units/ml suspension for injection 1.5ml cartridges (C P Pharmaceuticals Ltd) | | Insulin isophane porcine | |
| 4247 | Insulin isophane porcine 100units/ml suspension for injection 1.5ml cartridges | | Insulin isophane porcine | |
| 1843 | Pork Insulatard 100units/ml suspension for injection 10ml vials (Novo Nordisk Ltd) | | Insulin isophane porcine | |
| 67279 | Pork Insulatard 100units/ml suspension for injection 10ml vials (Waymade Healthcare Plc) | | Insulin isophane porcine | |
| 9503 | Hypurin Bovine Protamine Zinc 100units/ml suspension for injection 10ml vials (Wockhardt UK Ltd) | | Insulin protamine zinc bovine | |
| 14505 | Insulin protamine zinc bovine 100units/ml suspension for injection 10ml vials | | Insulin protamine zinc bovine | |
| 55907 | Insulin degludec 100units/ml solution for injection 3ml cartridges | | Insulin degludec | |
| 55687 | Insulin degludec 100units/ml solution for injection 3ml pre-filled disposable devices | | Insulin degludec | |
| 56691 | Insulin degludec 200units/ml solution for injection 3ml pre-filled disposable devices | | Insulin degludec | |
| 55462 | Tresiba FlexTouch 100units/ml solution for injection 3ml pre-filled pen (Novo Nordisk Ltd) | | Insulin degludec | |
| 55234 | Tresiba FlexTouch 200units/ml solution for injection 3ml pre-filled pen (Novo Nordisk Ltd) | | Insulin degludec | |
| 55910 | Tresiba Penfill 100units/ml solution for injection 3ml cartridges (Novo Nordisk Ltd) | | Insulin degludec | |
| 10184 | Insulin detemir 100 iu/ml Solution for injection | | Insulin Detemir | |
| 14301 | Insulin detemir 100units/ml solution for injection 3ml cartridges | | Insulin detemir | |
| 14330 | Insulin detemir 100units/ml solution for injection 3ml pre-filled disposable devices | | Insulin detemir | |
| 6958 | Levemir FlexPen 100units/ml solution for injection 3ml pre-filled pen (Novo Nordisk Ltd) | | Insulin detemir | |
| 55618 | Levemir FlexPen 100units/ml solution for injection 3ml pre-filled pen (Waymade Healthcare Plc) | | Insulin detemir | |
| 35260 | Levemir InnoLet 100units/ml solution for injection 3ml pre-filled pen (Novo Nordisk Ltd) | | Insulin detemir | |
| 6965 | Levemir Penfill 100units/ml solution for injection 3ml cartridges (Novo Nordisk Ltd) | | Insulin detemir | |
| 64987 | Abasaglar 100units/ml solution for injection 3ml cartridges (Eli Lilly and Company Ltd) | | Insulin glargine | |
| 64723 | Abasaglar KwikPen 100units/ml solution for injection 3ml pre-filled pen (Eli Lilly and Company Ltd) | | Insulin glargine | |
| 5953 | Insulin glargine 100iu/ml Injection | | Insulin Glargine | |
| 10259 | Insulin glargine 100units/ml solution for injection 10ml vials | | Insulin glargine | |
| 7393 | Insulin glargine 100units/ml solution for injection 3ml cartridges | | Insulin glargine | |
| 7400 | Insulin glargine 100units/ml solution for injection 3ml pre-filled disposable devices | | Insulin glargine | |
| 64460 | Insulin glargine 300units/ml solution for injection 1.5ml pre-filled disposable devices | | Insulin glargine | |
| 6057 | Lantus 100iu/ml Injection (Aventis Pharma) | | Insulin Glargine | |
| 66316 | Lantus 100units/ml solution for injection 10ml vials (Mawdsley-Brooks & Company Ltd) | | Insulin glargine | |
| 7402 | Lantus 100units/ml solution for injection 10ml vials (Sanofi) | | Insulin glargine | |
| 50633 | Lantus 100units/ml solution for injection 3ml cartridges (Necessity Supplies Ltd) | | Insulin glargine | |
| 7266 | Lantus 100units/ml solution for injection 3ml cartridges (Sanofi) | | Insulin glargine | |
| 71351 | Lantus 100units/ml solution for injection 3ml cartridges (Waymade Healthcare Plc) | | Insulin glargine | |
| 10225 | Lantus 100units/ml solution for injection 3ml OptiClik cartridges (Sanofi) | | Insulin glargine | |
| 7237 | Lantus 100units/ml solution for injection 3ml pre-filled OptiSet pen (Sanofi) | | Insulin glargine | |
| 49831 | Lantus 100units/ml solution for injection 3ml pre-filled SoloStar pen (Necessity Supplies Ltd) | | Insulin glargine | |
| 36853 | Lantus 100units/ml solution for injection 3ml pre-filled SoloStar pen (Sanofi) | | Insulin glargine | |
| 67230 | Lantus 100units/ml solution for injection 3ml pre-filled SoloStar pen (Waymade Healthcare Plc) | | Insulin glargine | |
| 64354 | Toujeo 300units/ml solution for injection 1.5ml pre-filled SoloStar pen (Sanofi) | | Insulin glargine | |
| 56495 | Lantus 100units/ml solution for injection 3ml pre-filled OptiSet pen (Waymade Healthcare Plc) | | Insulin glargine | |
| 57147 | Bolamyn SR 1000mg tablets (Teva UK Ltd) | | Metformin hydrochloride | |
| 39560 | Bolamyn SR 500mg tablets (Teva UK Ltd) | | Metformin hydrochloride | |
| 52221 | Diagemet XL 500mg tablets (Thornton & Ross Ltd) | | Metformin hydrochloride | |
| 55270 | Duformin 500mg Tablet (Dumex Ltd) | | Metformin hydrochloride | |
| 25678 | Glucamet 500mg Tablet (Opus Pharmaceuticals Ltd) | | Metformin hydrochloride | |
| 26258 | Glucamet 850mg Tablet (Opus Pharmaceuticals Ltd) | | Metformin hydrochloride | |
| 64939 | Glucient SR 1000mg tablets (Consilient Health Ltd) | | Metformin hydrochloride | |
| 47939 | Glucient SR 500mg tablets (Consilient Health Ltd) | | Metformin hydrochloride | |
| 70477 | Glucient SR 750mg tablets (Consilient Health Ltd) | | Metformin hydrochloride | |
| 40007 | Glucophage 1000mg oral powder sachets (Merck Serono Ltd) | | Metformin hydrochloride | |
| 40110 | Glucophage 500mg oral powder sachets (Merck Serono Ltd) | | Metformin hydrochloride | |
| 7166 | Glucophage 500mg tablets (Merck Serono Ltd) | | Metformin hydrochloride | |
| 7610 | Glucophage 850mg tablets (Merck Serono Ltd) | | Metformin hydrochloride | |
| 39729 | Glucophage SR 1000mg tablets (Merck Serono Ltd) | | Metformin hydrochloride | |
| 66136 | Glucophage SR 1000mg tablets (Waymade Healthcare Plc) | | Metformin hydrochloride | |
| 52634 | Glucophage SR 500mg tablets (DE Pharmaceuticals) | | Metformin hydrochloride | |
| 50570 | Glucophage SR 500mg tablets (Lexon (UK) Ltd) | | Metformin hydrochloride | |
| 49502 | Glucophage SR 500mg tablets (Mawdsley-Brooks & Company Ltd) | | Metformin hydrochloride | |
| 16044 | Glucophage SR 500mg tablets (Merck Serono Ltd) | | Metformin hydrochloride | |
| 59620 | Glucophage SR 500mg tablets (Waymade Healthcare Plc) | | Metformin hydrochloride | |
| 38400 | Glucophage SR 750mg tablets (Merck Serono Ltd) | | Metformin hydrochloride | |
| 51080 | Metabet SR 1000mg tablets (Actavis UK Ltd) | | Metformin hydrochloride | |
| 46989 | Metabet SR 1000mg tablets (Morningside Healthcare Ltd) | | Metformin hydrochloride | |
| 53774 | Metabet SR 500mg tablets (Actavis UK Ltd) | | Metformin hydrochloride | |
| 45581 | Metabet SR 500mg tablets (Morningside Healthcare Ltd) | | Metformin hydrochloride | |
| 54442 | Metformin (roi) 1000mg Tablet | | Metformin Hydrochloride | |
| 735 | Metformin 100mg/ml Oral solution | | Metformin Hydrochloride | |
| 39598 | Metformin 1g modified-release tablets | | Metformin hydrochloride | |
| 49738 | Metformin 1g modified-release tablets (A A H Pharmaceuticals Ltd) | | Metformin hydrochloride | |
| 62824 | Metformin 1g modified-release tablets (Actavis UK Ltd) | | Metformin hydrochloride | |
| 72001 | Metformin 1g modified-release tablets (DE Pharmaceuticals) | | Metformin hydrochloride | |
| 65923 | Metformin 1g modified-release tablets (Mawdsley-Brooks & Company Ltd) | | Metformin hydrochloride | |
| 60074 | Metformin 1g modified-release tablets (Waymade Healthcare Plc) | | Metformin hydrochloride | |
| 40233 | Metformin 1g oral powder sachets sugar free | | Metformin hydrochloride | |
| 69221 | Metformin 1g oral powder sachets sugar free (J M McGill Ltd) | | Metformin hydrochloride | |
| 63307 | Metformin 1g/5ml oral solution | | Metformin hydrochloride | |
| 68589 | Metformin 1g/5ml oral solution sugar free | | Metformin hydrochloride | |
| 7048 | Metformin 500mg modified-release tablets | | Metformin hydrochloride | |
| 51135 | Metformin 500mg modified-release tablets (A A H Pharmaceuticals Ltd) | | Metformin hydrochloride | |
| 60968 | Metformin 500mg modified-release tablets (Actavis UK Ltd) | | Metformin hydrochloride | |
| 68203 | Metformin 500mg modified-release tablets (Almus Pharmaceuticals Ltd) | | Metformin hydrochloride | |
| 62144 | Metformin 500mg modified-release tablets (DE Pharmaceuticals) | | Metformin hydrochloride | |
| 53478 | Metformin 500mg modified-release tablets (Kent Pharmaceuticals Ltd) | | Metformin hydrochloride | |
| 62265 | Metformin 500mg modified-release tablets (Mawdsley-Brooks & Company Ltd) | | Metformin hydrochloride | |
| 65694 | Metformin 500mg modified-release tablets (Waymade Healthcare Plc) | | Metformin hydrochloride | |
| 39988 | Metformin 500mg oral powder sachets sugar free | | Metformin hydrochloride | |
| 72052 | Metformin 500mg Tablet (Lagap) | | Metformin hydrochloride | |
| 34135 | Metformin 500mg Tablet (M & A Pharmachem Ltd) | | Metformin hydrochloride | |
| 23 | Metformin 500mg tablets | | Metformin hydrochloride | |
| 34323 | Metformin 500mg tablets (A A H Pharmaceuticals Ltd) | | Metformin hydrochloride | |
| 33087 | Metformin 500mg tablets (Actavis UK Ltd) | | Metformin hydrochloride | |
| 55711 | Metformin 500mg tablets (Alliance Healthcare (Distribution) Ltd) | | Metformin hydrochloride | |
| 48149 | Metformin 500mg tablets (Almus Pharmaceuticals Ltd) | | Metformin hydrochloride | |
| 57457 | Metformin 500mg tablets (Aurobindo Pharma Ltd) | | Metformin hydrochloride | |
| 51527 | Metformin 500mg tablets (Boston Healthcare Ltd) | | Metformin hydrochloride | |
| 50970 | Metformin 500mg tablets (Bristol Laboratories Ltd) | | Metformin hydrochloride | |
| 72046 | Metformin 500mg tablets (DE Pharmaceuticals) | | Metformin hydrochloride | |
| 34004 | Metformin 500mg tablets (IVAX Pharmaceuticals UK Ltd) | | Metformin hydrochloride | |
| 34598 | Metformin 500mg tablets (Mylan) | | Metformin hydrochloride | |
| 52442 | Metformin 500mg tablets (Pfizer Ltd) | | Metformin hydrochloride | |
| 71012 | Metformin 500mg tablets (Phoenix Healthcare Distribution Ltd) | | Metformin hydrochloride | |
| 34917 | Metformin 500mg tablets (Teva UK Ltd) | | Metformin hydrochloride | |
| 55739 | Metformin 500mg tablets (Tillomed Laboratories Ltd) | | Metformin hydrochloride | |
| 34504 | Metformin 500mg tablets (Wockhardt UK Ltd) | | Metformin hydrochloride | |
| 71198 | Metformin 500mg tablets (Zanza Laboratories Ltd) | | Metformin hydrochloride | |
| 53867 | Metformin 500mg tablets (Zentiva) | | Metformin hydrochloride | |
| 58051 | Metformin 500mg/5ml oral solution | | Metformin hydrochloride | |
| 44250 | Metformin 500mg/5ml Oral solution (Hillcross Pharmaceuticals Ltd) | | Metformin hydrochloride | |
| 11990 | Metformin 500mg/5ml oral solution sugar free | | Metformin hydrochloride | |
| 68214 | Metformin 500mg/5ml oral solution sugar free (A A H Pharmaceuticals Ltd) | | Metformin hydrochloride | |
| 71890 | Metformin 500mg/5ml oral solution sugar free (Almus Pharmaceuticals Ltd) | | Metformin hydrochloride | |
| 70744 | Metformin 500mg/5ml oral solution sugar free (Focus Pharmaceuticals Ltd) | | Metformin hydrochloride | |
| 68389 | Metformin 500mg/5ml oral solution sugar free (Pinewood Healthcare) | | Metformin hydrochloride | |
| 43270 | Metformin 500mg/5ml oral solution sugar free (Rosemont Pharmaceuticals Ltd) | | Metformin hydrochloride | |
| 58607 | Metformin 500mg/5ml oral solution sugar free (Zentiva) | | Metformin hydrochloride | |
| 60286 | Metformin 500mg/5ml oral suspension | | Metformin hydrochloride | |
| 38355 | Metformin 750mg modified-release tablets | | Metformin hydrochloride | |
| 93 | Metformin 850mg tablets | | Metformin hydrochloride | |
| 33674 | Metformin 850mg tablets (A A H Pharmaceuticals Ltd) | | Metformin hydrochloride | |
| 34836 | Metformin 850mg tablets (Actavis UK Ltd) | | Metformin hydrochloride | |
| 54898 | Metformin 850mg tablets (Almus Pharmaceuticals Ltd) | | Metformin hydrochloride | |
| 34020 | Metformin 850mg tablets (IVAX Pharmaceuticals UK Ltd) | | Metformin hydrochloride | |
| 62605 | Metformin 850mg tablets (Kent Pharmaceuticals Ltd) | | Metformin hydrochloride | |
| 50821 | Metformin 850mg tablets (Pfizer Ltd) | | Metformin hydrochloride | |
| 63045 | Metformin 850mg tablets (Relonchem Ltd) | | Metformin hydrochloride | |
| 34742 | Metformin 850mg tablets (Teva UK Ltd) | | Metformin hydrochloride | |
| 34697 | Metformin 850mg tablets (Wockhardt UK Ltd) | | Metformin hydrochloride | |
| 68636 | Metformin 850mg/5ml oral solution sugar free | | Metformin hydrochloride | |
| 31146 | Metsol 500mg/5ml oral solution (Kappin Ltd) | | Metformin hydrochloride | |
| 27501 | Orabet 500mg Tablet (Lagap) | | Metformin hydrochloride | |
| 42161 | Orabet 500mg Tablet (Sandoz Ltd) | | Metformin hydrochloride | |
| 61043 | Sukkarto SR 1000mg tablets (Morningside Healthcare Ltd) | | Metformin hydrochloride | |
| 61559 | Sukkarto SR 500mg tablets (Morningside Healthcare Ltd) | | Metformin hydrochloride | |
| 54150 | Jentadueto 2.5mg/850mg tablets (Boehringer Ingelheim Ltd) | | Linagliptin/Metformin hydrochloride | |
| 52449 | Linagliptin 2.5mg / Metformin 850mg tablets | | Linagliptin/Metformin hydrochloride | |
| 60497 | Alogliptin 12.5mg / Metformin 1g tablets | | Metformin hydrochloride/Alogliptin benzoate | |
| 59385 | Vipdomet 12.5mg/1000mg tablets (Takeda UK Ltd) | | Metformin hydrochloride/Alogliptin benzoate | |
| 50682 | Jentadueto 2.5mg/1000mg tablets (Boehringer Ingelheim Ltd) | | Metformin hydrochloride/Linagliptin | |
| 52445 | Linagliptin 2.5mg / Metformin 1g tablets | | Metformin hydrochloride/Linagliptin | |
| 56965 | Komboglyze 2.5mg/1000mg tablets (AstraZeneca UK Ltd) | | Metformin hydrochloride/Saxagliptin hydrochloride | |
| 54891 | Saxagliptin 2.5mg / Metformin 1g tablets | | Metformin hydrochloride/Saxagliptin hydrochloride | |
| 39203 | Eucreas 50mg/850mg tablets (Novartis Pharmaceuticals UK Ltd) | | Metformin hydrochloride/Vildagliptin | |
| 37874 | Vildagliptin 50mg / Metformin 850mg tablets | | Metformin hydrochloride/Vildagliptin | |
| 58865 | Komboglyze 2.5mg/850mg tablets (AstraZeneca UK Ltd) | | Saxagliptin hydrochloride/Metformin hydrochloride | |
| 54973 | Saxagliptin 2.5mg / Metformin 850mg tablets | | Saxagliptin hydrochloride/Metformin hydrochloride | |
| 43684 | Janumet 50mg/1000mg tablets (Merck Sharp & Dohme Ltd) | | Sitagliptin phosphate/Metformin hydrochloride | |
| 43619 | Metformin 1g / Sitagliptin 50mg tablets | | Sitagliptin phosphate/Metformin hydrochloride | |
| 38551 | Eucreas 50mg/1000mg tablets (Novartis Pharmaceuticals UK Ltd) | | Vildagliptin/Metformin hydrochloride | |
| 37902 | Vildagliptin 50mg / Metformin 1g tablets | | Vildagliptin/Metformin hydrochloride | |
| 31077 | Competact 15mg/850mg tablets (Takeda UK Ltd) | | Pioglitazone hydrochloride/Metformin hydrochloride | |
| 18220 | Pioglitazone 15mg / Metformin 850mg tablets | | Pioglitazone hydrochloride/Metformin hydrochloride | |
| 17580 | Avandamet 1mg/500mg tablets (GlaxoSmithKline UK Ltd) | | Rosiglitazone maleate/Metformin hydrochloride | |
| 14164 | Avandamet 2mg/1000mg tablets (GlaxoSmithKline UK Ltd) | | Rosiglitazone maleate/Metformin hydrochloride | |
| 7325 | Avandamet 4mg/1000mg tablets (GlaxoSmithKline UK Ltd) | | Rosiglitazone maleate/Metformin hydrochloride | |
| 11604 | Rosiglitazone 1mg / Metformin 500mg tablets | | Rosiglitazone maleate/Metformin hydrochloride | |
| 11717 | Rosiglitazone 2mg / Metformin 1g tablets | | Rosiglitazone maleate/Metformin hydrochloride | |
| 7375 | Rosiglitazone 4mg / Metformin 1g tablets | | Rosiglitazone maleate/Metformin hydrochloride | |
| 63929 | Canagliflozin 50mg / Metformin 1g tablets | | Metformin hydrochloride/Canagliflozin hemihydrate | |
| 64743 | Canagliflozin 50mg / Metformin 850mg tablets | | Metformin hydrochloride/Canagliflozin hemihydrate | |
| 66854 | Vokanamet 50mg/1000mg tablets (Napp Pharmaceuticals Ltd) | | Metformin hydrochloride/Canagliflozin hemihydrate | |
| 60012 | Dapagliflozin 5mg / Metformin 1g tablets | | Metformin hydrochloride/Dapagliflozin | |
| 63031 | Dapagliflozin 5mg / Metformin 850mg tablets | | Metformin hydrochloride/Dapagliflozin | |
| 60643 | Xigduo 5mg/1000mg tablets (AstraZeneca UK Ltd) | | Metformin hydrochloride/Dapagliflozin | |
| 65059 | Xigduo 5mg/850mg tablets (AstraZeneca UK Ltd) | | Metformin hydrochloride/Dapagliflozin | |
| 65066 | Empagliflozin 12.5mg / Metformin 1g tablets | | Metformin hydrochloride/Empagliflozin | |
| 66008 | Synjardy 12.5mg/1000mg tablets (Boehringer Ingelheim Ltd) | | Metformin hydrochloride/Empagliflozin | |
| 66855 | Empagliflozin 12.5mg / Metformin 850mg tablets | | Empagliflozin/Metformin hydrochloride | |
| 65057 | Empagliflozin 5mg / Metformin 1g tablets | | Empagliflozin/Metformin hydrochloride | |
| 65344 | Empagliflozin 5mg / Metformin 850mg tablets | | Empagliflozin/Metformin hydrochloride | |
| 70463 | Synjardy 12.5mg/850mg tablets (Boehringer Ingelheim Ltd) | | Empagliflozin/Metformin hydrochloride | |
| 65083 | Synjardy 5mg/1000mg tablets (Boehringer Ingelheim Ltd) | | Empagliflozin/Metformin hydrochloride | |
| 69370 | Synjardy 5mg/850mg tablets (Boehringer Ingelheim Ltd) | | Empagliflozin/Metformin hydrochloride | |
| 11760 | Metformin with rosiglitazone 1000mg + 2mg Tablet | | Metformin Hydrochloride/Rosiglitazone Maleate | |
| 11737 | Metformin with rosiglitazone 1000mg + 4mg Tablet | | Metformin Hydrochloride/Rosiglitazone Maleate | |
| 11609 | Metformin with rosiglitazone 500mg + 1mg Tablet | | Metformin Hydrochloride/Rosiglitazone Maleate | |
| 11610 | Metformin with rosiglitazone 500mg + 2mg Tablet | | Metformin Hydrochloride/Rosiglitazone Maleate | |
| 6855 | Avandamet 2mg/500mg tablets (GlaxoSmithKline UK Ltd) | | Metformin hydrochloride/Rosiglitazone maleate | |
| 11601 | Rosiglitazone 2mg / Metformin 500mg tablets | | Metformin hydrochloride/Rosiglitazone maleate | |
| 30316 | Metformin with pioglitazone 850mg + 15mg Tablet | | Metformin/Pioglitazone | |
| 24795 | Insulin aspart biphasic 30/70 100units/ml suspension for injection 3ml cartridges | | Insulin aspart/Insulin aspart protamine | |
| 23099 | Insulin aspart biphasic 30/70 100units/ml suspension for injection 3ml pre-filled disposable devices | | Insulin aspart/Insulin aspart protamine | |
| 7228 | NovoMix 30 FlexPen 100units/ml suspension for injection 3ml pre-filled pen (Novo Nordisk Ltd) | | Insulin aspart/Insulin aspart protamine | |
| 71369 | NovoMix 30 FlexPen 100units/ml suspension for injection 3ml pre-filled pen (Sigma Pharmaceuticals Plc) | | Insulin aspart/Insulin aspart protamine | |
| 7267 | NovoMix 30 Penfill 100units/ml suspension for injection 3ml cartridges (Novo Nordisk Ltd) | | Insulin aspart/Insulin aspart protamine | |
| 56489 | NovoMix 30 Penfill 100units/ml suspension for injection 3ml cartridges (Waymade Healthcare Plc) | | Insulin aspart/Insulin aspart protamine | |
| 10067 | Insulin biphasic aspart human pyr 30:70; 100 units/ml Injection | | Insulin Aspart/Insulin Aspart Protamine | |
| 6061 | Novomix 30 30/70 100units/ml Injection (Novo Nordisk Ltd) | | Insulin Aspart/Insulin Aspart Protamine | |
| 20422 | Insuman comb 15 100iu/ml Injection (Aventis Pharma) | | Insulin isophane human/Insulin soluble human | |
| 8895 | Initard 50/50 100unit/ml Injection (Novo Nordisk Ltd) | | Insulin Isophane Porcine/Insulin Soluble Porcine | |
| 66335 | Insulin biphasic isophane porcine 50:50; 100 units/ml Injection | | Insulin Isophane Porcine/Insulin Soluble Porcine | |
| 4715 | Humalog mix 25 25/75 100units/ml Injection (Eli Lilly and Company Ltd) | | Insulin Lispro/Insulin Lispro Protamine | |
| 42395 | Humalog Mix25 100units/ml suspension for injection 10ml vials (Eli Lilly and Company Ltd) | | Insulin lispro/Insulin lispro protamine | |
| 10243 | Humalog Mix25 100units/ml suspension for injection 3ml cartridges (Eli Lilly and Company Ltd) | | Insulin lispro/Insulin lispro protamine | |
| 71395 | Humalog Mix25 100units/ml suspension for injection 3ml cartridges (Sigma Pharmaceuticals Plc) | | Insulin lispro/Insulin lispro protamine | |
| 69583 | Humalog Mix25 100units/ml suspension for injection 3ml cartridges (Waymade Healthcare Plc) | | Insulin lispro/Insulin lispro protamine | |
| 39006 | Humalog Mix25 KwikPen 100units/ml suspension for injection 3ml pre-filled pen (Eli Lilly and Company Ltd) | | Insulin lispro/Insulin lispro protamine | |
| 68031 | Humalog Mix25 KwikPen 100units/ml suspension for injection 3ml pre-filled pen (Sigma Pharmaceuticals Plc) | | Insulin lispro/Insulin lispro protamine | |
| 14270 | Humalog Mix25 Pen 100units/ml suspension for injection 3ml pre-filled pen (Eli Lilly and Company Ltd) | | Insulin lispro/Insulin lispro protamine | |
| 71430 | Humalog Mix25 Pen 100units/ml suspension for injection 3ml pre-filled pen (Waymade Healthcare Plc) | | Insulin lispro/Insulin lispro protamine | |
| 18593 | Humalog Mix50 100units/ml suspension for injection 3ml cartridges (Eli Lilly and Company Ltd) | | Insulin lispro/Insulin lispro protamine | |
| 52522 | Humalog Mix50 KwikPen 100units/ml suspension for injection 3ml pre-filled pen (DE Pharmaceuticals) | | Insulin lispro/Insulin lispro protamine | |
| 39086 | Humalog Mix50 KwikPen 100units/ml suspension for injection 3ml pre-filled pen (Eli Lilly and Company Ltd) | | Insulin lispro/Insulin lispro protamine | |
| 57622 | Humalog Mix50 KwikPen 100units/ml suspension for injection 3ml pre-filled pen (Waymade Healthcare Plc) | | Insulin lispro/Insulin lispro protamine | |
| 10001 | Humalog Mix50 Pen 100units/ml suspension for injection 3ml pre-filled pen (Eli Lilly and Company Ltd) | | Insulin lispro/Insulin lispro protamine | |
| 5250 | Insulin biphasic lispro human prb 25:75; 100 units/ml Injection | | Insulin Lispro/Insulin Lispro Protamine | |
| 27177 | Insulin biphasic lispro human prb 50:50; 100 units/ml Injection | | Insulin Lispro/Insulin Lispro Protamine | |
| 43953 | Insulin lispro biphasic 25/75 100units/ml suspension for injection 10ml vials | | Insulin lispro/Insulin lispro protamine | |
| 28185 | Insulin lispro biphasic 25/75 100units/ml suspension for injection 3ml cartridges | | Insulin lispro/Insulin lispro protamine | |
| 31258 | Insulin lispro biphasic 25/75 100units/ml suspension for injection 3ml pre-filled disposable devices | | Insulin lispro/Insulin lispro protamine | |
| 36146 | Insulin lispro biphasic 50/50 100units/ml suspension for injection 3ml cartridges | | Insulin lispro/Insulin lispro protamine | |
| 35701 | Insulin lispro biphasic 50/50 100units/ml suspension for injection 3ml pre-filled disposable devices | | Insulin lispro/Insulin lispro protamine | |
| 19513 | Humulin M3 100units/ml suspension for injection 10ml vials (Eli Lilly and Company Ltd) | | Insulin soluble human/Insulin isophane human | |
| 60933 | Humulin M3 100units/ml suspension for injection 10ml vials (Sigma Pharmaceuticals Plc) | | Insulin soluble human/Insulin isophane human | |
| 10277 | Humulin M3 100units/ml suspension for injection 3ml cartridges (Eli Lilly and Company Ltd) | | Insulin soluble human/Insulin isophane human | |
| 67324 | Humulin M3 100units/ml suspension for injection 3ml cartridges (Waymade Healthcare Plc) | | Insulin soluble human/Insulin isophane human | |
| 43991 | Humulin M3 KwikPen 100units/ml suspension for injection 3ml pre-filled pen (Eli Lilly and Company Ltd) | | Insulin soluble human/Insulin isophane human | |
| 56857 | Insulin isophane biphasic human 15/85 100units/ml suspension for injection 3ml cartridges | | Insulin soluble human/Insulin isophane human | |
| 36194 | Insulin isophane biphasic human 25/75 100units/ml suspension for injection 3ml cartridges | | Insulin soluble human/Insulin isophane human | |
| 44378 | Insulin isophane biphasic human 25/75 100units/ml suspension for injection 3ml pre-filled disposable devices | | Insulin soluble human/Insulin isophane human | |
| 42954 | Insulin isophane biphasic human 25/75 100units/ml suspension for injection 5ml vials | | Insulin soluble human/Insulin isophane human | |
| 21232 | Insulin isophane biphasic human 30/70 100units/ml suspension for injection 10ml vials | | Insulin soluble human/Insulin isophane human | |
| 16152 | Insulin isophane biphasic human 30/70 100units/ml suspension for injection 3ml cartridges | | Insulin soluble human/Insulin isophane human | |
| 19878 | Insulin isophane biphasic human 30/70 100units/ml suspension for injection 3ml pre-filled disposable devices | | Insulin soluble human/Insulin isophane human | |
| 28096 | Insulin isophane biphasic human 50/50 100units/ml suspension for injection 3ml cartridges | | Insulin soluble human/Insulin isophane human | |
| 41120 | Insulin isophane biphasic human 50/50 100units/ml suspension for injection 3ml pre-filled disposable devices | | Insulin soluble human/Insulin isophane human | |
| 45158 | Insuman Comb 15 100units/ml suspension for injection 3ml cartridges (Sanofi) | | Insulin soluble human/Insulin isophane human | |
| 30819 | Insuman Comb 15 100units/ml suspension for injection 3ml pre-filled OptiSet pen (Sanofi) | | Insulin soluble human/Insulin isophane human | |
| 24993 | Insuman Comb 25 100units/ml suspension for injection 3ml cartridges (Sanofi) | | Insulin soluble human/Insulin isophane human | |
| 25133 | Insuman Comb 25 100units/ml suspension for injection 3ml pre-filled OptiSet pen (Sanofi) | | Insulin soluble human/Insulin isophane human | |
| 44480 | Insuman Comb 25 100units/ml suspension for injection 3ml pre-filled SoloStar pen (Sanofi) | | Insulin soluble human/Insulin isophane human | |
| 24002 | Insuman Comb 25 100units/ml suspension for injection 5ml vials (Sanofi) | | Insulin soluble human/Insulin isophane human | |
| 35253 | Insuman Comb 50 100units/ml suspension for injection 3ml cartridges (Sanofi) | | Insulin soluble human/Insulin isophane human | |
| 31205 | Insuman Comb 50 100units/ml suspension for injection 3ml pre-filled OptiSet pen (Sanofi) | | Insulin soluble human/Insulin isophane human | |
| 7300 | Mixtard 30 100units/ml suspension for injection 10ml vials (Novo Nordisk Ltd) | | Insulin soluble human/Insulin isophane human | |
| 5845 | Mixtard 30 InnoLet 100units/ml suspension for injection 3ml pre-filled pen (Novo Nordisk Ltd) | | Insulin soluble human/Insulin isophane human | |
| 7231 | Mixtard 30 Penfill 100units/ml suspension for injection 3ml cartridges (Novo Nordisk Ltd) | | Insulin soluble human/Insulin isophane human | |
| 10915 | Humaject m1 100iu/ml M1 pen (Eli Lilly and Company Ltd) | | Insulin Soluble Human/Insulin Isophane Human | |
| 10910 | Humaject m2 100iu/ml M2 pen (Eli Lilly and Company Ltd) | | Insulin Soluble Human/Insulin Isophane Human | |
| 17809 | Humaject m4 100iu/ml M4 pen (Eli Lilly and Company Ltd) | | Insulin Soluble Human/Insulin Isophane Human | |
| 22155 | Humaject m5 100iu/ml M5 pen (Eli Lilly and Company Ltd) | | Insulin Soluble Human/Insulin Isophane Human | |
| 1649 | Human actraphane 100iu/ml Injection (Novo Nordisk Ltd) | | Insulin Soluble Human/Insulin Isophane Human | |
| 34097 | Human initard 50/50 100unit/ml Injection (Novo Nordisk Ltd) | | Insulin Soluble Human/Insulin Isophane Human | |
| 4199 | Humulin m1 100unit/ml M1 injection (Eli Lilly and Company Ltd) | | Insulin Soluble Human/Insulin Isophane Human | |
| 4198 | Humulin m3 100unit/ml M3 injection (Eli Lilly and Company Ltd) | | Insulin Soluble Human/Insulin Isophane Human | |
| 11107 | Humulin m4 100unit/ml M4 injection (Eli Lilly and Company Ltd) | | Insulin Soluble Human/Insulin Isophane Human | |
| 33167 | Insulin biphasic isophane human crb 25:75; 100 units/ml Injection | | Insulin Soluble Human/Insulin Isophane Human | |
| 54462 | Insulin biphasic isophane human emp 25:75; 100 units/ml Injection | | Insulin Soluble Human/Insulin Isophane Human | |
| 13837 | Insulin biphasic isophane human prb 10:90; 100 units/ml Injection | | Insulin Soluble Human/Insulin Isophane Human | |
| 14644 | Insulin biphasic isophane human prb 20:80; 100 units/ml Injection | | Insulin Soluble Human/Insulin Isophane Human | |
| 29837 | Insulin biphasic isophane human prb 25:75; 100 units/ml Injection | | Insulin Soluble Human/Insulin Isophane Human | |
| 9341 | Insulin biphasic isophane human prb 30:70; 100 units/ml Injection | | Insulin Soluble Human/Insulin Isophane Human | |
| 21374 | Insulin biphasic isophane human prb 40:60; 100 units/ml Injection | | Insulin Soluble Human/Insulin Isophane Human | |
| 21110 | Insulin biphasic isophane human prb 50:50; 100 units/ml Injection | | Insulin Soluble Human/Insulin Isophane Human | |
| 14649 | Insulin biphasic isophane human pyr 10:90; 100 units/ml Injection | | Insulin Soluble Human/Insulin Isophane Human | |
| 11055 | Insulin biphasic isophane human pyr 20:80; 100 units/ml Injection | | Insulin Soluble Human/Insulin Isophane Human | |
| 11056 | Insulin biphasic isophane human pyr 30:70; 100 units/ml Injection | | Insulin Soluble Human/Insulin Isophane Human | |
| 21395 | Insulin biphasic isophane human pyr 40:60; 100 units/ml Injection | | Insulin Soluble Human/Insulin Isophane Human | |
| 15199 | Insuman comb 25 100iu/ml Injection (Aventis Pharma) | | Insulin Soluble Human/Insulin Isophane Human | |
| 21554 | Insuman comb 50 100iu/ml Injection (Aventis Pharma) | | Insulin soluble human/Insulin isophane human | |
| 5255 | Mixtard 10 penfill 100 100iu/ml Penfill (Novo Nordisk Ltd) | | Insulin Soluble Human/Insulin Isophane Human | |
| 3551 | Mixtard 20 penfill 100 100iu/ml Penfill (Novo Nordisk Ltd) | | Insulin Soluble Human/Insulin Isophane Human | |
| 2929 | Mixtard 30 100iu/ml GE injection (Novo Nordisk Ltd) | | Insulin Soluble Human/Insulin Isophane Human | |
| 2454 | Mixtard 30 penfill 100 100iu/ml Penfill (Novo Nordisk Ltd) | | Insulin Soluble Human/Insulin Isophane Human | |
| 1805 | Mixtard 30/70 100unit/ml Injection (Novo Nordisk Ltd) | | Insulin Soluble Human/Insulin Isophane Human | |
| 3550 | Mixtard 40 penfill 100 100iu/ml Penfill (Novo Nordisk Ltd) | | Insulin Soluble Human/Insulin Isophane Human | |
| 4790 | Mixtard 50 penfill 100 100iu/ml Penfill (Novo Nordisk Ltd) | | Insulin soluble human/Insulin isophane human | |
| 3439 | Penmix 10/90 Pen (Novo Nordisk Ltd) | | Insulin Soluble Human/Insulin Isophane Human | |
| 3396 | Penmix 10/90 Penfill (Novo Nordisk Ltd) | | Insulin Soluble Human/Insulin Isophane Human | |
| 2220 | Penmix 20/80 Pen (Novo Nordisk Ltd) | | Insulin Soluble Human/Insulin Isophane Human | |
| 10484 | Penmix 20/80 Penfill (Novo Nordisk Ltd) | | Insulin Soluble Human/Insulin Isophane Human | |
| 27614 | Penmix 30/70 100iu/ml Injection (Novo Nordisk Ltd) | | Insulin Soluble Human/Insulin Isophane Human | |
| 1806 | Penmix 30/70 100iu/ml Penfill (Novo Nordisk Ltd) | | Insulin Soluble Human/Insulin Isophane Human | |
| 21347 | Penmix 40/60 100iu/ml Injection (Novo Nordisk Ltd) | | Insulin Soluble Human/Insulin Isophane Human | |
| 10887 | Penmix 40/60 100iu/ml Penfill (Novo Nordisk Ltd) | | Insulin Soluble Human/Insulin Isophane Human | |
| 17731 | Penmix 50/50 100iu/ml Injection (Novo Nordisk Ltd) | | Insulin soluble human/Insulin isophane human | |
| 8203 | Penmix 50/50 100iu/ml Penfill (Novo Nordisk Ltd) | | Insulin soluble human/Insulin isophane human | |
| 22058 | Pur-in mix 15/85 Injection (C P Pharmaceuticals Ltd) | | Insulin Soluble Human/Insulin Isophane Human | |
| 26403 | Pur-in mix 25/75 Injection (C P Pharmaceuticals Ltd) | | Insulin Soluble Human/Insulin Isophane Human | |
| 7793 | HumaJect M3 Pen 100units/ml suspension for injection (Eli Lilly and Company Ltd) | | Insulin soluble human/Insulin isophane human | |
| 50691 | Human Mixtard 20 Penfill 100units/ml suspension for injection 1.5ml cartridges (Novo Nordisk Ltd) | | Insulin soluble human/Insulin isophane human | |
| 52722 | Human Mixtard 30 Penfill 100units/ml suspension for injection 1.5ml cartridges (Novo Nordisk Ltd) | | Insulin soluble human/Insulin isophane human | |
| 12818 | Human Mixtard 50 100units/ml suspension for injection 10ml vials (Novo Nordisk Ltd) | | Insulin soluble human/Insulin isophane human | |
| 4093 | Humulin M2 100units/ml suspension for injection 3ml cartridges (Eli Lilly and Company Ltd) | | Insulin soluble human/Insulin isophane human | |
| 57620 | Humulin M3 100units/ml suspension for injection 10ml vials (Mawdsley-Brooks & Company Ltd) | | Insulin soluble human/Insulin isophane human | |
| 16160 | Humulin M3 Pen 100units/ml suspension for injection 3ml pre-filled pen (Eli Lilly and Company Ltd) | | Insulin soluble human/Insulin isophane human | |
| 8841 | Humulin M5 100units/ml suspension for injection 10ml vials (Eli Lilly and Company Ltd) | | Insulin soluble human/Insulin isophane human | |
| 25736 | Insulin isophane biphasic human 10/90 100units/ml suspension for injection 3ml cartridges | | Insulin soluble human/Insulin isophane human | |
| 25735 | Insulin isophane biphasic human 20/80 100units/ml suspension for injection 3ml cartridges | | Insulin soluble human/Insulin isophane human | |
| 21422 | Insulin isophane biphasic human 40/60 100units/ml suspension for injection 3ml cartridges | | Insulin soluble human/Insulin isophane human | |
| 22697 | Insulin isophane biphasic human 50/50 100units/ml suspension for injection 1.5ml cartridges | | Insulin soluble human/Insulin isophane human | |
| 33232 | Insulin isophane biphasic human 50/50 100units/ml suspension for injection 5ml vials | | Insulin soluble human/Insulin isophane human | |
| 2456 | Mixtard 10 NovoLet 100units/ml suspension for injection (Novo Nordisk Ltd) | | Insulin soluble human/Insulin isophane human | |
| 10245 | Mixtard 10 Penfill 100units/ml suspension for injection 3ml cartridges (Novo Nordisk Ltd) | | Insulin soluble human/Insulin isophane human | |
| 2455 | Mixtard 20 NovoLet 100units/ml suspension for injection (Novo Nordisk Ltd) | | Insulin soluble human/Insulin isophane human | |
| 7319 | Mixtard 20 Penfill 100units/ml suspension for injection 3ml cartridges (Novo Nordisk Ltd) | | Insulin soluble human/Insulin isophane human | |
| 60938 | Mixtard 30 100units/ml suspension for injection 10ml vials (Waymade Healthcare Plc) | | Insulin soluble human/Insulin isophane human | |
| 67267 | Mixtard 30 InnoLet 100units/ml suspension for injection 3ml pre-filled pen (Waymade Healthcare Plc) | | Insulin soluble human/Insulin isophane human | |
| 2221 | Mixtard 30 NovoLet 100units/ml suspension for injection (Novo Nordisk Ltd) | | Insulin soluble human/Insulin isophane human | |
| 71340 | Mixtard 30 Penfill 100units/ml suspension for injection 3ml cartridges (Waymade Healthcare Plc) | | Insulin soluble human/Insulin isophane human | |
| 2812 | Mixtard 40 NovoLet 100units/ml suspension for injection (Novo Nordisk Ltd) | | Insulin soluble human/Insulin isophane human | |
| 10244 | Mixtard 40 Penfill 100units/ml suspension for injection 3ml cartridges (Novo Nordisk Ltd) | | Insulin soluble human/Insulin isophane human | |
| 5933 | Mixtard 50 NovoLet 100units/ml suspension for injection (Novo Nordisk Ltd) | | Insulin soluble human/Insulin isophane human | |
| 13277 | Mixtard 50 Penfill 100units/ml suspension for injection 3ml cartridges (Novo Nordisk Ltd) | | Insulin soluble human/Insulin isophane human | |
| 67266 | Mixtard 50 Penfill 100units/ml suspension for injection 3ml cartridges (Waymade Healthcare Plc) | | Insulin soluble human/Insulin isophane human | |
| 24800 | Hypurin Porcine 30/70 Mix 100units/ml suspension for injection 10ml vials (Wockhardt UK Ltd) | | Insulin soluble porcine/Insulin isophane porcine | |
| 20995 | Hypurin Porcine 30/70 Mix 100units/ml suspension for injection 3ml cartridges (Wockhardt UK Ltd) | | Insulin soluble porcine/Insulin isophane porcine | |
| 27280 | Insulin isophane biphasic porcine 30/70 100units/ml suspension for injection 10ml vials | | Insulin soluble porcine/Insulin isophane porcine | |
| 36031 | Insulin isophane biphasic porcine 30/70 100units/ml suspension for injection 3ml cartridges | | Insulin soluble porcine/Insulin isophane porcine | |
| 9618 | Hypurin Porcine 30/70 Mix 100units/ml suspension for injection 1.5ml cartridges (C P Pharmaceuticals Ltd) | | Insulin soluble porcine/Insulin isophane porcine | |
| 14619 | Insulin isophane biphasic porcine 30/70 100units/ml suspension for injection 1.5ml cartridges | | Insulin soluble porcine/Insulin isophane porcine | |
| 2459 | Pork Mixtard 30 100units/ml suspension for injection 10ml vials (Novo Nordisk Ltd) | | Insulin soluble porcine/Insulin isophane porcine | |
| 8322 | Insulin zinc suspension mixed human pyr 100unit/ml Injection | | Insulin Zinc Suspension Crystalline Human/Insulin Zinc Suspension Mixed Human | |
| 17712 | Hypurin Bovine Lente 100units/ml suspension for injection 10ml vials (Wockhardt UK Ltd) | | Insulin zinc suspension mixed bovine | |
| 12035 | Insulin zinc mixed bovine 100units/ml suspension for injection 10ml vials | | Insulin zinc suspension mixed bovine | |
| 16700 | Insulin zinc mixed bovine vial 100unit/ml Sterile suspension injection | | Insulin Zinc Suspension Mixed Bovine | |
| 41834 | Insulin zinc suspension lente 100iu/ml Injection (Celltech Pharma Europe Ltd) | | Insulin zinc suspension mixed bovine | |
| 16682 | Tempulin 100unit/ml Injection (Knoll Ltd) | | Insulin zinc suspension mixed bovine | |
| 10547 | Humulin Lente 100units/ml suspension for injection 10ml vials (Eli Lilly and Company Ltd) | | Insulin zinc suspension mixed human | |
| 18461 | Insulin zinc mixed human 100units/ml suspension for injection 10ml vials | | Insulin zinc suspension mixed human | |
| 1587 | Monotard 100units/ml suspension for injection 10ml vials (Novo Nordisk Ltd) | | Insulin zinc suspension mixed human | |
| 13416 | Insulin biphasic 100 units/ml Injection | | Insulin/Insulin Soluble Porcine | |
| 4163 | Rapitard MC 100unit/ml Injection (Novo Nordisk Ltd) | | Insulin/Insulin Soluble Porcine | |
| 26498 | Insulin zinc suspension mixed bovine and porcine 100unit/ml Injection | | Pork Insulin/Insulin Zinc Suspension Mixed Bovine | |
| 4784 | Lentard mc 100unit/ml Injection (Novo Nordisk Ltd) | | Pork Insulin/Insulin Zinc Suspension Mixed Bovine | |
| 20287 | Actos 15mg tablets (Takeda UK Ltd) | | Pioglitazone hydrochloride | |
| 20889 | Actos 30mg tablets (Takeda UK Ltd) | | Pioglitazone hydrochloride | |
| 19472 | Actos 45mg tablets (Takeda UK Ltd) | | Pioglitazone hydrochloride | |
| 68934 | Diabiom 30mg tablets (Tillomed Laboratories Ltd) | | Pioglitazone hydrochloride | |
| 64900 | Glidipion 30mg tablets (Actavis UK Ltd) | | Pioglitazone hydrochloride | |
| 548 | Pioglitazone 15mg tablets | | Pioglitazone hydrochloride | |
| 56208 | Pioglitazone 15mg tablets (A A H Pharmaceuticals Ltd) | | Pioglitazone hydrochloride | |
| 65563 | Pioglitazone 15mg tablets (Alliance Healthcare (Distribution) Ltd) | | Pioglitazone hydrochloride | |
| 9699 | Pioglitazone 30mg tablets | | Pioglitazone hydrochloride | |
| 48139 | Pioglitazone 30mg tablets (A A H Pharmaceuticals Ltd) | | Pioglitazone hydrochloride | |
| 62426 | Pioglitazone 30mg tablets (Accord Healthcare Ltd) | | Pioglitazone hydrochloride | |
| 57659 | Pioglitazone 30mg tablets (Actavis UK Ltd) | | Pioglitazone hydrochloride | |
| 65562 | Pioglitazone 30mg tablets (Alliance Healthcare (Distribution) Ltd) | | Pioglitazone hydrochloride | |
| 69885 | Pioglitazone 30mg tablets (Consilient Health Ltd) | | Pioglitazone hydrochloride | |
| 63421 | Pioglitazone 30mg tablets (Teva UK Ltd) | | Pioglitazone hydrochloride | |
| 10051 | Pioglitazone 45mg tablets | | Pioglitazone hydrochloride | |
| 63046 | Pioglitazone 45mg tablets (A A H Pharmaceuticals Ltd) | | Pioglitazone hydrochloride | |
| 63107 | Pioglitazone 45mg tablets (Waymade Healthcare Plc) | | Pioglitazone hydrochloride | |
| 48120 | Avandia 2mg Tablet (GlaxoSmithKline UK Ltd) | | Rosiglitazone Maleate | |
| 37617 | Rosiglitazone 2mg tablet | | Rosiglitazone Maleate | |
| 9662 | Avandia 4mg tablets (GlaxoSmithKline UK Ltd) | | Rosiglitazone maleate | |
| 15232 | Avandia 8mg tablets (GlaxoSmithKline UK Ltd) | | Rosiglitazone maleate | |
| 469 | Rosiglitazone 4mg tablets | | Rosiglitazone maleate | |
| 5227 | Rosiglitazone 8mg tablets | | Rosiglitazone maleate | |
| 56376 | Rosiglitazone 4mg with glimepiride 4mg tablet | | Rosiglitazone Maleate/Glimepiride | |
| 60211 | Canagliflozin 100mg tablets | | Canagliflozin hemihydrate | |
| 60386 | Canagliflozin 300mg tablets | | Canagliflozin hemihydrate | |
| 60430 | Invokana 100mg tablets (Napp Pharmaceuticals Ltd) | | Canagliflozin hemihydrate | |
| 60379 | Invokana 300mg tablets (Napp Pharmaceuticals Ltd) | | Canagliflozin hemihydrate | |
| 54182 | Dapagliflozin 10mg tablets | | Dapagliflozin | |
| 54265 | Dapagliflozin 5mg tablets | | Dapagliflozin | |
| 54203 | Forxiga 10mg tablets (AstraZeneca UK Ltd) | | Dapagliflozin | |
| 63516 | Forxiga 10mg tablets (Waymade Healthcare Plc) | | Dapagliflozin | |
| 54480 | Forxiga 5mg tablets (AstraZeneca UK Ltd) | | Dapagliflozin | |
| 61756 | Empagliflozin 10mg tablets | | Empagliflozin | |
| 62172 | Empagliflozin 25mg tablets | | Empagliflozin | |
| 62760 | Jardiance 10mg tablets (Boehringer Ingelheim Ltd) | | Empagliflozin | |
| 64217 | Jardiance 25mg tablets (Boehringer Ingelheim Ltd) | | Empagliflozin | |
| 19491 | Apidra 100units/ml solution for injection 10ml vials (Sanofi) | | Insulin glulisine | |
| 14345 | Apidra 100units/ml solution for injection 3ml cartridges (Sanofi) | | Insulin glulisine | |
| 29953 | Apidra 100units/ml solution for injection 3ml OptiClik cartridges (Sanofi) | | Insulin glulisine | |
| 21583 | Apidra 100units/ml solution for injection 3ml pre-filled OptiSet pen (Sanofi) | | Insulin glulisine | |
| 36920 | Apidra 100units/ml solution for injection 3ml pre-filled SoloStar pen (Sanofi) | | Insulin glulisine | |
| 28442 | Insulin glulisine 100unit/ml Solution for injection | | Insulin Glulisine | |
| 28101 | Insulin glulisine 100units/ml solution for injection 10ml vials | | Insulin glulisine | |
| 14299 | Insulin glulisine 100units/ml solution for injection 3ml cartridges | | Insulin glulisine | |
| 21590 | Insulin glulisine 100units/ml solution for injection 3ml pre-filled disposable devices | | Insulin glulisine | |
| 62276 | Humulin R 500units/ml solution for injection 20ml vials (Imported (United States)) | | Insulin human | |
| 60951 | Insulin human 100units/ml solution for injection 10ml vials | | Insulin human | |
| 67429 | Insulin human 100units/ml solution for injection 3.15ml cartridges | | Insulin human | |
| 53710 | Insulin human 500units/ml solution for injection 20ml vials | | Insulin human | |
| 57529 | Humalog 100units/ml solution for injection 10ml vials (Dowelhurst Ltd) | | Insulin lispro | |
| 18224 | Humalog 100units/ml solution for injection 10ml vials (Eli Lilly and Company Ltd) | | Insulin lispro | |
| 7318 | Humalog 100units/ml solution for injection 3ml cartridges (Eli Lilly and Company Ltd) | | Insulin lispro | |
| 55603 | Humalog KwikPen 100units/ml solution for injection 3ml pre-filled pen (DE Pharmaceuticals) | | Insulin lispro | |
| 38986 | Humalog KwikPen 100units/ml solution for injection 3ml pre-filled pen (Eli Lilly and Company Ltd) | | Insulin lispro | |
| 57564 | Humalog KwikPen 100units/ml solution for injection 3ml pre-filled pen (Waymade Healthcare Plc) | | Insulin lispro | |
| 63464 | Humalog KwikPen 200units/ml solution for injection 3ml pre-filled pen (Eli Lilly and Company Ltd) | | Insulin lispro | |
| 10264 | Humalog Pen 100units/ml solution for injection 3ml pre-filled pen (Eli Lilly and Company Ltd) | | Insulin lispro | |
| 26060 | Insulin lispro 100units/ml solution for injection 10ml vials | | Insulin lispro | |
| 14313 | Insulin lispro 100units/ml solution for injection 3ml cartridges | | Insulin lispro | |
| 14362 | Insulin lispro 100units/ml solution for injection 3ml pre-filled disposable devices | | Insulin lispro | |
| 322 | Humalog 100units/ml solution for injection 1.5ml cartridges (Eli Lilly and Company Ltd) | | Insulin lispro | |
| 5214 | Insulin lispro 100units/ml solution for injection 1.5ml cartridges | | Insulin lispro | |
| 12297 | Hypurin bovine neutral 100unit/ml Injection (C P Pharmaceuticals Ltd) | | Insulin Soluble Bovine | |
| 14339 | Hypurin Bovine Neutral 100units/ml solution for injection 10ml vials (Wockhardt UK Ltd) | | Insulin soluble bovine | |
| 23231 | Hypurin Bovine Neutral 100units/ml solution for injection 3ml cartridges (Wockhardt UK Ltd) | | Insulin soluble bovine | |
| 63679 | Hypurin soluble 100iu/ml Injection (C P Pharmaceuticals Ltd) | | Insulin Soluble Bovine | |
| 10572 | Insulin soluble bovine 100unit/ml Injection | | Insulin Soluble Bovine | |
| 18592 | Insulin soluble bovine 100units/ml solution for injection 10ml vials | | Insulin soluble bovine | |
| 14938 | Insulin soluble bovine cartridge 100unit/ml Solution for injection | | Insulin Soluble Bovine | |
| 47360 | Neutral insulin 100unit/ml Injection (Celltech Pharma Europe Ltd) | | Insulin Soluble Bovine | |
| 24593 | Neutral insulin bovine 100unit/ml Injection | | Insulin Soluble Bovine | |
| 7349 | Actrapid 100units/ml solution for injection 10ml vials (Novo Nordisk Ltd) | | Insulin soluble human | |
| 21235 | Humulin S 100units/ml solution for injection 10ml vials (Eli Lilly and Company Ltd) | | Insulin soluble human | |
| 14944 | Humulin S 100units/ml solution for injection 3ml cartridges (Eli Lilly and Company Ltd) | | Insulin soluble human | |
| 27402 | Insulin soluble human 100units/ml solution for injection 10ml vials | | Insulin soluble human | |
| 16129 | Insulin soluble human 100units/ml solution for injection 3ml cartridges | | Insulin soluble human | |
| 36430 | Insulin soluble human 100units/ml solution for injection 3ml pre-filled disposable devices | | Insulin soluble human | |
| 22983 | Insuman Rapid 100units/ml solution for injection 3ml cartridges (Sanofi) | | Insulin soluble human | |
| 23993 | Insuman Rapid 100units/ml solution for injection 3ml pre-filled OptiSet pen (Sanofi) | | Insulin soluble human | |
| 1588 | Actrapid 100iu/ml Injection (Novo Nordisk Ltd) | | Insulin Soluble Human | |
| 1592 | Actrapid penfill 100 100iu/ml Penfill (Novo Nordisk Ltd) | | Insulin Soluble Human | |
| 1840 | Humulin s 100unit/ml Injection (Eli Lilly and Company Ltd) | | Insulin Soluble Human | |
| 26621 | Insulin soluble human crb 100iu/ml Injection | | Insulin Soluble Human | |
| 15710 | Insulin soluble human emp 100unit/ml Injection | | Insulin Soluble Human | |
| 12654 | Insulin soluble human prb 100unit/ml Injection | | Insulin Soluble Human | |
| 12638 | Insulin soluble human pyr 100unit/ml Injection | | Insulin Soluble Human | |
| 22945 | Insuman rapid 100iu/ml Injection (Aventis Pharma) | | Insulin Soluble Human | |
| 17336 | Novopen 100unit/ml Injection device (Novo Nordisk Ltd) | | Insulin Soluble Human | |
| 41959 | Penject 100unit/ml Injection device (Hypoguard Ltd) | | Insulin Soluble Human | |
| 24846 | Pur-in neutral 100unit/ml Injection (C P Pharmaceuticals Ltd) | | Insulin Soluble Human | |
| 1594 | Actrapid NovoLet 100units/ml solution for injection (Novo Nordisk Ltd) | | Insulin soluble human | |
| 56502 | Actrapid Penfill 100units/ml solution for injection 3ml cartridges (Novo Nordisk Ltd) | | Insulin soluble human | |
| 9565 | HumaJect S Pen 100units/ml solution for injection (Eli Lilly and Company Ltd) | | Insulin soluble human | |
| 56115 | Human Actrapid Penfill 100units/ml solution for injection 1.5ml cartridges (Novo Nordisk Ltd) | | Insulin soluble human | |
| 4706 | Velosulin 100units/ml solution for injection 10ml vials (Novo Nordisk Ltd) | | Insulin soluble human | |
| 9521 | Pork Actrapid 100units/ml solution for injection 10ml vials (Novo Nordisk Ltd) | | Insulin soluble porcine | |
| 30209 | Actrapid mc 100unit/ml Injection (Arun Products Ltd) | | Insulin soluble porcine | |
| 13622 | Hypurin porcine neutral 100unit/ml Injection (C P Pharmaceuticals Ltd) | | Insulin soluble porcine | |
| 26098 | Hypurin Porcine Neutral 100units/ml solution for injection 10ml vials (Wockhardt UK Ltd) | | Insulin soluble porcine | |
| 14930 | Hypurin Porcine Neutral 100units/ml solution for injection 3ml cartridges (Wockhardt UK Ltd) | | Insulin soluble porcine | |
| 27396 | Insulin soluble porcine 100units/ml solution for injection 10ml vials | | Insulin soluble porcine | |
| 25479 | Insulin soluble porcine 100units/ml solution for injection 3ml cartridges | | Insulin soluble porcine | |
| 1842 | Pork velosulin 100unit/ml Injection (Novo Nordisk Ltd) | | Insulin soluble porcine | |
| 36513 | Velosulin cartridge 100unit/ml Injection (Novo Nordisk Ltd) | | Insulin soluble porcine | |
| 4129 | Insulin soluble porcine 100units/ml solution for injection 1.5ml cartridges | | Insulin soluble porcine | |
| 9376 | Insulin zinc suspension crystalline human pyr 100unit/ml long acting Injection | | Insulin Zinc Suspension Crystalline Human | |
| 7537 | Humulin Zn 100units/ml suspension for injection 10ml vials (Eli Lilly and Company Ltd) | | Insulin zinc suspension crystalline human | |
| 18931 | Insulin zinc crystalline human 100units/ml suspension for injection 10ml vials | | Insulin zinc suspension crystalline human | |
| 1844 | Ultratard 100units/ml suspension for injection 10ml vials (Novo Nordisk Ltd) | | Insulin zinc suspension crystalline human | |
| 44251 | Insulin zinc suspension mixed porcine 100unit/ml Injection | | Pork Insulin | |
| 34031 | Monotard mc 100unit/ml Injection (Novo Nordisk Ltd) | | Pork Insulin | |
| 12299 | Semitard mc 100unit/ml Injection (Novo Nordisk Ltd) | | Pork Insulin | |
| 69715 | Fiasp 100units/ml solution for injection 10ml vials (Novo Nordisk Ltd) | | Insulin aspart | |
| 69823 | Fiasp FlexTouch 100units/ml solution for injection 3ml pre-filled pen (Novo Nordisk Ltd) | | Insulin aspart | |
| 70055 | Fiasp Penfill 100units/ml solution for injection 3ml cartridges (Novo Nordisk Ltd) | | Insulin aspart | |
| 62180 | Insulin aspart 100units/ml solution for injection 1.6ml cartridges | | Insulin aspart | |
| 29567 | Insulin aspart 100units/ml solution for injection 10ml vials | | Insulin aspart | |
| 16142 | Insulin aspart 100units/ml solution for injection 3ml cartridges | | Insulin aspart | |
| 19877 | Insulin aspart 100units/ml solution for injection 3ml pre-filled disposable devices | | Insulin aspart | |
| 6447 | Insulin aspart human pyr 100 iu/ml Injection | | Insulin Aspart | |
| 6209 | NovoRapid 100units/ml solution for injection 10ml vials (Novo Nordisk Ltd) | | Insulin aspart | |
| 67313 | NovoRapid 100units/ml solution for injection 10ml vials (Sigma Pharmaceuticals Plc) | | Insulin aspart | |
| 53118 | NovoRapid FlexPen 100units/ml solution for injection 3ml pre-filled pen (Mawdsley-Brooks & Company Ltd) | | Insulin aspart | |
| 5892 | NovoRapid FlexPen 100units/ml solution for injection 3ml pre-filled pen (Novo Nordisk Ltd) | | Insulin aspart | |
| 59533 | NovoRapid FlexPen 100units/ml solution for injection 3ml pre-filled pen (Sigma Pharmaceuticals Plc) | | Insulin aspart | |
| 46666 | NovoRapid FlexTouch 100units/ml solution for injection 3ml pre-filled pen (Novo Nordisk Ltd) | | Insulin aspart | |
| 53251 | NovoRapid Penfill 100units/ml solution for injection 3ml cartridges (DE Pharmaceuticals) | | Insulin aspart | |
| 49108 | NovoRapid Penfill 100units/ml solution for injection 3ml cartridges (Necessity Supplies Ltd) | | Insulin aspart | |
| 5021 | NovoRapid Penfill 100units/ml solution for injection 3ml cartridges (Novo Nordisk Ltd) | | Insulin aspart | |
| 51743 | NovoRapid Penfill 100units/ml solution for injection 3ml cartridges (Sigma Pharmaceuticals Plc) | | Insulin aspart | |
| 61845 | NovoRapid PumpCart 100units/ml solution for injection 1.6ml cartridges (Novo Nordisk Ltd) | | Insulin aspart | |
| 67231 | NovoRapid FlexPen 100units/ml solution for injection 3ml pre-filled pen (Dowelhurst Ltd) | | Insulin aspart | |
| 11337 | NovoRapid Novolet 100units/ml solution for injection (Novo Nordisk Ltd) | | Insulin aspart | |
| 16602 | Calabren 2.5mg Tablet (Berk Pharmaceuticals Ltd) | | Glibenclamide | |
| 26218 | Calabren 5mg Tablet (Berk Pharmaceuticals Ltd) | | Glibenclamide | |
| 57601 | Daonil 5mg tablets (Dowelhurst Ltd) | | Glibenclamide | |
| 7744 | Daonil 5mg tablets (Sanofi) | | Glibenclamide | |
| 4862 | Diabetamide 2.5mg tablets (Ashbourne Pharmaceuticals Ltd) | | Glibenclamide | |
| 21832 | Diabetamide 5mg tablets (Ashbourne Pharmaceuticals Ltd) | | Glibenclamide | |
| 8976 | Euglucon 2.5mg tablets (Aventis Pharma) | | Glibenclamide | |
| 13331 | Euglucon 5mg tablets (Sanofi) | | Glibenclamide | |
| 2219 | Glibenclamide 2.5mg tablets | | Glibenclamide | |
| 34676 | Glibenclamide 2.5mg tablets (A A H Pharmaceuticals Ltd) | | Glibenclamide | |
| 34706 | Glibenclamide 2.5mg tablets (IVAX Pharmaceuticals UK Ltd) | | Glibenclamide | |
| 41593 | Glibenclamide 2.5mg tablets (Teva UK Ltd) | | Glibenclamide | |
| 34507 | Glibenclamide 2.5mg tablets (Wockhardt UK Ltd) | | Glibenclamide | |
| 1254 | Glibenclamide 5mg tablets | | Glibenclamide | |
| 41559 | Glibenclamide 5mg tablets (A A H Pharmaceuticals Ltd) | | Glibenclamide | |
| 41558 | Glibenclamide 5mg tablets (Teva UK Ltd) | | Glibenclamide | |
| 34563 | Glibenclamide 5mg tablets (Wockhardt UK Ltd) | | Glibenclamide | |
| 21424 | Glibenclamide 5mg/5ml oral suspension | | Glibenclamide | |
| 25636 | Libanil 2.5mg Tablet (Approved Prescription Services Ltd) | | Glibenclamide | |
| 31474 | Libanil 5mg Tablet (Approved Prescription Services Ltd) | | Glibenclamide | |
| 28708 | Malix 2.5mg Tablet (Lagap) | | Glibenclamide | |
| 30460 | Malix 5mg Tablet (Lagap) | | Glibenclamide | |
| 7912 | Semi-Daonil 2.5mg tablets (Sanofi) | | Glibenclamide | |
| 70432 | Bilxona 30mg modified-release tablets (Actavis UK Ltd) | | Gliclazide | |
| 70490 | Bilxona 60mg modified-release tablets (Actavis UK Ltd) | | Gliclazide | |
| 45831 | Dacadis MR 30mg tablets (Mylan) | | Gliclazide | |
| 21892 | Diaglyk 80mg tablets (Ashbourne Pharmaceuticals Ltd) | | Gliclazide | |
| 11695 | Diamicron 30mg MR tablets (Servier Laboratories Ltd) | | Gliclazide | |
| 1964 | Diamicron 80mg tablets (Servier Laboratories Ltd) | | Gliclazide | |
| 33562 | Duclazide 80mg Tablet (Dumex Ltd) | | Gliclazide | |
| 44473 | Edicil MR 30mg tablets (Teva UK Ltd) | | Gliclazide | |
| 58882 | Gliclazide 120mg/5ml oral suspension | | Gliclazide | |
| 69669 | Gliclazide 160mg/5ml oral suspension | | Gliclazide | |
| 5627 | Gliclazide 30mg modified-release tablets | | Gliclazide | |
| 53288 | Gliclazide 30mg modified-release tablets (A A H Pharmaceuticals Ltd) | | Gliclazide | |
| 57830 | Gliclazide 30mg modified-release tablets (Alliance Healthcare (Distribution) Ltd) | | Gliclazide | |
| 68415 | Gliclazide 30mg modified-release tablets (Phoenix Healthcare Distribution Ltd) | | Gliclazide | |
| 43065 | Gliclazide 40mg tablets | | Gliclazide | |
| 61957 | Gliclazide 40mg tablets (A A H Pharmaceuticals Ltd) | | Gliclazide | |
| 71781 | Gliclazide 40mg tablets (Teva UK Ltd) | | Gliclazide | |
| 15374 | Gliclazide 40mg/5ml oral suspension | | Gliclazide | |
| 56437 | Gliclazide 60mg modified-release tablets | | Gliclazide | |
| 42790 | Gliclazide 80mg Tablet (Merck Generics (UK) Ltd) | | Gliclazide | |
| 45215 | Gliclazide 80mg Tablet (Neo Laboratories Ltd) | | Gliclazide | |
| 32 | Gliclazide 80mg tablets | | Gliclazide | |
| 17343 | Gliclazide 80mg tablets (A A H Pharmaceuticals Ltd) | | Gliclazide | |
| 51955 | Gliclazide 80mg tablets (Accord Healthcare Ltd) | | Gliclazide | |
| 31212 | Gliclazide 80mg tablets (Actavis UK Ltd) | | Gliclazide | |
| 63048 | Gliclazide 80mg tablets (Alliance Healthcare (Distribution) Ltd) | | Gliclazide | |
| 56008 | Gliclazide 80mg tablets (Almus Pharmaceuticals Ltd) | | Gliclazide | |
| 54764 | Gliclazide 80mg tablets (Arrow Generics Ltd) | | Gliclazide | |
| 68819 | Gliclazide 80mg tablets (Bristol Laboratories Ltd) | | Gliclazide | |
| 34932 | Gliclazide 80mg tablets (Genus Pharmaceuticals Ltd) | | Gliclazide | |
| 34399 | Gliclazide 80mg tablets (IVAX Pharmaceuticals UK Ltd) | | Gliclazide | |
| 67781 | Gliclazide 80mg tablets (Milpharm Ltd) | | Gliclazide | |
| 29939 | Gliclazide 80mg tablets (Mylan) | | Gliclazide | |
| 36856 | Gliclazide 80mg tablets (Sandoz Ltd) | | Gliclazide | |
| 48056 | Gliclazide 80mg tablets (Sovereign Medical Ltd) | | Gliclazide | |
| 60495 | Gliclazide 80mg tablets (Teva UK Ltd) | | Gliclazide | |
| 21564 | Gliclazide 80mg tablets (Wockhardt UK Ltd) | | Gliclazide | |
| 47074 | Gliclazide 80mg/5ml oral suspension | | Gliclazide | |
| 55862 | Gliclazide Oral solution | | Gliclazide | |
| 62034 | Laaglyda MR 60mg tablets (Consilient Health Ltd) | | Gliclazide | |
| 47894 | Nazdol MR 30mg tablets (Consilient Health Ltd) | | Gliclazide | |
| 40425 | Nazdol MR 30mg tablets (Teva UK Ltd) | | Gliclazide | |
| 63131 | Ziclaseg 30mg modified-release tablets (Lupin (Europe) Ltd) | | Gliclazide | |
| 43465 | Zicron 40mg tablets (Bristol Laboratories Ltd) | | Gliclazide | |
| 67056 | Amaryl 1mg tablets (Lexon (UK) Ltd) | | Glimepiride | |
| 7332 | Amaryl 1mg tablets (Zentiva) | | Glimepiride | |
| 7284 | Amaryl 2mg tablets (Zentiva) | | Glimepiride | |
| 7409 | Amaryl 3mg tablets (Zentiva) | | Glimepiride | |
| 11284 | Amaryl 4mg tablets (Zentiva) | | Glimepiride | |
| 5276 | Glimepiride 1mg tablets | | Glimepiride | |
| 40365 | Glimepiride 1mg tablets (Actavis UK Ltd) | | Glimepiride | |
| 71477 | Glimepiride 1mg tablets (Alliance Healthcare (Distribution) Ltd) | | Glimepiride | |
| 71667 | Glimepiride 1mg tablets (Teva UK Ltd) | | Glimepiride | |
| 5353 | Glimepiride 2mg tablets | | Glimepiride | |
| 66399 | Glimepiride 2mg tablets (A A H Pharmaceuticals Ltd) | | Glimepiride | |
| 62014 | Glimepiride 2mg tablets (Accord Healthcare Ltd) | | Glimepiride | |
| 71476 | Glimepiride 2mg tablets (Alliance Healthcare (Distribution) Ltd) | | Glimepiride | |
| 6337 | Glimepiride 3mg tablets | | Glimepiride | |
| 71665 | Glimepiride 3mg tablets (Alliance Healthcare (Distribution) Ltd) | | Glimepiride | |
| 5316 | Glimepiride 4mg tablets | | Glimepiride | |
| 61311 | Glimepiride 4mg tablets (Sigma Pharmaceuticals Plc) | | Glimepiride | |
| 68675 | Glimepiride 4mg tablets (Somex Pharma) | | Glimepiride | |
| 68289 | Glimepiride 4mg tablets (Waymade Healthcare Plc) | | Glimepiride | |
| 44738 | Niddaryl 1mg tablets (Dee Pharmaceuticals Ltd) | | Glimepiride | |
| 12513 | Glibenese 5mg tablets (Pfizer Ltd) | | Glipizide | |
| 5636 | Glipizide 5mg tablets | | Glipizide | |
| 34802 | Glipizide 5mg tablets (IVAX Pharmaceuticals UK Ltd) | | Glipizide | |
| 29326 | Glipizide 5mg tablets (Mylan) | | Glipizide | |
| 17698 | Minodiab 5mg tablets (Pfizer Ltd) | | Glipizide | |
| 547 | Glipizide 2.5mg tablets | | Glipizide | |
| 17706 | Minodiab 2.5mg tablets (Pfizer Ltd) | | Glipizide | |
| 8390 | Gliquidone 30mg tablets | | Gliquidone | |
| 19658 | Glurenorm 30mg tablets (Sanofi) | | Gliquidone | |
| 44304 | Glyconon 500mg Tablet (DDSA Pharmaceuticals Ltd) | | Tolbutamide | |
| 12455 | Rastinon 500mg Tablet (Hoechst Marion Roussel) | | Tolbutamide | |
| 1965 | Tolbutamide 500mg tablets | | Tolbutamide | |
| 34957 | Tolbutamide 500mg tablets (A A H Pharmaceuticals Ltd) | | Tolbutamide | |
| 33673 | Tolbutamide 500mg tablets (Actavis UK Ltd) | | Tolbutamide | |
| 46927 | Tolbutamide 500mg tablets (Teva UK Ltd) | | Tolbutamide | |
| 11946 | Tolbutamide 50mg/ml Injection | | Tolbutamide | |
| 39149 | Galvus 50mg tablets (Novartis Pharmaceuticals UK Ltd) | | Vildagliptin | |
| 37875 | Vildagliptin 50mg tablets | | Vildagliptin | |
| **Statin therapies** | | | | |
| **Product Code** | **Product Name** | | **Drug substance name** | |
| 25 | Simvastatin 20mg tablets | | Simvastatin | |
| 28 | Atorvastatin 10mg tablets | | Atorvastatin calcium trihydrate | |
| 42 | Simvastatin 10mg tablets | | Simvastatin | |
| 51 | Simvastatin 40mg tablets | | Simvastatin | |
| 75 | Atorvastatin 20mg tablets | | Atorvastatin calcium trihydrate | |
| 379 | Fluvastatin 20mg capsules | | Fluvastatin sodium | |
| 490 | Pravastatin 10mg tablets | | Pravastatin sodium | |
| 713 | Rosuvastatin 10mg tablets | | Rosuvastatin calcium | |
| 730 | Pravastatin 20mg tablets | | Pravastatin sodium | |
| 745 | Atorvastatin 40mg tablets | | Atorvastatin calcium trihydrate | |
| 802 | Simvador 40mg tablets (Discovery Pharmaceuticals) | | Simvastatin | |
| 818 | Simvastatin 20mg/5ml oral solution sugar free | | Simvastatin | |
| 1219 | Pravastatin 40mg tablets | | Pravastatin sodium | |
| 1221 | Lipostat 10mg tablets (Bristol-Myers Squibb Pharmaceuticals Ltd) | | Pravastatin sodium | |
| 1223 | Lipostat 40mg tablets (Bristol-Myers Squibb Pharmaceuticals Ltd) | | Pravastatin sodium | |
| 2137 | Fluvastatin 40mg capsules | | Fluvastatin sodium | |
| 2718 | Zocor 10mg tablets (Merck Sharp & Dohme Ltd) | | Simvastatin | |
| 2955 | Lipitor 40mg tablets (Pfizer Ltd) | | Atorvastatin calcium trihydrate | |
| 3411 | Lipitor 10mg tablets (Pfizer Ltd) | | Atorvastatin calcium trihydrate | |
| 3690 | Lipostat 20mg tablets (Bristol-Myers Squibb Pharmaceuticals Ltd) | | Pravastatin sodium | |
| 4961 | Lipobay 300microgram Tablet (Bayer Plc) | | Cerivastatin sodium | |
| 5148 | Simvastatin 80mg tablets | | Simvastatin | |
| 5775 | Atorvastatin 80mg tablets | | Atorvastatin calcium trihydrate | |
| 5985 | Lescol XL 80mg tablets (Novartis Pharmaceuticals UK Ltd) | | Fluvastatin sodium | |
| 6168 | Zocor 40mg tablets (Merck Sharp & Dohme Ltd) | | Simvastatin | |
| 6213 | Rosuvastatin 20mg tablets | | Rosuvastatin calcium | |
| 7196 | Zocor 20mg tablets (Merck Sharp & Dohme Ltd) | | Simvastatin | |
| 7347 | Crestor 10mg tablets (AstraZeneca UK Ltd) | | Rosuvastatin calcium | |
| 7374 | Lipitor 20mg tablets (Pfizer Ltd) | | Atorvastatin calcium trihydrate | |
| 7552 | Simvastatin 20mg / Ezetimibe 10mg tablets | | Ezetimibe/Simvastatin | |
| 7554 | Rosuvastatin 5mg tablets | | Rosuvastatin calcium | |
| 8380 | Lescol 20mg capsules (Novartis Pharmaceuticals UK Ltd) | | Fluvastatin sodium | |
| 9153 | Lescol 40mg capsules (Novartis Pharmaceuticals UK Ltd) | | Fluvastatin sodium | |
| 9315 | Lipobay 100microgram Tablet (Bayer Plc) | | Cerivastatin sodium | |
| 9316 | Lipobay 200microgram Tablet (Bayer Plc) | | Cerivastatin sodium | |
| 9897 | Rosuvastatin 40mg tablets | | Rosuvastatin calcium | |
| 9920 | Simvador 20mg tablets (Discovery Pharmaceuticals) | | Simvastatin | |
| 9930 | Crestor 40mg tablets (AstraZeneca UK Ltd) | | Rosuvastatin calcium | |
| 10172 | Simvastatin 40mg / Ezetimibe 10mg tablets | | Simvastatin/Ezetimibe | |
| 10183 | Simvastatin 40mg with ezetimibe 10mg tablet | | Simvastatin/Ezetimibe | |
| 10206 | Simvastatin 80mg with ezetimibe 10mg tablet | | Simvastatin/Ezetimibe | |
| 11627 | Fluvastatin 80mg modified-release tablets | | Fluvastatin sodium | |
| 11815 | Simvastatin 20mg with ezetimibe 10mg tablet | | Simvastatin/Ezetimibe | |
| 13041 | Simvador 10mg tablets (Discovery Pharmaceuticals) | | Simvastatin | |
| 14219 | Simvastatin 80mg / Ezetimibe 10mg tablets | | Simvastatin/Ezetimibe | |
| 15252 | Crestor 20mg tablets (AstraZeneca UK Ltd) | | Rosuvastatin calcium | |
| 16186 | Inegy 10mg/80mg tablets (Merck Sharp & Dohme Ltd) | | Simvastatin/Ezetimibe | |
| 17059 | Inegy 10mg/40mg tablets (Merck Sharp & Dohme Ltd) | | Simvastatin/Ezetimibe | |
| 17683 | Lipitor 80mg tablets (Pfizer Ltd) | | Atorvastatin calcium trihydrate | |
| 17688 | Crestor 5mg tablets (AstraZeneca UK Ltd) | | Rosuvastatin calcium | |
| 18442 | Lipobay 400microgram Tablet (Bayer Plc) | | Cerivastatin sodium | |
| 21020 | Inegy 10mg/20mg tablets (Merck Sharp & Dohme Ltd) | | Ezetimibe/Simvastatin | |
| 22579 | Zocor 80mg tablets (Merck Sharp & Dohme Ltd) | | Simvastatin | |
| 31930 | Zocor heart-pro 10mg Tablet (McNeil Products Ltd) | | Simvastatin | |
| 32909 | Simvastatin 80mg tablets (A A H Pharmaceuticals Ltd) | | Simvastatin | |
| 32921 | Pravastatin 10mg Tablet (Dr Reddy's Laboratories (UK) Ltd) | | Pravastatin sodium | |
| 33082 | Simvastatin 20mg tablets (A A H Pharmaceuticals Ltd) | | Simvastatin | |
| 34312 | Simvastatin 20mg tablets (Mylan) | | Simvastatin | |
| 34316 | Simvastatin 20mg tablets (Teva UK Ltd) | | Simvastatin | |
| 34353 | Simvastatin 40mg tablets (Mylan) | | Simvastatin | |
| 34366 | Simvastatin 20mg tablets (IVAX Pharmaceuticals UK Ltd) | | Simvastatin | |
| 34376 | Simvastatin 40mg tablets (Teva UK Ltd) | | Simvastatin | |
| 34381 | Simvastatin 40mg tablets (IVAX Pharmaceuticals UK Ltd) | | Simvastatin | |
| 34476 | Simvastatin 20mg Tablet (Ratiopharm UK Ltd) | | Simvastatin | |
| 34481 | Simvastatin 10mg tablets (IVAX Pharmaceuticals UK Ltd) | | Simvastatin | |
| 34502 | Simvastatin 40mg tablets (A A H Pharmaceuticals Ltd) | | Simvastatin | |
| 34535 | Simvastatin 10mg tablets (Mylan) | | Simvastatin | |
| 34545 | Simvastatin 40mg Tablet (Ratiopharm UK Ltd) | | Simvastatin | |
| 34560 | Simvastatin 10mg Tablet (Ratiopharm UK Ltd) | | Simvastatin | |
| 34746 | Simvastatin 20mg Tablet (Niche Generics Ltd) | | Simvastatin | |
| 34814 | Simvastatin 20mg tablets (Wockhardt UK Ltd) | | Simvastatin | |
| 34820 | Pravastatin 40mg tablets (A A H Pharmaceuticals Ltd) | | Pravastatin sodium | |
| 34879 | Simvastatin 40mg Tablet (Niche Generics Ltd) | | Simvastatin | |
| 34891 | Simvastatin 20mg tablets (Kent Pharmaceuticals Ltd) | | Simvastatin | |
| 34907 | Simvastatin 40mg tablets (Wockhardt UK Ltd) | | Simvastatin | |
| 34955 | Simvastatin 10mg tablets (A A H Pharmaceuticals Ltd) | | Simvastatin | |
| 34969 | Simvastatin 40mg tablets (Actavis UK Ltd) | | Simvastatin | |
| 36377 | Pravastatin 20mg tablets (Teva UK Ltd) | | Pravastatin sodium | |
| 37434 | Simvastatin 40mg tablets (Sandoz Ltd) | | Simvastatin | |
| 39060 | Simvastatin 20mg tablets (Dexcel-Pharma Ltd) | | Simvastatin | |
| 39652 | Simvastatin 40mg/5ml oral solution sugar free | | Simvastatin | |
| 39675 | Simvastatin 20mg/5ml Oral suspension (Martindale Pharmaceuticals Ltd) | | Simvastatin | |
| 39870 | Simvador 80mg tablets (Discovery Pharmaceuticals) | | Simvastatin | |
| 40340 | Simvastatin 10mg tablets (Teva UK Ltd) | | Simvastatin | |
| 40382 | Pravastatin 20mg tablets (A A H Pharmaceuticals Ltd) | | Pravastatin sodium | |
| 40601 | Simvastatin 20mg tablets (Ranbaxy (UK) Ltd) | | Simvastatin | |
| 41657 | Simvastatin 80mg tablets (Teva UK Ltd) | | Simvastatin | |
| 43218 | Pravastatin 10mg tablets (Teva UK Ltd) | | Pravastatin sodium | |
| 44528 | Simvastatin 20mg/5ml oral suspension sugar free (Rosemont Pharmaceuticals Ltd) | | Simvastatin | |
| 44650 | Simvastatin 40mg tablets (Dexcel-Pharma Ltd) | | Simvastatin | |
| 44878 | Ranzolont 10mg tablets (Ranbaxy (UK) Ltd) | | Simvastatin | |
| 45219 | Simvastatin 40mg tablets (Kent Pharmaceuticals Ltd) | | Simvastatin | |
| 45235 | Simvastatin 20mg tablets (Sandoz Ltd) | | Simvastatin | |
| 45245 | Simvastatin 20mg tablets (Actavis UK Ltd) | | Simvastatin | |
| 45346 | Simvastatin 40mg tablets (Arrow Generics Ltd) | | Simvastatin | |
| 46878 | Simvastatin 40mg tablets (Almus Pharmaceuticals Ltd) | | Simvastatin | |
| 46956 | Simvastatin 80mg tablets (Arrow Generics Ltd) | | Simvastatin | |
| 47721 | Lipitor 10mg chewable tablets (Pfizer Ltd) | | Atorvastatin calcium trihydrate | |
| 47774 | Simvastatin 10mg tablets (Arrow Generics Ltd) | | Simvastatin | |
| 47948 | Simvastatin 10mg tablets (Tillomed Laboratories Ltd) | | Simvastatin | |
| 47988 | Pravastatin 40mg tablets (Mylan) | | Pravastatin sodium | |
| 48018 | Simvastatin 20mg tablets (Arrow Generics Ltd) | | Simvastatin | |
| 48051 | Simvastatin 10mg tablets (Kent Pharmaceuticals Ltd) | | Simvastatin | |
| 48058 | Simvastatin 10mg tablets (Ranbaxy (UK) Ltd) | | Simvastatin | |
| 48078 | Simvastatin 10mg tablets (Actavis UK Ltd) | | Simvastatin | |
| 48097 | Pravastatin 40mg tablets (Teva UK Ltd) | | Pravastatin sodium | |
| 48221 | Simvastatin 20mg/5ml oral suspension sugar free | | Simvastatin | |
| 48346 | Atorvastatin 60mg tablets | | Atorvastatin calcium trihydrate | |
| 48431 | Simvastatin 40mg/5ml oral suspension sugar free | | Simvastatin | |
| 48518 | Atorvastatin 10mg/5ml oral solution | | Atorvastatin calcium trihydrate | |
| 48867 | Simvastatin 40mg tablets (Alliance Healthcare (Distribution) Ltd) | | Simvastatin | |
| 48973 | Atorvastatin 30mg tablets | | Atorvastatin calcium trihydrate | |
| 49061 | Simvastatin 40mg tablets (Bristol Laboratories Ltd) | | Simvastatin | |
| 49062 | Simvastatin 20mg tablets (Alliance Healthcare (Distribution) Ltd) | | Simvastatin | |
| 49558 | Atorvastatin 20mg tablets (A A H Pharmaceuticals Ltd) | | Atorvastatin calcium trihydrate | |
| 49587 | Simvastatin 80mg tablets (Almus Pharmaceuticals Ltd) | | Simvastatin | |
| 49751 | Atorvastatin 40mg tablets (Alliance Healthcare (Distribution) Ltd) | | Atorvastatin calcium trihydrate | |
| 50236 | Atorvastatin 10mg tablets (Zentiva) | | Atorvastatin calcium trihydrate | |
| 50272 | Atorvastatin 40mg tablets (Pfizer Ltd) | | Atorvastatin calcium trihydrate | |
| 50483 | Simvastatin 40mg tablets (Relonchem Ltd) | | Simvastatin | |
| 50564 | Simvastatin 20mg tablets (Relonchem Ltd) | | Simvastatin | |
| 50670 | Simvastatin 40mg tablets (Aurobindo Pharma Ltd) | | Simvastatin | |
| 50703 | Simvastatin 40mg tablets (Accord Healthcare Ltd) | | Simvastatin | |
| 50754 | Simvastatin 20mg tablets (Medreich Plc) | | Simvastatin | |
| 50788 | Atorvastatin 20mg tablets (Pfizer Ltd) | | Atorvastatin calcium trihydrate | |
| 50790 | Atorvastatin 20mg tablets (Dexcel-Pharma Ltd) | | Atorvastatin calcium trihydrate | |
| 50882 | Simvastatin 40mg tablets (Somex Pharma) | | Simvastatin | |
| 50925 | Pravastatin 10mg tablets (Sigma Pharmaceuticals Plc) | | Pravastatin sodium | |
| 50963 | Atorvastatin 40mg tablets (Teva UK Ltd) | | Atorvastatin calcium trihydrate | |
| 51085 | Simvastatin 10mg tablets (Medreich Plc) | | Simvastatin | |
| 51134 | Atorvastatin 10mg tablets (A A H Pharmaceuticals Ltd) | | Atorvastatin calcium trihydrate | |
| 51166 | Simvastatin 40mg tablets (Medreich Plc) | | Simvastatin | |
| 51200 | Atorvastatin 40mg tablets (Arrow Generics Ltd) | | Atorvastatin calcium trihydrate | |
| 51233 | Simvastatin 10mg tablets (Alliance Healthcare (Distribution) Ltd) | | Simvastatin | |
| 51359 | Atorvastatin 20mg tablets (Arrow Generics Ltd) | | Atorvastatin calcium trihydrate | |
| 51483 | Simvastatin 20mg tablets (Aurobindo Pharma Ltd) | | Simvastatin | |
| 51622 | Atorvastatin 20mg tablets (Consilient Health Ltd) | | Atorvastatin calcium trihydrate | |
| 51676 | Pravastatin 40mg tablets (Medreich Plc) | | Pravastatin sodium | |
| 51715 | Simvastatin 10mg tablets (Sigma Pharmaceuticals Plc) | | Simvastatin | |
| 51876 | Atorvastatin 40mg tablets (Consilient Health Ltd) | | Atorvastatin calcium trihydrate | |
| 51890 | Pravastatin 20mg tablets (Medreich Plc) | | Pravastatin sodium | |
| 52097 | Atorvastatin 40mg tablets (Wockhardt UK Ltd) | | Atorvastatin calcium trihydrate | |
| 52098 | Simvastatin 40mg tablets (Ranbaxy (UK) Ltd) | | Simvastatin | |
| 52168 | Atorvastatin 20mg tablets (Aspire Pharma Ltd) | | Atorvastatin calcium trihydrate | |
| 52211 | Atorvastatin 20mg tablets (Actavis UK Ltd) | | Atorvastatin calcium trihydrate | |
| 52257 | Simvastatin 20mg tablets (Accord Healthcare Ltd) | | Simvastatin | |
| 52397 | Atorvastatin 40mg tablets (Dr Reddy's Laboratories (UK) Ltd) | | Atorvastatin calcium trihydrate | |
| 52398 | Atorvastatin 40mg tablets (A A H Pharmaceuticals Ltd) | | Atorvastatin calcium trihydrate | |
| 52459 | Atorvastatin 80mg tablets (Actavis UK Ltd) | | Atorvastatin calcium trihydrate | |
| 52460 | Atorvastatin 40mg tablets (Aspire Pharma Ltd) | | Atorvastatin calcium trihydrate | |
| 52625 | Simvastatin 10mg tablets (Wockhardt UK Ltd) | | Simvastatin | |
| 52676 | Simvastatin 10mg/5ml oral suspension | | Simvastatin | |
| 52755 | Pravastatin 20mg tablets (Alliance Healthcare (Distribution) Ltd) | | Pravastatin sodium | |
| 52812 | Simvastatin 20mg tablets (Sigma Pharmaceuticals Plc) | | Simvastatin | |
| 52821 | Atorvastatin 80mg tablets (Dr Reddy's Laboratories (UK) Ltd) | | Atorvastatin calcium trihydrate | |
| 52953 | Simvastatin 20mg tablets (Bristol Laboratories Ltd) | | Simvastatin | |
| 52962 | Simvastatin 80mg tablets (Medreich Plc) | | Simvastatin | |
| 53087 | Simvastatin 20mg tablets (Somex Pharma) | | Simvastatin | |
| 53340 | Zocor 40mg tablets (Lexon (UK) Ltd) | | Simvastatin | |
| 53415 | Simvastatin 10mg tablets (Aurobindo Pharma Ltd) | | Simvastatin | |
| 53460 | Crestor 10mg tablets (DE Pharmaceuticals) | | Rosuvastatin calcium | |
| 53594 | Lipitor 80mg tablets (Mawdsley-Brooks & Company Ltd) | | Atorvastatin calcium trihydrate | |
| 53676 | Simvastatin 20mg tablets (Tillomed Laboratories Ltd) | | Simvastatin | |
| 53770 | Fluvastatin 40mg capsules (A A H Pharmaceuticals Ltd) | | Fluvastatin sodium | |
| 53772 | Atorvastatin 80mg tablets (Alliance Healthcare (Distribution) Ltd) | | Atorvastatin calcium trihydrate | |
| 53822 | Simvastatin 10mg tablets (Bristol Laboratories Ltd) | | Simvastatin | |
| 53887 | Atorvastatin 40mg tablets (Actavis UK Ltd) | | Atorvastatin calcium trihydrate | |
| 53890 | Atorvastatin 80mg tablets (Pfizer Ltd) | | Atorvastatin calcium trihydrate | |
| 53908 | Simvastatin 10mg tablets (Dexcel-Pharma Ltd) | | Simvastatin | |
| 53966 | Simvastatin 40mg tablets (Phoenix Healthcare Distribution Ltd) | | Simvastatin | |
| 54240 | Simvastatin 40mg tablets (Sigma Pharmaceuticals Plc) | | Simvastatin | |
| 54266 | Simvastatin 20mg/5ml oral suspension | | Simvastatin | |
| 54435 | Pravastatin 40mg tablets (Almus Pharmaceuticals Ltd) | | Pravastatin sodium | |
| 54493 | Simvastatin 10mg tablets (Relonchem Ltd) | | Simvastatin | |
| 54535 | Atorvastatin 10mg tablets (Pfizer Ltd) | | Atorvastatin calcium trihydrate | |
| 54606 | Simvastatin 20mg/5ml oral suspension sugar free (A A H Pharmaceuticals Ltd) | | Simvastatin | |
| 54607 | Pravastatin 20mg tablets (Almus Pharmaceuticals Ltd) | | Pravastatin sodium | |
| 54655 | Simvastatin 10mg tablets (Accord Healthcare Ltd) | | Simvastatin | |
| 54819 | Simvastatin 40mg/5ml oral suspension sugar free (Rosemont Pharmaceuticals Ltd) | | Simvastatin | |
| 54947 | Simvastatin 20mg tablets (Almus Pharmaceuticals Ltd) | | Simvastatin | |
| 54976 | Simvastatin 10mg tablets (Somex Pharma) | | Simvastatin | |
| 54985 | Simvastatin 40mg/5ml oral suspension | | Simvastatin | |
| 54992 | Atorvastatin 10mg/5ml oral suspension | | Atorvastatin calcium trihydrate | |
| 55032 | Atorvastatin 10mg tablets (Dexcel-Pharma Ltd) | | Atorvastatin calcium trihydrate | |
| 55034 | Atorvastatin 40mg/5ml oral suspension | | Atorvastatin calcium trihydrate | |
| 55444 | Atorvastatin 40mg tablets (Zentiva) | | Atorvastatin calcium trihydrate | |
| 55452 | Simvastatin 20mg tablets (Phoenix Healthcare Distribution Ltd) | | Simvastatin | |
| 55727 | Atorvastatin 10mg tablets (Actavis UK Ltd) | | Atorvastatin calcium trihydrate | |
| 55912 | Pravastatin 40mg tablets (Alliance Healthcare (Distribution) Ltd) | | Pravastatin sodium | |
| 56016 | Lipitor 20mg chewable tablets (Pfizer Ltd) | | Atorvastatin calcium trihydrate | |
| 56065 | Simvastatin 20mg/5ml oral suspension sugar free (Waymade Healthcare Plc) | | Simvastatin | |
| 56097 | Atorvastatin 10mg chewable tablets sugar free | | Atorvastatin calcium trihydrate | |
| 56146 | Pravastatin 10mg tablets (Waymade Healthcare Plc) | | Pravastatin sodium | |
| 56165 | Atorvastatin 20mg chewable tablets sugar free | | Atorvastatin calcium trihydrate | |
| 56182 | Atorvastatin 80mg tablets (Zentiva) | | Atorvastatin calcium trihydrate | |
| 56248 | Atorvastatin 20mg tablets (Sigma Pharmaceuticals Plc) | | Atorvastatin calcium trihydrate | |
| 56481 | Zocor 10mg tablets (Sigma Pharmaceuticals Plc) | | Simvastatin | |
| 56494 | Zocor 20mg tablets (Sigma Pharmaceuticals Plc) | | Simvastatin | |
| 56564 | Atorvastatin 20mg tablets (Almus Pharmaceuticals Ltd) | | Atorvastatin calcium trihydrate | |
| 56607 | Pravastatin 20mg tablets (Waymade Healthcare Plc) | | Pravastatin sodium | |
| 56735 | Pravastatin 20mg tablets (Mylan) | | Pravastatin sodium | |
| 56841 | Atorvastatin 40mg tablets (Dexcel-Pharma Ltd) | | Atorvastatin calcium trihydrate | |
| 56893 | Pravastatin 40mg tablets (Accord Healthcare Ltd) | | Pravastatin sodium | |
| 56916 | Pravastatin 40mg tablets (PLIVA Pharma Ltd) | | Pravastatin sodium | |
| 57108 | Pravastatin 40mg tablets (Waymade Healthcare Plc) | | Pravastatin sodium | |
| 57117 | Atorvastatin 80mg tablets (Waymade Healthcare Plc) | | Atorvastatin calcium trihydrate | |
| 57137 | Pravastatin 10mg tablets (Almus Pharmaceuticals Ltd) | | Pravastatin sodium | |
| 57296 | Pravastatin 20mg tablets (Phoenix Healthcare Distribution Ltd) | | Pravastatin sodium | |
| 57329 | Simvastatin 25mg/5ml oral suspension | | Simvastatin | |
| 57348 | Atorvastatin 10mg tablets (Consilient Health Ltd) | | Atorvastatin calcium trihydrate | |
| 57397 | Pravastatin 10mg tablets (Accord Healthcare Ltd) | | Pravastatin sodium | |
| 57568 | Zocor 10mg tablets (Lexon (UK) Ltd) | | Simvastatin | |
| 57763 | Rosuvastatin 10mg tablets (Waymade Healthcare Plc) | | Rosuvastatin calcium | |
| 57834 | Atorvastatin 40mg tablets (DE Pharmaceuticals) | | Atorvastatin calcium trihydrate | |
| 57836 | Atorvastatin 80mg tablets (Teva UK Ltd) | | Atorvastatin calcium trihydrate | |
| 57999 | Crestor 40mg tablets (Lexon (UK) Ltd) | | Rosuvastatin calcium | |
| 58041 | Atorvastatin 20mg tablets (Teva UK Ltd) | | Atorvastatin calcium trihydrate | |
| 58110 | Atorvastatin 20mg tablets (Zentiva) | | Atorvastatin calcium trihydrate | |
| 58315 | Simvastatin 20mg tablets (Waymade Healthcare Plc) | | Simvastatin | |
| 58394 | Atorvastatin 20mg tablets (Alliance Healthcare (Distribution) Ltd) | | Atorvastatin calcium trihydrate | |
| 58418 | Atorvastatin 80mg tablets (A A H Pharmaceuticals Ltd) | | Atorvastatin calcium trihydrate | |
| 58617 | Rosuvastatin 20mg/5ml oral suspension | | Rosuvastatin calcium | |
| 58742 | Atorvastatin 80mg tablets (Arrow Generics Ltd) | | Atorvastatin calcium trihydrate | |
| 58755 | Simvastatin 10mg tablets (Phoenix Healthcare Distribution Ltd) | | Simvastatin | |
| 58834 | Atorvastatin 10mg tablets (DE Pharmaceuticals) | | Atorvastatin calcium trihydrate | |
| 58868 | Atorvastatin 10mg tablets (Sigma Pharmaceuticals Plc) | | Atorvastatin calcium trihydrate | |
| 59272 | Atorvastatin 20mg tablets (Waymade Healthcare Plc) | | Atorvastatin calcium trihydrate | |
| 59278 | Fluvastatin 20mg capsules (Zentiva) | | Fluvastatin sodium | |
| 59331 | Lipitor 10mg tablets (DE Pharmaceuticals) | | Atorvastatin calcium trihydrate | |
| 59357 | Atorvastatin 10mg tablets (Ranbaxy (UK) Ltd) | | Atorvastatin calcium trihydrate | |
| 59446 | Atorvastatin 40mg tablets (Almus Pharmaceuticals Ltd) | | Atorvastatin calcium trihydrate | |
| 59447 | Crestor 20mg tablets (Waymade Healthcare Plc) | | Rosuvastatin calcium | |
| 59452 | Rosuvastatin 5mg tablets (Waymade Healthcare Plc) | | Rosuvastatin calcium | |
| 59508 | Pravastatin 20mg tablets (Accord Healthcare Ltd) | | Pravastatin sodium | |
| 59776 | Atorvastatin 80mg tablets (Aspire Pharma Ltd) | | Atorvastatin calcium trihydrate | |
| 59859 | Atorvastatin 10mg tablets (Teva UK Ltd) | | Atorvastatin calcium trihydrate | |
| 60160 | Rosuvastatin 5mg tablets (Mawdsley-Brooks & Company Ltd) | | Rosuvastatin calcium | |
| 60251 | Pravastatin 10mg tablets (Sandoz Ltd) | | Pravastatin sodium | |
| 60464 | Atorvastatin 20mg/5ml oral suspension | | Atorvastatin calcium trihydrate | |
| 60511 | Atorvastatin 40mg tablets (Ranbaxy (UK) Ltd) | | Atorvastatin calcium trihydrate | |
| 60607 | Atorvastatin 80mg tablets (DE Pharmaceuticals) | | Atorvastatin calcium trihydrate | |
| 60989 | Atorvastatin 80mg tablets (Phoenix Healthcare Distribution Ltd) | | Atorvastatin calcium trihydrate | |
| 61134 | Pravastatin 20mg tablets (Sigma Pharmaceuticals Plc) | | Pravastatin sodium | |
| 61149 | Atorvastatin 10mg tablets (Waymade Healthcare Plc) | | Atorvastatin calcium trihydrate | |
| 61155 | Simvastatin 40mg/5ml oral suspension sugar free (A A H Pharmaceuticals Ltd) | | Simvastatin | |
| 61321 | Simvastatin 10mg tablets (Sandoz Ltd) | | Simvastatin | |
| 61360 | Simvastatin 10mg tablets (Almus Pharmaceuticals Ltd) | | Simvastatin | |
| 61665 | Simvastatin 10mg tablets (Waymade Healthcare Plc) | | Simvastatin | |
| 62137 | Simvastatin 40mg tablets (Waymade Healthcare Plc) | | Simvastatin | |
| 62148 | Fluvastatin 20mg capsules (Actavis UK Ltd) | | Fluvastatin sodium | |
| 62219 | Atorvastatin 20mg tablets (DE Pharmaceuticals) | | Atorvastatin calcium trihydrate | |
| 62429 | Atorvastatin 20mg tablets (DE Pharmaceuticals) | | Atorvastatin calcium trihydrate | |
| 62476 | Atorvastatin 80mg tablets (Almus Pharmaceuticals Ltd) | | Atorvastatin calcium trihydrate | |
| 62979 | Pravastatin 40mg tablets (Kent Pharmaceuticals Ltd) | | Pravastatin sodium | |
| 63074 | Pravastatin 20mg tablets (PLIVA Pharma Ltd) | | Pravastatin sodium | |
| 63140 | Atorvastatin 10mg tablets (Alliance Healthcare (Distribution) Ltd) | | Atorvastatin calcium trihydrate | |
| 63249 | Atorvastatin 80mg tablets (Consilient Health Ltd) | | Atorvastatin calcium trihydrate | |
| 63469 | Atorvastatin 30mg tablets (Consilient Health Ltd) | | Atorvastatin calcium trihydrate | |
| 63787 | Pravastatin 10mg tablets (Tillomed Laboratories Ltd) | | Pravastatin sodium | |
| 64067 | Atorvastatin 20mg/5ml oral solution | | Atorvastatin calcium trihydrate | |
| 64104 | Simvastatin 20mg tablets (Crescent Pharma Ltd) | | Simvastatin | |
| 64180 | Simvastatin 10mg tablets (Crescent Pharma Ltd) | | Simvastatin | |
| 64307 | Simvastatin 40mg tablets (Crescent Pharma Ltd) | | Simvastatin | |
| 64702 | Atorvastatin 30mg tablets (A A H Pharmaceuticals Ltd) | | Atorvastatin calcium trihydrate | |
| 64810 | Atorvastatin 40mg tablets (Phoenix Healthcare Distribution Ltd) | | Atorvastatin calcium trihydrate | |
| 64825 | Atorvastatin 10mg tablets (Phoenix Healthcare Distribution Ltd) | | Atorvastatin calcium trihydrate | |
| 64868 | Atorvastatin 40mg tablets (Sigma Pharmaceuticals Plc) | | Atorvastatin calcium trihydrate | |
| 64968 | Simvastatin 10mg tablets (DE Pharmaceuticals) | | Simvastatin | |
| 65181 | Simvastatin 40mg tablets (DE Pharmaceuticals) | | Simvastatin | |
| 65193 | Atorvastatin 20mg tablets (Ranbaxy (UK) Ltd) | | Atorvastatin calcium trihydrate | |
| 65679 | Simvastatin 20mg tablets (DE Pharmaceuticals) | | Simvastatin | |
| 65901 | Simvastatin 40mg tablets (Zentiva) | | Simvastatin | |
| 65925 | Simvastatin 20mg/5ml oral suspension sugar free (Alliance Healthcare (Distribution) Ltd) | | Simvastatin | |
| 66505 | Fenofibrate 145mg / Simvastatin 40mg tablets | | Simvastatin/Fenofibrate | |
| 66780 | Fenofibrate 145mg / Simvastatin 20mg tablets | | Fenofibrate/Simvastatin | |
| 66963 | Atorvastatin 80mg tablets (Sigma Pharmaceuticals Plc) | | Atorvastatin calcium trihydrate | |
| 67098 | Simvastatin 10mg tablets (Brown & Burk UK Ltd) | | Simvastatin | |
| 67328 | Lescol XL 80mg tablets (Mawdsley-Brooks & Company Ltd) | | Fluvastatin sodium | |
| 67402 | Atorvastatin 40mg tablets (Kent Pharmaceuticals Ltd) | | Atorvastatin calcium trihydrate | |
| 67573 | Atorvastatin 10mg tablets (DE Pharmaceuticals) | | Atorvastatin calcium trihydrate | |
| 67660 | Atorvastatin 80mg tablets (Ranbaxy (UK) Ltd) | | Atorvastatin calcium trihydrate | |
| 67745 | Simvastatin 10mg tablets (Zentiva) | | Simvastatin | |
| 67773 | Simvastatin 20mg tablets (Zentiva) | | Simvastatin | |
| 67829 | Pravastatin 20mg tablets (Sandoz Ltd) | | Pravastatin sodium | |
| 67846 | Atorvastatin 10mg tablets (Almus Pharmaceuticals Ltd) | | Atorvastatin calcium trihydrate | |
| 68023 | Atorvastatin 10mg tablets (Aspire Pharma Ltd) | | Atorvastatin calcium trihydrate | |
| 68048 | Atorvastatin 20mg tablets (Phoenix Healthcare Distribution Ltd) | | Atorvastatin calcium trihydrate | |
| 68156 | Pravastatin 10mg tablets (A A H Pharmaceuticals Ltd) | | Pravastatin sodium | |
| 68467 | Atorvastatin 20mg tablets (Kent Pharmaceuticals Ltd) | | Atorvastatin calcium trihydrate | |
| 68563 | Simvastatin 40mg tablets (Brown & Burk UK Ltd) | | Simvastatin | |
| 68686 | Simvastatin 20mg tablets (Genesis Pharmaceuticals Ltd) | | Simvastatin | |
| 68785 | Atorvastatin 10mg tablets (Mylan) | | Atorvastatin calcium trihydrate | |
| 68827 | Atorvastatin 20mg tablets (Mylan) | | Atorvastatin calcium trihydrate | |
| 69093 | Atorvastatin 80mg tablets (Wockhardt UK Ltd) | | Atorvastatin calcium trihydrate | |
| 69413 | Simvastatin 20mg tablets (Brown & Burk UK Ltd) | | Simvastatin | |
| 69427 | Atorvastatin 40mg tablets (Mylan) | | Atorvastatin calcium trihydrate | |
| 69528 | Cholib 145mg/20mg tablets (Mylan) | | Fenofibrate/Simvastatin | |
| 70308 | Crestor 20mg tablets (Sigma Pharmaceuticals Plc) | | Rosuvastatin calcium | |
| 70486 | Cholib 145mg/40mg tablets (Mylan) | | Simvastatin/Fenofibrate | |
| 70693 | Atorvastatin 10mg tablets (Sigma Pharmaceuticals Plc) | | Atorvastatin calcium trihydrate | |
| 70987 | Atorvastatin 10mg tablets (Dr Reddy's Laboratories (UK) Ltd) | | Atorvastatin calcium trihydrate | |
| 71014 | Rosuvastatin 20mg tablets (Waymade Healthcare Plc) | | Rosuvastatin calcium | |
| 71015 | Pravastatin 10mg tablets (Medreich Plc) | | Pravastatin sodium | |
| 71017 | Atorvastatin 20mg tablets (Dr Reddy's Laboratories (UK) Ltd) | | Atorvastatin calcium trihydrate | |
| 71029 | Fluvastatin 40mg capsules (Sandoz Ltd) | | Fluvastatin sodium | |
| 71773 | Simvastatin 80mg tablets (Brown & Burk UK Ltd) | | Simvastatin | |
| 72048 | Pravastatin 40mg tablets (Actavis UK Ltd) | | Pravastatin sodium | |
| 72050 | Simvastatin 10mg tablets (Genesis Pharmaceuticals Ltd) | | Simvastatin | |
