# Supplementary material for: Safety of antidepressants in a primary care cohort of adults with obesity and depression
Source: PLoS One. 2021 Jan 29;16(1):e0245722. doi: 10.1371/journal.pone.0245722 (PMC7846000; doi:10.1371/journal.pone.0245722)
Supplement: Attachment — Submitted filename: Response to Reviewers.docx [file pone.0245722.s012.docx]

**RESPONSE TO REVIEWER 1**

*The risks presented in this study are not contextualized to suggest if this population is at an elevated risk of mortality when using these medications, as is suggested in the conclusion. The empirical risk in this population is not incorrect, however, interpreting these results as a comparison relative to other populations is complicated by the lack of a direct comparison group.*

We thank the reviewer for this comment. We have now added in a statement and corresponding references to the Introduction (first paragraph, line 50) to highlight that there is a dose-response relationship between BMI and depression, and also between BMI and mortality. We hope that this contextualises more fully that depression is a particular challenge in this population and that treatment approaches need to consider potential risks associated with depression and obesity.

In terms of comparisons with the general population, we agree that it is a limitation that we were unable to make comparisons with the general population in the ‘normal’ (and underweight) BMI category. We have now highlighted this in the Discussion (please see limitations section, lines 390-391).

*- Keeping their population the same, the authors could redesign the study to understand the association between antidepressant use and the outcomes incorporating obesity as a dose-response of BMI. This would allow them to directly evaluate the interaction between antidepressant use and obesity on mortality.*

We did not find an interaction between antidepressant use and overweight/obese/severely obese for any of the outcomes under investigation (p>0.01 for all) – see results line 312. We can confirm that we adjusted for the relationship between BMI (categorised as overweight, obese and severely obese) in this analysis. We have now made this clearer (see covariates sub-heading / Methods, line 152).

*Second, Model 1 (all-cause mortality) and Model 4 (suicidality) are subject to confounding by indication bias. Suicidal ideation is an indication for antidepressant use. The authors are not able to determine, with this study design, whether the risk of these outcomes affects the prescribing of antidepressants or if they are actually a consequence of antidepressant use. This leads to biased results, complicating their interpretation, and the authors mention this but are unable to estimate the magnitude of the possible error. Furthermore, if the participants in this study are at a very high risk of suicide, antidepressants could possibly lower their risk, so the residual risk remains high, but is not in fact due to their use of antidepressants, rather was underlying and a biased estimate was made apparent then interpreted as an outcome of antidepressant use. Without being able to estimate these associations, it is impossible to determine if the drugs* *are protective or harmful, greatly reducing the benefit of these associations to guiding care.*

We have discussed this issue more thoroughly in the limitation section of the Discussion (lines 374-386). Confounding by indication with antidepressants in relation to suicidality and mortality is complex. On the one hand an obese person with depression at high suicide risk is more likely to be prescribed antidepressants. However a person who self-harms by overdose with little intent to end their life is less likely to be prescribed antidepressants in case they took the antidepressants as an overdose. Given that the risk of self-harm is 40 times greater than suicide in people who are obese (reference 28 in the paper), then there is likely to be confounding by indication but the confounding is operating to both increase and decrease the likelihood of antidepressant prescribing.

In relation to mortality, suicide is a rare outcome compared to deaths from other causes such as cardiovascular disease and cancer. While a high risk of death by suicide would certainly increase the likelihood that antidepressants would be prescribed, a risk of mortality due to other possible causes would deter the prescription of antidepressants e.g. people with obesity who are prescribed opiate drugs, antidepressant prescribing would be discouraged because of the risk of mortality from respiratory depression.

*- The authors could consider dropping suicidality as an outcome or mentioning these results as a secondary outcome with unknown causality. Similarly, without understanding whether all cause mortality could be driven by an indication for the medication use, it is hard to interpret.*

We agree that that results of the analysis in relation to suicide and mortality is not straight forward because of confounding by indication that may be operating to both increase and decrease these risks. We have outlined these in the Discussion (lines 374 to 386). We describe these sources of confounding and what impact they may have in the discussion.

*- Alternatively, they could incorporate a systematically measured index of severity of depression or suicide ideation at baseline and follow-up to contextualize if there is actually a change relative to taking antidepressants.*

We agree that ideally we would include a measure of severity of depression but we have not have not found a suitable proxy measure for this. The reviewer suggested number of switches of antidepressants (which would be subject to immortal time bias given that the patient has to have survived long enough in order to have a switch) or number of consultations prior to the depression diagnosis (which would interact with the adjustments for glucose-lowering therapies, statins, CKD and cancer diagnosis). We have acknowledged that inability to account for depression severity is a significant limitation of our study (lines 374-386).

*Additionally, in their conclusion, the authors recommend discontinuation of antidepressants after 12 months, which could undermine the effect of antidepressants if they need to be restarted. Evidence of an increased risk of suicide in the short term following initiation of antidepressants guides limiting initiation of medications as much as possible, and encouraging discontinuation could prove harmful if it leads to increased initiation. It does not appear that the authors directly evaluate this strategy (such as with dynamic treatment regimens) to improve this recommendation nor due they present a formal risk/benefit analysis for discontinuation. This remains conjectural until more careful analysis is done and should be noted as such.*

We agree that the conclusion should be changed to a recommendation to clinically review each patient after being on antidepressants for 12 months. The clinical consideration is the need to manage depression and its risks by remaining on antidepressants or start an alternative treatment for depression e.g. psychological treatment and the risk of harm from antidepressants. We have altered our conclusion accordingly (lines 409-412).

*Overall, this is a good paper and provides some important evidence on CVD and fracture outcomes. There remain some crucial concerns in this paper that need to be addressed to improve the validity and utility of this study, or a need to focus it more directly on the outcomes less susceptible to confounding by indication.*

We thank the reviewer for these comments.

**RESPONSE TO REVIEWER 2**

*Major comments:

1. I would like to get a clearer picture on how the authors accounted for immortal time bias. It is not clear from the manuscript as it is written if it was appropriately considered.*

We used a time-dependent approach (for our exposure measure, antidepressant use) to control for immortal time bias (see papers [1] and [2] below for justification of this approach). This choice means that a patient who is not exposed to an antidepressant is treated as ‘unexposed’ (i.e. no antidepressant at that time period – our reference category ‘none’). We have now clarified this more fully in the statistical analyses section [Methods, lines 152-154].

[1] Zhou et al. (2005) <https://academic.oup.com/aje/article/162/10/1016/65057>

[2] Suissa (2007). <https://academic.oup.com/aje/article/167/4/492/233064>

*2. The use of forward selection strategy to adjust for confounders is not recommended and not reasonable given the sample size. A directed acyclic graph will be a more robust approach, or PS analysis.*

We would like to reassure the reviewer that the confounders/covariates were selected a priori as being conceptually related to the outcome of interest and not through forward selection. We did not draw a DAG specifically, but a similar approach was adopted. A PS analysis may have been possible, but would have been complex, given the different number of antidepressants under investigation. We would have needed to define the probability of being prescribed each individual antidepressant, and censoring on switch date, thereby compromising length of follow up. We are confident that our approach is sufficiently robust.

*3.There was no attempt to minimize confounding by severity, for example adjusting for number of visits in the prior year or number of switches*

We acknowledge that controlling for severity of depression is a limitation of our study. However, adding in number of switches would introduce immortal time bias (given that a person has to have survived/be event-free in order to have a switch). Similarly, there will be an interaction between number of consultations and glucose-lowering therapies, cancer, CKD and statins. We are reluctant to add in additional covariates at this stage as this was neither an a priori hypothesis nor in the approved ISAC protocol. However, we have acknowledged in the discussion that severity of depression was not possible as a limitation of the study (lines 374 to 386).

*4. Table 1 should be divided to include comparison group.*

Given that antidepressant exposure was treated as a time-dependent measure, there is no comparison group – only those who are not exposed during the time period. For example, a patient who enters the study at age 30 (i.e. first date of depression diagnosis) and who is initially prescribed no antidepressants for 1 year followed by sertraline for 1 year before leaving the practice contributes 1 year (age 30) to the “none” category (i.e. non-exposed) and 1 year (age 31) to the sertraline category. Therefore, the same patients contribute to both the reference and exposure category. We hope that this is now clearer with the new narrative added (Statistical analysis section lines 152-154).

*other:
how Weight and BMI index are also variably recorded?? this could be a major issue*

We agree that this is a limitation of the study, given that weight is variably recorded on the CPRD and is not missing at random. We have now added this to the limitations section (please see Discussion, lines 382-386) and have added in two new references to support this.

Of particular note, we included onlypeople with known

BMI measurement. Complete BMI reporting in UK GP surgeries,

which has improved over time, is known to be more common in older

individuals, females, people with lower socioeconomic status and

higher BMIs, and people with coexisting chronic conditions (43,44

*The conclusion should be rewritten to account for the major limitation, indication bias*

We agree. We have added the statement “However some of these results need to be considered cautiously as there was likely to be indication bias and residual confounding (lines406-407)” to our conclusion.

**EDITORIAL TEAM**

*We note that you have indicated that data from this study are available upon request.* *PLOS only allows data to be available upon request if there are legal or ethical restrictions on sharing data publicly. For information on unacceptable data access restrictions, please see http://journals.plos.org/plosone/s/data-availability#loc-unacceptable-data-access-restrictions.*

*In your revised cover letter, please address the following prompts:*

*a) If there are ethical or legal restrictions on sharing a de-identified data set, please explain them in detail (e.g., data contain potentially identifying or sensitive patient information) and who has imposed them (e.g., an ethics committee). Please also provide contact information for a data access committee, ethics committee, or other institutional body to which data requests may be sent.*

*b) If there are no restrictions, please upload the minimal anonymized data set necessary to replicate your study findings as either Supporting Information files or to a stable, public repository and provide us with the relevant URLs, DOIs, or accession numbers. Please see http://www.bmj.com/content/340/bmj.c181.long for guidelines on how to de-identify and prepare clinical data for publication. For a list of acceptable repositories, please see http://journals.plos.org/plosone/s/data-availability#loc-recommended-repositories.*

*We will update your Data Availability statement on your behalf to reflect the information you provide.*

There is a legal restriction on the authors sharing a de-identified data set from CPRD. There is on-going data collection of psuedoanonymised data from primary care practices, hospitals and public health as a national resource by CPRD on behalf of the National Institute for Health Research in England. The authors of the manuscript applied for, paid and were loaned the data for a set period of time after which they do not have legal access to the data. This has been set legally for the data protection of the participants and staff who collect the data because the information that is kept is very detailed, personal and sensitive including detailed patterned activity that over time might identify participants. There are also such safeguards in place because participants have their data entered into the database without formal consent; participants are only allowed to opt out so there are additional safeguards on access to the data in line with the United Kingdom Data Protection Act and GPDR. The data operates on this basis to maximise population coverage and data completeness to minimise selection, non-responder and attrition bias, thereby enhancing the generalizability of findings to clinical practice. Access to the database requires the development of a suitable protocol that is peer reviewed and approved by a board. A key principle is that there has to be a strong scientific case for the use of the data and that only the data is released to researchers with the expertise to utilise it to answer the research. The researchers also need to provide adequate assurances around data protection and data security.

However, researchers internationally can apply for access to any data including the same data that we used. The process for obtaining such access is outlined at <https://www.cprd.com/research-applications> (accessed 05/28/2020).
