# Supplementary material for: Safety of antidepressants in a primary care cohort of adults with obesity and depression
Source: PLoS One. 2021 Jan 29;16(1):e0245722. doi: 10.1371/journal.pone.0245722 (PMC7846000; doi:10.1371/journal.pone.0245722)
Supplement: Attachment — Submitted filename: Response to reviewers obesity and antidepressants.docx [file pone.0245722.s013.docx]

**Response to Reviewers**

Reviewer #1: This paper investigates adverse outcomes associated with anti-depressant use in a large cohort of overweight and obese adults. While there are some useful results from the study, there continue to be serious issues with the authors’ approach and conclusions.

The context of this paper, as a study among people with overweight or obese BMI, is overreached to suggest that the observed associations may exist among the general population (a group including people with underweight or normal weight BMI measures). The authors should make it much clearer that their results are observed in a population only including overweight and obese individuals. If the authors suggest that their study sample is representative, then their results are dissimilar from the literature exploring similar questions, which includes RCTs (PMID: 27367876, PMID: 32617669), and potential sources of confounding and bias should be further investigated. Even the randomized studies and meta-analyses of randomized studies that find short term risk, show much lower risk levels – which suggests that this estimate is only sensible if there is no increase in suicide among participants who are not overweight. This would be a major finding, if true, but more likely there is confounding by indication.

*Reply. We have added some text to the discussion and conclusion that further explains that the findings apply to obese and overweight people by:*

1. *Line 375 removing the reference to the representativeness of the sample to the general population.*
2. *Lines 403-404. Adding a statement that the results apply only to obese and overweight people not normal or underweight people.*
3. *Line 432. State that the clinical review at 12 months should be conducted in obese/overweight people.*

*The abstract makes no reference to the general population and its conclusion only mentions people who are obese or overweight.*

This paper would be stronger (and not subject to the bias it currently is) if the authors only included CVD, diabetes, and falls/fractures outcomes in their analyses/results. Suicide and all-cause mortality are subject to serious confounding by indication, which the authors agree with, and it is unclear what the value of giving a biased estimate is to the literature. Given that the study cohort is relatively young (mean age: 46, 25% under 34 years) and less likely to experience some of the outcomes related to age, premature death, such as due to suicide, should be a consideration in the methods and particular attention should be made to limit confounding in these analyses. The authors’ inability to account for severity of depression prevents them from attaining unbiased results from models that are subject to confounding related to this measure. Additionally, the inclusion of individuals taking multiple antidepressants (e.g. 2+ SSRIs) exacerbates the confounding by indication since individuals are requiring a second medication, potentially an indicator for severe depression and suicide ideation. The authors report very high hazard ratios for the risk of suicide among these individuals, which are likely severely confounded. All-cause mortality encompasses suicide, thus is subject to the same issues.

*Reply. We have removed all data for suicide/self-harm from the paper because we agree that there may be serious confounding by indication so there are now four outcomes. However, we think that all cause morality is extremely unlikely to be seriously confounded by indication because only 19 (0.7%) of 2717 deaths were attributed to suicide taking all sources of information available to us from the database. Even if there were a substantial number of other deaths due to suicide that were attributed to other causes, then it is still unlikely that there would be serious confounding. Another database study cited in the paper also founds that suicide is a relatively uncommon cause of death in overweight or obese with depression. Unfortunately apart from suicide we cannot say what the specific causes of death were. Given that the SMR for cardiovascular death in depression is approximately 2, obesity is also a risk factor for cardiovascular death and trials of antidepressants have not reduced cardiovascular events, then it is plausible that many of these young deaths might be due to cardiovascular causes.*

*In summary we have removed references to suicide and self-harm from the abstract, introduction, aims, method, results, Figures 1 and 2, Table 2 and discussion. Under all-cause mortality results we have added the data that there were 19 suicides out of 2717 deaths (lines 214-215). We have acknowledged the possibility that there may be more deaths from suicide than are reported but have also stated that since suicide is a rare outcome in this and other database studies of overweight and obese people with depression (reference number 27), then we consider the risk of serious confounding by indication to be low (lines 388-400). We acknowledge that we do not know the cause of death other than suicide in the participants of this study and this would be important to study.*

The authors slightly modified their conclusion; however, their recommendation for possible discontinuation of antidepressants is an unsubstantiated claim based off their study. An interesting study could have been designed on the benefits or risk of discontinuing medication, where there is an absence of randomized evidence. This conclusion could even prove to be harmful, as there is evidence of adverse impact on risk of suicide in the short-term following antidepressant initiation and current recommendations suggest minimizing the amount of times initiating antidepressants. The conclusion should be further adjusted to not suggest discontinuation of antidepressants.

*Reply. We have further modified our conclusion (lines 432-436) that people who are overweight or obese with depression who are taking antidepressants should be clinically reviewed to consider the balance of risks and benefits of continuing antidepressants in view of the risks from both the antidepressants and depression. We have also added that alternative effective treatments for depression such as psychological treatments should be considered if they are available. We think our data support such a conclusion and we are not advocating that on the basis of this data that everyone should stop taking antidepressants after one year. Furthermore in many countries, including the UK, these conclusions would not be controversial as they are recommended practice for people with mental disorder whatever their weight. Access to psychological treatments is readily available. However we know that this is not the case in many countries so we hope that we have now struck the correct clinical balance in our conclusion.*

Overall, this paper contains important content related to antidepressants and CVD, diabetes, and falls/fractures, but the authors present biased estimates that need to be addressed before this paper provides a beneficial contribution to the literature.

*Reply. We hope the above addresses these concerns.*

Reviewer #2: If confounders were selected as a priori and adjusted for in full models and not using forward selection, then this should be stated and considered the superior method.

*Reply. We thank the reviewer for pointing this out. We can confirm that we adjusted for the covariates selected a priori in all models and retained the covariates even if they were not statistically significant in line with the a priori selection. We have now clarified this (Line 155–157).*

Have the authors made all data underlying the findings in their manuscript fully available? The PLOS Data policy requires authors to make all data underlying the findings described in their manuscript fully available without restriction, with rare exception (please refer to the Data Availability Statement in the manuscript PDF file). The data should be provided as part of the manuscript or its supporting information, or deposited to a public repository. For example, in addition to summary statistics, the data points behind means, medians and variance measures should be available. If there are restrictions on publicly sharing data—e.g. participant privacy or use of data from a third party—those must be specified.

*Reply. We have already explained that there are restrictions on publicly sharing data because the data is owned by a third party and licensed for a period of time for a fee to complete a defined protocol of work that is peer reviewed and agreed between the guardians of this publicly owned data and the research team. Anyone who wishes to check or use the data in this study would need to apply to the guardians of the data and go through a similar process and pay an appropriate fee to obtain access to such data. We have outlined these procedures in our last revision. Unfortunately we are unable to modify these procedures or provide greater access.*
